# System immunology-based identification of blood transcriptional modules correlating to antibody responses in sheep

**DOI:** 10.1038/s41541-018-0078-0

**Published:** 2018-10-03

**Authors:** Roman Othmar Braun, Livia Brunner, Kurt Wyler, Gaël Auray, Obdulio García-Nicolás, Sylvie Python, Beatrice Zumkehr, Véronique Gaschen, Michael Hubert Stoffel, Nicolas Collin, Christophe Barnier-Quer, Rémy Bruggmann, Artur Summerfield

**Affiliations:** 1 Institute of Virology and Immunology, Mittelhäusern, Switzerland; 2 Graduate School for Cellular and Biomedical Sciences, University of Bern, Bern, Switzerland; 3 Vetsuisse Faculty, Department of Infectious Disease and Pathobiology, University of Bern, Länggassstrasse 122, 3001 Bern, Switzerland; 4 Vaccine Formulation Laboratory, Department of Biochemistry, University of Lausanne, Lausanne, Switzerland; 5 Interfaculty Bioinformatics Unit and Swiss Institute of Bioinformatics, University of Bern, Bern, Switzerland; 6 Division of Veterinary Anatomy, University of Bern, Bern, Switzerland

## Abstract

Lacking immunogenicity, inactivated vaccines require potent adjuvants. To understand their effects, we used a system immunology-based analysis of ovine blood transcriptional modules (BTMs) to dissect innate immune responses relating to either antibody or haptoglobin levels. Using inactivated foot-and-mouth disease virus as an antigen, we compared non-adjuvanted to liposomal-formulated vaccines complemented or not with TLR4 and TLR7 ligands. Early after vaccination, BTM relating to myeloid cells, innate immune responses, dendritic cells, and antigen presentation correlated positively, whereas BTM relating to T and natural killer cells, as well as cell cycle correlated negatively with antibody responses. Interestingly, BTM relating to myeloid cells, inflammation and antigen presentation also correlated with haptoglobin, but in a reversed manner, indicating that acute systemic inflammation is not beneficial for early antibody responses. Analysis of vaccine-dependent BTM modulation showed that liposomal formulations induced similar responses to those correlating to antibody levels, while addition of TLR ligands reduced myeloid cells, inflammation and antigen presentation BTM expression despite promoting antibody responses. Furthermore, this vaccine was more potent at downregulating T and natural killer cell BTM. When pre-vaccination BTM were analyzed, we found that high vaccine responders expressed higher levels of cell cycle and myeloid cell BTMs as compared with low responders. In conclusion, we have transferred human BTM to sheep and identified early vaccine-induced responses associated with antibody levels or unwanted inflammation. Such readouts are applicable to other veterinary species and very useful to identify efficient vaccine adjuvants, their mechanism of action, and factors related to low responders.

## Gene expression: developing a platform to identify vaccine effects

A next-generation method to identify genetic changes in response to vaccination opens up the detailed study of vaccine and adjuvant effects. Researchers led by the Swiss University of Bern’s Artur Summerfield inoculated sheep with foot-and-mouth disease (FMD) virus antigens, either alone, adjuvanted (in an immunity-boosting formulation), or adjuvanted with molecules speculated to help generate long-term immunity against FMD. The team found only the latter group stimulated host immunity to a level considered protective, albeit with high variability. Differences in gene expression were identified that correlated to virus-neutralizing responses. The team used a powerful bioinformatics tool, measuring ‘blood transcriptional modules (BTM),’ to identify ovine specific immune processes. The BTM approach offers an advantageous way to identify vaccine and adjuvant effects down to the genetic level. This study also offers data to inform future FMD vaccine development efforts.

## Introduction

In the past, vaccines were developed empirically requiring many animal studies with a long-term follow-up to assess vaccine efficacy. Furthermore, although the mechanisms of many vaccine adjuvants at the molecular and cellular level are clear, in vivo protection is much more complex and still difficult to understand and predict. Systems immunology offers powerful approaches towards dissecting the early innate immune response relating to potent adaptive immune responses in human.^1–3^ This enabled to identify genes expressed in peripheral blood leukocytes within the first days after vaccination, which correlated to CD8 T-cell or antibody responses.^2,3^ Nevertheless, a main problem with this relatively simple approach was the high heterogeneity of individual responders and the difficulty to understand immunological processes from individual gene information. For these reasons, a much more powerful analysis of transcriptomic data was developed by identifying gene sets such as blood transcriptional modules (BTMs) through large-scale data integration.^1,4^ To this end, Li et al.^1^ established human blood transcriptomes from 540 data series containing over 32,000 samples obtained from many disease-related studies to identify master networks of genes co-regulated and interacting in peripheral blood immune system during many physiological and pathological conditions^1^. Certain BTM were demonstrated to correlate with adaptive immune responses in a vaccine type-specific manner enabling conclusions on immunological processes associated with desired vaccine responses.^1^

For foot-and-mouth disease (FMD), a devastating viral infection affecting cloven-hooved animals, traditional vaccine approaches are based on the formulation of inactivated antigen with conventional adjuvants such as oil-in-water emulsions.^5^ In countries free of FMD, besides culling infected herds, vaccines can have an important role in the control of outbreaks. Nevertheless, the ability to induce early protection is an essential element of such vaccines. A problem associated with the elimination of FMD from endemic areas is the fact that current vaccines offer protection with a very limited duration of immunity.^6^ A potential to improve such vaccines would be to enhance their ability to activate the innate immune system by targeting Toll-like receptors (TLRs), which can promote T-cell help and differentiation of B lymphocytes into memory or long-lived plasma cells.^7^ TLR are a family of pathogen recognition receptors (PRR) typically expressed at high levels on specialized antigen-presenting cells and are central for alerting the innate immune system in case of infection. Cell surface TLRs, such as TLR4, recognize microbial pattern molecules such as bacterial lipopolysaccharides, whereas endosomal TLRs such as TLR7 and TLR9 sense single-stranded RNA or microbial unmethylated DNA with many CpG motifs, respectively. In fact, TLR4 and TLR7 ligands were shown to strongly enhance antibody responses in the mouse model,^7^ and were therefore selected for the present study.

Based on this, one aim of this work was to determine the potential of such TLR ligands to improve the immunogenicity of an inactivated vaccine in large animals using an FMD vaccine as a model. We used sheep as a model for an ungulate that is naturally susceptible to FMD virus (FMDV). Although in western countries small ruminants usually are not considered in vaccination program, the immune system of sheep and cattle are relatively similar and the use of sheep requires less space and financial resources. Furthermore, in certain areas of the world small ruminant represent the main FMD-susceptible livestock and can play an important role for spreading the infection during outbreaks.^8–10^

Our second important aim was to transfer human BTMs to the sheep model and identify gene sets correlating with antibody levels as well as vaccine side effects. We also successfully employed this bioinformatics pipeline to characterize vaccine-adjuvant-dependent modulation of the immune responses as well as the pre-vaccination transcriptome associated with high immune responses.

## Results

### Antibody responses

**Fig. 1:**
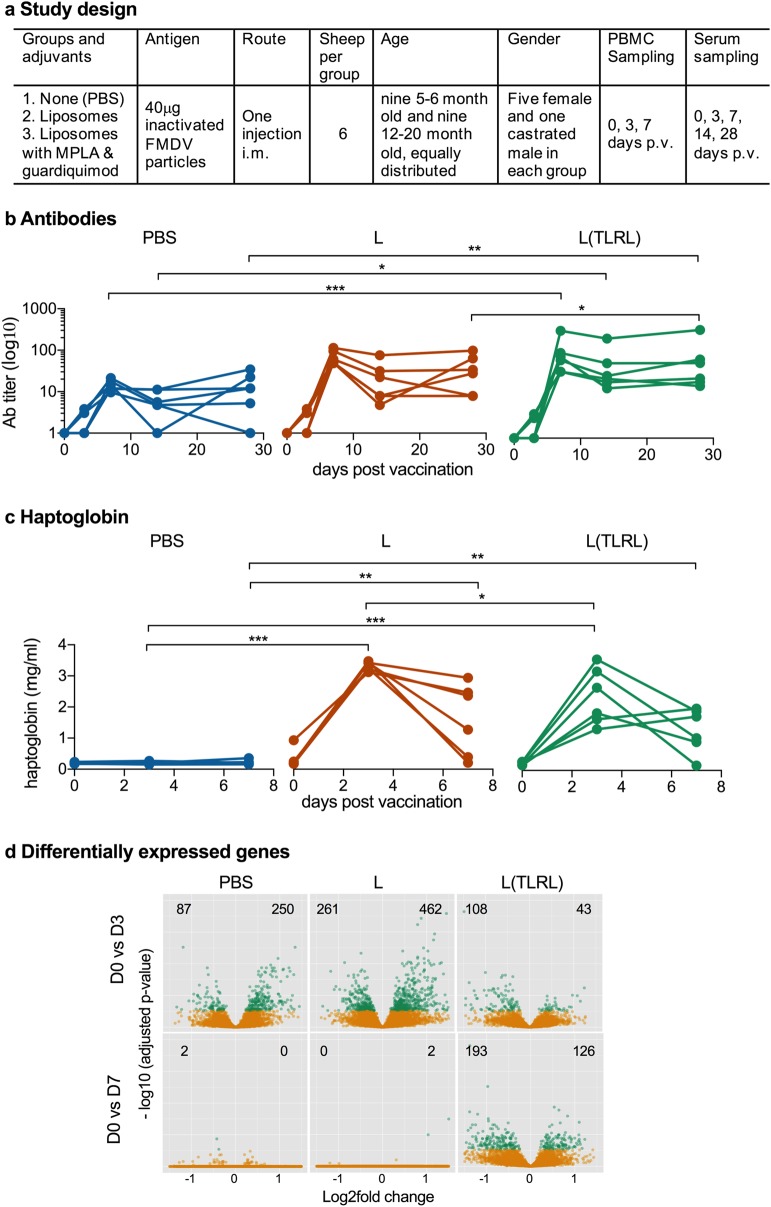
Neutralizing antibodies, haptoglobin and differentially expressed genes in PBMC induced by vaccination. **a** Study design. **b** Serum neutralizing antibody titers following vaccination of sheep with antigen in phosphate buffered saline (PBS), antigen formulated in liposomes (L) and antigen formulated in liposomes with TLRL (L(TLRL)). **c** Vaccine-induced haptoglobin responses. **d** Vulcano plots showing differentially expressed genes which were up- or downregulation on day 3 (D3) and day 7 (D7) compared with before immunization, respectively. Green dots represent significant genes (*p* adjusted < 0.05). Digits in the plots indicate the number of significantly down or upregulated genes. In **b** and **c**, statistical significant differences between two groups were calculated using unpaired two-way ANOVA with repeated measures followed by Tukey’s multiple comparisons test (***p < 0.001; **p < 0.002; *p < 0.033)

Three groups of sheep were vaccinated with FMDV antigen formulated either in buffer (PBS), in liposomes (L) or in liposomes containing TLR4 and TLR7 ligands (L(TLRL)), (Fig. 1a) and the ability of the vaccines to induce an early neutralizing antibody response was tested (Fig. 1b). For the physicochemical characterization of the vaccines see Supplementary Fig. 1. All groups responded to vaccination when day 0 p.v. (D0) was compared to D7, D14 and D28 using ratio-paired T tests (*p* < 0.05). Only the L(TLRL) group was able to significantly enhance the antibody levels above the levels of the PBS group, at D7, D14 and D28 (Fig. 1b). At D28, the L(TLRL) group had significantly higher levels of antibodies compared to the L group (Fig. 1b).

### Vaccine-induced haptoglobin and transcriptional modulations

As liposomal formulations caused lameness in some animals for 1–2 days after injection, we also quantified systemic inflammation by measuring serum haptoglobin, an acute-phase protein induced in the liver by pro-inflammatory cytokines,^11^ and which represents a sensitive marker of inflammation following sheep vaccination.^12^ Our results demonstrate that liposomal formulations were indeed associated with an acute-phase response peaking at D3 and still detectable in most animals at D7 (Fig. 1c). Interestingly, the TLRL did not enhanced but actually reduced this response (*p* = 0.038 for L versus L(TLRL) at D3).

Also at the transcriptional level, the liposomal formulations caused a stronger perturbation of the immune system, but this was dampened by the addition of TLRL (Fig. 1d). For example, when comparing and peripheral blood mononuclear cells (PBMCs) before and at D3 (D0 vs. D3), PBS induced the significant upregulation of 250 genes, L of 462 genes, and L(TLRL) only of 43 genes. Nevertheless, at D7, the TLRL-adjuvant group had clearly the most up- or downregulated transcripts (Fig. 1d and Supplementary Fig. 2). We found 150 common genes between the L and L(TLRL) groups, and 120 common genes for the L and PBS groups and seven common genes for all three formulations.

### Individual transcripts correlating with neutralizing antibody titers

Individual changes in gene expression were calculated and correlated with the SNT on day 7, 14, and 28 p.v. (Supplementary Fig. 3). For the D0 vs. D3 differentially expressed genes, 26 transcripts (ZNF646, STIM2, C9orf3, NPEPPS, SDR39U1, HEXDC, NUCB2, GHDC, LSR, MYO1E, TRAPPC6A, DCAF12, ABCC2, ZDHHC24, TRIM41, GZMA, NAGK, GMNN, OLFML3, MYO7A, TNFSF13B, GGA3, ASB8, ARNTL, GNG3, PLA2G12A) were identified to correlate with the SNT of all three time points (7, 14, and 28 days). For the D0 vs. D7 differentially expressed genes, six transcripts were found to correlate with SNT of all three time points (CHPT1, RPL12, RNASEL, CCDC93, MFAP2, MYO1E). Only MYO1E was correlating at D3 as well as D7 with the SNT at all time points.

### Translation of human BTM to the sheep

While some of the genes identified above are clearly linked to antibody responses such as TNFSF13B (B-cell-activating factor), the potential involvement of most genes in promoting antibody responses remained elusive. We therefore employed gene set enrichment (GSEA) using BTM developed in the human system. These have been demonstrated to provide a comprehensive picture of human immune responses correlating with adaptive immune responses induced by different classes of vaccines.^1^ To this end the gene composition of the BTM was adapted to sheep by replacing genes known to differ in name for human and sheep such as those belonging to the MHC, CD1, TCR, Ig and IFN families with sheep homologues. In addition, we manually annotated many sheep genes focussing on those represented in many BTM. This resulted in a median coverage of 88.2% (Supplementary Fig. 4). The list of all BTM with their gene compositions can be downloaded from the Supplementary Data.

**Fig. 2:**
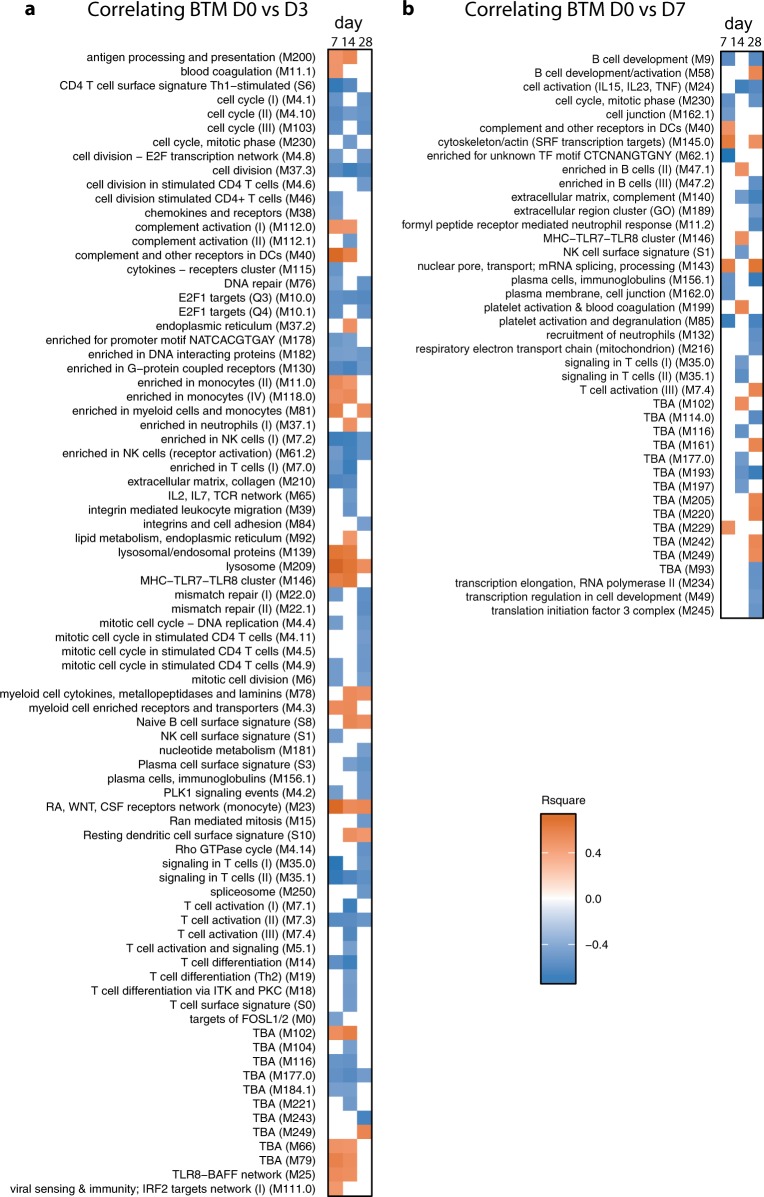
BTM correlating to serum neutralizing antibody titers. **a** Heatmap showing the correlation of D3 BTM with antibody levels on day 7, 14, or 28 after immunization. Animals from all groups were included (*n* = 18, cutoff *p* < 0.05). **b** Heatmap showing the correlation of D7 BTM with antibody levels in analogy to **a**

### BTM correlating with antibody responses

The correlation of BTM with antibody responses measured at 7, 14, and 28 days p.v. were determined for the D3 and D7 transcriptome (Fig. 2). Although some BTM correlated only with one of the time points p.v., other correlated with two or all of the time points analyzed. This was expected as some animals with low early antibody levels caught up at day 28 p.v. (Fig. 1b). Nevertheless, for D3 the BTM M209 (lysosome) and M23 (RA, WNT, CSF receptor network) positively correlated with the SNT at all time points. Furthermore, despite the variation in antibody responses over time, a number of BTM were found that negatively correlated to antibody responses measured at all three time points. These were mainly BTM related to cell proliferation (M4.10, M37.3, M10.0, M182) and T/natural killer (NK) cells (M7.2, M61.2, M7.3).

**Fig. 3:**
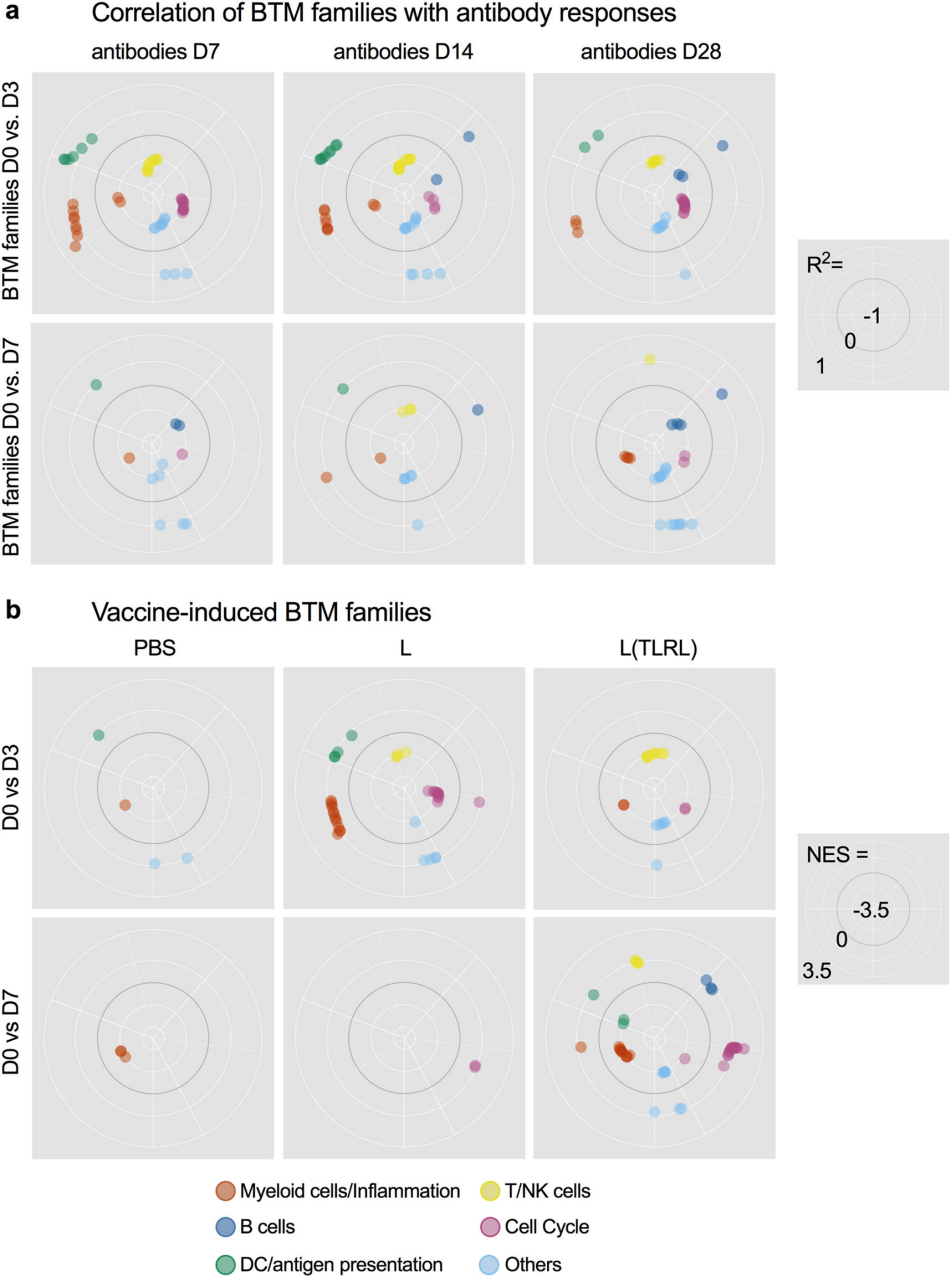
BTM families correlating with antibody responses and induced by the vaccines. BTM families were created as described in Table 1. **a** The polar plots show the correlation coefficients for BTM of the module families with significant positive or negative correlation to neutralizing antibody levels in serum (*p* < 0.05). BTM for the D3 (upper plots) and D7 (lower plots) transcriptome are shown. **b** Vaccine-dependent BTM modulations were calculated as normalized enrichment scores (NES) using GSEA. The significantly (*p* < 0.05) modulated BTM were grouped as BTM families as in **a**. The data are shown was calculated for each vaccine group with the D3 (upper plots) and D7 (lower plots) transcriptome

Antibody-correlating D7 BTM were reduced in numbers (Fig. 2). Nevertheless, some BTM were found again, including M146 (MHC-TLR7-TLR8 cluster, positive correlation), M40 (complement and other receptors, positive correlation), M35.0/M35.1 (signaling in T cells, negative correlation) and M230 (cell cycle, mitotic phase, negative correlation, S1 (NK cell surface signature, negative correlation), S3 (plasma cell surface signature, negative correlation) and a number of unnamed BTM (M102, M116, M177 and M249). BTM 7.4 (T cell activation, positive correlation) was the only BTM found with inverse correlation).

To enable an easier interpretation of the BTM results, we classified them into six BTM families (Table 1 and Supplementary Excel file “BTM composition and families”). To this end, BTM were attributed to immune cell types or immunological processes, for which we decided to make large families to obtain a simplified synopsis of early immune responses correlating to antibody titers. As an example, the BTM family “myeloid cells/inflammation” was composed of all BTM’s relating to monocytic cells, neutrophils, and other elements of inflammatory reactions. We also included BTM related to coagulation and complement activation, as these are processes induced during inflammation (Table 1). The result of such an analysis for the correlating BTM is shown in Fig. 3 and demonstrate that the D3 dominant BTM families positively correlating with antibody responses were “myeloid cells/inflammation” and “DC/antigen presentation,” whereas the BTM families “T/NK cells” and “cell cycle” were the most prominent to negatively correlate. This dominance was seen in terms of the number of correlating BTM’s attributed to the family for each of the analyses. The polar plots created for day 7 did not enable the identification of clearly dominant BTM families because of the reduced number of correlating BTMs. Interestingly, at this later time point the BTM family “myeloid cells/inflammation” was negatively correlating with antibody levels.

**Table 1:** Definition and composition of BTM families

BTM families	Immune cells and processes	BTM included
Myeloid cells/inflammation	Myeloid cells, innate/inflammatory response (inflammatory cytokines and chemokines), leukocyte migration, coagulation and platelet activation, complement activation	M0, M4.3, M4.15, M11.0, M11.1, M11.2, M16, M21, M23, M24, M27.0, M27.1, M29, M33, M37.1, M38, M39, M45, M53, M59, M73, M78, M81, M85, M86.0, M86.1, M88.0, M91, M112.0, M112.1, M115, M118.0, M118.1, M132, M163, M199, S4, M30, M32.0, M32.1, M37.0, M42
DC/antigen presentation	DC surface (resting and activated), antigen processing and presentation, DC activation	M5.0, M25, M28, M40, M43.0, M43.1, M50, M64, M67, M71, M87, M95.0, M95.1, M119, M138, M139, M146, M165, M168, M200, M209, S5, S10, S11
T/NK cells	NK and T-cell surface, differentiation, activation, signaling, co-stimulation, and proliferation	M4.5, M4.6, M4.9, M4.11, M5.1, M7.0, M7.1, M7.2, M7.3, M7.4, M12, M14, M18, M19, M35.0, M35.1, M36, M44, M46, M52, M61.0, M61.1, M61.2, M65, M126, M157, M223, S0, S1, S6, S7
B cells	B cells, BCR signaling, B-cell development and differentiation, plasma cells, immunoglobulins	M9, M47.0, M47.1, M47.2, M47.3, M47.4, M58, M69, M83, M123, M156.0, M156.1, M217, S2, S3, S8, S9, M152.0, M152.1, M152.2, M182
Cell cycle	Cell cycle, proliferation, DNA repair, nucleotide and amino acid metabolism, splicing	M76, M103, M4.10, M4.12, M4.0, M4.1, M4.2, M4.4, M4.7, M4.8, M6, M8, M10.0, M10.1, M20, M22.0, M22.1, M31, M32.2, M32.4, M37.3, M49, M144, M167, M175, M181, M230, M250, M15, M143, M154.0, M169
IFN type-I	Nucleic acid sensing, IFN type-I induction, and IFN response signature	M13, M68, M75, M111.0, M111.1, M127, M150, M158.0, M158.1

**Fig. 4:**
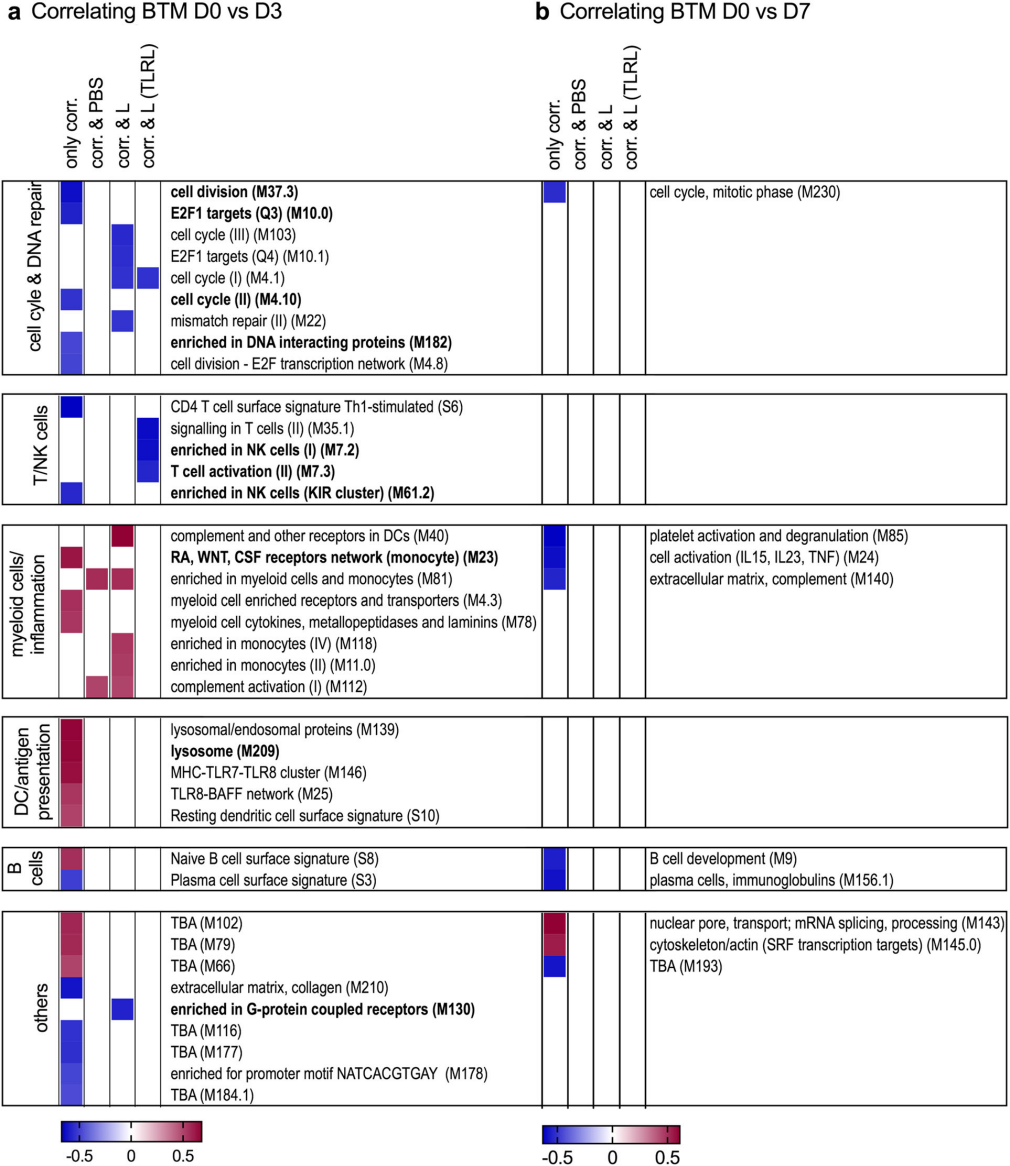
Relationship of BTM correlating to antibody levels and their induction by the vaccines. The heatmap shows the average correlation coefficient *r*^2^ from BTM correlating to antibodies at least at two different time points. BTM correlating to antibody levels at all time points (day 7, 14 and 28) are indicated in bold letters. BTMs were allocated to BTM families defined in Table 1 and grouped as BTM which were not induced by any of the vaccine group (only corr.), induced by the PBS-based vaccine (corr. & PBS), the L (corr. & L) or the L(TLRL) vaccines (corr. & L(TLRL); for data on vaccine-induced BTM see Supplementary Fig. 4). The blank cells indicate that there was no significant correlation for this time point post vaccination

### Vaccine-induced BTM’s

We next calculated BTM’s significantly induced by the different vaccines with the aim to compare these responses with the antibody-correlating BTM, and to determine the effects of formulation and TLRL. The results are shown as BTM families in Fig. 3b and as heatmap for individual BTM in Supplementary Fig. 5. In the PBS group, only few BTM were significantly modulated at both D3 and D7. On D3 the liposomal formulations strongly promoted “myeloid cells/inflammation” and “DC/antigen presentation” but downregulated “T/NK cells” and “cell cycle” BTM families, creating a picture resembling the correlation data shown in Fig. 3a. Interestingly, the addition of the TLRL abrogated the induction of “myeloid cells/inflammation” and “DC/antigen presentation” BTM. In contrast, on D7 we only found many significantly modulated BTM in the L(TLRL) group. While “myeloid cells/inflammation” and “DC/antigen presentation” were downregulated, the “B cell” and “cell cycle” family was found to be upregulated only in the TLRL group.

As a main aim of the present study was to identify modules induced by our vaccine formulation and correlating with antibody response we combined the data of Fig. 3 to search for BTM which would correlate to the antibody response and also be induced by a particular vaccine. This approach was also used to identify BTM correlating independent from the vaccine-induced responses. Figure 4 shows average correlations coefficients for BTM which significantly correlated with antibody responses found at least for two different time points. The BTM were grouped in BTM which were not found to be induced by a vaccine (“only corr.”), which were induced by the PBS-formulated vaccine (corr & PBS), by the liposomal formulation (corr. & L) or by the TLRL-adjuvanted liposomal vaccine (corr. & L(TLRL)). On D3, for many BTM such as those belonging to the “DC/antigen presentation” and “B cells” families, correlating BTM were actually not identified to be significantly induced by the vaccines, indicating that these could more reflect differences in the host response to the vaccination. The L vaccine group induced many correlating “myeloid cells/inflammation” BTM, while the L(TLRL) group was potent at downregulating “T/NK cell” BTM (Fig. 4a). Overall, this demonstrated a strong immunomodulatory effect of the TLRL. On D7, none of the correlating BTM were not found to be induced by the vaccines (Fig. 4b), again indicating that these BTM reflect differences in the host responses to the vaccines.

### Impact of vaccines on peripheral blood mononuclear cell populations

Considering that changes in many BTMs could have been the result of a modulation of relative frequencies of cell populations we quantified CD14+ and CD16+ monocytes as well as CD4+ and CD8+ T cells in D0 and D3 PBMC. Only a significant reduction of CD8+ T cells was found in the L and L(TLRL) groups (Supplementary Fig. 6), indicating that the vaccine-induced BTM modulation is not solely reflecting changes in the cellular composition of the PBMC.

### BTM correlating to the acute phase response

**Fig. 5:**
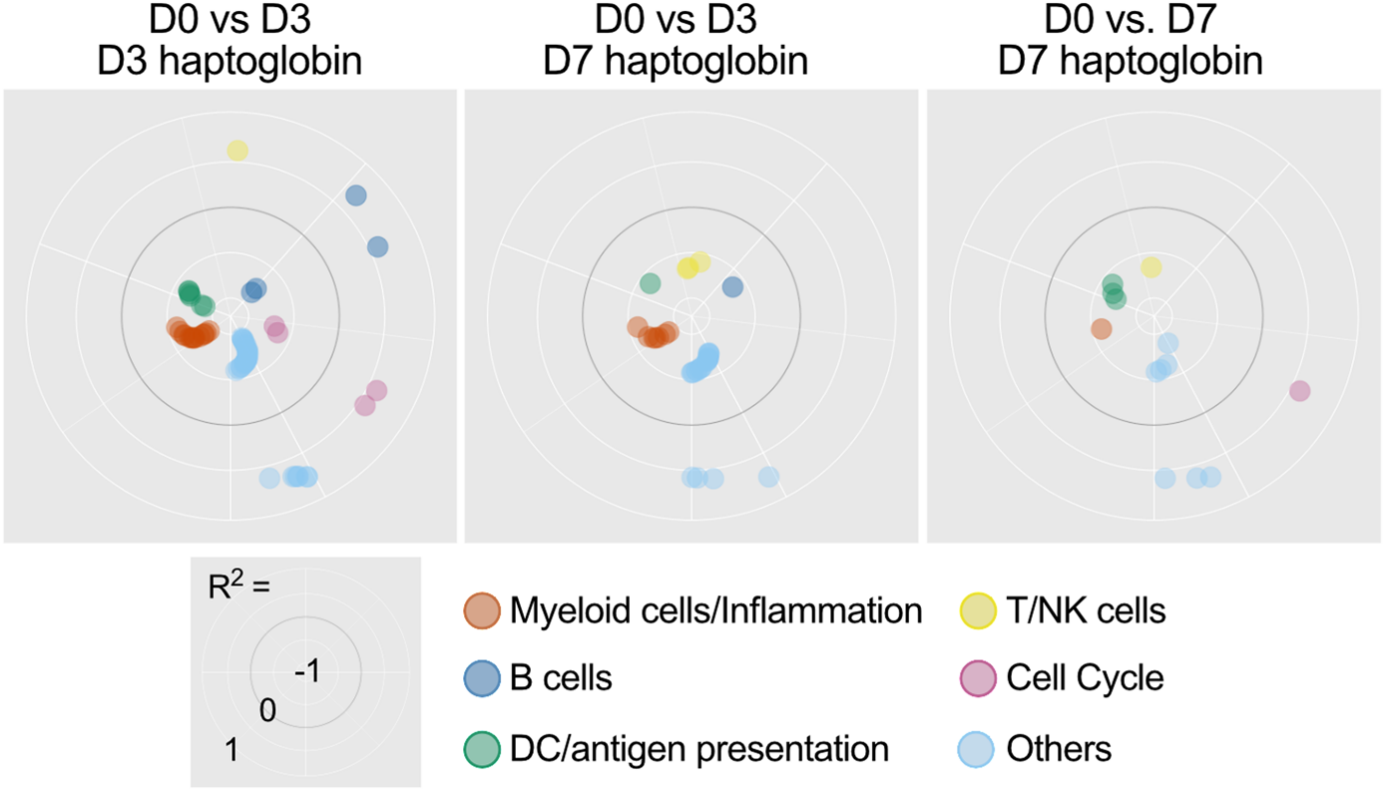
BTM families correlating to vaccine-induced haptoglobin levels. BTM families were created as described in Table 1. The polar plots show correlation coefficients for module families with significant (*p* < 0.05) positive or negative correlation to serum haptoglobin levels. BTM families for the D3 (upper plots) and D7 (lower plots) transcriptome are shown

To differentiate innate immune responses associated with antibody responses or unwanted inflammation, we calculated the correlation of BTM with the haptoglobin responses (Fig. 5 and Supplementary Fig. 7). We excluded the PBS group as no haptoglobin responses were found in these animals (Fig. 1c). For the D3 transcriptome, many negative correlations were found for the “myeloid cells/inflammation” and “DC/antigen presentation” but positive correlation for the “T/NK cells” and “cell cycle” BTM. In addition, a few “B cells”, “cell cycle” and “others” BTM families were negatively correlated. For the haptoglobin responses on day 7 p.v., the dominant modulation was again in the “myeloid cells/inflammation” BTMs. For the D7 transcriptome the number of correlations was markedly reduced. If BTM families are compared with those correlating with antibody levels measured at D3, we found that “myeloid cells/inflammation” and “DC/antigen presentation” BTM families were regulated in an opposite manner. When looking at individual BTM (D0 vs D3), only seven out of 93 correlated in the same direction to the antibody responses (M130, M61.2, M210, M65, M39, M14, M0, Supplementary Fig. 7 and Fig. 2). Five correlated in an opposite manner (M11.1, M22.0, M181, M23, M111.0). These results indicate that the host responses associate with the acute phase response are largely not linked to those resulting in potent antibody responses.

### Pre-vaccination BTM associated with antibody levels

**Fig. 6:**
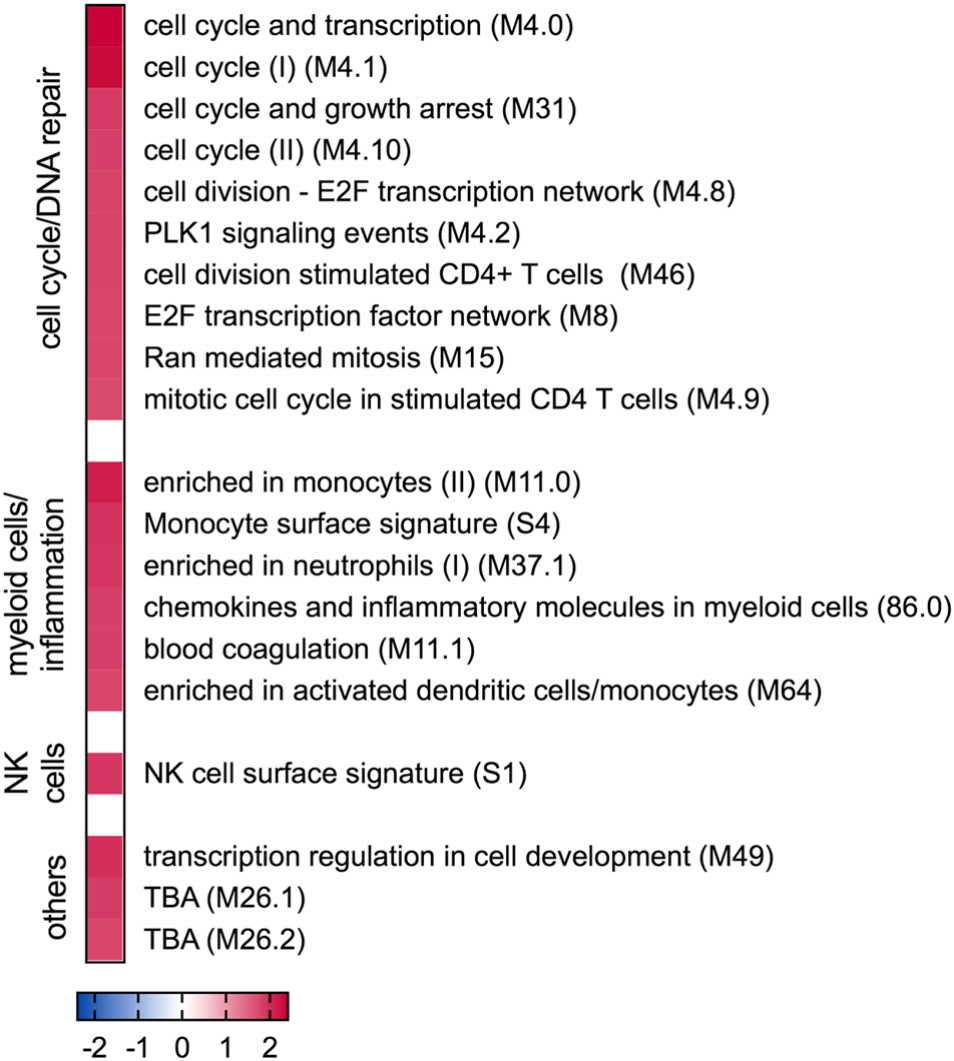
Pre-vaccination BTM associated with antibody levels. **a** GSEA was used to determine the BTM enriched in “high responders” (*n* = 6, red) and “low responders” (*n* = 6, blue). The colors indicates the NES. **b** Heatmap for the BTM “monocyte surface signature” (S4) as an example of the analysis in a. Each square represents the indicated gene expression for individual animals (same color code as in **a**)

Considering the heterogeneity of vaccine-induced antibody responses (Fig. 1b), we tested if certain BTM measured before vaccination would be associated with higher levels of antibody response. To this end, in each vaccine group animals having an SNT at day 28 of > 50% above the median were defined as “high responders” (*n* = 6) and those with < 50% of the median as “low responders” (*n* = 6). A comparison of high and low responders using GSEA demonstrated that 10 BTM related to cell cycle and DNA repair, four BTM related to myeloid cells, two BTM related to inflammation and innate immune responses, one to NK cells were expressed at relatively higher levels in the “high responders” (Fig. 6). No BTM was higher in the “low responder” animals.

## Discussion

The present study adapted BTM originally developed for human blood to sheep PBMC to understand vaccine-induced responses correlating with antibody and acute-phase responses. For efficient delivery to antigen-presenting cells, we employed synthetic liposomes as vaccine platform and tested the effect of a combination of TLR4 and TLR7 ligands on antibody and acute-phase protein response. These ligands were selected based on their synergistic potentiation of antibody responses in a mouse model.^7^ Specifically, the TLR7 ligand Gardiquimod was selected based on its ability to induce proliferation and induce IFN-α in PBMC,^13,14^ and the TLR4 ligand monophosphoryl lipid A (MPLA) based on its potent triggering of nuclear factor-κB signaling, which promotes pro-inflammatory responses in mononuclear phagocytes.^15,16^ Furthermore, MPLA is clinically approved and was also recently reported to induce a similar response in sheep compared with human PBMC.^17^ The vaccination of sheep was successful in terms of induction of neutralizing antibody titers to levels likely associated with protective immunity.^18^^–^^20^ As anticipated, the addition of the TLRL significantly enhanced the responses. Nevertheless, it is clear that the present vaccine still requires significant improvements, and thus future studies focussing on the effects of formulation, TLR ligand selection and dose in sheep are needed.

Interestingly, although the increase in antibody responses induced by the TLRL was weak compared to previous studies in mice,^7^ it had a clear effect on the expression of individual genes. Strikingly, the overall perturbation of gene expression was reduced at D3 in terms of differentially up- or downregulated genes when compared with the L group. Furthermore, on day three p.v., the PBS group shared many upregulated genes with the L, but not with the L(TLRL) group. At day 7, this was even more evident in that differentially regulated genes were found almost exclusively in the TLRL group. Also, comparing the vaccine groups using BTM confirmed clear effects of the TLRL. The reduction of upregulated “myeloid cells/inflammation” and DC/antigen presentation” BTM at D3 together with the above reduced perturbation of individual genes and the reduced induction of haptoglobin indicates an anti-inflammatory effect of the TLRL. Future studies are required to understand how this is induced and if it is an effect of one of the TLRL or their combinations. The fact that at D7 the L(TLRL) group showed the strongest perturbations, both at the individual gene and BTM level, demonstrates that this is not a simple suppressive effect but that rather long-lasting immunomodulatory circuits are induced.

The analysis of genes correlating positively or negatively with antibody titers revealed a relatively large number of genes. We focused mainly on those found to correlate at least for two different time points to increase the chance to have truly relevant genes. Nevertheless, for most of the correlating genes, their role in immune responses is unclear, with the exception of TNFSF13B representing BAFF. Furthermore, only few of the correlating genes matched those identified in PBMC from humans vaccinated with an inactivated viral vaccine.^2^ For these reasons, we employed the BTM as a much more powerful bioinformatics analysis of transcriptomic data. BTM represent master networks of immunological genes strongly co-regulated and interacting during many physiological and pathological conditions.^1^ This approach permitted to identify many BTM expressed on D3 and D7 correlating with antibody responses on day 7, 14, and 28 p.v. Remarkably, many of the correlating BTMs were also found in human vaccination studies using protein-based or inactivated vaccines. Prominent amongst these are positively correlating BTM relating to monocytes such as M11.0, M23, and M118,^1,21–23^ to complement activation such as M112,^1,23^ to TLR/antigen presentation such as M146, M25^1,21,23^. Among prominent negatively correlating modules appearing in both human and sheep vaccination studies is the NK cell signature BTM M61.1, M61.2, and M61.3^18,20,21^. In sheep, many T-cell modules including CD4 T-cell BTM (M4.6, S6) as well as general T-cell activation modules (M7.X) were negatively correlated with antibodies. Nevertheless, the various reports on human studies indicate that this can strongly vary depending on the time point being analyzed, the vaccine employed, the number of vaccinations, and the age,^1,23,24^ although M7.1 and M7.2 were often negatively correlated with antibody response also in human studies.^1,21,23,24^ Our phenotyping data confirmed that at least for the CD8 T cells the liposomal vaccine formulations induced a relative reduction of circulating T cells. For the cell cycle modules which were also among the BTM with a negative correlation in sheep, this was rarely found in the human studies using protein-based vaccines, indicating possible species differences or alternatively a specific effect of the liposomal vaccine employed in the present study. Clearly, the correlating BTM response strongly depends on the type of vaccine.^1^ Altogether, we propose that the common correlating BTM found for several different inactivated/protein-based vaccines in both species could be particular robust measurements. From our data, we also conclude that measuring the transcriptome early after vaccines provides more correlating BTM and, therefore, more information regarding the effects of the vaccine on the immune system.

For the immunological interpretation of these results, it must be considered that blood represents only a transit compartment for immune cells. As a result of vaccine-induced innate immune responses, haematopoiesis of myeloid cells and DC is likely to be enhanced explaining the increase of many modules relating to these cell types. Furthermore, it is obvious that despite the intramuscular injection, the vaccines cause systemic inflammatory effects, some of which obviously correlate with antibody responses. These are not only a number of modules relating the DC and antigen presentation but also to complement activation and blood coagulation. With these reactions, it is also possible to explain the negative correlation of T/NK cell modules, as these cells are very quickly recruited to lymphoid tissues following acute innate immune response such as virus infections.^25^ The negative correlation of cell cycle BTM could be related to the same mechanisms as T cell represent the main cell fraction in PBMC and many such BTM were actually related to T cells, in particular CD4 T cells.

An important question arising from such studies is whether the above BTM represent either mechanistically relevant modules, non-functional correlates or even modules that also reflect unwanted side effects of strong adjuvants. To address this question, we performed two additional analyses, which were to compare the correlating BTM with the vaccine-induced BTM, as well as to identify BTM correlating with an acute-phase protein. Our results demonstrate that many of the correlating BTM were actually not identified to be significantly induced by the vaccines, indicating that these could more reflect differences in the host response of individual animals to vaccination. Some BTM relating to “DC/antigen presentation” and “cell cycle” appeared to be particular interesting to identify individual variability due to their strong correlation with antibody levels without apparently being influenced by the vaccine. These analyses also permitted to identify potent effects of the TLRL potentially involved in their beneficial action. While BTM related to “cell cycle” had a strong negative correlation to antibodies they were strongly induced by the L vaccine but not by L(TLRL) formulation. Similar observations but as positive correlation/upregulation were made for some “myeloid cells/inflammation” and “DC/antigen presentation” BTM. In contrast, some “T/NK cells” BTM correlated negatively with antibodies and were also downregulated by the L(TLRL) vaccine. It will thus be interesting to address which of the above effect of the TLRL, in particular the prominent effects on the T/NK cell modules, are mechanistically relevant to better understand their mode of action in promoting immune responses.

The main difference with the PBS group was that it did not impact the “T/NK cell” BTM and had an opposite effect (upregulation) on the “cell cycle” BTM. We speculate that in the absence of liposomal formulation the inflammatory responses and the presence of antigen-loaded activated DC in draining lymph nodes could be reduced explaining a reduced recruitment of these cells to lymphoid tissue.

The comparison of the BTM correlation analyses to antibodies and haptoglobin levels indicated that the host responses associate with the acute phase response are largely not linked to those resulting in potent antibody responses. In particular, the BTM families “myeloid cells/inflammation” and “DC/antigen presentation” were found to correlate in a opposite manner at 3 days p.v.

Finally, we also tested whether different pre-vaccination expression levels of BTM can be identified in low or high antibody-producing sheep. Our data indicate that this seems possible even with relatively small group sizes, although additional work is required for confirmation. The relative upregulation of cell cycle, monocyte, DC and innate/inflammatory response BTM in the “high responders” would indicate that the immune system of those animals is in a kind of pre-activated status. A possible explanation for the combination of increased levels of myeloid cell/DC and cell cycle BTM could be increased hematopoiesis and egress of cells from the bone marrow and thymus. Future studies could aim at inducing such a status to enhance vaccine-induced responses.

Obviously, there many other possibilities to analyze gene sets and a choice from a multitude of possible gene sets, including pathways and molecular network-based gene sets,^26,27^ must be made. An example for a different approach selected in cattle is the study by Laughlin et al. using established canonical pathways and gene ontology networks analyzed using a method called Dynamic Bayesian Gene Group Activation.^28^ Although many of the gene sets identified to correlate are related to those found using the BTM method, a direct comparison is difficult. To enable a comparison of our data with a relatively large set of human vaccination data, we selected the BTM method for the present study.

In conclusion, the present work has developed a system immunology readout in the ovine model composed of many immunologically relevant gene expression networks, which correlate with antibody responses. We anticipate that this is applicable to other species and will be very useful to both the identification of efficient vaccine adjuvants causing little unwanted side effects and to find factors related to low vaccine responders.

## Methods

### Vaccine preparation

The TLR4 ligand MPLA was from Avanti Polar Lipids (USA) and the TLR7 ligand guardiquimod from Chemdea (USA). DPPC, DCChol, and PHAD were purchased from Avanti Polar Lipids; Hepes from Applichem; sucrose, chloroform, and ethanol from Sigma-Aldrich (Switzerland). Extruder membranes of 400 and 200 nm pore size, as well as draining disks, were purchased from VWR International (Switzerland). Small-scale liposome formulations for evaluation were prepared as follows. DPPC and DCChol stocks were prepared at 50 mg/ml in methanol/methylen-chloride 1:1 (Sigma-Aldrich). Sixteen milligrams of total lipid mix was prepared and evaporated in an Eppendorf Vacufuge. Lipids were resuspended in 1 ml 20 mM HEPES, 10% sucrose buffer on a shaker for 60 min. Liposomes were serially extruded using an Avanti Mini Extruder (Avanti, USA) with 400, 200, and 100 nm membranes (Avanti). For each filtration step, liposomal solutions were passed 11 times. Large-scale liposomal formulation for the in vivo vaccine trial were made as follows. A solution of DPPC (2.6 mL) at 30 mg/mL in chloroform (78.5 mg) and 1.9 mL of a solution of DCChol at 30 mg/mL in chloroform (57.3 mg) were mixed in a 50 ml round-bottom flask. The solvent was evaporated with rotary evaporator (Laborota 4001, Heidolph) at 40 °C. The lipid film was rehydrated with 8.5 ml of pre-warmed (40 °C) 20 mM Hepes buffer containing 10% sucrose, pH 7.4, to obtain a lipid concentration (DPPC + DCChol) of 16 mg/ml, a DPPC concentration of 9.2 mg/ml, and DCChol concentration of 6.7 mg/ml. The mixture was magnetically stirred for 25 min and incubated for 5 min at room temperature (RT). The mixture was inserted into an extruder (T.001, Northern Lipids, Inc.) equipped with a thermo barrel of 10 ml and equilibrated at 50 °C. After five extrusion cycles with a filter of pore size of 400 nm and five cycles with a filter of 200 nm, the liposome suspension was filtered through a 0.22 µm filter to recover 7.5 ml of the sterile product. The filtered product was flushed with a nitrogen stream and stored at 4 °C.

Cationic liposomes containing gardiquimod were prepared with the same procedure by rehydrating the lipid film with 8.5 ml of pre-warmed (40 °C) 20 mM Hepes buffer containing 10% sucrose and gardiquimod at 1 mg/ml, final pH 7.4. Cationic liposomes containing MPLA were prepared with the same procedure, except that 1.7 ml of a solution of MPLA in chloroform at 1 mg/ml was added to the chloroform solutions of DPPC and DCChol before evaporation and formation of the lipid film. Liposomes were formulated with antigen to final concentration of 8 mg/ml lipid and 20 μg/ml FMDV 146S antigen using a HEPES/Sucrose buffer under stirring. The 146S antigen represents purified inactivated FMDV particles (sedimentation coefficient of 146) prepared from FMDV A Iran96 and was kindly provided by Merial Ltd, Pirbright, UK. The antigen dose was selected as the highest possible dose not strongly altering the physical characteristics of the liposomal vaccine formulations (Supplementary Fig. 1).

### Vaccine characterization

The liposome products were characterized immediately after preparation by measuring pH, average particle size, polydispersion, zeta potential (light scattering, Zetasizer Nano ZS, Malvern), and lipid component concentration (RP-HPLC, Agilent). In addition, liposomes were assessed by transmission electron microscopy after negative staining. A droplet of the liposome suspension diluted 1:5 in buffer was placed on a collodion/carbon-coated 300-mesh copper grid (Electron Microscopy Sciences, Hatfield, PA). After waiting 1 min to permit adsorption, excess liquid was blotted with a filter paper and a drop of 2% phosphotungstic acid at pH 7.0 was added. After another 45 s, the negative stain was removed by touching a filter paper to the edge of the grid. Samples were dried overnight at RT and examined with a Philips CM12 transmission electron microscope (FEI, The Netherlands) at an accelerating voltage of 80 kV with primary magnifications ranging from × 25,000 to × 110,000. Micrographs were captured with a Mega View III camera using the iTEM software version 5.2 (Olympus Soft Imaging Solutions GmbH, Münster, Germany). For the data see Supplementary Fig. 1.

### Immunization of sheep

Three groups of six Skudden sheep were immunized intramuscular with 2 ml vaccine dose containing 146S antigen in PBS (PBS), antigen formulated in liposomes (L), or antigen formulated in liposomes together with MPLA/Gardiquimod as TLR ligands (L(TLRL)). For the group size we performed a power analysis assuming to find an increase of antibodies by 90% with a power of 1 − *β* = 0.8 (80% security), with a standard error of lower than 40%. An error *ɑ* of 0.05 and an error *β* of 0.2 was employed. Each group was composed of three young (5–6 month of age) and three older (1–2 years of age). Each group was composed of five females and one castrated male. A rationale to have a such mixed group of animals was to introduce natural variability which would also be present in field situations. We also reasoned that correlation analyses can only be performed with a certain degree of variation. Serum and PBMC samples were taken previous to vaccination and on day 3, 14, and 28. The animal experiment was performed in accordance to the Article 18 of the Swiss Animal Welfare Act (TSchG) and was approved by the Cantonal Ethical Committee for animal experiments.

### Serum neutralization assay

Clot activator tubes (Becton Dickinson) were used to sample serum, which was centrifuged at 1000 × *g* and stored at − 20 °C until analysis. Serum was heat-inactivated at 56 °C for 30 min. Twofold serial dilutions of serum starting with 1:10 were performed in 50 μl final volume. Fifty microliters of FMDV A Iran96 at 100 TCID_50_ was added to each well and incubated at 37 °C 5% CO_2_ for 1 h. Samples of vaccinated cattle were used for standardization purpose. LFBK cells (kindly obtained from Dr. Luis Rodriguez, Plum Island Animal Disease Center, New York, USA) were added to wells in suspension (100 μl at 2 × 10^5^ cells/ml) and incubated for 72 h. Cytopathic effect was used for readout. The Reed and Muench formula was used for calculation of neutralizing titer.^29^ Statistical analyses for differences in antibody responses used two-way analysis of variance (ANOVA) combined with Tukey’s multiple comparison test.

### Haptoglobin

Serum haptoglobin was measured using a the Tridelta PHASE haptoglobin assay (Tridelta Development Ltd, Ireland). Statistical analyses for differences in haptoglobin responses used two-way ANOVA combined with Tukey’s multiple comparison test.

### Phenotyping of PBMC

Blood was collected in EDTA-coated tubes (Becton Dickinson) and PBMCs were isolated by density centrifugation (1000 × *g*, 25 min) over Ficoll-Paque (GE Healthcare, UK) using citrated blood. On D0 and D3, PBMCs from all animals were frozen in liquid nitrogen and used for phenotyping of monocyte and T cells populations by flow cytometry. For monocyte populations, cells were stained with anti-CD14 (IgM, CAM66A, Kingfisher Biotech, MN, USA), anti-CD16 (IgG2a, KD1, Biorad) and anti-CD172a (IgG1, DH59B, Kingfisher) followed by secondary conjugates including anti-mouse IgM AlexaFluor 647, anti-mouse IgG2a AlexaFluor 488 and anti-mouse IgG2b PECy7 (all Thermofisher). Monocyte subsets were then identified as CD14+ and CD14-CD172highCD16+ cells. For T cell populations, PBMCs were incubated with anti-CD4 (IgG2a, 44.38, Biorad) and anti-CD8 (IgG1, CACT80C, Kingfisher) antibodies followed by anti-mouse IgG2a AlexaFluor 647 and anti-mouse IgG1 PE antibodies.

### Isolation of PBMC RNA

Five million PBMCs were centrifuged at 350 × *g* and the pellet was resuspended in 1 ml TRIzol(R) (Thermo Fisher). RNA was isolated using the NucleoSpin RNA kit (Macherey-Nagel, Germany). Chloroform:IAA (0.2 ml; 49:1) was added per ml of TRIzol and tubes were shaken vigorously for 15 s followed by incubation for 3 min at RT. Then, the tubes were centrifuged at 12,000 × *g*, 4 °C for 15 min and the aqueous phase was transferred to new tubes and mixed with 500 μl isopropanol. After incubation for 10 min at RT, the samples were loaded on to NucleoSpin RNA columns and centrifuged at 11,000 × *g* for 30 s. MDB (350 μl) was added to columns followed by centrifugation at 11,000 × *g* for 30 s. DNase mixture (95 μl) was applied on the membranes for 20 min followed by washing with RAW2 buffer and centrifugation as above. This was repeated twice with 600 μl and 250 μl RA3 buffer, respectively. RNA was eluted with 40 μl RNase-free water and centrifugation at 11,000 × *g* for 1 min. Samples were stored at − 70 °C until analysis.

### RNA-seq data analysis

The RNA quality was checked using a Fragment Analyzer. The average RIN value of the samples is 9.1. The average insert size of the library was 446 bp (min:413 bp, max 473 bp). The library kit used was TruSeq Stranded mRNA from Illumina. The libraries were barcoded and 24 samples multiplexed per sequencing lane. The libraries were single-end sequenced at 100bp. The instrument used was an Illumina HiSeq3000 of the NGS platform of the University of Bern (http://ngs.unibe.ch). The average number of reads per library was 16,056,611 and their median 16,312,387. The quality of the reads was assessed using fastqc v.0.11.2 (http://www.bioinformatics.babraham.ac.uk/projects/fastqc/). Ribosomal RNA (rRNA) was removed by mapping the reads to a collection of rRNA sequences (ensembl release 3.1) with Bowtie2 v. 2.2.1.^30^ The remaining reads were mapped to the Ovis aries reference genome (Oar_v3.1) with Tophat v.2.0.13.^31^ We used htseq-count v.0.6.1^32^ to count the number of reads overlapping with each gene, as specified in the ensembl annotation (Oar_v3.1.86). The Bioconductor package DESeq2 v. 1.10.1^33^ was used to test for differential gene expression between the different time points and vaccines. As cutoff for significance we employed an adjusted *p* value of 0.05 based on the Benjamini–Hochberg procedure. Detailed information about the genes including the Entrez Gene ID, the description of the gene, the human homolog ensembl gene ID and the human homolog gene name was obtained using the Bioconductor package BioMart v. 2.26.1.^34^ The raw data was uploaded to the European Nucleotide Archive accession number PRJEB26387.

### BTM analyses

To further analyze the molecular signatures of the response to different vaccines, we performed a GSEA using blood transcription modules defined by Li et. al.^1^ These modules are composed of 9 up to 347 different genes. Modules were translated to Ensembl annotation and some genes lacking human homolog gene name were manually added to allow higher coverage. To this end we employed Biomart (www.ensembl.org/info/data/biomart) to create a list of putative sheep homologue Ensembl numbers. All genes known to differ in name for human and sheep were replaced by the sheep homologues if sufficient information was found. This included MHC, CD1, TCR, Ig and IFN genes. We also completed the list with many genes not annotated, focussing on those represented in many BTM. Ovine genes with high homology (>80) to another mammal were manually annotated and included in the BTM. Human genes without HGNC symbol in the original BTM list published by Li et al.^1^ were removed as most of them were found to be either withdrawn, represent non-coding RNAs or pseudogenes. The composition of sheep BTM can be downloaded from the Supplementary Material. The GSEA tool (software.broadinsititute.org)^35^ was used to calculate enrichment scores for BTMs with each individual animal. Significant modules were defined using *p* < 0.05 corrected for multiple testing by false discovery rate *q* < 0.05. Pearson’s correlation of enrichment scores with antibody or haptoglobin levels was calculated in R. GSEA was also used to compare to compare the different vaccines. To this end, a grouped GSEA was performed. *P* < 0.05 was considered significant. To analyze difference in high and low antibody responders, animals in each vaccine group having an SNT at day 28 of >50% above the median were arbitrarily defined as “high responders” (*n* = 6) and those with < 50% of the median as “low responders” (*n* = 6). Two animals per vaccine group fitted into each these criteria. Then GSEA was used to compare BTM levels found in high and low responders. Heatmaps, scatter plots, and polar plots were created using the ggplot 2 package or Graphpad Prism 7.0.

## Supplementary information

Supplementary Material

Supplementary file

## Data Availability

The raw sequence data was uploaded to the European Nucleotide Archive accession number PRJEB26387. Other data sets are available in the Supplementary Data. Raw flow cytometry data are available from the corresponding author upon reasonable request.

## Supplementary information

**Supplementary information** accompanies the paper on the *npj Vaccines* website (10.1038/s41541-018-0078-0).
