# Author Correction: System immunology-based identification of blood transcriptional modules correlating to antibody responses in sheep

**DOI:** 10.1038/s41541-019-0100-1

**Published:** 2019-02-19

**Authors:** Roman Othmar Braun, Livia Brunner, Kurt Wyler, Gaël Auray, Obdulio García-Nicolás, Sylvie Python, Beatrice Zumkehr, Véronique Gaschen, Michael Hubert Stoffel, Nicolas Collin, Christophe Barnier-Quer, Rémy Bruggmann, Artur Summerfield

**Affiliations:** 1 Institute of Virology and Immunology, Mittelhäusern, Switzerland; 2 Graduate School for Cellular and Biomedical Sciences, University of Bern, Bern, Switzerland; 3 Vetsuisse Faculty, Department of Infectious Disease and Pathobiology, University of Bern, Länggassstrasse 122, 3001 Bern, Switzerland; 4 Vaccine Formulation Laboratory, Department of Biochemistry, University of Lausanne, Lausanne, Switzerland; 5 Interfaculty Bioinformatics Unit and Swiss Institute of Bioinformatics, University of Bern, Bern, Switzerland; 6 Division of Veterinary Anatomy, University of Bern, Bern, Switzerland

**Correction to:** *npj Vaccines* 10.1038/s41541-018-0078-0, Published online 03 October 2018

In the original published version of this article, substantive revisions that had been requested during peer review, and assessed by the reviewers were omitted from the final version submitted by the authors. The corrections address the scientifically substantive issues that were not included in that version and have been corrected in the HTML and PDF versions of the article. The Supplementary Information for this correction includes the details of the changes made to the article. All authors (R.O.B., L.B., K.W., G.A., O.G.N., S.P., B.Z., V.G., M.H.S., N.C., C.B.Q., R.B. and A.S.) have approved these changes.

## Supplementary Information

Supplementary Information

## Supplementary information

**Supplementary information** accompanies the paper on the *npj Vaccines* website (10.1038/s41541-019-0100-1).

