# Supplementary material for: System immunology-based identification of blood transcriptional modules correlating to antibody responses in sheep
Source: NPJ Vaccines. 2018 Oct 3;3:41. doi: 10.1038/s41541-018-0078-0 (PMC6170373; doi:10.1038/s41541-018-0078-0)
Supplement: Supplementary file 1 — Supplementary Material [file 41541_2018_78_MOESM1_ESM.pdf]

SUPPLEMENTARY FIGURES

System immunology-based identification of blood transcriptional modules correlating to antibody responses in sheep

Roman Othmar Braun[1,6], Livia Brunner[4], Gaël Auray[1], Kurt Wyler[2], Obdulio García-Nicolás[1], Véronique Gaschen[5], Michael Stoffel[5], Nicolas Collin[4], Christophe Barnier-Quer[4], Rémy Bruggmann[2], Artur Summerfield[1,7]

[1] *Institute of Virology and Immunology, Mittelhäusern, Switzerland*
[2] *Interfaculty Bioinformatics Unit and Swiss Institute of Bioinformatics, University of Bern, Switzerland*
[3] *Department of Clinical Research, University of Bern, Switzerland*
[4] *Vaccine Formulation Laboratory, Department of Biochemistry, University of Lausanne, Switzerland*
[5] *Division of Veterinary Anatomy, University of Bern, Switzerland*
[6] *Graduate School for Cellular and Biomedical Sciences, University of Bern, Switzerland*
[7] *Department of Infectious Disease and Pathobiology, Vetsuisse Faculty, University of Bern, Switzerland*

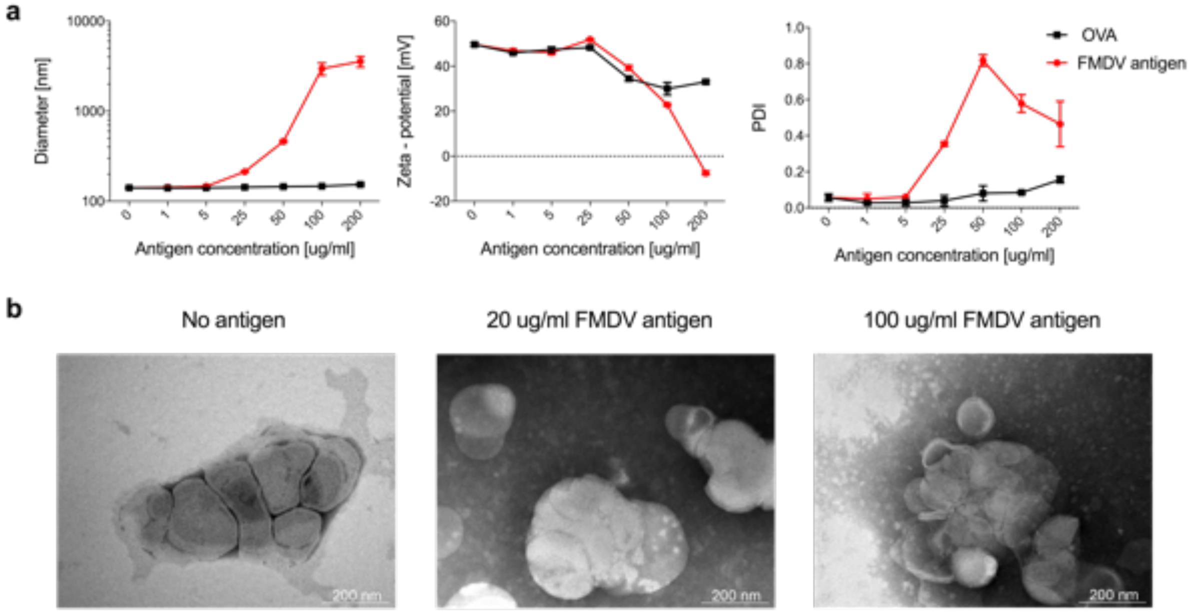

Supplementary Fig. 1. Physicochemical characterisation and transmission electron microscopy of liposomal antigen formulations. a. Size, zeta-potential and poly dispersity index of liposomes formulated with increasing concentrations of FMDV antigen or ovalbumin (OVA). Representative results of three independent experiments are shown. **b.** Negative stain transmission electron microscopy of distinct concentrations of FMDV antigen formulated with liposomes. The FMDV antigen concentration selected for the immunization was 20 μg/ml.

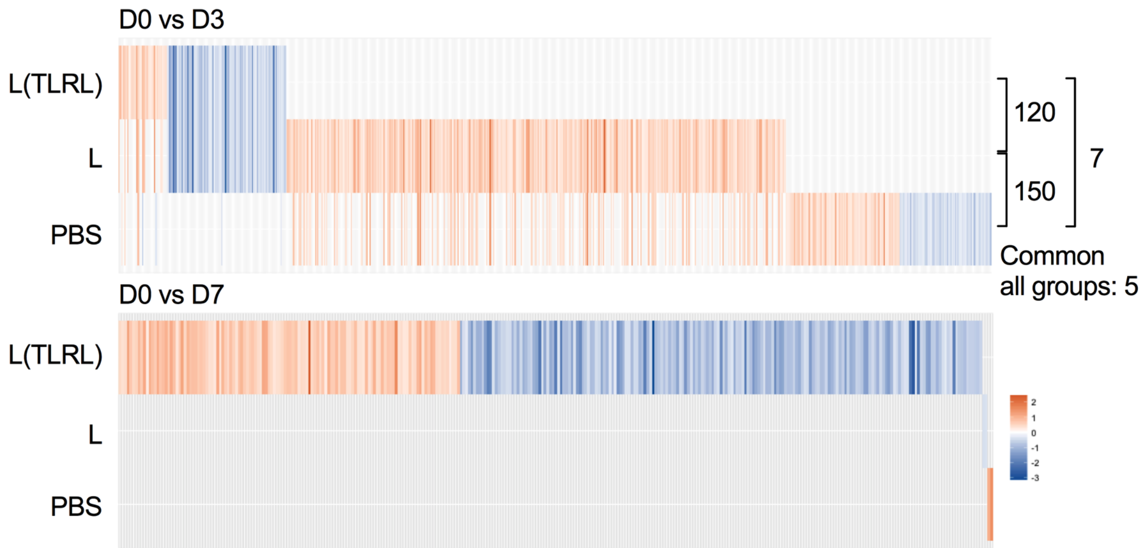

Supplementary Fig. 2. Vaccine-dependent differential gene expression on day 3 and day 7 compared to pre-immunization. A heatmap of significantly up (red) and down (blue) regulated genes is shown to visualize common differentially expressed genes between treatment groups. The total number of common genes between treatments is shown on the right. The list of the genes can be obtained upon request.

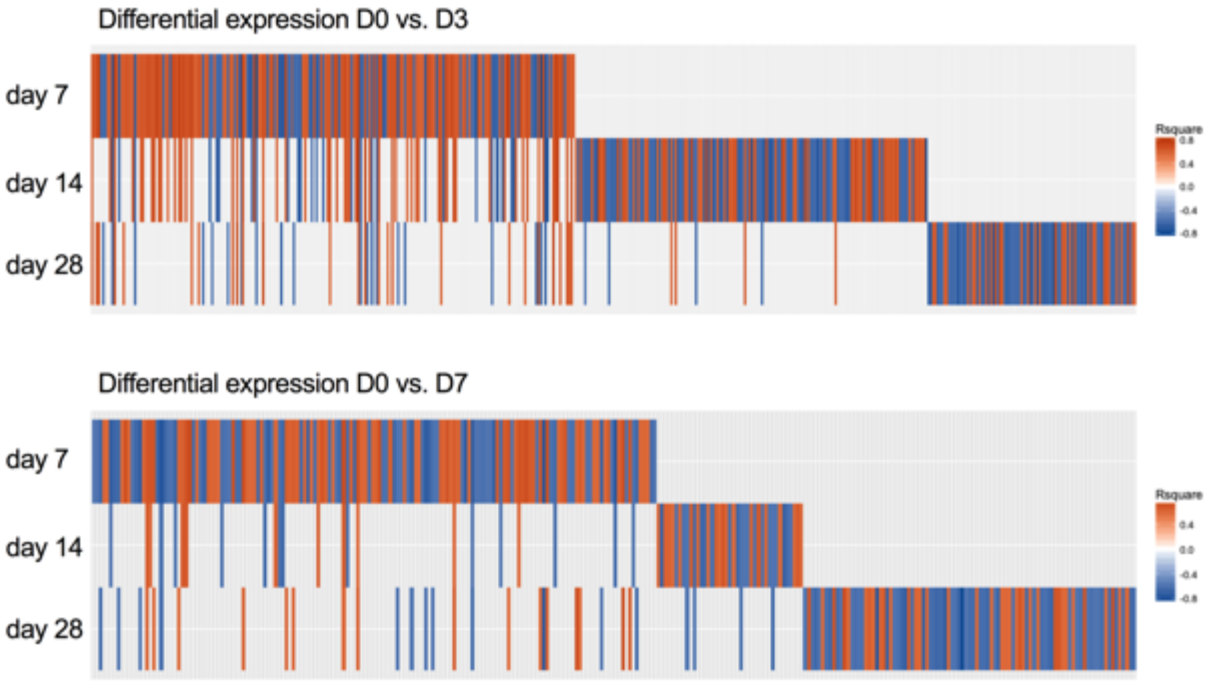

Supplementary Fig. 3. Heatmap of significant genes correlating with neutralizing antibody levels. Colors indicate positive (red) and negative (blue) correlation. Differential expression was analyzed for D0 versus D3 (upper panel) and D0 versus D7 (lower panel). The list of the genes can be obtained upon request.

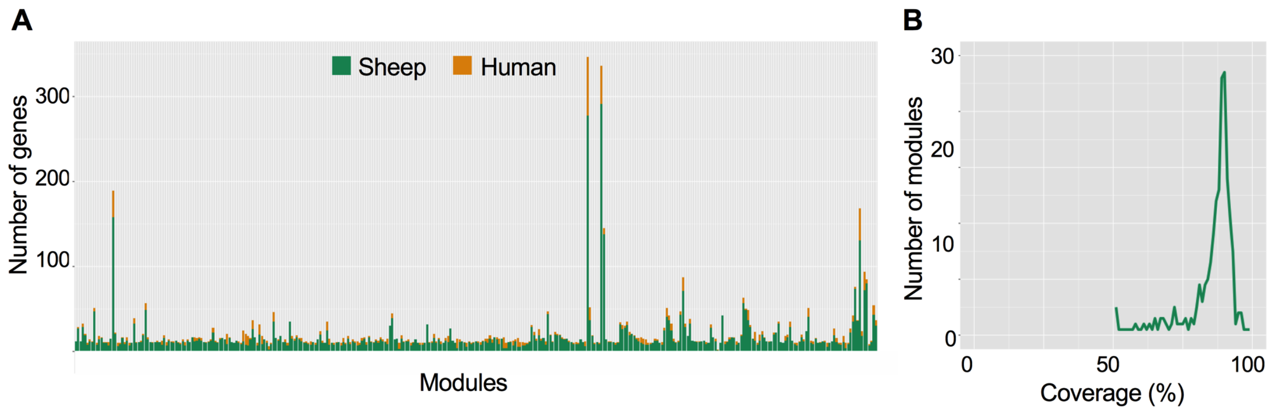

Supplementary Fig. 4. Translation of human blood transcriptional modules to the ovine system. **a.** Number of human and sheep genes assigned to 349 BTM (based on annotation of the sheep genome at the time of analyses). **b.** Coverage of sheep genes assigned to BTM compared to the original composition[1].

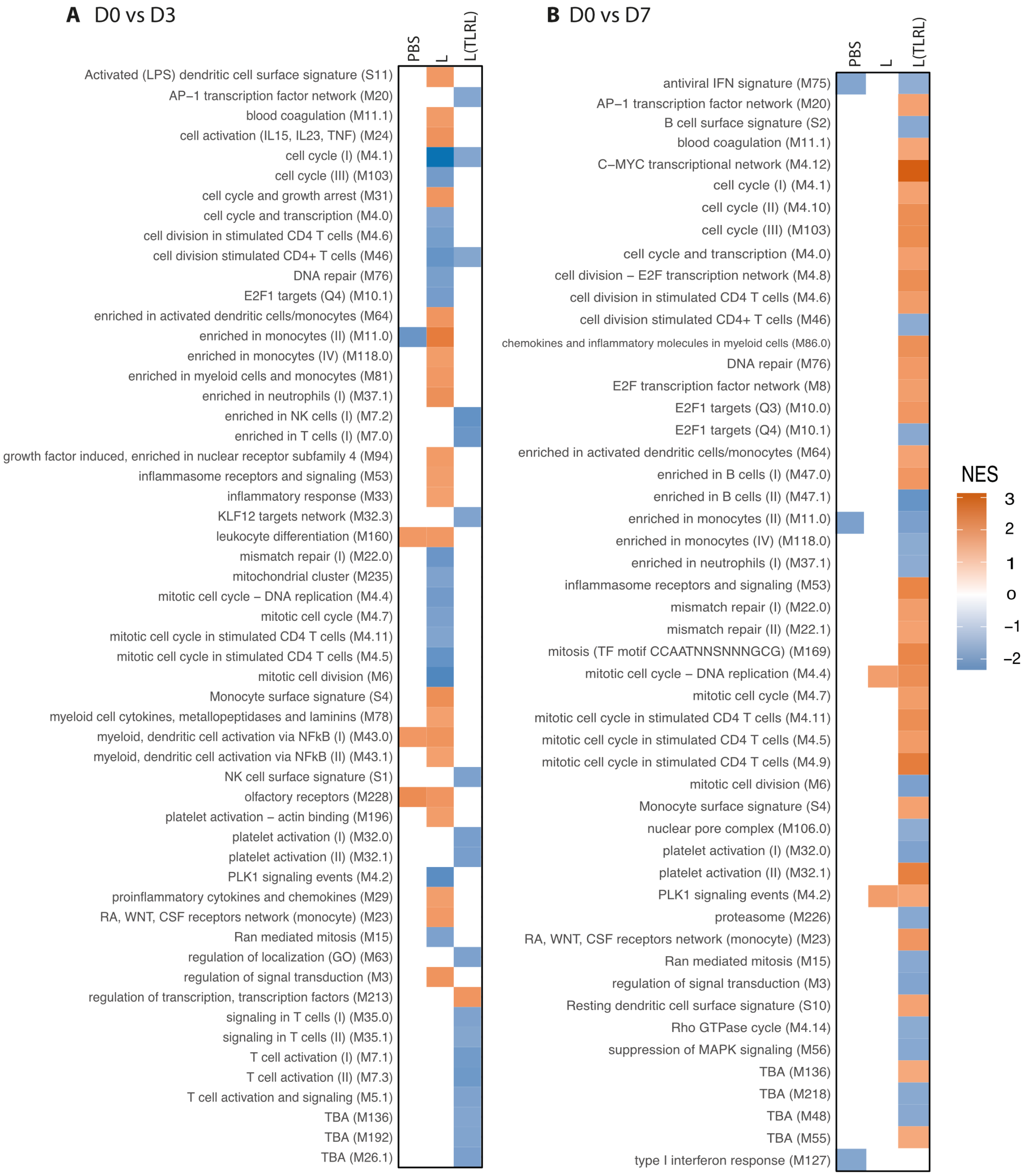

Supplementary Fig. 5. Vaccine-dependent modulation of BTM. Normalized enrichment scores (NES) for the three tested vaccines were calculated using GSEA. **a.** Heatmap showing vaccine-dependent NES for modules significantly modulated (q<0.05) when comparing D3 transcriptome to pre-vaccination values. **b.** Heatmap showing vaccine-dependent NES for modules significantly modulated (q<0.05) when comparing D7 transcriptome to pre-vaccination values. The blank cells indicate that there was no significant modulation for this time point post vaccination.

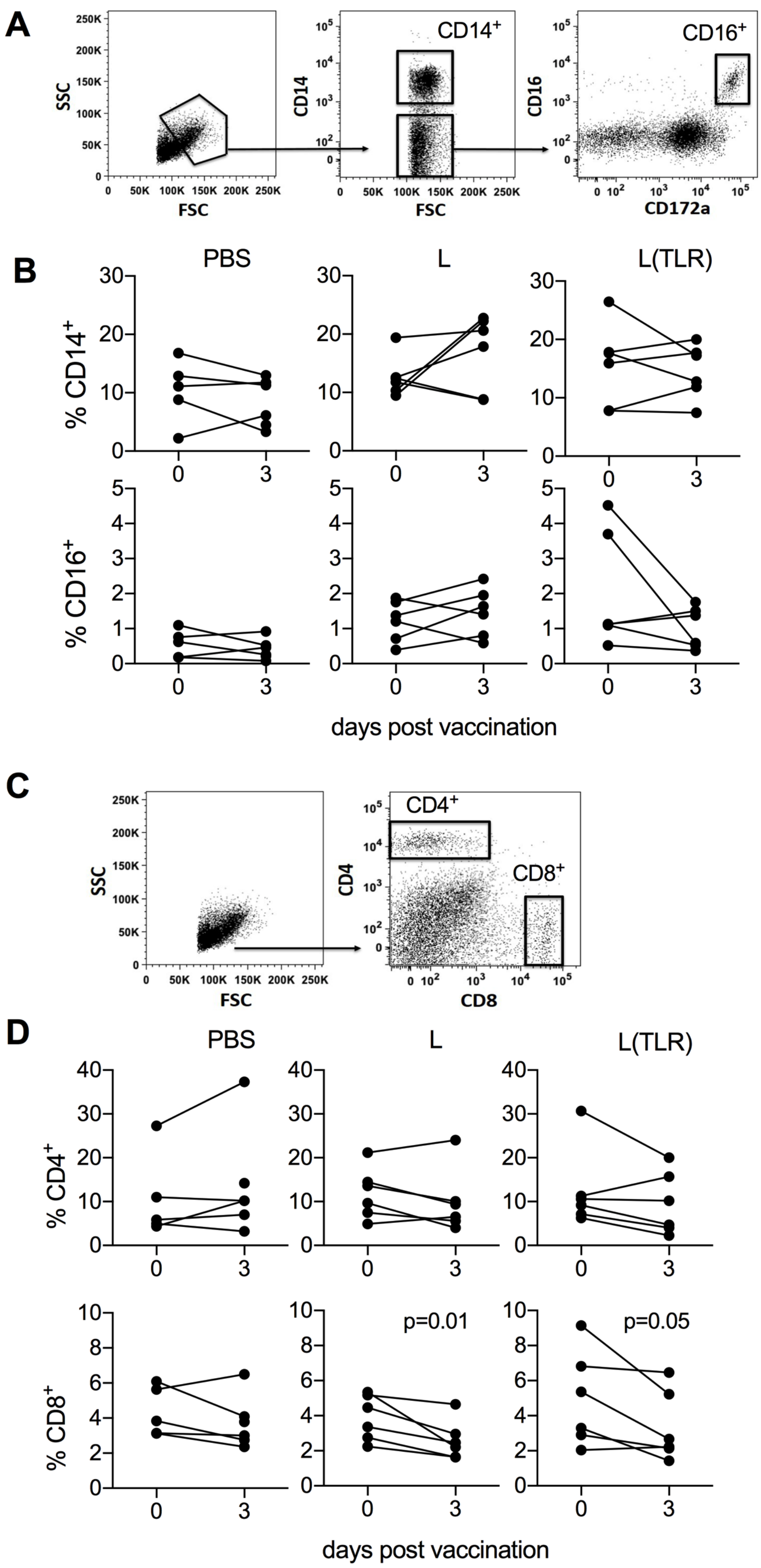

Supplementary Fig. 6. Vaccine-dependent modulation of peripheral monocyte and T cell subsets relative frequencies. PBMC isolated before vaccination (D0) and at D3 were phenotyped to determine the relative frequencies A. PBMC were stained for CD14, CD172a and CD16. Monocytes were defined as CD14$^+$ or CD172a$^+$CD16$^+$. B. Vaccine-dependent changes in frequencies of monocytes subsets. D PBMC were stained for CD4 and CD8. D. Vaccine-dependent changes in frequencies of CD4$^+$ and CD8$^+$ T-cell subsets. A paired T test was employed to determine p values.

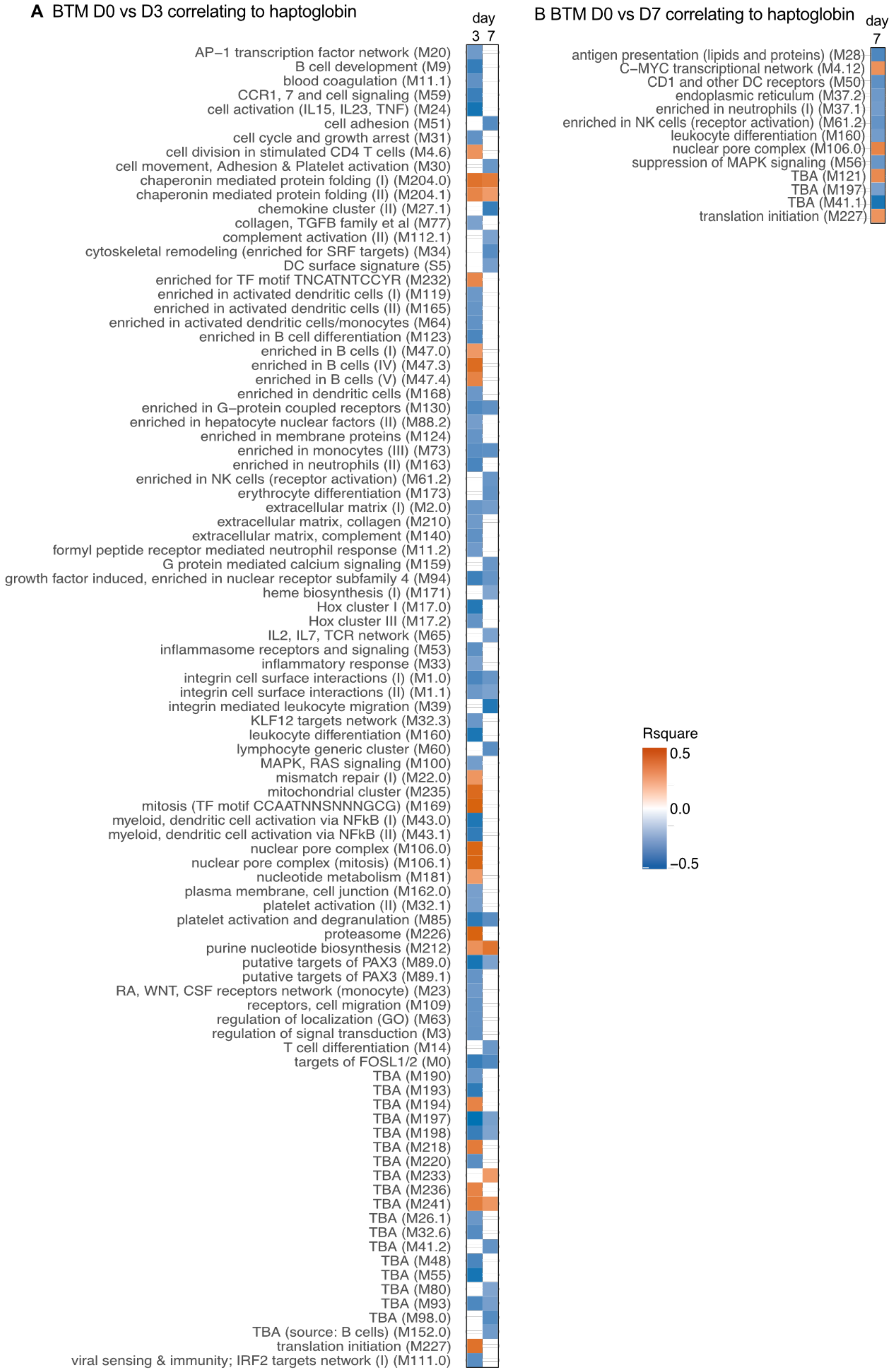
A BTM D0 vs D3 correlating to haptoglobin
day
3 7
AP−1 transcription factor network (M20)
B cell development (M9)
blood coagulation (M11.1)
CCR1, 7 and cell signaling (M59)
cell activation (IL15, IL23, TNF) (M24)
cell adhesion (M51)
cell cycle and growth arrest (M31)
cell division in stimulated CD4 T cells (M4.6)
cell movement, Adhesion & Platelet activation (M30)
chaperonin mediated protein folding (I) (M204.0)
chaperonin mediated protein folding (II) (M204.1)
chemokine cluster (II) (M27.1)
collagen, TGFB family et al (M77)
complement activation (II) (M112.1)
cytoskeletal remodeling (enriched for SRF targets) (M34)
DC surface signature (S5)
enriched for TF motif TNCATNTCCYR (M232)
enriched in activated dendritic cells (I) (M119)
enriched in activated dendritic cells (II) (M165)
enriched in activated dendritic cells/monocytes (M64)
enriched in B cell differentiation (M123)
enriched in B cells (I) (M47.0)
enriched in B cells (IV) (M47.3)
enriched in B cells (V) (M47.4)
enriched in dendritic cells (M168)
enriched in G−protein coupled receptors (M130)
enriched in hepatocyte nuclear factors (II) (M88.2)
enriched in membrane proteins (M124)
enriched in monocytes (III) (M73)
enriched in neutrophils (II) (M163)
enriched in NK cells (receptor activation) (M61.2)
erythrocyte differentiation (M173)
extracellular matrix (I) (M2.0)
extracellular matrix, collagen (M210)
extracellular matrix, complement (M140)
formyl peptide receptor mediated neutrophil response (M11.2)
G protein mediated calcium signaling (M159)
growth factor induced, enriched in nuclear receptor subfamily 4 (M94)
heme biosynthesis (I) (M171)
Hox cluster I (M17.0)
Hox cluster III (M17.2)
IL2, IL7, TCR network (M65)
inflammasome receptors and signaling (M53)
inflammatory response (M33)
integrin cell surface interactions (I) (M1.0)
integrin cell surface interactions (II) (M1.1)
integrin mediated leukocyte migration (M39)
KLF12 targets network (M32.3)
leukocyte differentiation (M160)
lymphocyte generic cluster (M60)
MAPK, RAS signaling (M100)
mismatch repair (I) (M22.0)
mitochondrial cluster (M235)
mitosis (TF motif CCAATNNSNNNGCG) (M169)
myeloid, dendritic cell activation via NFkB (I) (M43.0)
myeloid, dendritic cell activation via NFkB (II) (M43.1)
nuclear pore complex (M106.0)
nuclear pore complex (mitosis) (M106.1)
nucleotide metabolism (M181)
plasma membrane, cell junction (M162.0)
platelet activation (II) (M32.1)
platelet activation and degranulation (M85)
proteasome (M226)
purine nucleotide biosynthesis (M212)
putative targets of PAX3 (M89.0)
putative targets of PAX3 (M89.1)
RA, WNT, CSF receptors network (monocyte) (M23)
receptors, cell migration (M109)
regulation of localization (GO) (M63)
regulation of signal transduction (M3)
T cell differentiation (M14)
targets of FOSL1/2 (M0)
TBA (M190)
TBA (M193)
TBA (M194)
TBA (M197)
TBA (M198)
TBA (M218)
TBA (M220)
TBA (M233)
TBA (M236)
TBA (M241)
TBA (M26.1)
TBA (M32.6)
TBA (M41.2)
TBA (M48)
TBA (M55)
TBA (M80)
TBA (M93)
TBA (M98.0)
TBA (source: B cells) (M152.0)
translation initiation (M227)
viral sensing & immunity; IRF2 targets network (I) (M111.0)
B BTM D0 vs D7 correlating to haptoglobin
day
7
antigen presentation (lipids and proteins) (M28)
C−MYC transcriptional network (M4.12)
CD1 and other DC receptors (M50)
endoplasmic reticulum (M37.2)
enriched in neutrophils (I) (M37.1)
enriched in NK cells (receptor activation) (M61.2)
leukocyte differentiation (M160)
nuclear pore complex (M106.0)
suppression of MAPK signaling (M56)
TBA (M121)
TBA (M197)
TBA (M41.1)
translation initiation (M227)
Rsquare
0.5
0.0
−0.5

Supplementary Fig. 7. BTM correlating to serum haptoglobin levels. Normalized enrichment scores for the BTM were determined from individual animals using GSEA and correlated to the levels of neutralizing antibodies **a.** Heatmap showing the correlation of D3 BTM with haptoglobin levels on day 7, 14 or 28 after immunization. Only animals from the L and L(TLRL) groups were included (n = 12, cutoff $p < 0.05$). **b.** Heat map showing the correlation of D7 BTM with haptoglobin levels in analogy to (a). The blank cells indicate that there was no significant correlation for this time point post vaccination.

Supplementary Table 1. Sample library metrics

Group	Name	RIN	Method	Insert size library	# reads	% Q30 PF
L(TLRL) d0	201	8.9	Fragment Analyzer	444	11'542'191	88.32
L(TLRL) d0	202	9.5	Fragment Analyzer	442	14'057'197	86.23
L(TLRL) d0	203	8.6	Fragment Analyzer	455	16'383'943	83.01
L(TLRL) d0	204	9.3	Fragment Analyzer	454	17'387'396	87.12
L(TLRL) d0	205	9.8	Fragment Analyzer	455	16'182'875	87.08
L(TLRL) d0	206	9.4	Fragment Analyzer	418	15'296'178	82.17
PBS d0	207	9.2	Fragment Analyzer	462	2'904'856	81.54
PBS d0	208	9.8	Fragment Analyzer	458	18'123'354	86.56
PBS d0	209	9.7	Fragment Analyzer	453	18'983'793	86.98
PBS d0	210	9.9	Fragment Analyzer	456	17'160'430	87.03
PBS d0	211	10	Fragment Analyzer	464	15'014'435	85.3
PBS d0	212	9.8	Fragment Analyzer	426	18'664'786	79.81
L d0	213	9.9	Fragment Analyzer	459	14'457'653	88.92
L d0	214	10	Fragment Analyzer	473	19'652'027	87.67
L d0	215	9	Fragment Analyzer	459	19'598'992	86.87
L d0	216	9.8	Fragment Analyzer	435	16'104'039	83.31
L d0	217	9.7	Fragment Analyzer	438	17'283'847	88.47
L d0	218	9.4	Fragment Analyzer	441	16'875'155	88.38
L(TLRL) d3	301	8.1	Fragment Analyzer	455	15'002'778	88.16
L(TLRL) d3	302	8.6	Fragment Analyzer	441	16'709'612	87.67
L(TLRL) d3	303	9.8	Fragment Analyzer	445	17'611'288	87.07
L(TLRL) d3	304	9.5	Fragment Analyzer	434	15'552'772	87.41
L(TLRL) d3	305	10	Fragment Analyzer	440	18'174'100	87.48
L(TLRL) d3	306	9.7	Fragment Analyzer	435	17'264'220	87.77
PBS d3	307	9.6	Fragment Analyzer	433	14'428'164	88.82
PBS d3	308	10	Fragment Analyzer	449	14'529'744	87.02
PBS d3	309	9.7	Fragment Analyzer	448	16'479'451	88.22
PBS d3	310	10	Fragment Analyzer	458	17'266'591	86.64
PBS d3	311	10	Fragment Analyzer	445	22'481'215	87.7
PBS d3	312	9.7	Fragment Analyzer	445	18'610'834	87.99
L d3	313	8.8	Fragment Analyzer	456	15'399'335	88.53
L d3	314	9.8	Fragment Analyzer	443	14'840'266	87.04
L d3	315	9.2	Fragment Analyzer	473	18'471'093	87.77
L d3	316	9.5	Fragment Analyzer	443	21'933'127	88.33
L d3	317	9.3	Fragment Analyzer	433	17'459'822	87.65
L d3	318	9.9	Fragment Analyzer	456	13'110'128	88.64
L(TLRL) d7	401	7.7	Fragment Analyzer	454	14'748'119	86.85
L(TLRL) d7	402	7.3	Fragment Analyzer	413	16'240'830	87.11
L(TLRL) d7	403	6.6	Fragment Analyzer	430	15'793'094	83.38
L(TLRL) d7	404	5.5	Fragment Analyzer	445	17'488'349	86.82
L(TLRL) d7	405	8.4	Fragment Analyzer	459	17'884'330	87.41
L(TLRL) d7	406	7.3	Fragment Analyzer	441	14'540'294	86.69
PBS d7	407	5.9	Fragment Analyzer	451	8'413'441	89.27
PBS d7	408	7.6	Fragment Analyzer	429	15'403'961	88.11
PBS d7	409	8.7	Fragment Analyzer	449	15'414'334	87.09
PBS d7	410	9.4	Fragment Analyzer	447	16'220'707	87.25
PBS d7	411	8.8	Fragment Analyzer	437	16'692'600	88.35
PBS d7	412	8.9	Fragment Analyzer	432	14'585'303	80.33
L d7	413	9.8	Fragment Analyzer	442	18'707'973	89.13
L d7	414	10	Fragment Analyzer	439	16'467'147	87.96
L d7	415	10	Fragment Analyzer	460	18'207'168	88.55
L d7	416	9.5	Fragment Analyzer	443	13'042'340	88.52
L d7	417	10	Fragment Analyzer	466	14'523'043	89.19
L d7	418	9.4	Fragment Analyzer	448	11'686'254	87.53
**average**		**9.1**	NA	**446**	**16'056'611**	**86.9**
**max**		**10.0**	NA	**473**	**22'481'215**	**89.3**
**min**		**5.5**	NA	**413**	**2'904'856**	**79.8**
**median**		**9.5**	NA	**445**	**16'312'387**	**87.4**
