# Supplementary material for: System immunology-based identification of blood transcriptional modules correlating to antibody responses in sheep
Source: NPJ Vaccines. 2018 Oct 3;3:41. doi: 10.1038/s41541-018-0078-0 (PMC6170373; doi:10.1038/s41541-018-0078-0)
Supplement: Supplementary file 2 — Supplementary file [file 41541_2018_78_MOESM2_ESM.pdf]

BTM composition and families

ID	Module.title	Module.member.genes	Family.name.RBr	ENS_ID	NR_FOUND	NR_NOT_FOUND	GENES_NOT_FOUND	TOTAL_GENES
**M0**	targets of FOSL1/2	CCL2,DCN,LIF,PLAU,IL6,MGP,COL1A2,MMP9,THBD,MMP2,CXCL8,MMP1	myeloid cells/inflammation	ENSOARG00000009627,ENSOARG00000015737,ENSOARG00000006322,ENSOARG00000008473,ENSOARG00000012021,ENSOARG00000020724,ENSOARG00000001508,ENSOARG00000007908,ENSOARG00000005117,ENSOARG00000018035,ENSOARG00000014496,ENSOARG00000005315	12	0		12
**M1.0**	integrin cell surface interactions (I)	ITGA9,PDGFRA,PTK2,TNC,PDGFRB,EGFR,ITGA5,LAMB1,COL3A1,LAMB2,CAV2,MYL9,ITGA2B,KDR,ITGB3,COL5A2,COL5A1,COL4A2,COL4A1,LAMC1,FN1,CCND2,ITGAV,MET,COL1A2,COL1A1,COL6A1,COL6A3,COL6A2	others	ENSOARG00000000498,ENSOARG00000019070,ENSOARG00000003418,ENSOARG00000005941,ENSOARG00000006538,ENSOARG00000013753,ENSOARG00000016181,ENSOARG00000007228,ENSOARG00000016476,ENSOARG00000014559,ENSOARG00000001379,ENSOARG00000015847,ENSOARG00000001060,ENSOARG00000016440,ENSOARG00000002129,ENSOARG00000006515,ENSOARG00000006115,ENSOARG00000019180,ENSOARG00000019329,ENSOARG00000010352,ENSOARG00000016607,ENSOARG00000000778,ENSOARG00000001508,ENSOARG00000004871,ENSOARG00000012810,ENSOARG00000019080,ENSOARG00000012880	27	2	ITGA2B,ITGB3	29
**M1.1**	integrin cell surface interactions (II)	LAMC1,TNC,FN1,FBN1,COL4A5,COL1A2,COL4A2,COL4A1,COL1A1,LAMB1,VWF,LAMB2	others	ENSOARG00000019180,ENSOARG00000005941,ENSOARG00000019329,ENSOARG00000021018,ENSOARG00000018187,ENSOARG00000001508,ENSOARG00000006515,ENSOARG00000006115,ENSOARG00000004871,ENSOARG00000007228,ENSOARG00000008752,ENSOARG00000014559	12	0		12
**M2.0**	extracellular matrix (I)	CDH11,PDGFRB,PDGFRA,VCAN,BGN,AEBP1,COL3A1,THBS1,LUM,DCN,THBS2,FSTL1,NID2,NID1,PCOLCE,SPP1,PDGFC,LAMA4,FBN1,COL5A2,COL5A1,COL4A2,COL4A1,MXRA8,COL1A2,COL1A1,COL6A1,COL6A3,COL6A2,MMP2	others	ENSOARG00000001829,ENSOARG00000006538,ENSOARG00000019070,ENSOARG00000015632,ENSOARG00000007656,ENSOARG00000016204,ENSOARG00000016476,ENSOARG00000020058,ENSOARG00000015727,ENSOARG00000015737,ENSOARG00000005037,ENSOARG00000019900,ENSOARG00000020710,ENSOARG00000003598,ENSOARG00000016574,ENSOARG00000002590,ENSOARG00000006586,ENSOARG00000009580,ENSOARG00000021018,ENSOARG00000016440,ENSOARG00000002129,ENSOARG00000006515,ENSOARG00000006115,ENSOARG00000003922,ENSOARG00000001508,ENSOARG00000004871,ENSOARG00000012810,ENSOARG00000019080,ENSOARG00000012880,ENSOARG00000018035	30	0		30
**M2.1**	extracellular matrix (II)	TNFSF13,CD40LG,MPO,VCAN,TFF3,AEBP1,OLFM4,COL3A1,LOX,TNFAIP2,HTRA1,LILRB2,PLA2G7,CHIT1,THBS2,RETN,NID2,NID1,CRISP3,CST3,CTSH,LAMA4,ENTPD1,FBN1,COL5A2,POSTN,COL5A1,COL4A2,CEACAM8,GRN,ACHE,LRG1,CTSG,CXCL1,SMPDL3A,ORM1,ARG1,SPARC,FGL2,COL1A2,ANGPTL2,COL1A1,TGFBI,COL6A2,MMP2	others	ENSOARG00000014243,ENSOARG00000011076,ENSOARG00000009287,ENSOARG00000015632,ENSOARG00000010659,ENSOARG00000016204,ENSOARG00000006710,ENSOARG00000016476,ENSOARG00000000317,ENSOARG00000007785,ENSOARG00000011007,ENSOARG00000005037,ENSOARG00000002291,ENSOARG00000020710,ENSOARG00000003598,ENSOARG00000012520,ENSOARG00000006202,ENSOARG00000014664,ENSOARG00000009580,ENSOARG00000006716,ENSOARG00000021018,ENSOARG00000016440,ENSOARG00000010041,ENSOARG00000002129,ENSOARG00000006515,ENSOARG00000008164,ENSOARG00000015526,ENSOARG00000009290,ENSOARG00000014775,ENSOARG00000008143,ENSOARG00000013943,ENSOARG00000009032,ENSOARG00000018670,ENSOARG00000001508,ENSOARG00000012521,ENSOARG00000004871,ENSOARG00000015081,ENSOARG00000012880,ENSOARG00000018035	39	6	TNFAIP2,LILRB2,CHIT1,CEACAM8,CTSG,ORM1	45
**M2.2**	extracellular matrix (III)	CDH11,DCN,LAMA4,VCAN,FBN1,COL5A2,NID2,COL5A1,COL1A2,ANGPTL2,COL3A1,EDNRA,AEBP1,MMP1	others	ENSOARG00000001829,ENSOARG00000015737,ENSOARG00000009580,ENSOARG00000015632,ENSOARG00000021018,ENSOARG00000016440,ENSOARG00000020710,ENSOARG00000002129,ENSOARG00000001508,ENSOARG00000012521,ENSOARG00000016476,ENSOARG00000008169,ENSOARG00000016204,ENSOARG00000005315	14	0		14

**M3**	regulation of signal transduction	PTK2,TIMP2,WLS,KIF26A,ECM1,GAB1,CCR1,FOXO1,C5AR1,APCDD1,S100A12,SOX7,PLXNB2,FZD8,TRAT1,CSF1R,SULF2,CDK14,PSD3,JUP,ADAP2,NOD2,TIAM2,LYN,FGD4,ENG,FPR1,KLF4,PRKCD,UBASH3A,SHANK3,GATA3,GH1,HPSE,IL1RN,LRP1,BMP2,SYK,HMOX1,TLR2,RGL1,CTGF,ZNF423,TLR5,BCL6,CD3E,ASAP2	others	ENSOARG00000003418,ENSOARG00000003264,ENSOARG00000011383,ENSOARG00000007197,ENSOARG00000020820,ENSOARG00000010978,ENSOARG00000014260,ENSOARG00000006012,ENSOARG00000011005,ENSOARG00000001340,ENSOARG00000000432,ENSOARG00000019806,ENSOARG00000019045,ENSOARG00000006358,ENSOARG00000010080,ENSOARG00000016384,ENSOARG00000004073,ENSOARG00000017474,ENSOARG00000011933,ENSOARG00000017441,ENSOARG00000004194,ENSOARG00000014778,ENSOARG00000019356,ENSOARG00000011387,ENSOARG00000007169,ENSOARG00000000156,ENSOARG00000010806,ENSOARG00000020018,ENSOARG00000013941,ENSOARG00000013932,ENSOARG00000002360,ENSOARG00000020828,ENSOARG00000007332,ENSOARG00000018738,ENSOARG00000007814,ENSOARG00000018475,ENSOARG00000015044,ENSOARG00000002070,ENSOARG00000014240,ENSOARG00000017044,ENSOARG00000005017,ENSOARG00000020487,ENSOARG00000008984,ENSOARG00000014894	44	3	SOX7,FZD8,FPR1	47
**M4.0**	cell cycle and transcription	FXYD6,FAM198B,HSPA6,BUB1B,PLK1,NUF2,CD33,TIMP2,PLK4,CD36,C5AR1,FCGR1B,FCGR1A,S100A11,SEMA4A,MAFB,S100A12,KIF2C,CPPED1,SKA1,TPX2,CCDC99,NUSAP1,TYROBP,UBE2T,FGR,MARCKS,ESPL1,C15orf42,DLGAP5,IRAK3,SIRPA,CDCA7,BRCA1,CENPN,CENPM,CENPK,OIP5,CENPI,CENPH,CENPF,ALOX5,NOD2,MYBL2,ITGAX,CENPA,CLEC4E,SPAG5,ZWINT,CSF3R,CENPW,SLC15A3,DEPDC1B,KIFC1,HPSE,KCTD12,TNFAIP2,LST1,CD300LF,ORC1L,WHSC1,RBM47,H2AFX,SECTM1,KIF23,RAB31,TLR2,CD163,CCNF,DAPK1,TLR4,GLT1D1,CHAC2,PTAFR,TLR8,TOP2A,TCF19,SPC24,SPC25,CDC20,TNFSF13,C16orf75,CD1D,CSF2RA,STMN1,IGSF6,DTL,PTTG3P,EMR2,RASSF4,TROAP,SLC31A2,FEN1,FAM64A,TMEM106C,FGL2,RIN2,POLA2,LY86,PLXNB2,STIL,LILRB3,LILRB2,LILRB1,CLEC7A,CCNE2,EXO1,TIMELESS,RNF130,PAICS,TNS3,CEACAM8,NEK2,DONSON,HNMT,WDFY3,CHEK1,GCA,FCN1,SUV39H2,BUB1,TFEC,CDC25C,CDC25A,NCAPH,SGOL2,RTN1,NCAPG,NCF1C,PGLYRP1,GRN,HCK,KIF18B,KIF15,PBK,LO[illegible]	cell cycle	ENSOARG00000007061,ENSOARG00000006033,ENSOARG00000007452,ENSOARG00000020126,ENSOARG00000017343,ENSOARG00000011189,ENSOARG00000003264,ENSOARG00000015691,ENSOARG00000017123,ENSOARG00000011005,ENSOARG00000020667,ENSOARG00000021079,ENSOARG00000005579,ENSOARG00000001355,ENSOARG00000000432,ENSOARG00000001102,ENSOARG00000004359,ENSOARG00000001419,ENSOARG00000020348,ENSOARG00000005167,ENSOARG00000018545,ENSOARG00000003365,ENSOARG00000009527,ENSOARG00000016587,ENSOARG00000021089,ENSOARG00000002799,ENSOARG00000007425,ENSOARG00000000578,ENSOARG00000004835,ENSOARG00000008206,ENSOARG00000018792,ENSOARG00000006321,ENSOARG00000020342,ENSOARG00000001366,ENSOARG00000005750,ENSOARG00000008368,ENSOARG00000002819,ENSOARG00000017441,ENSOARG00000003547,ENSOARG00000009130,ENSOARG00000002718,ENSOARG00000001001,ENSOARG00000013084,ENSOARG00000019729,ENSOARG00000007744,ENSOARG00000015974,ENSOARG00000007132,ENSOARG00000009637,ENSOARG00000002360,ENSOARG00000015960,ENSOARG00000008167,ENSOARG00000015690,ENSOARG00000012711,ENSOARG00000017039,ENSOARG00000018647,ENSOARG00000001243,ENSOARG00000015044,ENSOARG00000002862,ENSOARG00000018424,ENSOARG00000008706,ENSOARG00000005792,ENSOARG00000016604,ENSOARG00000004190,ENSOARG00000019840,ENSOARG00000018099,ENSOARG00000014321,ENSOARG[illegible]	293	43	CD33,FCGR1B,CPPED1,CCDC99,C15orf42,CENPA,TNFAIP2,CD300LF,ORC1L,SECTM1,C16orf75,PTTG3P,EMR2,LILRB3,LILRB2,LILRB1,PAICS,CEACAM8,FCN1,LOC100288693,NPL,FPR1,C21orf45,KIAA0101,FAM54A,PTTG1,C11orf82,KIAA1598,TACC3,APOBEC3A,APOBEC3B,FAM72A,PILRA,DHFR,MNDA,MS4A6A,FCGR2A,PPIL5,C12orf48,TYMP,MLF1IP,SEPX1,LILRA2	336

**M4.1**	cell cycle (I)	BUB1B,PLK1,PLK4,KIF2C,SKA1,TPX2,CCDC99,ESPL1,DLGAP5,BRCA1,CENPN,CENPM,CENPK,OIP5,CENPI,CENPH,CENPF,CENPE,SPAG5,MYBL2,CENPA,CENPW,KIFC1,H2AFX,KIF23,CCNF,NCAPH,NCAPG,TOP2A,SPC24,SPC25,KIF11,NCAPG2,STMN1,DTL,ZWINT,FEN1,NEK2,CCNE2,EXO1,TIMELESS,TTK,TYMS,CHEK1,SUV39H2,BUB1,CDC25C,CDC25A,SGOL2,NCAPD3,ZWILCH,PBK,GINS2,GINS1,GINS4,GPSM2,UBE2C,CDC20,MELK,DTYMK,POLE2,UBE2S,DSCC1,SMC2,POLA2,RFC5,CDT1,RFC3,SMC4,KIF15,PRKAR2B,FOXM1,FBXO5,FANCD2,GMNN,BRIP1,NDC80,CDC6,CDC7,NUSAP1,RACGAP1,ASPM,ERCC6L,KNTC1,CKS1B,PRIM1,RANBP1,FANCI,HELLS,CKS2,PTTG1,UHRF1,C11orf82,TACC3,CDK1,RAD51,CDC45,KIF20B,KIF20A,MKI67,POLA1,MAD2L1,DHFR,BIRC5,CEP55,RRM2,CHAF1B,PRC1,APITD1,FAM83D,DNA2,CCNA2,ECT2,TIPIN,CDKN3,NUF2,CDCA2,CDCA3,SKA3,AURKA,AURKB,CDCA5,CDCA8,PCNA,MCM10,MND1,ANLN,KIF18A,KIF18B,MCM6,MCM5,MCM4,MCM2,CASC5,HJURP,TRIP13,E2F7,CCNB2,CCNB1,E2F2,R[illegible]	cell cycle	ENSOARG00000020126,ENSOARG00000017343,ENSOARG00000015691,ENSOARG00000001102,ENSOARG00000004359,ENSOARG00000001419,ENSOARG00000016587,ENSOARG00000021089,ENSOARG00000004835,ENSOARG00000008206,ENSOARG00000018792,ENSOARG00000006321,ENSOARG00000020342,ENSOARG00000001366,ENSOARG00000005750,ENSOARG00000008368,ENSOARG00000011059,ENSOARG00000001001,ENSOARG00000003547,ENSOARG00000007744,ENSOARG00000009637,ENSOARG00000017039,ENSOARG00000018647,ENSOARG00000018424,ENSOARG00000013908,ENSOARG00000004016,ENSOARG00000014321,ENSOARG00000017725,ENSOARG00000004104,ENSOARG00000000647,ENSOARG00000007995,ENSOARG00000012293,ENSOARG00000010843,ENSOARG00000013084,ENSOARG00000015633,ENSOARG00000011466,ENSOARG00000006180,ENSOARG00000007232,ENSOARG00000008907,ENSOARG00000007151,ENSOARG00000009367,ENSOARG00000011655,ENSOARG00000007434,ENSOARG00000014305,ENSOARG00000016166,ENSOARG00000002373,ENSOARG00000016031,ENSOARG00000013282,ENSOARG00000018225,ENSOARG00000014858,ENSOARG00000011432,ENSOARG00000007717,ENSOARG00000002379,ENSOARG00000018981,ENSOARG00000006520,ENSOARG00000020542,ENSOARG00000011541,ENSOARG00000017620,ENSOARG00000020607,ENSOARG00000001369,ENSOARG00000016330,ENSOARG00000007399,ENSOARG00000014045,ENSOARG00000003356,ENSOARG00000013512,ENSOARG00000010697,ENSOARG[illegible]	138	7	CCDC99,CENPA,PTTG1,C11orf82,TACC3,DHFR,MLF1IP	145
**M4.2**	PLK1 signaling events	BUB1B,PLK1,CEP55,KIF14,FBXO5,PRC1,MYBL2,NDC80,TPX2,HJURP,ECT2,ASPM,ERCC6L,RAD51AP1,ESPL1,AURKA,WEE1,CLSPN,ASF1B,BUB1,CDC25C,CENPE,SGOL1,NUSAP1,TRIP13,GINS2,KIF23,CDC20,TUBG1,NCAPG,MLF1IP,CKAP2L,SPC25,KIF20A	cell cycle	ENSOARG00000020126,ENSOARG00000017343,ENSOARG00000003158,ENSOARG00000016116,ENSOARG00000003968,ENSOARG00000012267,ENSOARG00000003547,ENSOARG00000009604,ENSOARG00000001419,ENSOARG00000019189,ENSOARG00000020736,ENSOARG00000014713,ENSOARG00000005908,ENSOARG00000009844,ENSOARG00000016587,ENSOARG00000018369,ENSOARG00000012355,ENSOARG00000019542,ENSOARG00000006370,ENSOARG00000014305,ENSOARG00000016166,ENSOARG00000011059,ENSOARG00000020348,ENSOARG00000016070,ENSOARG00000011432,ENSOARG00000018647,ENSOARG00000020542,ENSOARG00000002687,ENSOARG00000004016,ENSOARG00000020887,ENSOARG00000004104,ENSOARG00000015873,ENSOARG00000020126,ENSOARG00000017343,ENSOARG00000003158,ENSOARG00000016116,ENSOARG00000003968,ENSOARG00000012267,ENSOARG00000003547,ENSOARG00000009604,ENSOARG00000001419,ENSOARG00000019189,ENSOARG00000020736,ENSOARG00000014713,ENSOARG00000005908,ENSOARG00000009844,ENSOARG00000016587,ENSOARG00000018369,ENSOARG00000012355,ENSOARG00000019542,ENSOARG00000006370,ENSOARG00000014305,ENSOARG00000016166,ENSOARG00000011059,ENSOARG00000020348,ENSOARG00000016070,ENSOARG00000011432,ENSOARG00000018647,ENSOARG00000020542,ENSOARG00000002687,ENSOARG00000004016,ENSOARG00000020887,ENSOARG00000004104,ENSOARG00000015873	32	2	SGOL1,MLF1IP	34

**M4.3**	myeloid cell enriched receptors and transporters	CD1D,SLC7A7,AMICA1,CCR1,LRRC25,GPBAR1,MYOF,MARCO,SULF2,CD86,SLC1A3,SLC8A1,IGSF6,MPEG1,MTMR11,RNASE6,SLC24A4,SIGLEC16,ASGR1,SLC15A3,KCTD12,CHST15,SLC2A6,SECTM1,CLEC4A,CD14,TLR5,TLR8,SIGLEC7,MS4A14,LILRA1	myeloid cells/inflammation	ENSOARG00000007252,ENSOARG00000019424,ENSOARG00000014260,ENSOARG00000012307,ENSOARG00000002223,ENSOARG00000012328,ENSOARG00000010080,ENSOARG00000020112,ENSOARG00000010729,ENSOARG00000015971,ENSOARG00000011743,ENSOARG00000020733,ENSOARG00000012752,ENSOARG00000012969,ENSOARG00000010137,ENSOARG00000015974,ENSOARG00000015960,ENSOARG00000009887,ENSOARG00000003228,ENSOARG00000002516,ENSOARG00000025180,ENSOARG00000005017,ENSOARG00000018099,ENSOARG00000014547,ENSOARG00000012878,ENSOARG00000002513, ENSOARG00000007252,ENSOARG00000019424,ENSOARG00000014260,ENSOARG00000012307,ENSOARG00000002223,ENSOARG00000012328,ENSOARG00000010080,ENSOARG00000020112,ENSOARG00000010729,ENSOARG00000015971,ENSOARG00000011743,ENSOARG00000020733,ENSOARG00000012752,ENSOARG00000012969,ENSOARG00000010137,ENSOARG00000015974,ENSOARG00000015960,ENSOARG00000009887,ENSOARG00000003228,ENSOARG00000002516,ENSOARG00000025180,ENSOARG00000005017,ENSOARG00000018099,ENSOARG00000014547,ENSOARG00000012878,ENSOARG00000002513	26	5	AMICA1,GPBAR1,SLC8A1,SIGLEC16,SECTM1	31
**M4.4**	mitotic cell cycle - DNA replication	POLA2,POLA1,PLK4,KIF15,CDC6,CDC7,STIL,EXO1,CDT1,ORC6L,PRIM1,PRIM2,PCNA,C1orf112,MCM10,OIP5,MND1,TPX2,MCM7,MCM6,MCM5,MCM4,MCM3,MCM2,MCM8,C12orf48,RPA2,CDK2,POLE2,CDC45	cell cycle	ENSOARG00000014045,ENSOARG00000017574,ENSOARG00000015691,ENSOARG00000004780,ENSOARG00000014063,ENSOARG00000015943,ENSOARG00000003564,ENSOARG00000007232,ENSOARG00000013512,ENSOARG00000008185,ENSOARG00000005654,ENSOARG00000017133,ENSOARG00000011174,ENSOARG00000005416,ENSOARG00000020342,ENSOARG00000003004,ENSOARG00000001419,ENSOARG00000017802,ENSOARG00000010614,ENSOARG00000018527,ENSOARG00000012797,ENSOARG00000014143,ENSOARG00000002774,ENSOARG00000018145,ENSOARG00000003240,ENSOARG00000010667,ENSOARG00000020607,ENSOARG00000017745	28	2	ORC6L,C12orf48	30
**M4.5**	mitotic cell cycle in stimulated CD4 T cells	MAD2L1,CEP55,HNRPLL,LAG3,GPR56,KIAA0101,NDC80,TYMS,FBXO5,MELK,GRAP2,CDCA7,CDCA5,RRM2,CXCR6,MIR155HG,CENPM,CENPK,NUF2,IFNG,ZNF367,MCM6,ENO2,KIF11,DEPDC1B,ZEB1,UHRF1,TACC3,CDC20,CDK1,RNF144A,CHAC2,TOP2A,UBE2T,KIF20A	T/NK cells	ENSOARG00000015665,ENSOARG00000003158,ENSOARG00000008915,ENSOARG00000006528,ENSOARG00000009604,ENSOARG00000009367,ENSOARG00000003968,ENSOARG00000011541,ENSOARG00000017108,ENSOARG00000000578,ENSOARG00000012000,ENSOARG00000015333,ENSOARG00000014277,ENSOARG00000018792,ENSOARG00000006321,ENSOARG00000011189,ENSOARG00000001958,ENSOARG00000008474,ENSOARG00000010614,ENSOARG00000005238,ENSOARG00000000647,ENSOARG00000007132,ENSOARG00000013971,ENSOARG00000008530,ENSOARG00000020542,ENSOARG00000004318,ENSOARG00000014661,ENSOARG00000004190,ENSOARG00000014321,ENSOARG00000018545,ENSOARG00000015873	31	4	GPR56,KIAA0101,MIR155HG,TACC3	35
**M4.6**	cell division in stimulated CD4 T cells	BRCA1,GINS2,CENPM,BRCA2,CDKN3,ANLN,CEP55,HMMR,POLE2,CDCA2,GINS1,NCAPG2,CDCA7,FANCD2,UBE2T,DEPDC1B,FANCL,FAM72A,FAM72B,FAM72C,FAM72D,FANCI,DSCC1	T/NK cells	ENSOARG00000004835,ENSOARG00000011432,ENSOARG00000018792,ENSOARG00000011179,ENSOARG00000021070,ENSOARG00000004361,ENSOARG00000003158,ENSOARG00000014126,ENSOARG00000020607,ENSOARG00000009851,ENSOARG00000007717,ENSOARG00000007995,ENSOARG00000000578,ENSOARG00000005211,ENSOARG00000018545,ENSOARG00000007132,ENSOARG00000001759,ENSOARG00000011362,ENSOARG00000016330	19	4	FAM72A,FAM72B,FAM72C,FAM72D	23

**M4.7**	mitotic cell cycle	CCNB2,CCNB1,CCNA2,SHCBP1,PLK1,CDC25C,CDC25A,EXO1,CDT1,CEP152,PKMYT1,E2F8,TUBG1,CDK1,CEP76,FOXM1,SGOL2,CEP72,NEK2,PLK4,CKAP5	cell cycle	ENSOARG00000020836,ENSOARG00000012913,ENSOARG00000014176,ENSOARG00000015392,ENSOARG00000017343,ENSOARG00000016166,ENSOARG00000002373,ENSOARG00000007232,ENSOARG00000013512,ENSOARG00000021014,ENSOARG00000000736,ENSOARG00000008807,ENSOARG00000002687,ENSOARG00000004318,ENSOARG00000001861,ENSOARG00000011054,ENSOARG00000016031,ENSOARG00000015903,ENSOARG00000011466,ENSOARG00000015691,ENSOARG00000004217,ENSOARG00000020836,ENSOARG00000012913,ENSOARG00000014176,ENSOARG00000015392,ENSOARG00000017343,ENSOARG00000016166,ENSOARG00000002373,ENSOARG00000007232,ENSOARG00000013512,ENSOARG00000021014,ENSOARG00000000736,ENSOARG00000008807,ENSOARG00000002687,ENSOARG00000004318,ENSOARG00000001861,ENSOARG00000011054,ENSOARG00000016031,ENSOARG00000015903,ENSOARG00000011466,ENSOARG00000015691,ENSOARG00000004217	21	0		21
**M4.8**	cell division - E2F transcription network	E2F7,PCNA,NASP,MAD2L1,CASP2,TOPBP1,RFC5,TIMELESS,DNMT1,H2AFX,KIF4A,PRKDC,MCM4,DTYMK,MCM2,ZWINT,RNASEH2A,MCM3,RANBP1	cell cycle	ENSOARG00000014941,ENSOARG00000017133,ENSOARG00000015665,ENSOARG00000017720,ENSOARG00000008582,ENSOARG00000003356,ENSOARG00000008907,ENSOARG00000015380,ENSOARG00000017039,ENSOARG00000015491,ENSOARG00000011884,ENSOARG00000012797,ENSOARG00000017620,ENSOARG00000002774,ENSOARG00000013084,ENSOARG00000010721,ENSOARG00000014143,ENSOARG00000018318	18	1	NASP	19
**M4.9**	mitotic cell cycle in stimulated CD4 T cells	RBBP8,CCDC99,CENPF,CENPE,MASTL,PLK4,ZNF367,KIF14,EZH2,RAD51,NCAPH,GMNN,TRIP13,RRM1,CDC6,DONSON	T/NK cells	ENSOARG00000008368,ENSOARG00000011059,ENSOARG00000015826,ENSOARG00000015691,ENSOARG00000008474,ENSOARG00000016116,ENSOARG00000018856,ENSOARG00000020254,ENSOARG00000013908,ENSOARG00000005900,ENSOARG00000016070,ENSOARG00000001301,ENSOARG00000014063,ENSOARG00000013016,ENSOARG00000008368,ENSOARG00000011059,ENSOARG00000015826,ENSOARG00000015691,ENSOARG00000008474,ENSOARG00000016116,ENSOARG00000018856,ENSOARG00000020254,ENSOARG00000013908,ENSOARG00000005900,ENSOARG00000016070,ENSOARG00000001301,ENSOARG00000014063,ENSOARG00000013016	14	2	RBBP8,CCDC99	16
**M4.10**	cell cycle (II)	BRCA1,CCNB2,CCNB1,DTL,CCNE2,CDC25A,CCNE1,GINS2,CDK1,PERP,MELK,RRM2,STEAP3,CHEK1	cell cycle	ENSOARG00000004835,ENSOARG00000020836,ENSOARG00000012913,ENSOARG00000010843,ENSOARG00000006180,ENSOARG00000002373,ENSOARG00000004067,ENSOARG00000011432,ENSOARG00000004318,ENSOARG00000000612,ENSOARG00000011541,ENSOARG00000015333,ENSOARG00000012368,ENSOARG00000011655	14	0		14
**M4.11**	mitotic cell cycle in stimulated CD4 T cells	CCNA2,CCDC99,MELK,RAD54L,GSG2,SUV39H1,CYB5B,CDCA3,AURKB,TK1,STMN1,RAD51C	T/NK cells	ENSOARG00000014176,ENSOARG00000011541,ENSOARG00000002264,ENSOARG00000017746,ENSOARG00000012096,ENSOARG00000003575,ENSOARG00000005652,ENSOARG00000019052,ENSOARG00000005673,ENSOARG00000012293,ENSOARG00000011574	11	1	CCDC99	12
**M4.12**	C-MYC transcriptional network	CCNB1,NME1,CKS2,BIRC5,CENPA,HMGA1,MCM6,KIF11,BCAT1,TK1,TOP2A,CDCA7	cell cycle	ENSOARG00000012913,ENSOARG00000003026,ENSOARG00000007721,ENSOARG00000005456,ENSOARG00000010561,ENSOARG00000010614,ENSOARG00000000647,ENSOARG00000020103,ENSOARG00000005673,ENSOARG00000014321,ENSOARG00000000578	11	1	CENPA	12
**M4.13**	cell junction (GO)	LCP1,KCTD12,EPB41L3,PRKCD,IFI30,PAK1,FES,TNS3,HCK,HOMER3,CTNNA1	others	ENSOARG00000007864,ENSOARG00000015960,ENSOARG00000010473,ENSOARG00000000156,ENSOARG00000013291,ENSOARG00000007238,ENSOARG00000012648,ENSOARG00000012535,ENSOARG00000002820,ENSOARG00000009936,ENSOARG00000016676	11	0		11
**M4.14**	Rho GTPase cycle	E2F2,ECT2,RACGAP1,CEP55,DEPDC1,ARHGAP11A,DEPDC1B,PRC1,C11orf82,ARHGAP19	cell cycle	ENSOARG00000007334,ENSOARG00000020736,ENSOARG00000018102,ENSOARG00000003158,ENSOARG00000011569,ENSOARG00000019982,ENSOARG00000007132,ENSOARG00000012267	8	2	C11orf82,ARHGAP19	10
**M4.15**	enriched in monocytes (I)	TNFSF13,MYCL1,EPB41L3,DPYSL2,RTN1,SLC31A2,FES,LGALS3,HCK,APLP2,LGALS1	myeloid cells/inflammation	ENSOARG00000014243,ENSOARG00000010473,ENSOARG00000009770,ENSOARG00000021121,ENSOARG00000006395,ENSOARG00000012648,ENSOARG00000021088,ENSOARG00000002820,ENSOARG00000014725,ENSOARG00000013956	10	1	MYCL1	11

**M5.0**	regulation of antigen presentation and immune response	NCKAP1L,THEMIS,JUN,NFKBIA,PTPN6,TLR5,CD4,PRKCQ,PLCG1,CD247,TRBC1,FCGR1B,CCR7,TLR6,TLR10,FYB,TLR2,FCER1G,TRAT1,LILRB2,LILRB1,CLEC7A,BTLA,FYN,FGR,CD86,GRAP2,HLA-DQB1,ZAP70,PAG1,MNDA,IRAK3,LCK,DUSP6,PTPN22,PAK1,CTSH,CTLA4,LCP2,TLR4,CSK,FOS,LAT2,CARD11,CHUK,CD24,UBASH3A,MAP2K6,TLR7,LY96,HCK,VASP,LYN,LIME1,MAPK14,MYD88,INPP5D,ITK,TRAC,SLA2,KLRK1,RPS6KA1,MEF2C,SYK,MEF2A,TLR1,PTPRC,CD19,CD3D,CD3E,BTK,CD3G,DQA,HLA-DOA,HLA-DOB,HLA-DMA,HLA-DRA,OVAR-DM,OVAR-DQA1,OVAR-DQA2,OVAR-DQB1,OVAR-DQB2,OVAR-DRB1,OVAR-DRB1,OVAR-DRB1,OVAR-DRB5,OVAR-DRB3	DC/antigen presentation	ENSOARG00000016133,ENSOARG00000013490,ENSOARG00000009999,ENSOARG00000007502,ENSOARG00000005032,ENSOARG00000005017,ENSOARG00000006301,ENSOARG00000013242,ENSOARG00000001700,ENSOARG00000012086,ENSOARG00000006374,ENSOARG00000014777,ENSOARG00000000552,ENSOARG00000000520,ENSOARG00000009551,ENSOARG00000015044,ENSOARG00000009800,ENSOARG00000019045,ENSOARG00000021015,ENSOARG00000019308,ENSOARG00000009732,ENSOARG00000003365,ENSOARG00000020112,ENSOARG00000017108,ENSOARG00000013646,ENSOARG00000008858,ENSOARG00000002799,ENSOARG00000020968,ENSOARG00000015628,ENSOARG00000019888,ENSOARG00000007238,ENSOARG00000014664,ENSOARG00000018321,ENSOARG00000003167,ENSOARG00000005792,ENSOARG00000003249,ENSOARG00000001783,ENSOARG00000011842,ENSOARG00000004356,ENSOARG00000013829,ENSOARG00000011081,ENSOARG00000010806,ENSOARG00000013869,ENSOARG00000011288,ENSOARG00000006376,ENSOARG00000002820,ENSOARG00000010075,ENSOARG00000014778,ENSOARG00000012870,ENSOARG00000001031,ENSOARG00000019324,ENSOARG00000011171,ENSOARG00000019445,ENSOARG00000015970,ENSOARG00000021000,ENSOARG00000004384,ENSOARG00000016099,ENSOARG00000007814,ENSOARG00000009878,ENSOARG00000000538,ENSOARG00000015718,ENSOARG00000001710,ENSOARG00000009172,ENSOARG00000008984,ENSOARG00000001620,ENSOARG00000009256,ENSOARG00000015485,ENSOARG00000008091,ENSOARG000[illegible]	71	16	FCGR1B,LILRB2,LILRB1,HLA-DQB1,MNDA,LIME1,OVAR-DM,OVAR-DQA1,OVAR-DQA2,OVAR-DQB1,OVAR-DQB2,OVAR-DRB1,OVAR-DRB1,OVAR-DRB1,OVAR-DRB5,OVAR-DRB3	87
**M5.1**	T cell activation and signaling	CD52,CD4,CD247,CD2,CCR7,BIN2,HLA-DRA,IL12RB1,TRAT1,ZAP70,TRAF3IP3,HLA-DRB1,PRKAR1B,GIMAP6,ITK,IL2RG,TRAC,CD3E,PTPRC,PRKACB,CD3D,LCK,CD3G,OVAR-DRB1,OVAR-DRB1,OVAR-DRB1,TRGC2,TRGC3,TRGC1,TRGC4,TRBC1,TRAC,TRDC	T/NK cells	ENSOARG00000004803,ENSOARG00000006301,ENSOARG00000012086,ENSOARG00000020249,ENSOARG00000014777,ENSOARG00000017344,ENSOARG00000016610,ENSOARG00000013918,ENSOARG00000019045,ENSOARG00000013646,ENSOARG00000012918,ENSOARG00000001215,ENSOARG00000001356,ENSOARG00000011171,ENSOARG00000016109,ENSOARG00000019445,ENSOARG00000008984,ENSOARG00000015718,ENSOARG00000013816,ENSOARG00000009172,ENSOARG00000020968,ENSOARG00000009256,ENSOARG00000001797,ENSOARG00000009188,ENSOARG00000017877,ENSOARG00000009129,ENSOARG00000006374,ENSOARG00000019445,ENSOARG00000019448,ENSOARG00000004803,ENSOARG00000006301,ENSOARG00000012086,ENSOARG00000020249,ENSOARG00000014777,ENSOARG00000017344,ENSOARG00000016610,ENSOARG00000013918,ENSOARG00000019045,ENSOARG00000013646,ENSOARG00000012918,ENSOARG00000001215,ENSOARG00000001356,ENSOARG00000011171,ENSOARG00000016109,ENSOARG00000019445,ENSOARG00000008984,ENSOARG00000015718,ENSOARG00000013816,ENSOARG00000009172,ENSOARG00000020968,ENSOARG00000009256,ENSOARG00000017970,ENSOARG00000009188,ENSOARG00000017877,ENSOARG00000009129,ENSOARG00000006374,ENSOARG00000019445,ENSOARG00000019448	29	4	HLA-DRB1,OVAR-DRB1,OVAR-DRB1,OVAR-DRB1	33
**M6**	mitotic cell division	MAD2L1,BUB1B,PLK1,ZWINT,BIRC5,CENPK,CDC20,NDC80,KIF2C,CCDC99,ERCC6L,SKA1,AURKB,CDCA8,CENPO,CENPN,CENPM,BUB1,CENPI,CENPH,CENPF,CENPE,KIF18A,CENPA,SGOL2,CASC5,ZWILCH,NUF2,APITD1,MLF1IP,SPC25,SPC24	cell cycle	ENSOARG00000015665,ENSOARG00000020126,ENSOARG00000017343,ENSOARG00000013084,ENSOARG00000005456,ENSOARG00000006321,ENSOARG00000020542,ENSOARG00000009604,ENSOARG00000001102,ENSOARG00000005908,ENSOARG00000004359,ENSOARG00000019052,ENSOARG00000019830,ENSOARG00000008206,ENSOARG00000018792,ENSOARG00000014305,ENSOARG00000001366,ENSOARG00000005750,ENSOARG00000008368,ENSOARG00000011059,ENSOARG00000015211,ENSOARG00000016031,ENSOARG00000020247,ENSOARG00000018225,ENSOARG00000011189,ENSOARG00000005198,ENSOARG00000004104,ENSOARG00000017725	28	4	CCDC99,CENPO,CENPA,MLF1IP	32

**M7.0**	enriched in T cells (I)	IL7R,KLRB1,CCL5,RASGRP1,SH2D1A,BCL11B,CD6,CD7,CD2,LEF1,NKG7,UBASH3A,PTPRCAP,TRAT1,SLA2,RORA,MAL,EOMES,XCL2,XCL1,ETS1,ZAP70,SAMD3,SIT1,NLRC3,SIRPG,PRKCH,ICOS,CD28,NELL2,GNLY,CD27,LAT,FLT3LG,PRF1,PRKCQ,GIMAP6,GIMAP7,GATA3,ITK,LCK,GZMA,GZMB,CD96,GZMM,GZMH,TIGIT,GZMK,ITM2A,CD247,GPR171,IL32,TARP,CD3D,CD3E,CD3G,TRGC2,TRGC3,TRGC1,TRGC4,TRBC1,TRAC,TRDC	T/NK cells	ENSOARG00000011239,ENSOARG00000017210,ENSOARG00000004964,ENSOARG00000020044,ENSOARG00000014085,ENSOARG00000000266,ENSOARG00000016062,ENSOARG00000016754,ENSOARG00000020249,ENSOARG00000008064,ENSOARG00000014768,ENSOARG00000010806,ENSOARG00000011983,ENSOARG00000019045,ENSOARG00000015970,ENSOARG00000020816,ENSOARG00000014467,ENSOARG00000009179,ENSOARG00000013102,ENSOARG00000013646,ENSOARG00000013826,ENSOARG00000011944,ENSOARG00000001980,ENSOARG00000021134,ENSOARG00000018331,ENSOARG00000018277,ENSOARG00000019622,ENSOARG00000008332,ENSOARG00000001158,ENSOARG00000013140,ENSOARG00000006139,ENSOARG00000013242,ENSOARG00000001356,ENSOARG00000013941,ENSOARG00000011171,ENSOARG00000020968,ENSOARG00000007970,ENSOARG00000002990,ENSOARG00000019179,ENSOARG00000008703,ENSOARG00000019616,ENSOARG00000007987,ENSOARG00000000398,ENSOARG00000012086,ENSOARG00000015348,ENSOARG00000002464,ENSOARG00000009172,ENSOARG00000008984,ENSOARG00000009256,ENSOARG00000001797,ENSOARG00000009188,ENSOARG00000017877,ENSOARG00000009129,ENSOARG00000006374,ENSOARG00000019445,ENSOARG00000019448, ENSOARG00000011239,ENSOARG00000017210,ENSOARG00000004964,ENSOARG00000020044,ENSOARG00000014085,ENSOARG00000000266,ENSOARG00000016062,ENSOARG00000016754,ENSOARG000 [illegible]	56	7	MAL,XCL2,SIRPG,GNLY,GIMAP7,GZMH,TARP	63
**M7.1**	T cell activation (I)	IL7R,RASGRP1,THEMIS,CCL5,TCF7,BCL11B,CD5,TIGIT,CD7,CD2,CCR7,CD247,MS4A1,PTPN22,TXK,TRBC1,IL12RB1,IKZF1,SLA2,CAMK4,RORA,EOMES,PRKCQ,ZAP70,SIT1,NLRC3,DPP4,SIRPG,PRKCH,ICOS,CD28,CD27,RHOH,CD8A,ITPR3,LEF1,CD8B,GATA3,CRTAM,ITK,TRAC,KLRK1,IL21R,LCK,CORO1A,SLAMF7,CD3D,CD3E,SLAMF1,CD3G	T/NK cells	ENSOARG00000011239,ENSOARG00000020044,ENSOARG00000013490,ENSOARG00000004964,ENSOARG00000013765,ENSOARG00000000266,ENSOARG00000016138,ENSOARG00000019616,ENSOARG00000016754,ENSOARG00000020249,ENSOARG00000014777,ENSOARG00000012086,ENSOARG00000013081,ENSOARG00000019888,ENSOARG00000016822,ENSOARG00000006374,ENSOARG00000013918,ENSOARG00000014822,ENSOARG00000015970,ENSOARG00000000184,ENSOARG00000020816,ENSOARG00000014467,ENSOARG00000013242,ENSOARG00000013646,ENSOARG00000011944,ENSOARG00000001980,ENSOARG00000006454,ENSOARG00000021134,ENSOARG00000018331,ENSOARG00000018277,ENSOARG00000008332,ENSOARG00000000571,ENSOARG00000020734,ENSOARG00000010180,ENSOARG00000008064,ENSOARG00000020747,ENSOARG00000013941,ENSOARG00000018699,ENSOARG00000011171,ENSOARG00000019445,ENSOARG00000021000,ENSOARG00000018894,ENSOARG00000020968,ENSOARG00000003604,ENSOARG00000008967,ENSOARG00000009172,ENSOARG00000008984,ENSOARG00000008893,ENSOARG00000009256, ENSOARG00000011239,ENSOARG00000020044,ENSOARG00000013490,ENSOARG00000004964,ENSOARG00000013765,ENSOARG00000000266,ENSOARG00000016138,ENSOARG00000019616,ENSOARG00000016754,ENSOARG00000020249,ENSOARG00000014777,ENSOARG00000012086,ENSOARG00000013081,ENSOARG00000019888,ENSOARG00000016822,ENSOARG00000006374,ENSOARG00000013918,ENS [illegible]	49	1	SIRPG	50

**M7.2**	enriched in NK cells (I)	TGFBR3,KLRB1,KLRD1,CCL5,TBX21,FCGR3B,PTGDR,CD247,NLRC3,GPR56,MYBL1,ARL4C,KLRF1,STAT4,PLEKHF1,IL18RAP,RORA,EOMES,XCL2,XCL1,FGFBP2,SAMD3,CTSW,CST7,ZAP70,KLRC1,PRKCH,S1PR5,CLIC3,GNLY,SH2D2A,FASLG,PRF1,PRKCQ,GIMAP7,GZMA,IL2RB,HOPX,ITK,GZMB,NKG7,CD96,GZMM,GZMH,KLRK1,SLAMF7,TARP,TRAC,TRDC	T/NK cells	ENSOARG00000016047,ENSOARG00000017210,ENSOARG00000004964,ENSOARG00000008340,ENSOARG00000020714,ENSOARG00000012086,ENSOARG00000001980,ENSOARG00000017663,ENSOARG00000025194,ENSOARG00000021058,ENSOARG00000013903,ENSOARG00000004052,ENSOARG00000013079,ENSOARG00000020816,ENSOARG00000014467,ENSOARG00000009179,ENSOARG00000013826,ENSOARG00000018487,ENSOARG00000006327,ENSOARG00000013646,ENSOARG00000021134,ENSOARG00000006400,ENSOARG00000014582,ENSOARG00000006139,ENSOARG00000013242,ENSOARG00000007970,ENSOARG00000019197,ENSOARG00000004859,ENSOARG00000011171,ENSOARG00000002990,ENSOARG00000014768,ENSOARG00000019179,ENSOARG00000008703,ENSOARG00000021000,ENSOARG00000008967,ENSOARG00000019445,ENSOARG00000019448	37	12	KLRD1,FCGR3B,GPR56,XCL2,FGFBP2,KLRC1,S1PR5,CLIC3,GNLY,GIMAP7,GZMH,TARP	49
**M7.3**	T cell activation (II)	CCL5,SP140,TIGIT,CD247,CCR5,NLRC3,PTPRCAP,STAT4,IL12RB1,TRAT1,SLA2,CXCR3,ZAP70,NKG7,SIRPG,ICOS,IFNG,IL18R1,SLAMF7,PTPN7,ITK,CRTAM,GZMA,CD3E,GPR171,TARP,CD3D,LCK,SLAMF1,CD3G,TRAC	T/NK cells	ENSOARG00000004964,ENSOARG00000020646,ENSOARG00000019616,ENSOARG00000012086,ENSOARG00000014214,ENSOARG00000001980,ENSOARG00000011983,ENSOARG00000013903,ENSOARG00000013918,ENSOARG00000019045,ENSOARG00000015970,ENSOARG00000017562,ENSOARG00000013646,ENSOARG00000014768,ENSOARG00000018331,ENSOARG00000001958,ENSOARG00000013096,ENSOARG00000008967,ENSOARG00000000687,ENSOARG00000011171,ENSOARG00000018699,ENSOARG00000007970,ENSOARG00000008984,ENSOARG00000015348,ENSOARG00000009172,ENSOARG00000020968,ENSOARG00000008893,ENSOARG00000009256,ENSOARG00000019445	29	2	SIRPG,TARP	31
**M7.4**	T cell activation (III)	KLF12,RASGRP1,THEMIS,NELL2,CD96,CAMK4,RORA,LCK,ATP8B2,ETS1,ZMYND11,PRKCQ,LEF1,GLYR1,SEPT6	T/NK cells	ENSOARG00000015513,ENSOARG00000020044,ENSOARG00000013490,ENSOARG00000019622,ENSOARG00000019179,ENSOARG00000000184,ENSOARG00000020816,ENSOARG00000020968,ENSOARG00000002448,ENSOARG00000013102,ENSOARG00000016155,ENSOARG00000013242,ENSOARG00000008064,ENSOARG00000004173,ENSOARG00000016123	15	0		15
**M8**	E2F transcription factor network	BRCA1,CCNA2,E2F2,E2F1,MELK,ORC1L,ZWINT,CDC25A,ERCC6L,ORC6L,C12orf48,KIAA1524,RAD54L,CDCA5,UBE2C,KIAA0101,FAM54A,CDC6	cell cycle	ENSOARG00000004835,ENSOARG00000014176,ENSOARG00000007334,ENSOARG00000008548,ENSOARG00000011541,ENSOARG00000013084,ENSOARG00000002373,ENSOARG00000005908,ENSOARG00000018970,ENSOARG00000002264,ENSOARG00000012000,ENSOARG00000006520,ENSOARG00000014063	13	5	ORC1L,ORC6L,C12orf48,KIAA0101,FAM54A	18
**M9**	B cell development	TNFSF13,LIF,HHEX,CEBPE,CBFA2T3,LTA,SPI1,TLR4,CCR7,LYN,TNF	B cells	ENSOARG00000014243,ENSOARG00000006322,ENSOARG00000019360,ENSOARG00000013723,ENSOARG00000008484,ENSOARG00000006553,ENSOARG00000005792,ENSOARG00000014777,ENSOARG00000014778,ENSOARG00000008333	10	1	HHEX	11

**M10.0**	E2F1 targets (Q3)	MAP4K1,STMN1,ERBB2IP,RFC1,TLE4,EZH2,FBXO5,GMNN,ARID4A,ARHGAP6,KIAA0101,CDC6,DCK,MEIS2,SFRS1,WEE1,RANBP1,PCNA,NR3C2,ZNF367,HMGA1,MCM6,MCM4,MCM2,UNG,OSBPL7,HIST1H2BK,E2F7,ATAD2,MELK,POLE2,RAD51,HOXA9	cell cycle	ENSOARG00000005746,ENSOARG00000012293,ENSOARG00000006023,ENSOARG00000011358,ENSOARG00000012336,ENSOARG00000018856,ENSOARG00000003968,ENSOARG00000005900,ENSOARG00000021110,ENSOARG00000010821,ENSOARG00000014063,ENSOARG00000012101,ENSOARG00000020017,ENSOARG00000012355,ENSOARG00000018318,ENSOARG00000017133,ENSOARG00000008474,ENSOARG00000010561,ENSOARG00000010614,ENSOARG00000012797,ENSOARG00000002774,ENSOARG00000016873,ENSOARG00000008101,ENSOARG00000014941,ENSOARG00000010247,ENSOARG00000011541,ENSOARG00000020607,ENSOARG00000020254,ENSOARG00000009680, ENSOARG00000005746,ENSOARG00000012293,ENSOARG00000006023,ENSOARG00000011358,ENSOARG00000012336,ENSOARG00000018856,ENSOARG00000003968,ENSOARG00000005900,ENSOARG00000021110,ENSOARG00000010821,ENSOARG00000014063,ENSOARG00000012101,ENSOARG00000020017,ENSOARG00000012355,ENSOARG00000018318,ENSOARG00000017133,ENSOARG00000008474,ENSOARG00000010561,ENSOARG00000010614,ENSOARG00000012797,ENSOARG00000002774,ENSOARG00000016873,ENSOARG00000008101,ENSOARG00000014941,ENSOARG00000010247,ENSOARG00000011541,ENSOARG00000020607,ENSOARG00000020254,ENSOARG00000009680	29	4	KIAA0101,SFRS1,NR3C2,HIST1H2BK	33
**M10.1**	E2F1 targets (Q4)	E2F7,PCNA,NASP,POLE2,SKP2,ERBB2IP,SLBP,POLA2,KNTC1,PKMYT1,MELK,MCM4,PRIM1,FBXO5,RAD51,CIT,STMN1,WEE1,RANBP1,CDC6,DCLRE1A	cell cycle	ENSOARG00000014941,ENSOARG00000017133,ENSOARG00000020607,ENSOARG00000006023,ENSOARG00000016090,ENSOARG00000014045,ENSOARG00000009349,ENSOARG00000000736,ENSOARG00000011541,ENSOARG00000012797,ENSOARG00000008185,ENSOARG00000003968,ENSOARG00000020254,ENSOARG00000000627,ENSOARG00000012293,ENSOARG00000012355,ENSOARG00000018318,ENSOARG00000014063,ENSOARG00000013383	19	2	NASP,SKP2	21
**M11.0**	enriched in monocytes (II)	FXYD6,KCNE3,FAM198B,HSPA6,LAT2,CD33,TIMP2,CD36,TNFSF12-TNFSF13,C5AR1,MARCH1,FCGR1B,FCGR1A,SLC15A3,S100A11,MS4A7,MAFB,S100A12,CPPED1,TYROBP,FGR,PLA2G7,IRAK3,LYN,PYGL,SIRPA,FCAR,CRISPLD2,RNASE2,TBXAS1,PLBD1,HK3,FPR2,NOD2,IFI30,AQP9,IMPA2,LRP1,TMEM176A,CSF3R,RBP7,CACNA2D3,MS4A4A,HPSE,KCTD12,SMPDL3A,LST1,RBM47,SYK,SECTM1,PLXDC2,TLR2,CD163,BST1,TLR4,GLT1D1,TREM1,TLR8,FBP1,TNFSF13,CD1D,CSF2RA,SLC7A7,EMR2,RASSF4,SLC31A2,MGST1,IFNGR2,RIN2,LY86,PLXNB2,LILRB3,LILRB2,LILRB1,CLEC7A,QPCT,RNF130,PILRA,NFE2,TNS3,HNMT,GCA,FCN1,TFEC,VNN2,VNN3,TLR5,RTN1,NCF1C,GAS2L1,GRN,BLVRB,ITGAX,MS4A6A,IL13RA1,CD93,TNFAIP2,CD14,ITGAM,SIRPB1,SORT1,AIF1,BTK,NCF2,NCF1,MYCL1,CCR1,CD302,PTAFR,HCK,STAB1,P2RY13,FCER1G,SPI1,CYP1B1,NLRP3,CSF1R,CD86,SCPEP1,ADAP2,S100A9,S100A8,CST3,KIAA0513,EPB41L3,MTMR11,CSTA,RXRA,ASGR1,ASGR2,FES,LGALS2,PRAM1,KIAA1598,CDA,SGK1,LMO2,ALD[illegible]	myeloid cells/inflammation	ENSOARG00000007061,ENSOARG00000006737,ENSOARG00000006033,ENSOARG00000007452,ENSOARG00000011842,ENSOARG00000003264,ENSOARG00000017123,ENSOARG00000011005,ENSOARG00000013445,ENSOARG00000020667,ENSOARG00000015974,ENSOARG00000021079,ENSOARG00000012815,ENSOARG00000001355,ENSOARG00000000432,ENSOARG00000005167,ENSOARG00000003365,ENSOARG00000011007,ENSOARG00000002799,ENSOARG00000014778,ENSOARG00000020686,ENSOARG00000007425,ENSOARG00000002196,ENSOARG00000011051,ENSOARG00000011915,ENSOARG00000020776,ENSOARG00000003252,ENSOARG00000017441,ENSOARG00000013291,ENSOARG00000020858,ENSOARG00000001725,ENSOARG00000007332,ENSOARG00000001756,ENSOARG00000019729,ENSOARG00000015530,ENSOARG00000002360,ENSOARG00000015960,ENSOARG00000008143,ENSOARG00000008167,ENSOARG00000012711,ENSOARG00000007814,ENSOARG00000000344,ENSOARG00000015044,ENSOARG00000002862,ENSOARG00000009499,ENSOARG00000005792,ENSOARG00000016604,ENSOARG00000001869,ENSOARG00000018099,ENSOARG00000008650,ENSOARG00000014243,ENSOARG00000007252,ENSOARG00000007541,ENSOARG00000019424,ENSOARG00000002866,ENSOARG00000006395,ENSOARG00000020582,ENSOARG00000013168,ENSOARG00000001865,ENSOARG00000018347,ENSOARG00000019806,ENSOARG00000021015,ENSOARG00000009260,ENSOARG00000001974,ENSOARG00000016228,ENSOARG00000012535,ENSOARG[illegible]	160	29	CD33,TNFSF12-TNFSF13,FCGR1B,CPPED1,RNASE2,FPR2,RBP7,MS4A4A,SECTM1,EMR2,LILRB3,LILRB2,LILRB1,PILRA,FCN1,VNN3,MS4A6A,TNFAIP2,SIRPB1,MYCL1,KIAA1598,APOB48R,SEPX1,FPR1,MNDA,FCGR2A,TYMP,LILRA2,LILRA3	189

**M11.1**	blood coagulation	SLC7A7,AMICA1,CD36,TREM1,P2RX1,F13A1,PECAM1,FCER1G,DOK2,PLAUR,LYN,SIRPA,SERPINA1,GNB4,SERPINB2,ITGAX,F5,SYK,SLC16A3,PSAP,ITGAM,CFD	myeloid cells/inflammation	ENSOARG00000019424,ENSOARG00000017123,ENSOARG00000001869,ENSOARG00000017892,ENSOARG00000018445,ENSOARG00000014779,ENSOARG00000009800,ENSOARG00000010631,ENSOARG00000008867,ENSOARG00000014778,ENSOARG00000007425,ENSOARG00000014882,ENSOARG00000020697,ENSOARG00000006889,ENSOARG00000009130,ENSOARG00000010135,ENSOARG00000007814,ENSOARG00000016912,ENSOARG00000006957,ENSOARG00000009000,ENSOARG00000009779	21	1	AMICA1	22
**M11.2**	formyl peptide receptor mediated neutrophil response	NCF2,SLC11A1,CAMK1,FPR1,IGSF6,SECTM1,FCGR2A,PILRA,NCF1C,PAK1	myeloid cells/inflammation	ENSOARG00000001695,ENSOARG00000019535,ENSOARG00000006159,ENSOARG00000015971,ENSOARG00000011046,ENSOARG00000007238	6	4	FPR1,SECTM1,FCGR2A,PILRA	10
**M12**	CD28 costimulation	CD28,CHST15,LIME1,SH2D1A,TRAT1,CD24,GRAP2,THEM4,LAT,SPNS1,LCK	T/NK cells	ENSOARG00000018277,ENSOARG00000009887,ENSOARG00000014085,ENSOARG00000019045,ENSOARG00000011081,ENSOARG00000017108,ENSOARG00000021072,ENSOARG00000001158,ENSOARG00000001386,ENSOARG00000020968	10	1	LIME1	11
**M13**	innate activation by cytosolic DNA sensing	DDX58,IRF7,CCL4,CCL5,ZBP1,NFKBIA,AIM2,IL6,PYCARD,CXCL10,IL1B	IFN type I	ENSOARG00000014731,ENSOARG00000006626,ENSOARG00000004253,ENSOARG00000004964,ENSOARG00000017418,ENSOARG00000007502,ENSOARG00000012021,ENSOARG00000008908,ENSOARG00000016611,ENSOARG00000020866, ENSOARG00000014731,ENSOARG00000006626,ENSOARG00000004253,ENSOARG00000004964,ENSOARG00000017418,ENSOARG00000007502,ENSOARG00000012021,ENSOARG00000008908,ENSOARG00000016611,ENSOARG00000020866	10	1	AIM2	11
**M14**	T cell differentiation	UBASH3A,TCF7,CD28,TXK,ITK,NOG,LRRN3,BCL11B,RASGRP1,SPOCK2,SH2D1A,MAL	T/NK cells	ENSOARG00000010806,ENSOARG00000013765,ENSOARG00000018277,ENSOARG00000016822,ENSOARG00000011171,ENSOARG00000013752,ENSOARG00000003374,ENSOARG00000000266,ENSOARG00000020044,ENSOARG00000007029,ENSOARG00000014085	11	1	MAL	12
**M15**	Ran mediated mitosis	TPX2,BUB1B,PLK1,H2AFX,KIF15,RAN,ESPL1,AURKA,AURKB,UBE2S,RANBP1,KIF2C,KPNA2	cell cycle	ENSOARG00000001419,ENSOARG00000020126,ENSOARG00000017343,ENSOARG00000017039,ENSOARG00000004780,ENSOARG00000010707,ENSOARG00000016587,ENSOARG00000018369,ENSOARG00000019052,ENSOARG00000001369,ENSOARG00000018318,ENSOARG00000001102,ENSOARG00000015347	13	0		13
**M16**	TLR and inflammatory signaling	FCGR1A,FCGR1C,HSPA6,NCF4,TLR6,C5AR1,FCGR1B,APOB48R,LILRB3,KCNJ15,LILRB1,PILRA,TYROBP,MGAM,FGR,P2RY13,CXCR2,NFE2,IRAK3,MYD88,FPR2,SIGLEC9,FCAR,DYSF,NPL,FPR1,ALOX5,FCGR2C,AQP9,FES,CSF3R,TLR7,LY96,LILRB2,ITGAX,KCNJ2,WDFY3,TLR2,TLR1,BST1,ANPEP,TLR4,TLR5,LILRA6,TLR8,PADI4	myeloid cells/inflammation	ENSOARG00000020667,ENSOARG00000007452,ENSOARG00000019050,ENSOARG00000000552,ENSOARG00000011005,ENSOARG00000014447,ENSOARG00000005167,ENSOARG00000014509,ENSOARG00000003365,ENSOARG00000004209,ENSOARG00000019477,ENSOARG00000016228,ENSOARG00000002799,ENSOARG00000001031,ENSOARG00000014547,ENSOARG00000002196,ENSOARG00000011500,ENSOARG00000002819,ENSOARG00000020858,ENSOARG00000012648,ENSOARG00000019729,ENSOARG00000011288,ENSOARG00000006376,ENSOARG00000009130,ENSOARG00000013847,ENSOARG00000003468,ENSOARG00000015044,ENSOARG00000000538,ENSOARG00000009499,ENSOARG00000011841,ENSOARG00000005792,ENSOARG00000005017,ENSOARG00000002741,ENSOARG00000018099,ENSOARG00000011018	35	11	FCGR1C,FCGR1B,APOB48R,LILRB3,LILRB1,PILRA,FPR2,NPL,FPR1,FCGR2C,LILRB2	46
**M17.0**	Hox cluster I	CPNE8,HOXB9,LOC404266,HOXB2,HOXB6,HOXB4,HOXA10,HOXA7,HOXA5,HOXA4,HOXA3,MEIS1,NKX2-3,ZNF503,ITGA7,C10orf140	others	ENSOARG00000020074,ENSOARG00000006872,ENSOARG00000007134,ENSOARG00000007002,ENSOARG00000007067,ENSOARG00000009675,ENSOARG00000009717,ENSOARG00000009789,ENSOARG00000009797,ENSOARG00000019962,ENSOARG00000012498,ENSOARG00000011427	12	4	LOC404266,HOXA7,ZNF503,C10orf140	16
**M17.1**	Hox cluster II	PRDM16,PDGFD,HOXB3,HOXB6,HOXB7,HOXA11,HOXB5,HOXA5,HOXA4,HOXA3,TTR	others	ENSOARG00000017237,ENSOARG00000003293,ENSOARG00000007106,ENSOARG00000007002,ENSOARG00000006908,ENSOARG00000007034,ENSOARG00000009717,ENSOARG00000009789,ENSOARG00000009797,ENSOARG00000006342	10	1	HOXA11	11
**M17.2**	Hox cluster III	HOXB3,HOXB6,HOXB7,HOXA11,HOXA10,HOXA7,MEIS1,HOXA5,HOXA4,HOXB4,MEIS2,HOXA9	others	ENSOARG00000007106,ENSOARG00000007002,ENSOARG00000006908,ENSOARG00000009675,ENSOARG00000019962,ENSOARG00000009717,ENSOARG00000009789,ENSOARG00000007067,ENSOARG00000020017,ENSOARG00000009680	10	2	HOXA11,HOXA7	12

**M17.3**	Hox cluster IV	HOXB9,HOXB2,HOXB6,HOXB7,HOXB4,HOXB5,HOXA5,HOXA4,HOXA11,HOXA9	others	ENSOARG00000006872,ENSOARG00000007134,ENSOARG00000007002,ENSOARG00000006908,ENSOARG00000007067,ENSOARG00000007034,ENSOARG00000009717,ENSOARG00000009789,ENSOARG00000009680	9	1	HOXA11	10
**M18**	T cell differentiation via ITK and PKC	SIRPG,PRKCH,ITK,SH2D1A,ITM2A,ETS1,FASLG,SPOCK2,PRKCQ,ABLIM1,BCL2	T/NK cells	ENSOARG00000021134,ENSOARG00000011171,ENSOARG00000014085,ENSOARG00000000398,ENSOARG00000013102,ENSOARG00000014582,ENSOARG00000007029,ENSOARG00000013242,ENSOARG00000015139,ENSOARG00000006262	10	1	SIRPG	11
**M19**	T cell differentiation (Th2)	UBASH3A,CD28,LCK,TRAT1,PKIA,HNRPLL,GZMK,LAT,ITM2A,GRAP2,IL32,GPR171,SIT1,DPP4,GATA3,MAL,CD3G	T/NK cells	ENSOARG00000010806,ENSOARG00000018277,ENSOARG00000020968,ENSOARG00000019045,ENSOARG00000008915,ENSOARG00000007987,ENSOARG00000001158,ENSOARG00000000398,ENSOARG00000017108,ENSOARG00000002464,ENSOARG00000015348,ENSOARG00000011944,ENSOARG00000006454,ENSOARG00000013941,ENSOARG00000009256	15	2	PKIA,MAL	17
**M20**	AP-1 transcription factor network	CCL2,MMP9,JUNB,FOS,IFNG,FOSB,EGR1,JUN,PLAU,IL6,FOSL1,CXCL8,FOSL2,ATF3,MMP1	cell cycle	ENSOARG00000009627,ENSOARG00000007908,ENSOARG00000010854,ENSOARG00000001783,ENSOARG00000001958,ENSOARG00000009983,ENSOARG00000016470,ENSOARG00000009999,ENSOARG00000008473,ENSOARG00000012021,ENSOARG00000000072,ENSOARG00000014496,ENSOARG00000019483,ENSOARG00000010493,ENSOARG00000005315	15	0		15
**M21**	cell adhesion (lymphocyte homing)	SELL,SDK2,PTK2,CD72,MLLT4,CD24,VAV1,IL32,CCR7,CXCL13	myeloid cells/inflammation	ENSOARG00000010897,ENSOARG00000013295,ENSOARG00000003418,ENSOARG00000011957,ENSOARG00000004988,ENSOARG00000011081,ENSOARG00000004983,ENSOARG00000002464,ENSOARG00000014777,ENSOARG00000017732, ENSOARG00000010897,ENSOARG00000013295,ENSOARG00000003418,ENSOARG00000011957,ENSOARG00000004988,ENSOARG00000011081,ENSOARG00000004983,ENSOARG00000002464,ENSOARG00000014777,ENSOARG00000017732	10	0		10
**M22.0**	mismatch repair (I)	SMC1A,POLA1,NCAPG2,RFC5,RFC4,MSH2,TMPO,MSH6,RFC2,GMNN,BUB1,RMI1,RACGAP1,EXO1,POLD3,PRIM1,ZWINT,CHEK1,PCNA,CENPK,FIGNL1,MCM6,RFC3,SSBP1,TOPBP1,RPA3,SMC2	cell cycle	ENSOARG00000008581,ENSOARG00000017574,ENSOARG00000007995,ENSOARG00000003356,ENSOARG00000020512,ENSOARG00000005136,ENSOARG00000012899,ENSOARG00000005046,ENSOARG00000011737,ENSOARG00000005900,ENSOARG00000014305,ENSOARG00000003498,ENSOARG00000018102,ENSOARG00000007232,ENSOARG00000010224, ENSOARG00000008185,ENSOARG00000013084,ENSOARG00000011655,ENSOARG00000017133,ENSOARG00000006321,ENSOARG00000008066,ENSOARG00000010614,ENSOARG00000010697,ENSOARG00000014046,ENSOARG00000008582,ENSOARG00000005725,ENSOARG00000007399	27	0		27
**M22.1**	mismatch repair (II)	PCNA,RFC3,RFC5,RFC4,MSH2,EXO1,RPA1,MSH6,RFC2,POLD1,POLD3,MLH1,RPA3	cell cycle	ENSOARG00000017133,ENSOARG00000010697,ENSOARG00000003356,ENSOARG00000020512,ENSOARG00000005136,ENSOARG00000007232,ENSOARG00000014479,ENSOARG00000005046,ENSOARG00000011737,ENSOARG00000013923,ENSOARG00000010224,ENSOARG00000016550,ENSOARG00000005725	13	0		13
**M23**	RA, WNT, CSF receptors network (monocyte)	RARA,FZD1,CEBPA,CSF2RA,FOS,CSF1R,FZD2,RXRA,SPI1,PTGS2,CSF3R,CXCL8	myeloid cells/inflammation	ENSOARG00000014119,ENSOARG00000016629,ENSOARG00000004414,ENSOARG00000007541,ENSOARG00000001783,ENSOARG00000006358,ENSOARG00000008653,ENSOARG00000002366,ENSOARG00000006553,ENSOARG00000007592,ENSOARG00000019729,ENSOARG00000014496	12	0		12
**M24**	cell activation (IL15, IL23, TNF)	SBNO2,MAP3K8,IL23A,FN1,CD83,PDGFRA,IL15,TNFAIP3,SERPING1,TNF,TF,COL3A1,NFKB2,A2M,BCL3	inflammation	ENSOARG00000011130,ENSOARG00000015050,ENSOARG00000009246,ENSOARG00000019329,ENSOARG00000012936,ENSOARG00000019070,ENSOARG00000012119,ENSOARG00000000569,ENSOARG00000010035,ENSOARG00000008333,ENSOARG00000008456,ENSOARG00000016476,ENSOARG00000000483,ENSOARG00000000950,ENSOARG00000009239	15	0		15
**M25**	TLR8-BAFF network	SLC40A1,TNFSF13B,ASGR1,ASGR2,PCTP,AP1S2,TLR8,BCL6,IL1R2,TBXAS1	myeloid cells/inflammation	ENSOARG00000016390,ENSOARG00000005617,ENSOARG00000010137,ENSOARG00000010000,ENSOARG00000012817,ENSOARG00000018099,ENSOARG00000020487,ENSOARG00000013159,ENSOARG00000011915	9	1	PCTP	10

**M26.0**	TBA	TRIM15,CDH17,CDX1,CDX2,MYO1A,FABP1,MUC3B,CRABP2,SLC39A5,VIL1,USH1C,IL22RA1,CLRN3,HMGCS2,GUCY2C,TINAG,DDC,GIPC2,EPS8L3,PPP1R14D,LGALS4,LRRC19,HNF4G,HNF4A,CDHR5,A1CF,NR1I2,RNF128,GPA33,CCL14-CCL15,CCL15	others	ENSOARG00000015773,ENSOARG00000008009,ENSOARG00000006690,ENSOARG00000012435,ENSOARG00000007766,ENSOARG00000020752,ENSOARG00000006212,ENSOARG00000009687,ENSOARG00000019575,ENSOARG00000003142,ENSOARG00000006788,ENSOARG00000014062,ENSOARG00000020427,ENSOARG00000020763,ENSOARG00000006707,ENSOARG00000014391,ENSOARG00000013390,ENSOARG00000019334,ENSOARG00000020288,ENSOARG00000005839,ENSOARG00000014554,ENSOARG00000007140,ENSOARG00000003928,ENSOARG00000006446,ENSOARG00000013695,ENSOARG00000019817,ENSOARG00000018913,ENSOARG00000011969,ENSOARG00000004774	29	2	MUC3B,CCL14-CCL15	31
**M26.1**	TBA	TRIM15,UGT2A3,CYP3A4,TM4SF5,VIL1,USH1C,CLRN3,TINAG,DDC,CYP3A7,GIPC2,CYP3A5,LGALS4,ARSE,LRRC19,HNF4G,RNF186,HNF4A,CDHR5,RNF128,A1CF,CCL14-CCL15,CCL15	others	ENSOARG00000015773,ENSOARG00000009742,ENSOARG00000007152,ENSOARG00000019575,ENSOARG00000003142,ENSOARG00000014062,ENSOARG00000006707,ENSOARG00000014391,ENSOARG00000013390,ENSOARG00000005839,ENSOARG00000008499,ENSOARG00000014554,ENSOARG00000007140,ENSOARG00000009608,ENSOARG00000003928,ENSOARG00000006446,ENSOARG00000018913,ENSOARG00000013695,ENSOARG00000004774	19	4	CYP3A4,CYP3A7,CYP3A5,CCL14-CCL15	23
**M26.2**	TBA	TRIM15,LRRC19,HNF4G,HNF4A,VIL1,CLRN3,FOXA3,RNF128,DDC,USH1C,CLDN2,A1CF,CCL14-CCL15,CCL15,LGALS4	others	ENSOARG00000015773,ENSOARG00000014554,ENSOARG00000007140,ENSOARG00000003928,ENSOARG00000019575,ENSOARG00000014062,ENSOARG00000018913,ENSOARG00000014391,ENSOARG00000003142,ENSOARG00000018869,ENSOARG00000013695,ENSOARG00000004774,ENSOARG00000005839	13	2	FOXA3,CCL14-CCL15	15
**M27.0**	chemokine cluster (I)	CCL2,CCL1,CCL7,CCL4,CCL5,CCL8,PF4,XCL1,PPBP,CCL20,CCL23,IFNG,CXCL2,CXCL11,CXCL10,CXCL13,CXCL12,CXCL1,CXCL3,CCL13,CXCL5,CXCL6,CCL18,CCL19,CXCL9,IL8	myeloid cells/inflammation	ENSOARG00000009627,ENSOARG00000009184,ENSOARG00000004253,ENSOARG00000004964,ENSOARG00000009241,ENSOARG00000009179,ENSOARG00000014675,ENSOARG00000020576,ENSOARG00000001958,ENSOARG00000014775,ENSOARG00000016668,ENSOARG00000016611,ENSOARG00000017732,ENSOARG00000002991,ENSOARG00000014775,ENSOARG00000014841,ENSOARG00000014592,ENSOARG00000009119,ENSOARG00000016543,ENSOARG00000014496, ENSOARG00000009627,ENSOARG00000009184,ENSOARG00000004253,ENSOARG00000004964,ENSOARG00000009241,ENSOARG00000009179,ENSOARG00000014675,ENSOARG00000020576,ENSOARG00000001958,ENSOARG00000014775,ENSOARG00000016668,ENSOARG00000016611,ENSOARG00000017732,ENSOARG00000002991,ENSOARG00000014775,ENSOARG00000014841,ENSOARG00000014592,ENSOARG00000009119,ENSOARG00000016543,ENSOARG00000014496	20	6	CCL7,PF4,CCL23,CCL13,CXCL6,CCL18	26
**M27.1**	chemokine cluster (II)	ANXA1,CCL2,CXCL5,CCL7,CCL4,CCL5,CXCL9,CXCL6,CCR1,XCL1,CCR5,CXCL10,CXCL11,CCL20,CXCL13	myeloid cells/inflammation	ENSOARG00000012672,ENSOARG00000009627,ENSOARG00000014592,ENSOARG00000004253,ENSOARG00000004964,ENSOARG00000016543,ENSOARG00000014260,ENSOARG00000009179,ENSOARG00000014214,ENSOARG00000016611,ENSOARG00000016668,ENSOARG00000020576,ENSOARG00000017732, ENSOARG00000012672,ENSOARG00000009627,ENSOARG00000014592,ENSOARG00000004253,ENSOARG00000004964,ENSOARG00000016543,ENSOARG00000014260,ENSOARG00000009179,ENSOARG00000014214,ENSOARG00000016611,ENSOARG00000016668,ENSOARG00000020576,ENSOARG00000017732	13	2	CCL7,CXCL6	15

**M28**	antigen presentation (lipids and proteins)	CD36,CCR7,CD8A,HLA-DRA,OVAR-DRB1,OVAR-DRB1,OVAR-DRB1,OVAR-DRB5,OVAR-DRB3,CD1E,CD1A,CD1B,CD1D	DC/antigen presentation	ENSOARG00000017123,ENSOARG00000014777,ENSOARG00000020734,ENSOARG00000016610,ENSOARG00000007307,ENSOARG00000007267,ENSOARG00000007282,ENSOARG00000007252, ENSOARG00000017123,ENSOARG00000014777,ENSOARG00000020734,ENSOARG00000016610,ENSOARG00000007307,ENSOARG00000007322,ENSOARG00000007291,ENSOARG00000007252, ENSOARG00000017123,ENSOARG00000014777,ENSOARG00000020734,ENSOARG00000016610,ENSOARG00000007307,ENSOARG00000007267,ENSOARG00000001986,ENSOARG00000007252, ENSOARG00000017123,ENSOARG00000014777,ENSOARG00000020734,ENSOARG00000016610,ENSOARG00000007307,ENSOARG00000007322,ENSOARG00000001657,ENSOARG00000007252, ENSOARG00000017123,ENSOARG00000014777,ENSOARG00000020734,ENSOARG00000016610,ENSOARG00000007307,ENSOARG00000007267,ENSOARG00000002532,ENSOARG00000007252, ENSOARG00000017123,ENSOARG00000014777,ENSOARG00000020734,ENSOARG00000016610,ENSOARG00000007307,ENSOARG00000007322,ENSOARG00000002614,ENSOARG00000007252, ENSOARG00000017123,ENSOARG00000014777,ENSOARG00000020734,ENSOARG00000016610,ENSOARG00000007307,ENSOARG00000007267,ENSOARG00000002853,ENSOARG00000007252, ENSOARG00000017123,ENSOARG00000014777,ENSOARG00000020734,ENSOARG00000016610,ENSOARG00000007307,ENSOARG00000007322,ENSOARG00000001971,ENSOARG00000007252	8	5	OVAR-DRB1,OVAR-DRB1,OVAR-DRB1,OVAR-DRB5,OVAR-DRB3	13
**M29**	proinflammatory cytokines and chemokines	PTX3,CCRL2,IL6,CCL3,CCL3L1,CCL3L3,TNF,IL1A,C3,CCL20,IL1B,CXCL8	myeloid cells/inflammation	ENSOARG00000002754,ENSOARG00000014203,ENSOARG00000012021,ENSOARG00000008333,ENSOARG00000020877,ENSOARG00000020576,ENSOARG00000020866,ENSOARG00000014496	8	4	CCL3,CCL3L1,CCL3L3,C3	12
**M30**	cell movement, Adhesion & Platelet activation	PDGFA,ITGB3,PTK2,TNS1,SELP,MPP1,PROS1,KIF26A,TPM1,GAB1,NAV1,HBEGF,CTGF,COL5A1,ALOX12,ESAM,GP6,CALD1,CD9,SEMA6A	myeloid cells/inflammation	ENSOARG00000006153,ENSOARG00000003418,ENSOARG00000019446,ENSOARG00000010726,ENSOARG00000003150,ENSOARG00000017273,ENSOARG00000007197,ENSOARG00000020797,ENSOARG00000010978,ENSOARG00000018937,ENSOARG00000017521,ENSOARG00000014240,ENSOARG00000002129,ENSOARG00000009252,ENSOARG00000010670,ENSOARG00000002001,ENSOARG00000007819,ENSOARG00000008605,ENSOARG00000001863	19	1	ITGB3	20
**M31**	cell cycle and growth arrest	RARA,SIK1,GADD45B,JUNB,TNFAIP3,THBS1,PPP1R15A,OSM,IL1A,IL1B,DUSP1,CXCL8	cell cycle	ENSOARG00000014119,ENSOARG00000013671,ENSOARG00000010854,ENSOARG00000000569,ENSOARG00000020058,ENSOARG00000012344,ENSOARG00000006353,ENSOARG00000020877,ENSOARG00000020866,ENSOARG00000003963,ENSOARG00000014496	11	1	SIK1	12
**M32.0**	platelet activation (I)	TGFB1,PPP2R1A,CCDC22,AKT1,CORO1A,GNAI2,ZYX,H1FX,POR,TLN1,APEH,FAM108A1,NR1H2,ACAP1,GNB2,HGS,NUCB1,UBE2M,PPP1CA,ACTN4,PFN1,FLNA,NRBP1	myeloid cells/inflammation	ENSOARG00000007468,ENSOARG00000015049,ENSOARG00000010428,ENSOARG00000003604,ENSOARG00000010164,ENSOARG00000018034,ENSOARG00000014347,ENSOARG00000013448,ENSOARG00000011863,ENSOARG00000013387,ENSOARG00000013898,ENSOARG00000012323,ENSOARG00000016272,ENSOARG00000018265,ENSOARG00000012427, ENSOARG00000004012,ENSOARG00000011318,ENSOARG00000005791,ENSOARG00000005208,ENSOARG00000019315	20	3	AKT1,FAM108A1,PFN1	23
**M32.1**	platelet activation (II)	TGFB1,EHMT2,TLN1,ACTN4,PKN1,SIPA1,ACAP1,GNB2,AP2M1,AKT1,MAP7D1,MLF2,ZYX,NBEAL2,DNM2,FLNA,HGS,PFN1,GNAI2,MAP2K2,PNPLA6	coagulation	ENSOARG00000007468,ENSOARG00000003417,ENSOARG00000011863,ENSOARG00000005791,ENSOARG00000005558,ENSOARG00000017191,ENSOARG00000012323,ENSOARG00000016272,ENSOARG00000020615,ENSOARG00000019654,ENSOARG00000006618,ENSOARG00000018034,ENSOARG00000005857,ENSOARG00000017183,ENSOARG00000005208, ENSOARG00000018265,ENSOARG00000010164,ENSOARG00000010782,ENSOARG00000001448	19	2	AKT1,PFN1	21

**M32.2**	CORO1A-DEF6 network (I)	TGFB1,AES,FAM108A1,UBE2M,DEF6,H1FX,CCDC22,ACAP1,TECR,HGS,SIPA1,CORO1A,CLPTM1,TSC2,SH2B1,APEH,CBX4,STXBP2,BRD2,ATP5D	cell cycle	ENSOARG00000007468,ENSOARG00000013150,ENSOARG00000004012,ENSOARG00000011689,ENSOARG00000014347,ENSOARG00000010428,ENSOARG00000012323,ENSOARG00000005352,ENSOARG00000018265,ENSOARG00000017191,ENSOARG00000003604,ENSOARG00000009511,ENSOARG00000017283,ENSOARG00000002299,ENSOARG00000013387,ENSOARG00000002615,ENSOARG00000002236,ENSOARG00000008038,ENSOARG00000011366	19	1	FAM108A1	20
**M32.3**	KLF12 targets network	DGKZ,NRBP1,LTBP3,NR1H2,PPP2R1A,RHOG,POR,ACAP1,GNB2,CORO1A,CLPTM1,CNOT3,UBE2M,HGS,FAM108A1,GNAI2,NBEAL2	others	ENSOARG00000003287,ENSOARG00000019315,ENSOARG00000015227,ENSOARG00000013898,ENSOARG00000015049,ENSOARG00000013448,ENSOARG00000012323,ENSOARG00000016272,ENSOARG00000003604,ENSOARG00000009511,ENSOARG00000002981,ENSOARG00000004012,ENSOARG00000018265,ENSOARG00000010164,ENSOARG00000005857	15	2	RHOG,FAM108A1	17
**M32.4**	CORO1A-DEF6 network (II)	DGKZ,NRBP1,MAZ,UBE2M,DEF6,H1FX,ACAP1,GNB2,CORO1A,NR1H2,SH2B1,CNOT3,HGS,FAM108A1,BRD2	cell cycle	ENSOARG00000003287,ENSOARG00000019315,ENSOARG00000005520,ENSOARG00000004012,ENSOARG00000011689,ENSOARG00000014347,ENSOARG00000012323,ENSOARG00000016272,ENSOARG00000003604,ENSOARG00000013898,ENSOARG00000002299,ENSOARG00000002981,ENSOARG00000018265,ENSOARG00000008038	14	1	FAM108A1	15
**M32.5**	TBA	PPP2R1A,AES,MAZ,UBE2M,ASNA1,RALY,PKN1,ACIN1,AP2M1,MLF2,NRBP1,DNM2,PPP1R9B,NUCB1,HMG20B	others	ENSOARG00000015049,ENSOARG00000013150,ENSOARG00000005520,ENSOARG00000004012,ENSOARG00000011392,ENSOARG00000008957,ENSOARG00000005558,ENSOARG00000019368,ENSOARG00000020615,ENSOARG00000006618,ENSOARG00000019315,ENSOARG00000017183,ENSOARG00000005179,ENSOARG00000012427,ENSOARG00000012318	15	0		15
**M32.6**	TBA	PPP2R1A,MLF2,TGFB1,PKN1,CPNE1,HGS,PSMD3,CDC37,AP2M1,AKT1,DNM2,NUCB1,HMG20B	others	ENSOARG00000015049,ENSOARG00000006618,ENSOARG00000007468,ENSOARG00000005558,ENSOARG00000018265,ENSOARG00000012513,ENSOARG00000016156,ENSOARG00000020615,ENSOARG00000017183,ENSOARG00000012427,ENSOARG00000012318	11	2	CPNE1,AKT1	13
**M32.7**	TBA	AES,STK11,UBE2M,EIF4G1,H1FX,HGS,MAP2K2,MAP7D1,DCAF15,TSC2,BRD2	others	ENSOARG00000013150,ENSOARG00000011242,ENSOARG00000004012,ENSOARG00000020596,ENSOARG00000014347,ENSOARG00000018265,ENSOARG00000010782,ENSOARG00000019654,ENSOARG00000007462,ENSOARG00000017283,ENSOARG00000008038	11	0		11
**M32.8**	cytoskeletal remodeling	TGFB1,ATP2A3,SPTAN1,ACIN1,TLN1,AP2M1,AKT1,MAP7D1,FLNA,ZYX	others	ENSOARG00000007468,ENSOARG00000017976,ENSOARG00000009772,ENSOARG00000019368,ENSOARG00000011863,ENSOARG00000020615,ENSOARG00000019654,ENSOARG00000005208,ENSOARG00000018034	9	1	AKT1	10
**M33**	inflammatory response	TBC1D8,TNFSF13,FCER1G,STAB1,TYROBP,GPX1,CARD9,ARRB2,TLR4,APOB48R,AIF1	myeloid cells/inflammation	ENSOARG00000013258,ENSOARG00000014243,ENSOARG00000009800,ENSOARG00000003517,ENSOARG00000005167,ENSOARG00000004519,ENSOARG00000007797,ENSOARG00000005792,ENSOARG00000007932	9	2	GPX1,APOB48R	11
**M34**	cytoskeletal remodeling (enriched for SRF targets)	ACTN1,PPAP2B,VCL,MYLK,TPM1,NR2F2,CTGF,THBS1,CALD1,TPM2	others	ENSOARG00000021179,ENSOARG00000008576,ENSOARG00000020201,ENSOARG00000020797,ENSOARG00000010167,ENSOARG00000014240,ENSOARG00000020058,ENSOARG00000007819,ENSOARG00000011889	9	1	PPAP2B	10
**M35.0**	signaling in T cells (I)	IL2RA,JUNB,GZMB,FOS,IFNG,FOSB,EGR1,JUN,TNF,FASLG,PRF1,TNFRSF4,ATF3,TNFRSF9	T/NK cells	ENSOARG00000012860,ENSOARG00000010854,ENSOARG00000002990,ENSOARG00000001783,ENSOARG00000001958,ENSOARG00000009983,ENSOARG00000016470,ENSOARG00000009999,ENSOARG00000008333,ENSOARG00000014582,ENSOARG00000006139,ENSOARG00000006133,ENSOARG00000010493,ENSOARG00000010196	14	0		14
**M35.1**	signaling in T cells (II)	IL2RA,IL2RB,GZMB,IFNG,TNF,FOSL1,PRF1,TNFRSF4,FASLG,TNFRSF18,TNFRSF9	T/NK cells	ENSOARG00000012860,ENSOARG00000019197,ENSOARG00000002990,ENSOARG00000001958,ENSOARG00000008333,ENSOARG00000000072,ENSOARG00000006139,ENSOARG00000006133,ENSOARG00000014582,ENSOARG00000006215,ENSOARG00000010196, ENSOARG00000012860,ENSOARG00000019197,ENSOARG00000002990,ENSOARG00000001958,ENSOARG00000008333,ENSOARG00000000072,ENSOARG00000006139,ENSOARG00000006133,ENSOARG00000014582,ENSOARG00000006215,ENSOARG00000010196	11	0		11

**M36**	T cell surface, activation	CXADR,LILRB4,CD40LG,LILRB2,LILRB1,TYROBP,ITGA4,VCAM1,CD8A,LILRB3,CD3D,CD3E	T/NK cells	ENSOARG00000011076,ENSOARG00000005167,ENSOARG00000016867,ENSOARG00000020734,ENSOARG00000009172,ENSOARG00000008984	6	6	CXADR,LILRB4,LILRB2,LILRB1,VCAM1,LILRB3	12
**M37.0**	immune activation - generic cluster	ELANE,SAMD13,CAMK4,GPR109B,BANK1,DYSF,MACROD2,MS4A4A,PRRG4,ORM1,C19orf59,ERI2,LAT,PPIL4,CSRP2,MAL,CD1B,RASGRP4,MGST1,PRG2,VPREB1,VPREB3,ENG,GRAP2,NCAPD2,NCAPD3,BLM,TCN1,SLC22A4,ANPEP,SIRPB1,CD19,RETN,MBOAT2,CLEC5A,PLA2G7,CIITA,PTX3,SMC4,P2RY13,S100A8,RNF150,KIAA0513,C10orf10,MANSC1,LTF,PRAM1,SLC27A2,ACPP,F5,HMGB2,CHAF1A,CHAF1B,HLA-DRA,SULF2,CDK14,CXCR2,KLF5,DEFA4,DEFA1,JAG1,FPR2,SLC24A4,CTSG,TCL1A,C3AR1,NUP37,CA4,OGN,CMTM2,LILRA5,LILRA3,LIN7A,HNRPLL,DKFZp451A211,MARCH1,CHI3L2,CHI3L1,CYP4F2,CYP4F3,B3GNT5,ORC6L,ROPN1L,CRISPLD2,CENPL,CRISP3,BARD1,FAM69B,CCDC150,RBBP8,LY96,ACCN2,GH1,AZU1,DBF4,CD300C,LOC90925,EMR1,VANGL1,ARHGEF10L,OSCAR,MGAM,CCNE1,CEACAM1,CEACAM6,TACSTD2,BMX,PLXDC2,G0S2,JAM3,VNN2,VNN1,IGFBP7,BLVRB,GALNT6,STK32B,C15orf23,MEF2C,DTYMK,GPR97,BTK,CD72,CXorf50B,RFC2,PRKAR2B,TREM1,MCTP1,GAS2L1,NLR [illegible]	others	ENSOARG00000009696,ENSOARG00000013828,ENSOARG00000000184,ENSOARG00000013398,ENSOARG00000011500,ENSOARG00000016840,ENSOARG00000013256,ENSOARG00000001158,ENSOARG00000002675,ENSOARG00000014916,ENSOARG00000007282,ENSOARG00000005647,ENSOARG00000020582,ENSOARG00000012733,ENSOARG00000011387,ENSOARG00000017108,ENSOARG00000008036,ENSOARG00000013282,ENSOARG00000012875,ENSOARG00000012438,ENSOARG00000015213,ENSOARG00000011841,ENSOARG00000001710,ENSOARG00000002291,ENSOARG00000014789,ENSOARG00000014270,ENSOARG00000011007,ENSOARG00000006106,ENSOARG00000002754,ENSOARG00000002047,ENSOARG00000004209,ENSOARG00000000450,ENSOARG00000012236,ENSOARG00000011232,ENSOARG00000003708,ENSOARG00000020865,ENSOARG00000008620,ENSOARG00000004053,ENSOARG00000020994,ENSOARG00000009106,ENSOARG00000010135,ENSOARG00000015382,ENSOARG00000009704,ENSOARG00000013992,ENSOARG00000016610,ENSOARG00000010080,ENSOARG00000016384,ENSOARG00000019477,ENSOARG00000015505,ENSOARG00000010523,ENSOARG00000012969,ENSOARG00000015981,ENSOARG00000020341,ENSOARG00000015613,ENSOARG00000017880,ENSOARG00000008277,ENSOARG00000002005,ENSOARG00000001224,ENSOARG00000015171,ENSOARG00000008915,ENSOARG00000013445,ENSOARG00000019517,ENSOARG00000001370,ENSOARG00000002100,ENSOARG00000020655,ENSOARG00000013987,ENSOARG [illegible]	277	70	GPR109B,MACROD2,MS4A4A,ORM1,C19orf59,MAL,PRG2,VPREB1,SIRPB1,DEFA4,DEFA1,FPR2,CTSG,LILRA3,DKFZp451A211,CYP4F2,ORC6L,RBBP8,ACCN2,CD300C,LOC90925,EMR1,ARHGEF10L,OSCAR,CEACAM1,CEACAM6,BMX,C15orf23,GPR97,CXorf50B,ACPL2,LOC81691,CLC,LOC284757,BASP1,LCN2,VSTM1,C13orf34,CAMP,RGL4,LOC100130458,FCGR2C,MGC5566,EBF1,MOSC1,AMICA1,LOC643332,TXNDC3,CR1,PROK2,LOC10028 [illegible]	347
**M37.1**	enriched in neutrophils (I)	LIN7A,NCF4,EMR3,FCGR3A,S100P,FCGR3B,TNFRSF10C,C5AR1,MGC31957,CHI3L1,NLRP12,CYP4F3,MXD1,SEPX1,MGAM,DGAT2,RGL4,REPS2,VNN2,CXCR1,NFE2,KRT23,GPR109B,PYGL,FPR2,G0S2,KCNJ15,LRRC4,FPR1,CREB5,FCGR2A,CMTM2,MANSC1,CSF3R,FFAR2,RNF24,LRG1,ST6GALNAC2,ORM1,MME,CDA,PROK2,PFKFB4,VNN3,SLC22A4,BASP1,TREM1,GLT1D1,GPR97,PTAFR,STEAP4,ALPL	inflammation	ENSOARG00000015171,ENSOARG00000019050,ENSOARG00000010070,ENSOARG00000017294,ENSOARG00000011005,ENSOARG00000001370,ENSOARG00000000492,ENSOARG00000002100,ENSOARG00000019742,ENSOARG00000014509,ENSOARG00000012189,ENSOARG00000013320,ENSOARG00000014486,ENSOARG00000019492,ENSOARG00000016228,ENSOARG00000015540,ENSOARG00000020686,ENSOARG00000004314,ENSOARG00000014447,ENSOARG00000002353,ENSOARG00000009058,ENSOARG00000002005,ENSOARG00000020865,ENSOARG00000019729,ENSOARG00000011266,ENSOARG00000000500,ENSOARG00000009290,ENSOARG00000006862,ENSOARG00000003370,ENSOARG00000009205,ENSOARG00000017897,ENSOARG00000015213,ENSOARG00000001869,ENSOARG00000016604,ENSOARG00000019840,ENSOARG00000011811,ENSOARG00000008507, ENSOARG00000015171,ENSOARG00000019050,ENSOARG00000010070,ENSOARG00000017294,ENSOARG00000011005,ENSOARG00000001370,ENSOARG00000000492,ENSOARG00000002100,ENSOARG00000019742,ENSOARG00000014509,ENSOARG00000012189,ENSOARG00000013320,ENSOARG00000014486,ENSOARG00000019492,ENSOARG00000016228,ENSOARG00000015540,ENSOARG00000020686,ENSOARG00000004314,ENSOARG00000014447,ENSOARG00000002353,ENSOARG00000009058,ENSOARG00000002005,ENSOARG00000020865,ENSOARG00000019729,ENSOARG00000011266,ENSOARG00000000500,ENSOARG00000009290,ENSOARG000000068 [illegible]	37	15	EMR3,S100P,FCGR3B,MGC31957,SEPX1,RGL4,GPR109B,FPR2,FPR1,FCGR2A,ORM1,PROK2,VNN3,BASP1,GPR97	52
**M37.2**	endoplasmic reticulum	TMED6,WFS1,TBXAS1,SGK1,SCD,UNC93B1,ACSL1,CD4,FAM69B,ELOVL4,MGST1,LPCAT2,TLR7,SORT1,MARCH1,CYP4F2,CYP4F3,TLR8,DHCR24	DC/antigen presentation	ENSOARG00000003552,ENSOARG00000005787,ENSOARG00000011915,ENSOARG00000014710,ENSOARG00000014674,ENSOARG00000014140,ENSOARG00000007614,ENSOARG00000006301,ENSOARG00000003746,ENSOARG00000007109,ENSOARG00000020582,ENSOARG00000018091,ENSOARG00000011288,ENSOARG00000019184,ENSOARG00000013445,ENSOARG00000002100,ENSOARG00000018099,ENSOARG00000007493	18	1	CYP4F2	19

**M37.3**	cell division	SAC3D1,RBBP8,RUVBL1,CCNE1,NUP37,NCAPD3,BLM,NCAPD2,CKAP2,VRK1	cell cycle	ENSOARG00000011710,ENSOARG00000004977,ENSOARG00000004067,ENSOARG00000015613,ENSOARG00000013282,ENSOARG00000012875,ENSOARG00000008036,ENSOARG00000009289,ENSOARG00000000106	9	1	RBBP8	10
**M38**	chemokines and receptors	CCL2,CCL4,CCL5,DOCK2,TNF,CXCR6,ICAM1,CXCR4,HCK,CXCL8,CCR5	inflammation	ENSOARG00000009627,ENSOARG00000004253,ENSOARG00000004964,ENSOARG00000003028,ENSOARG00000008333,ENSOARG00000014277,ENSOARG00000015656,ENSOARG00000010462,ENSOARG00000002820,ENSOARG00000014496,ENSOARG00000014214	11	0		11
**M39**	integrin mediated leukocyte migration	ITGB2,TXK,ITK,VAV1,PIK3CD,PRKCB,ITGA4,PLCG1,ITGAL,VCAM1	myeloid cells/inflammation	ENSOARG00000012367,ENSOARG00000016822,ENSOARG00000011171,ENSOARG00000004983,ENSOARG00000007664,ENSOARG00000017631,ENSOARG00000016867,ENSOARG00000001700,ENSOARG00000006423	9	1	VCAM1	10
**M40**	complement and other receptors in DCs	ITGAX,SIRPA,OLR1,TYROBP,CD58,C1QC,C1QB,C1QA,CD86,LY96,TLR8,DQA,OVAR-DQA1,OVAR-DQA2,OVAR-DQB1,OVAR-DQB2	DC/antigen presentation	ENSOARG00000009130,ENSOARG00000007425,ENSOARG00000021012,ENSOARG00000005167,ENSOARG00000020234,ENSOARG00000007991,ENSOARG00000007977,ENSOARG00000008001,ENSOARG00000020112,ENSOARG00000006376,ENSOARG00000018099,ENSOARG00000015485, ENSOARG00000009130,ENSOARG00000007425,ENSOARG00000021012,ENSOARG00000005167,ENSOARG00000020234,ENSOARG00000007991,ENSOARG00000007977,ENSOARG00000008001,ENSOARG00000020112,ENSOARG00000006376,ENSOARG00000018099,ENSOARG00000015485	12	4	OVAR-DQA1,OVAR-DQA2,OVAR-DQB1,OVAR-DQB2	16
**M41.0**	TBA	MAGEA12,SLC7A11,OBSL1,PLUNC,TUBB4,CA14,SPRY4,AFF2,SLC17A1,HOXC11,HOXC10,DEFA5,CEACAM7,HIST1H3E,REEP1,PEG3,IFN-ALPHA	others	ENSOARG00000014253,ENSOARG00000020239,ENSOARG00000020773,ENSOARG00000009528,ENSOARG00000002375,ENSOARG00000016342,ENSOARG00000016331,ENSOARG00000009204,ENSOARG00000020650,ENSOARG00000003426	10	7	MAGEA12,PLUNC,TUBB4,SPRY4,DEFA5,CEACAM7,IFN-ALPHA	17
**M41.1**	TBA	MAGEA12,DMD,PRO2012,ERBB4,SPINLW1,DAAM2,SLC17A1,HOXC10,DEFA5,PEG3,ADCYAP1,MTMR8,CYP11B1,IFN-ALPHA,ATP7B	others	ENSOARG00000018256,ENSOARG00000019232,ENSOARG00000000237,ENSOARG00000002375,ENSOARG00000016331,ENSOARG00000003426,ENSOARG00000009539,ENSOARG00000000864,ENSOARG00000009056	9	6	MAGEA12,PRO2012,SPINLW1,DEFA5,MTMR8,IFN-ALPHA	15
**M41.2**	TBA	MAGEA12,OBSL1,ADCYAP1,ERBB4,GRID2,TUBB4,SLC7A11,POU4F2,SLC17A1,DEFA5,GFRA3,MTMR8,PEG3,IFN-ALPHA	others	ENSOARG00000020239,ENSOARG00000009539,ENSOARG00000019232,ENSOARG00000018241,ENSOARG00000014253,ENSOARG00000002375,ENSOARG00000016086,ENSOARG00000003426	8	6	MAGEA12,TUBB4,POU4F2,DEFA5,MTMR8,IFN-ALPHA	14
**M41.3**	TBA	EPHB1,DMD,PRO2012,ERBB4,TFAP2A,HOXC10,DEFA5,NPR3,CYP11B1,PEG3	others	ENSOARG00000008094,ENSOARG00000018256,ENSOARG00000019232,ENSOARG00000016395,ENSOARG00000016331,ENSOARG0000012147,ENSOARG00000000864,ENSOARG00000003426	8	2	PRO2012,DEFA5	10
**M41.4**	ATF targets network	SULT4A1,HOXC10,DAAM2,SPINLW1,FABP7,ADCYAP1,HIST1H3E,REEP1,PEG3,ATP7B	others	ENSOARG00000019215,ENSOARG00000016331,ENSOARG00000000237,ENSOARG00000008181,ENSOARG00000009539,ENSOARG00000009204,ENSOARG00000020650,ENSOARG00000003426,ENSOARG00000009056	9	1	SPINLW1	10
**M42**	platelet activation (III)	FCER1G,GNAQ,SYK,VAV1,PLCG2,PIK3R5,GNA15,COL1A1,LCP2,LYN	coagulation	ENSOARG00000009800,ENSOARG00000012393,ENSOARG00000007814,ENSOARG00000004983,ENSOARG00000008988,ENSOARG00000002479,ENSOARG00000012997,ENSOARG00000004871,ENSOARG00000003167,ENSOARG00000014778	10	0		10
**M43.0**	myeloid, dendritic cell activation via NFkB (I)	MAP3K8,IL23A,CCL5,CD83,NFKBID,FUT7,CTGF,TNF,VCAM1,ICAM1,SAMSN1,RELB,NFKB2,EBI3,BCL3	DC/antigen presentation	ENSOARG00000015050,ENSOARG00000009246,ENSOARG00000004964,ENSOARG00000012936,ENSOARG00000005154,ENSOARG00000014240,ENSOARG00000008333,ENSOARG00000015656,ENSOARG00000016184,ENSOARG00000009545,ENSOARG00000000483,ENSOARG00000010445,ENSOARG00000009239	13	2	FUT7,VCAM1	15
**M43.1**	myeloid, dendritic cell activation via NFkB (II)	IL1RN,IL23A,CCL5,NFKBIA,BIRC3,PRKCD,FUT7,LTB,TNIP3,ICAM1,RELB,NFKB2,EBI3,BCL3	DC/antigen presentation	ENSOARG00000020828,ENSOARG00000009246,ENSOARG00000004964,ENSOARG00000007502,ENSOARG00000006356,ENSOARG00000000156,ENSOARG00000008259,ENSOARG00000015256,ENSOARG00000015656,ENSOARG00000009545,ENSOARG00000000483,ENSOARG00000010445,ENSOARG00000009239, ENSOARG00000020828,ENSOARG00000009246,ENSOARG00000004964,ENSOARG00000007502,ENSOARG00000006356,ENSOARG00000000156,ENSOARG00000008259,ENSOARG00000015256,ENSOARG00000015656,ENSOARG00000009545,ENSOARG00000000483,ENSOARG00000010445,ENSOARG00000009239	13	1	FUT7	14

**M44**	T cell signaling and costimulation	IL7R,TCF7,IL23A,FYN,IFI30,CD37,MAPK14,ARHGDIB,LCP1,LCK	T/NK cells	ENSOARG00000011239,ENSOARG00000013765,ENSOARG00000009246,ENSOARG00000009732,ENSOARG00000013291,ENSOARG00000012851,ENSOARG00000012870,ENSOARG00000020698,ENSOARG00000007864,ENSOARG00000020968	10	0		10
**M45**	leukocyte activation and migration	SIRPG,FCER1G,INPP5D,CD48,FYN,ITGA4,SELPLG,PTPN6,ITGAL,CD2,LCK	myeloid cells/inflammation	ENSOARG00000009800,ENSOARG00000019324,ENSOARG00000009732,ENSOARG00000016867,ENSOARG00000005032,ENSOARG00000006423,ENSOARG00000020249,ENSOARG00000020968	8	3	SIRPG,CD48,SELPLG	11
**M46**	cell division stimulated CD4+ T cells	DHCR24,DTL,ZWINT,RRM2,KIAA0101,TST,TYMS,CDT1,KIF4A,CDCA3,CDCA7,MYB,GGH,TOP2A,CDK6,GNB4,LOC81691,UHRF1,SLC27A2,ACTN1,GINS1,ZNRF1,SHCBP1,APOBEC3B,CDK1,RAD51,CDC45,KIF20A	B cells	ENSOARG00000007493,ENSOARG00000010843,ENSOARG00000013084,ENSOARG00000015333,ENSOARG00000019145,ENSOARG00000009367,ENSOARG00000013512,ENSOARG00000015491,ENSOARG00000005652,ENSOARG00000000578,ENSOARG00000014901,ENSOARG00000016793,ENSOARG00000014321,ENSOARG00000018572,ENSOARG00000020697,ENSOARG00000008530,ENSOARG00000020994,ENSOARG00000021179,ENSOARG00000007717,ENSOARG00000006385,ENSOARG00000015392,ENSOARG00000004318,ENSOARG00000020254,ENSOARG00000017745,ENSOARG00000015873	25	3	KIAA0101,LOC81691,APOBEC3B	28
**M47.0**	enriched in B cells (I)	STAP1,BLK,CD72,TSPAN13,BCL11A,C13orf18,KIAA0125,CD200,CD79B,VPREB3,CD79A,P2RX5,BTLA,AFF3,PPAPDC1B,FCRLA,SPIB,FAM129C,ABCB4,FCRL1,BANK1,MS4A1,FCRL5,TCF4,KLHL14,CXCR5,PCDH9,RALGPS2,CD22,TCL1A,CD24,MACROD2,EBF1,PNOC,ADAM28,CR2,PAX5,E2F5,PLEKHG1,FCER2,PKIG,FCRL2,HLA-DOB,PTPRK,CD19,IGHM,IGHD,IGHA1,IGHE, IGHG,IGHG	B cells	ENSOARG00000007050,ENSOARG00000015215,ENSOARG00000011957,ENSOARG00000008934,ENSOARG00000001663,ENSOARG00000019298,ENSOARG00000014015,ENSOARG00000012733,ENSOARG00000008358,ENSOARG00000017481,ENSOARG00000019308,ENSOARG00000010166,ENSOARG00000016242,ENSOARG00000006960,ENSOARG00000013398,ENSOARG00000013081,ENSOARG00000006828,ENSOARG00000005018,ENSOARG00000006008,ENSOARG00000011654,ENSOARG00000014957,ENSOARG00000016177,ENSOARG00000004796,ENSOARG00000015981,ENSOARG00000011081,ENSOARG00000014922,ENSOARG00000010026,ENSOARG00000008208,ENSOARG00000011518,ENSOARG00000013715,ENSOARG00000003012,ENSOARG00000002445,ENSOARG00000004128,ENSOARG00000007086,ENSOARG00000007458,ENSOARG00000013550,ENSOARG00000001710,ENSOARG00000002846,ENSOARG00000009269,ENSOARG00000008862,ENSOARG00000008994, ENSOARG00000007050,ENSOARG00000015215,ENSOARG00000011957,ENSOARG00000008934,ENSOARG00000001663,ENSOARG00000019298,ENSOARG00000014015,ENSOARG00000012733,ENSOARG00000008358,ENSOARG00000017481,ENSOARG00000019308,ENSOARG00000010166,ENSOARG00000016242,ENSOARG00000006960,ENSOARG00000013398,ENSOARG00000013081,ENSOARG00000006828,ENSOARG00000005018,ENSOARG00000006008,ENSOARG00000011654,ENSOARG00000014957,ENSOARG00000016177,ENSOARG00000004796,ENSOARG00000015981,ENSOARG00000011081,ENSOARG000000	41	10	C13orf18,KIAA0125,AFF3,PPAPDC1B,SPIB,ABCB4,MACROD2,EBF1,IGHG,IGHG	51

**M47.1**	enriched in B cells (II)	BCL11A,KIAA0125,CD79B,CD79A,TPD52,CDK14,CXCR5,BANK1,MS4A1,FCRL5,BLNK,TCF4,SP140,KLHL14,RALGPS2,FCRLA,FCGR2B,TCL1A,CELSR1,CD24,DENND5B,EBF1,PNOC,PAWR,CD180,CR2,PAX5,PLEKHG1,POU2AF1,PKIG,FAM30A,HLA-DOB,KLF8,PTPRK,CD19,IGKV2D-26,IGKV,IGHM,IGHD,IGHA1,IGHE,IGHG	B cells	ENSOARG00000001663,ENSOARG00000014015,ENSOARG00000008358,ENSOARG00000008295,ENSOARG00000016384,ENSOARG00000011654,ENSOARG00000013398,ENSOARG00000013081,ENSOARG00000006828,ENSOARG00000007320,ENSOARG00000005018,ENSOARG00000020646,ENSOARG00000006008,ENSOARG00000016177,ENSOARG00000010166,ENSOARG00000010094,ENSOARG00000015981,ENSOARG00000019502,ENSOARG00000011081,ENSOARG00000019444,ENSOARG00000014922,ENSOARG00000015037,ENSOARG00000005907,ENSOARG00000008208,ENSOARG00000011518,ENSOARG00000003012,ENSOARG00000013870,ENSOARG00000004128,ENSOARG00000007458,ENSOARG00000005543,ENSOARG00000013550,ENSOARG00000001710,ENSOARG00000020807,ENSOARG00000002846,ENSOARG00000009269,ENSOARG00000008862,ENSOARG00000008994, ENSOARG00000001663,ENSOARG00000014015,ENSOARG00000008358,ENSOARG00000008295,ENSOARG00000016384,ENSOARG00000011654,ENSOARG00000013398,ENSOARG00000013081,ENSOARG00000006828,ENSOARG00000007320,ENSOARG00000005018,ENSOARG00000020646,ENSOARG00000006008,ENSOARG00000016177,ENSOARG00000010166,ENSOARG00000010094,ENSOARG00000015981,ENSOARG00000019502,ENSOARG00000011081,ENSOARG00000019444,ENSOARG00000014922,ENSOARG00000015037,ENSOARG00000005907,ENSOARG00000008208,ENSOARG00000011518,ENSOARG00000003012,ENSOARG00000013870,ENSOARG00000004128,ENSOARG00000007458,ENSOARG00000005543	37	5	KIAA0125,EBF1,FAM30A,IGKV,IGHG	42
**M47.2**	enriched in B cells (III)	KIAA0125,VPREB3,MEF2C,TPD52,CDK14,FCRL3,FCRL5,HHEX,TBC1D9,FCRLA,ALOX5,FCGR2B,CELSR1,CD24,CD69,PNOC,PAWR,CD180,FAIM3,FAM30A,HLA-DOA,DQA,BTK,DENND5B,IGHA1,IGKV2D-26,IGKV,IGHM,IGHD,IGHA1,IGHE,IGHG-,IGLV	B cells	ENSOARG00000012733,ENSOARG00000016099,ENSOARG00000008295,ENSOARG00000016384,ENSOARG00000006866,ENSOARG00000006828,ENSOARG00000012380,ENSOARG00000010166,ENSOARG00000002819,ENSOARG00000010094,ENSOARG00000019502,ENSOARG00000011081,ENSOARG00000021063,ENSOARG00000014922,ENSOARG00000015037,ENSOARG00000005907,ENSOARG00000008091,ENSOARG00000015485,ENSOARG00000001620,ENSOARG00000019444,ENSOARG00000008862,ENSOARG00000020807,ENSOARG00000002846,ENSOARG00000009269,ENSOARG00000008862,ENSOARG00000008994, ENSOARG00000012733,ENSOARG00000016099,ENSOARG00000008295,ENSOARG00000016384,ENSOARG00000006866,ENSOARG00000006828,ENSOARG00000012380,ENSOARG00000010166,ENSOARG00000002819,ENSOARG00000010094,ENSOARG00000019502,ENSOARG00000011081,ENSOARG00000021063,ENSOARG00000014922,ENSOARG00000015037,ENSOARG00000005907,ENSOARG00000008091,ENSOARG00000015485,ENSOARG00000001620,ENSOARG00000019444,ENSOARG00000008862,ENSOARG00000020807,ENSOARG00000002846,ENSOARG00000009269,ENSOARG00000008862,ENSOARG00000008994	26	7	KIAA0125,HHEX,FAIM3,FAM30A,IGKV,IGHG-,IGLV	33
**M47.3**	enriched in B cells (IV)	FAIM3,CPNE5,TPD52,CIITA,IGHA1,TLR10,IGHG,CELSR1,FAM30A,CDK14,PAWR,CD180,MEF2C,BTK,HLA-DOA	B cells	ENSOARG00000013791,ENSOARG00000008295,ENSOARG00000006106,ENSOARG00000008862,ENSOARG00000000520,ENSOARG00000019502,ENSOARG00000016384,ENSOARG00000015037,ENSOARG00000005907,ENSOARG00000016099,ENSOARG00000001620,ENSOARG00000008091	12	3	FAIM3,IGHG,FAM30A	15
**M47.4**	enriched in B cells (V)	GPR18,FAIM3,FCRL3,FCGR2B,LY9,CELSR1,CD37,TLR10,CD69,CD180,FCRL5	B cells	ENSOARG00000017207,ENSOARG00000006866,ENSOARG00000010094,ENSOARG00000008981,ENSOARG00000019502,ENSOARG00000012851,ENSOARG00000000520,ENSOARG00000021063,ENSOARG00000005907,ENSOARG00000006828	10	1	FAIM3	11
**M48**	TBA	TNFSF13,PLCB2,HHEX,STAB1,TYROBP,SH3TC1,KCNQ1,DOK2,SPI1,PTAFR,TLR4,APOB48R,LYN	others	ENSOARG00000014243,ENSOARG00000020162,ENSOARG00000003517,ENSOARG00000005167,ENSOARG00000012470,ENSOARG00000002794,ENSOARG00000010631,ENSOARG00000006553,ENSOARG00000019840,ENSOARG00000005792,ENSOARG00000014778	11	2	HHEX,APOB48R	13

**M49**	transcription regulation in cell development	MYH10,STMN1,TIMP2,HMGB2,MSH2,SPTB,EPB42,SPTA1,PLCG1,FBXO5,HBZ,SEMA4A,GSN,PLXNB2,CEBPA,TYMS,ECT2,ANK1,TAL1,ASPM,KIF4A,FHL2,KLF4,KLF1,CDH1,LYN,BRCA1,ARHGEF12,SOD2,VCAN,RXRA,FES,CASC5,ACHE,TRIP13,C11orf82,GATA1,CCNB1,LILRB1,LST1,SDC2,TMOD1,CDC20,CDK1,BASP1,TYROBP,PAK1	cell cycle	ENSOARG00000001617,ENSOARG00000012293,ENSOARG00000003264,ENSOARG00000015382,ENSOARG00000005136,ENSOARG00000021154,ENSOARG00000020591,ENSOARG00000007488,ENSOARG00000001700,ENSOARG00000003968,ENSOARG00000010888,ENSOARG00000005579,ENSOARG00000005473,ENSOARG00000019806,ENSOARG00000004414,ENSOARG00000009367,ENSOARG00000020736,ENSOARG00000002688,ENSOARG00000003450,ENSOARG00000014713,ENSOARG00000015491,ENSOARG00000012948,ENSOARG00000007169,ENSOARG00000010245,ENSOARG00000003455,ENSOARG00000014778,ENSOARG00000004835,ENSOARG00000017361,ENSOARG00000015632,ENSOARG00000002366,ENSOARG00000012648,ENSOARG00000020247,ENSOARG00000015526,ENSOARG00000016070,ENSOARG00000012017,ENSOARG00000012913,ENSOARG00000008167,ENSOARG00000004813,ENSOARG00000011271,ENSOARG00000020542,ENSOARG00000004318,ENSOARG00000005167,ENSOARG00000007238	43	4	SOD2,C11orf82,LILRB1,BASP1	47
**M50**	CD1 and other DC receptors	CD1E,CD1A,CD1B,CD1D,CSF1R,IL1R2,CSF2RA,ANPEP,CD9,IL1R1	DC/antigen presentation	ENSOARG00000007307,ENSOARG00000007267,ENSOARG00000007282,ENSOARG00000007252,ENSOARG00000006358,ENSOARG00000013159,ENSOARG00000007541,ENSOARG00000011841,ENSOARG00000008605,ENSOARG00000013144, ENSOARG00000007307,ENSOARG00000007322,ENSOARG00000007291,ENSOARG00000007252,ENSOARG00000006358,ENSOARG00000013159,ENSOARG00000007541,ENSOARG00000011841,ENSOARG00000008605,ENSOARG00000013144, ENSOARG00000007307,ENSOARG00000007267,ENSOARG00000001986,ENSOARG00000007252,ENSOARG00000006358,ENSOARG00000013159,ENSOARG00000007541,ENSOARG00000011841,ENSOARG00000008605,ENSOARG00000013144, ENSOARG00000007307,ENSOARG00000007322,ENSOARG00000001657,ENSOARG00000007252,ENSOARG00000006358,ENSOARG00000013159,ENSOARG00000007541,ENSOARG00000011841,ENSOARG00000008605,ENSOARG00000013144, ENSOARG00000007307,ENSOARG00000007267,ENSOARG00000002532,ENSOARG00000007252,ENSOARG00000006358,ENSOARG00000013159,ENSOARG00000007541,ENSOARG00000011841,ENSOARG00000008605,ENSOARG00000013144, ENSOARG00000007307,ENSOARG00000007322,ENSOARG00000002614,ENSOARG00000007252,ENSOARG00000006358,ENSOARG00000013159,ENSOARG00000007541,ENSOARG00000011841,ENSOARG00000008605,ENSOARG00000013144, ENSOARG00000007307,ENSOARG00000007267,ENS[illegible]	10	0		10
**M51**	cell adhesion	CDH11,THBS2,LAMB1,ALOX12,COL3A1,CHL1,LAMA4,ARHGAP6,IL1B,ITGA2B,MMRN1,JAM3,GP1BA,TPM1,THBS1,NID2,GP9,PARVB,ITGB3,ITGB5,ENG,POSTN,COL5A1,VCAM1,TNC,SNAI2,CXCL12,SLAMF7,FN1,CCDC80,SRPX,MGP,ESAM,COL1A1,COL6A1,VWF,SELP,IL8	others	ENSOARG00000001829,ENSOARG00000005037,ENSOARG00000007228,ENSOARG00000009252,ENSOARG00000016476,ENSOARG00000009067,ENSOARG00000009580,ENSOARG00000010821,ENSOARG00000020866,ENSOARG00000018398,ENSOARG00000013611,ENSOARG00000020797,ENSOARG00000020058,ENSOARG00000020710,ENSOARG00000019265,ENSOARG00000020222,ENSOARG00000011387,ENSOARG00000010041,ENSOARG00000002129,ENSOARG00000005941,ENSOARG00000013030,ENSOARG00000002991,ENSOARG00000008967,ENSOARG00000019329,ENSOARG00000019332,ENSOARG00000000486,ENSOARG00000020724,ENSOARG00000010670,ENSOARG00000004871,ENSOARG00000012810,ENSOARG00000008752,ENSOARG00000010726,ENSOARG00000014496	33	5	ITGA2B,GP1BA,GP9,ITGB3,VCAM1	38
**M52**	T cell activation (IV)	SELL,SPI1,GFI1,LPXN,XCL1,GATA3,LAG3,CD5,PTPN6,PTPRC,UNC93B1,LCK,CD3E	T/NK cells	ENSOARG00000010897,ENSOARG00000006553,ENSOARG00000016329,ENSOARG00000011101,ENSOARG00000009179,ENSOARG00000013941,ENSOARG00000006528,ENSOARG00000016138,ENSOARG00000005032,ENSOARG00000015718,ENSOARG00000014140,ENSOARG00000020968,ENSOARG00000008984	13	0		13

**M53**	inflammasome receptors and signaling	NLRP3,NFKBIA,BIRC3,BIRC2,TNFAIP3,RXRA,CARD9,RIPK2,TLR4,NOD2,NLRC4,PAK1	inflammation	ENSOARG00000007591,ENSOARG00000007502,ENSOARG00000006356,ENSOARG00000006491,ENSOARG00000000569,ENSOARG00000002366,ENSOARG00000004519,ENSOARG00000011197,ENSOARG00000005792,ENSOARG00000017441,ENSOARG00000010638,ENSOARG00000007238, ENSOARG00000007591,ENSOARG00000007502,ENSOARG00000006356,ENSOARG00000006491,ENSOARG00000000569,ENSOARG00000002366,ENSOARG00000004519,ENSOARG00000011197,ENSOARG00000005792,ENSOARG00000017441,ENSOARG00000010638,ENSOARG00000007238	12	0		12
**M54**	BCR signaling	INPP5D,SYK,PIK3CD,VAV1,PLCG2,PIK3R5,PIK3R3,RAC2,LYN,PTPN6,BTK,BLNK	B cells	ENSOARG00000019324,ENSOARG00000007814,ENSOARG00000007664,ENSOARG00000004983,ENSOARG00000008988,ENSOARG00000002479,ENSOARG00000002180,ENSOARG00000014778,ENSOARG00000005032,ENSOARG00000001620,ENSOARG00000007320	11	1	RAC2	12
**M55**	TBA	RARA,LRP1,RASGRP4,SPI1,GRINA,PRKCD,DOK3,PDLIM7,MBOAT7,CSF3R,RHOG,ZYX	others	ENSOARG00000014119,ENSOARG00000007332,ENSOARG00000005647,ENSOARG00000006553,ENSOARG00000015553,ENSOARG00000000156,ENSOARG00000004300,ENSOARG00000005380,ENSOARG00000002806,ENSOARG00000019729,ENSOARG00000018034	11	1	RHOG	12
**M56**	suppression of MAPK signaling	HSPA6,RASGRP4,FOS,CD14,ARRB2,ARRB1,CACNA2D3,IL1B,DUSP6,DUSP1,IL1R2,DUSP3	others	ENSOARG00000007452,ENSOARG00000005647,ENSOARG00000001783,ENSOARG00000025180,ENSOARG00000007797,ENSOARG00000011342,ENSOARG00000015530,ENSOARG00000020866,ENSOARG00000015628,ENSOARG00000003963,ENSOARG00000013159,ENSOARG00000005929	12	0		12
**M57**	immuregulation - monocytes, T and B cells	CTLA4,IL23A,CCL4,FCER2,CAMK2D,CSF3,OSM,IGHD,BLK,CD200,IL1A,KIT,CD79A	B cells	ENSOARG00000018321,ENSOARG00000009246,ENSOARG00000004253,ENSOARG00000002445,ENSOARG00000018416,ENSOARG00000012606,ENSOARG00000006353,ENSOARG00000009269,ENSOARG00000015215,ENSOARG00000019298,ENSOARG00000020877,ENSOARG00000000078,ENSOARG00000008358, ENSOARG00000018321,ENSOARG00000009246,ENSOARG00000004253,ENSOARG00000002445,ENSOARG00000018416,ENSOARG00000012606,ENSOARG00000006353,ENSOARG00000009269,ENSOARG00000015215,ENSOARG00000019298,ENSOARG00000020877,ENSOARG00000000078,ENSOARG00000008358	13	0		13
**M58**	B cell development/activation	TCF4,JAM3,NEDD4,GAB2,PIK3CD,SPIB,BCL2,LYN,DUSP6,BTK,BLNK	B cells	ENSOARG00000005018,ENSOARG00000013611,ENSOARG00000020889,ENSOARG00000007664,ENSOARG00000006262,ENSOARG00000014778,ENSOARG00000015628,ENSOARG00000001620,ENSOARG00000007320	9	2	GAB2,SPIB	11
**M59**	CCR1, 7 and cell signaling	DUSP6,ZNF423,TIMP2,SOCS2,MAPK14,CCR1,CISH,CCR7,BCL6,TRIB1	inflammation	ENSOARG00000015628,ENSOARG00000017044,ENSOARG00000003264,ENSOARG00000015864,ENSOARG00000012870,ENSOARG00000014260,ENSOARG00000008693,ENSOARG00000014777,ENSOARG00000020487,ENSOARG00000008183	10	0		10
**M60**	lymphocyte generic cluster	MEF2C,CD28,DTX3,GAB1,SH2D2A,ADARB1,LARGE,FAM69B,CTGF,PRKCZ,CHI3L2,CD2,CLIP4,BANK1,DHCR24,NAV1,CD19	others	ENSOARG00000016099,ENSOARG00000018277,ENSOARG00000005952,ENSOARG00000010978,ENSOARG00000006400,ENSOARG00000012452,ENSOARG00000018279,ENSOARG00000003746,ENSOARG00000014240,ENSOARG00000000422,ENSOARG00000019517,ENSOARG00000020249,ENSOARG00000019611,ENSOARG00000013398,ENSOARG00000007493, ENSOARG00000018937,ENSOARG00000001710	17	0		17
**M61.0**	enriched in NK cells (II)	KLRB1,CD7,GPR56,NKG7,KIR3DP1,KIR3DX1,S1PR5,KIR2DS1,KLRF1,TGFBR3,IL2RB,CD96,CD247, PVRIG,TARP	Tc/NK	ENSOARG00000017210,ENSOARG00000016754,ENSOARG00000014768,ENSOARG00000002371,ENSOARG00000002102,ENSOARG00000021058,ENSOARG00000016047,ENSOARG00000019197,ENSOARG00000019179,ENSOARG00000012086,ENSOARG00000017076, ENSOARG00000017210,ENSOARG00000016754,ENSOARG00000014768,ENSOARG00000002217,ENSOARG00000002102,ENSOARG00000021058,ENSOARG00000016047,ENSOARG00000019197,ENSOARG00000019179,ENSOARG00000012086,ENSOARG00000017076	11	4	GPR56,S1PR5, KIR2DS1,TARP	15
**M61.1**	enriched in NK cells (KIR cluster)	KIR3PD1,KIR3DX1	Tc/NK	ENSOARG00000002102	1	1	KIR3PD1	2

**M61.2**	enriched in NK cells (receptor activation)	ITK,CD96,CARD11,CD244,CD247,ZAP70,PRKCQ,KIR3DP1,KIR3DX1,KLRD1	Tc/NK	ENSOARG00000011171,ENSOARG00000019179,ENSOARG00000004356,ENSOARG00000009009,ENSOARG00000012086,ENSOARG00000013646,ENSOARG00000013242,ENSOARG00000002371,ENSOARG00000002102, ENSOARG00000011171,ENSOARG00000019179,ENSOARG00000004356,ENSOARG00000009009,ENSOARG00000012086,ENSOARG00000013646,ENSOARG00000013242,ENSOARG00000002217,ENSOARG00000002102	9	1	KLRD1	10
**M62.0**	T & B cell development, activation	FKBP14,PTK2,CD1B,EBF1,CD3G,GAB1,CD7,GPM6B,ICOSLG,VPREB1,CD9,VPREB3,PSD3,TRAT1,SAMD13,RGL1,SCD,NDST3,ELOVL4,NTRK2,NAV1,RNF150,UBASH3A,LRP12,ACPL2,CAMK2D,TCL1A,GXYLT2,STK32B,ACCN2,GH1,ADCY9,MAL,VSIG10,CTGF,OGN,FAM150B,SH2D1A,CSRP2,CD3E,HHIP	others	ENSOARG00000008649,ENSOARG00000003418,ENSOARG00000007282,ENSOARG00000009256,ENSOARG00000010978,ENSOARG00000016754,ENSOARG00000011595,ENSOARG00000011738,ENSOARG00000008605,ENSOARG00000012733,ENSOARG00000004073,ENSOARG00000019045,ENSOARG00000013828,ENSOARG00000002070,ENSOARG00000014674,ENSOARG00000017811,ENSOARG00000007109,ENSOARG00000008891,ENSOARG00000018937,ENSOARG00000012236,ENSOARG00000010806,ENSOARG00000015579,ENSOARG00000018416,ENSOARG00000015981,ENSOARG00000002684,ENSOARG00000007185,ENSOARG00000013932,ENSOARG00000002932,ENSOARG00000003005,ENSOARG00000014240,ENSOARG00000008277,ENSOARG00000014085,ENSOARG00000014916,ENSOARG00000008984,ENSOARG00000010036, ENSOARG00000008649,ENSOARG00000003418,ENSOARG00000007291,ENSOARG00000009256,ENSOARG00000010978,ENSOARG00000016754,ENSOARG00000011595,ENSOARG00000011738,ENSOARG00000008605,ENSOARG00000012733,ENSOARG00000004073,ENSOARG00000019045,ENSOARG00000013828,ENSOARG00000002070,ENSOARG00000014674,ENSOARG00000017811,ENSOARG00000007109,ENSOARG00000008891,ENSOARG00000018937,ENSOARG00000012236,ENSOARG00000010806,ENSOARG00000015579,ENSOARG00000018416,ENSOARG00000015981,ENSOARG00000002684,ENSOARG00000007185,ENSOARG00000013932,ENSOARG00000002932,ENSOARG00000003005,ENSOARG00000014240, [illegible]	35	6	EBF1,VPREB1,ACPL2,ACCN2,MAL,FAM150B	41
**M62.1**	enriched for unknown TF motif CTCNANGTGNY	TMPRSS3,ACCN2,PTK2,EBF1,ENG,VSIG10,DTX3,MGC5566,ELOVL4,GXYLT2,SMPD3,HHIP	others	ENSOARG00000010761,ENSOARG00000003418,ENSOARG00000011387,ENSOARG00000003005,ENSOARG00000005952,ENSOARG00000007109,ENSOARG00000002684,ENSOARG00000003422,ENSOARG00000010036	9	3	ACCN2,EBF1,MGC5566	12
**M63**	regulation of localization (GO)	SNX17,TGFB1,ARAP1,AKT1,PKN1,MAP2K2,STXBP2,NAPA,ARHGDIA,FLNA,TSC2,NR1H2	others	ENSOARG00000019287,ENSOARG00000007468,ENSOARG00000005950,ENSOARG00000005558,ENSOARG00000010782,ENSOARG00000002236,ENSOARG00000011245,ENSOARG00000017985,ENSOARG00000005208,ENSOARG00000017283,ENSOARG00000013898	11	1	AKT1	12
**M64**	enriched in activated dendritic cells/monocytes	CXCL1,DRAM1,G0S2,SOD2,SIRPA,IL1RN,NINJ1,IL13RA1,TNFAIP2,SLC31A2,BASP1,MARCKS,FAM129A,ANPEP,SLC15A3,IL1B,CXCL8	DC/antigen presentation	ENSOARG00000014775,ENSOARG00000015467,ENSOARG00000004314,ENSOARG00000007425,ENSOARG00000020828,ENSOARG00000008116,ENSOARG00000016570,ENSOARG00000006395,ENSOARG00000009527,ENSOARG00000003263,ENSOARG00000011841,ENSOARG00000015974,ENSOARG00000020866,ENSOARG00000014496	14	3	SOD2,TNFAIP2,BASP1	17
**M65**	IL2, IL7, TCR network	IL7R,PTPRCAP,ITK,TRAC,IL2RG,LTB,JAK3,CCR7,LCK,BCL2,TRGC2,TRGC3,TRGC1,TRGC4,TRBC1,TRAC,TRDC	Tc/NK	ENSOARG00000011239,ENSOARG00000011983,ENSOARG00000011171,ENSOARG00000019445,ENSOARG00000016109,ENSOARG00000008259,ENSOARG00000014631,ENSOARG00000014777,ENSOARG00000020968,ENSOARG00000006262,ENSOARG00000001797,ENSOARG00000009188,ENSOARG00000017877,ENSOARG00000009129,ENSOARG00000006374,ENSOARG00000019445,ENSOARG00000019448, ENSOARG00000011239,ENSOARG00000011983,ENSOARG00000011171,ENSOARG00000019445,ENSOARG00000016109,ENSOARG00000008259,ENSOARG00000014631,ENSOARG00000014777,ENSOARG00000020968,ENSOARG00000006262,ENSOARG00000017970,ENSOARG00000009188,ENSOARG00000017877,ENSOARG00000009129,ENSOARG00000006374,ENSOARG00000019445,ENSOARG00000019448	17	0		17

**M66**	TBA	FCER1G,IDO1,CYP1B1,KMO,SLC7A7,KIAA1598,HMOX1,KYNU,ALDH2,CYBB,SLC31A2,ADAP2,IFI30,CST3,MAFB,BTK,CPVL	others	ENSOARG00000009800,ENSOARG00000002251,ENSOARG00000009090,ENSOARG00000008426,ENSOARG00000019424,ENSOARG00000018475,ENSOARG00000009938,ENSOARG00000010650,ENSOARG00000018943,ENSOARG00000006395,ENSOARG00000011933,ENSOARG00000013291,ENSOARG00000006202,ENSOARG00000001355,ENSOARG00000001620,ENSOARG00000008943	16	1	KIAA1598	17
**M67**	activated dendritic cells	CD38,DDX58,IRF7,PLSCR1,BIRC3,C1QC,IFIH1,SERPING1,DDX60,C2,IGKC,RSAD2	DC/antigen presentation	ENSOARG00000014731,ENSOARG00000006626,ENSOARG00000005557,ENSOARG00000006356,ENSOARG00000007991,ENSOARG00000006142,ENSOARG00000010035,ENSOARG00000014648, ENSOARG00000014731,ENSOARG00000006626,ENSOARG00000005557,ENSOARG00000006356,ENSOARG00000007991,ENSOARG00000006142,ENSOARG00000010035,ENSOARG00000014648	8	4	CD38,DDX60,C2,IGKC	12
**M68**	RIG-1 like receptor signaling	DDX58,IRF7,DHX58,TRIM25,NFKBIA,IFIH1,TNF,ISG15,CXCL10,CXCL8	IFN type I	ENSOARG00000014731,ENSOARG00000006626,ENSOARG00000018383,ENSOARG00000007011,ENSOARG00000007502,ENSOARG00000006142,ENSOARG00000008333,ENSOARG00000007233,ENSOARG00000016611,ENSOARG00000014496, ENSOARG00000014731,ENSOARG00000006626,ENSOARG00000018383,ENSOARG00000007011,ENSOARG00000007502,ENSOARG00000006142,ENSOARG00000008333,ENSOARG00000007233,ENSOARG00000016611,ENSOARG00000014496	10	0		10
**M69**	enriched in B cells (VI)	CR2,PCDH9,TSPAN13,HLA-DOB,CD72,PTPRK,CD22,P2RX5,BTLA,MS4A1,PPAPDC1B,CXCR5,ABCB4,CD200,FCRL1,FCRL2,CD79A,CD79B,ADAM28,CD19	B cells	ENSOARG00000008208,ENSOARG00000014957,ENSOARG00000008934,ENSOARG00000007458,ENSOARG00000011957,ENSOARG00000013550,ENSOARG00000004796,ENSOARG00000017481,ENSOARG00000019308,ENSOARG00000013081,ENSOARG00000011654,ENSOARG00000019298,ENSOARG00000006960,ENSOARG00000007086,ENSOARG00000008358,ENSOARG00000014015,ENSOARG00000010026,ENSOARG00000001710	18	2	PPAPDC1B,ABCB4	20
**M70.0**	TBA	CDKN2B,TCF15,PDLIM7,IL4I1,NUP62,SIGLEC11,ELAVL3,LTB4R2,SEC14L2,CFB,SAA1,SAA2,PSD,ADARB1,FUT7,ZNF205,CES4,SIX5,COL16A1,GP1BB,MSC,CLDN5,MAPRE3	others	ENSOARG00000019015,ENSOARG00000005380,ENSOARG00000013677,ENSOARG00000013686,ENSOARG00000014823,ENSOARG00000018464,ENSOARG00000000653,ENSOARG00000012452,ENSOARG00000001319,ENSOARG00000010273,ENSOARG00000001113,ENSOARG00000004844,ENSOARG00000019040	13	10	CDKN2B,LTB4R2,SEC14L2,CFB,SAA1,SAA2,FUT7,CES4,GP1BB,CLDN5	23
**M70.1**	TBA	CDKN2B,PDLIM7,ELAVL3,SIX5,PIK3CD,SOX15,PSD,FUT7,BOK,FOXO4	others	ENSOARG00000005380,ENSOARG00000018464,ENSOARG00000010273,ENSOARG00000007664,ENSOARG00000014928,ENSOARG00000000653,ENSOARG00000017652,ENSOARG00000016065	8	2	CDKN2B,FUT7	10
**M71**	enriched in antigen presentation (I)	CD74,CYP1B1,PLAUR,PTGS2,HMOX1,AGPAT9,SLC27A2,ASPH,RTN1,FAR2,ITPR3,DQA,HLA-DRA,OVAR-DM,OVAR-DQA1,OVAR-DQA2,OVAR-DQB2,OVAR-DRB1,OVAR-DRB5,OVAR-DRB3	DC/antigen presentation	ENSOARG00000007229,ENSOARG00000009090,ENSOARG00000008867,ENSOARG00000007592,ENSOARG00000018475,ENSOARG00000003059,ENSOARG00000020994,ENSOARG00000016594,ENSOARG00000021121,ENSOARG00000019642,ENSOARG00000010180,ENSOARG00000015485,ENSOARG00000016610	13	7	OVAR-DM,OVAR-DQA1,OVAR-DQA2,OVAR-DQB2,OVAR-DRB1,OVAR-DRB5,OVAR-DRB3	20
**M72.0**	TBA	MORC3,MUDENG,ADNP,DEK,ATP11B,FMR1,ARID4B,NEK7,PANK3,ORC4L,PLEKHF2,MST4,ITSN2,SACM1L,RNF138,RAP2C,PHTF2,ZBTB11,UBA3,EFHA1,PTPN11,ROCK1,RNF6,BZW1	others	ENSOARG00000013952,ENSOARG00000013868,ENSOARG00000010301,ENSOARG00000020665,ENSOARG00000009673,ENSOARG00000003481,ENSOARG00000015482,ENSOARG00000002732,ENSOARG00000014688,ENSOARG00000017925,ENSOARG00000010162,ENSOARG00000006124,ENSOARG00000012473,ENSOARG00000017915,ENSOARG00000018474,ENSOARG00000010086,ENSOARG00000009565,ENSOARG00000008819,ENSOARG00000012743,ENSOARG00000016520	20	4	MUDENG,ORC4L,MST4,EFHA1	24
**M72.1**	TBA	NEK7,ITSN2,MUDENG,ERBB2IP,PLEKHF2,G3BP2,IMPA1,SP3,DEK,PPP1R12A,VPS4B,CCNC,FMR1,MBNL1,ARID4B,BZW1,SACM1L	others	ENSOARG00000015482,ENSOARG00000017925,ENSOARG00000006023,ENSOARG00000014688,ENSOARG00000015652,ENSOARG00000015101,ENSOARG00000000469,ENSOARG00000010301,ENSOARG00000015054,ENSOARG00000006303,ENSOARG00000011800,ENSOARG00000009673,ENSOARG00000003701,ENSOARG00000003481,ENSOARG00000016520,ENSOARG00000010162	16	1	MUDENG	17
**M72.2**	TBA	ZDHHC17,LBR,ERBB2IP,PLEKHF2,STAG2,RNF111,SP3,PPP1R12A,G3BP2,FMR1,ARID4B,SACM1L	others	ENSOARG00000014899,ENSOARG00000017157,ENSOARG00000006023,ENSOARG00000014688,ENSOARG00000013895,ENSOARG00000020839,ENSOARG00000000469,ENSOARG00000015054,ENSOARG00000015652,ENSOARG00000009673,ENSOARG00000003481,ENSOARG00000010162	12	0		12

**M73**	enriched in monocytes (III)	CEBPB,DUSP6,MAML3,TFEC,FOS,IER3,CCR1,RIN2,GPR109B,BCL6,PLXDC2,TIMP2	myeloid cells/inflammation	ENSOARG00000013395,ENSOARG00000015628,ENSOARG00000012912,ENSOARG00000001668,ENSOARG00000001783,ENSOARG00000011463,ENSOARG00000014260,ENSOARG00000001865,ENSOARG00000020487,ENSOARG00000000344,ENSOARG00000003264	11	1	GPR109B	12
**M74**	transcriptional targets of glucocorticoid receptor	LOXL1,PPIL4,ZC3H12D,KCTD15,PSD3,SCD,CTGF,MEF2C,JUP,HLA-DOA,CD3E,BTK	others	ENSOARG00000004368,ENSOARG00000002675,ENSOARG00000002626,ENSOARG00000004464,ENSOARG00000004073,ENSOARG00000014674,ENSOARG00000014240,ENSOARG00000016099,ENSOARG00000017474,ENSOARG00000008091,ENSOARG00000008984,ENSOARG00000001620	12	0		12
**M75**	antiviral IFN signature	IFIH1,ELANE,SERPING1,IL1B,RSAD2,IFIT1,RARA,DDX58,FCER1A,DHX58,PTX3,CARD9,OAS1,OAS3,PML,ANXA3,HERC5,DDX60,CXCL10,IRF7,C1QB,BCL3	IFN type I	ENSOARG00000006142,ENSOARG00000009696,ENSOARG00000010035,ENSOARG00000020866,ENSOARG00000014648,ENSOARG00000015177,ENSOARG00000014119,ENSOARG00000014731,ENSOARG00000007787,ENSOARG00000018383,ENSOARG00000002754,ENSOARG00000004519,ENSOARG00000002881,ENSOARG00000004233,ENSOARG00000018710,ENSOARG00000000530,ENSOARG00000016611,ENSOARG00000006626,ENSOARG00000007977,ENSOARG00000009239	20	2	OAS3,DDX60	22
**M76**	DNA repair	PCNA,RFC5,RFC4,RFC3,RFC2,FEN1,FANCD2,RPA1,MRE11A,FANCG,POLD1,POLD3,LIG1,FANCI,BRCA1,BRCA2,GTF2H1,H2AFX,RPA3,POLE2,RAD51,RAD50	cell cycle	ENSOARG00000017133,ENSOARG00000003356,ENSOARG00000020512,ENSOARG00000010697,ENSOARG00000011737,ENSOARG00000015633,ENSOARG00000005211,ENSOARG00000014479,ENSOARG00000002115,ENSOARG00000012171,ENSOARG00000013923,ENSOARG00000010224,ENSOARG00000011565,ENSOARG00000011362,ENSOARG00000004835,ENSOARG00000011179,ENSOARG00000009671,ENSOARG00000017039,ENSOARG00000005725,ENSOARG00000020607,ENSOARG00000020254,ENSOARG00000015021	22	0		22
**M77**	collagen, TGFB family et al	PDGFRB,PDGFRA,PTK2,BCL11B,VDR,COL3A1,EDNRA,CD9,SEMA6A,CAMK1,JAM3,SULF1,JUP,NPY,THY1,TCF4,ENG,CD24,COL5A2,IGFBP3,COL4A2,COL4A1,TGFB1I1,SNAI2,ADM,SHANK3,GATA3,BMP2,FN1,POU4F1,MEF2C,FZD8,COL1A2,COL6A3,COL6A2	others	ENSOARG00000006538,ENSOARG00000019070,ENSOARG00000003418,ENSOARG00000000266,ENSOARG00000019372,ENSOARG00000016476,ENSOARG00000008169,ENSOARG00000008605,ENSOARG00000001863,ENSOARG00000006159,ENSOARG00000013611,ENSOARG00000003329,ENSOARG00000017474,ENSOARG00000010915,ENSOARG00000016042,ENSOARG00000005018,ENSOARG00000011387,ENSOARG00000011081,ENSOARG00000016440,ENSOARG00000012862,ENSOARG00000006515,ENSOARG00000006115,ENSOARG00000009529,ENSOARG00000013030,ENSOARG00000011444,ENSOARG00000020018,ENSOARG00000013941,ENSOARG00000018738,ENSOARG00000019329,ENSOARG00000016099,ENSOARG00000001508,ENSOARG00000019080,ENSOARG00000012880	33	2	POU4F1,FZD8	35
**M78**	myeloid cell cytokines, metallopeptidases and laminins	IL1RN,IL23A,IL10,MMP19,IL6,LAMC2,MMP9,EDN1,SFN,LAMB3,GSN	myeloid cells/inflammation	ENSOARG00000020828,ENSOARG00000009246,ENSOARG00000006292,ENSOARG00000010943,ENSOARG00000012021,ENSOARG00000000744,ENSOARG00000007908,ENSOARG00000014073,ENSOARG00000018302,ENSOARG00000013143,ENSOARG00000005473	11	0		11
**M79**	TBA	CXCL3,RNASE4,TFEC,DPYD,MGAM,FAM20C,SULF2,AQP9,ANPEP,TBXAS1	others	ENSOARG00000014841,ENSOARG00000012938,ENSOARG00000001668,ENSOARG00000017549,ENSOARG00000014509,ENSOARG00000006178,ENSOARG00000010080,ENSOARG00000020858,ENSOARG00000011841,ENSOARG00000011915	10	0		10
**M80**	TBA	MEF2C,PTK2,FAM43A,ENG,RGL1,PLCB1,NDST3,GNG11,MGC5566,GAB1,LARGE,FAM69B,CTGF,TRAT1,MARCH3,GPR132,CCR9,CRIM1,HCP5,BLNK	others	ENSOARG00000016099,ENSOARG00000003418,ENSOARG00000020399,ENSOARG00000011387,ENSOARG00000002070,ENSOARG00000009195,ENSOARG00000017811,ENSOARG00000001101,ENSOARG00000010978,ENSOARG00000018279,ENSOARG00000003746,ENSOARG00000014240,ENSOARG00000019045,ENSOARG00000018055,ENSOARG00000009514,ENSOARG00000010127,ENSOARG00000007320	17	3	MGC5566,GPR132,HCP5	20

**M81**	enriched in myeloid cells and monocytes	LIN7A,MYH10,PTGS1,EHD3,PCSK6,CCR2,CHI3L1,EGF,CLEC5A,FCER1G,NLRP3,ITGA2B,MMRN1,CSF1R,THBS1,SPARC,MARCKS,SLC8A1,PDGFA,ITGB3,HHEX,PROS1,TF,CLU,CXCL12,PRAM1,ACTN1,F5,CD14,FKBP1B,TLR7,TLR4,SNCA,BCL6,SELP	myeloid cells/inflammation	ENSOARG00000015171,ENSOARG00000001617,ENSOARG00000014116,ENSOARG00000019698,ENSOARG00000016657,ENSOARG00000014236,ENSOARG00000001370,ENSOARG00000014270,ENSOARG00000009800,ENSOARG00000007591,ENSOARG00000018398,ENSOARG00000006358,ENSOARG00000020058,ENSOARG00000009032,ENSOARG00000009527,ENSOARG00000006153,ENSOARG00000017273,ENSOARG00000008456,ENSOARG00000002991,ENSOARG00000004053,ENSOARG00000021179,ENSOARG00000010135,ENSOARG00000025180,ENSOARG00000017806,ENSOARG00000011288,ENSOARG00000005792,ENSOARG00000018510,ENSOARG00000020487,ENSOARG00000010726	29	6	EGF,ITGA2B,SLC8A1,ITGB3,HHEX,CLU	35
**M82**	signal transduction, plasma membrane	SFRP2,PDGFRB,PDGFRA,FN1,FAP,IGFBP5,DOCK4,NR2F2,COL1A2,COL1A1,SNAI2,LAMC1,DLC1	others	ENSOARG00000001680,ENSOARG00000006538,ENSOARG00000019070,ENSOARG00000019329,ENSOARG00000006246,ENSOARG00000019421,ENSOARG00000002931,ENSOARG00000010167,ENSOARG00000001508,ENSOARG00000004871,ENSOARG00000013030,ENSOARG00000019180,ENSOARG00000009748	13	0		13
**M83**	enriched in naive and memory B cells	NR3C2,PCDH9,NRXN3,PKIG,FCGR2B,CD37,MEF2C,BANK1,MS4A1,LY86	B cells	ENSOARG00000014957,ENSOARG00000002748,ENSOARG00000004128,ENSOARG00000010094,ENSOARG00000012851,ENSOARG00000016099,ENSOARG00000013398,ENSOARG00000013081,ENSOARG00000018347	9	1	NR3C2	10
**M84**	integrins and cell adhesion	SELL,ITGB2,LILRB2,ITGB7,CD96,AMICA1,ITGA4,ICAM3,VCAM1,ITGAL	others	ENSOARG00000010897,ENSOARG00000012367,ENSOARG00000016642,ENSOARG00000019179,ENSOARG00000016867,ENSOARG00000015922,ENSOARG00000006423	7	3	LILRB2,AMICA1,VCAM1	10
**M85**	platelet activation and degranulation	SERPINA1,F3,TIMP1,FN1,THBS2,THBS1,SPARC,COL1A2,COL1A1,CLU,VWF,SERPINE1	myeloid cells/inflammation	ENSOARG00000014882,ENSOARG00000017328,ENSOARG00000013157,ENSOARG00000019329,ENSOARG00000005037,ENSOARG00000020058,ENSOARG00000009032,ENSOARG00000001508,ENSOARG00000004871,ENSOARG00000008752,ENSOARG00000015262	11	1	CLU	12
**M86.0**	chemokines and inflammatory molecules in myeloid cells	CXCL1,TRAF1,ADORA2A,CYTSA,CXCL2,NFKBIA,CCL4,BCL2A1,GCH1,G0S2,TNFAIP3,TNFAIP6,TNF,HS3ST3B1,NFKB1,SLAMF7,CCL20,IL1B,DUSP1,PDE4B	myeloid cells/inflammation	ENSOARG00000014775,ENSOARG00000005613,ENSOARG00000014207,ENSOARG00000014775,ENSOARG00000007502,ENSOARG00000004253,ENSOARG00000015167,ENSOARG00000021078,ENSOARG00000004314,ENSOARG00000000569,ENSOARG00000009019,ENSOARG00000008333,ENSOARG00000012718,ENSOARG00000008967,ENSOARG00000020576, ENSOARG00000020866,ENSOARG00000003963,ENSOARG00000010521, ENSOARG00000014775,ENSOARG00000005613,ENSOARG00000014207,ENSOARG00000014775,ENSOARG00000007502,ENSOARG00000004253,ENSOARG00000015167,ENSOARG00000021078,ENSOARG00000004314,ENSOARG00000000569,ENSOARG00000009019,ENSOARG00000008333,ENSOARG00000012718,ENSOARG00000008967,ENSOARG00000020576, ENSOARG00000020866,ENSOARG00000003963,ENSOARG00000010521	18	2	CYTSA,HS3ST3B1	20
**M86.1**	proinflammatory dendritic cell, myeloid cell response	ADORA2A,CYTSA,STAT4,CCL4,TNFAIP6,IFNG,CD80,GCH1,SERPINB9,TNF,HS3ST3B1,CCL20,IL1B,PDE4B	myeloid cells/inflammation	ENSOARG00000014207,ENSOARG00000013903,ENSOARG00000004253,ENSOARG00000009019,ENSOARG00000001958,ENSOARG00000019762,ENSOARG00000021078,ENSOARG00000002211,ENSOARG00000008333,ENSOARG00000020576,ENSOARG00000020866,ENSOARG00000010521	12	2	CYTSA,HS3ST3B1	14

**M87**	transmembrane transport (I)	VDR,SLC7A7,CD36,SLC31A2,SLC4A1,TLR2,NLRP3,SLC11A1,ABCB6,CDH1,DAPK1,SLC14A1,AQP1,HK3,ATP1B2,AQP9,SLC25A21,SLC15A3,ABCG2,KCNH2,HMOX1,SLC22A4,RHAG,TLR4	DC/antigen presentation	ENSOARG00000019372,ENSOARG00000019424,ENSOARG00000017123,ENSOARG00000006395,ENSOARG00000007821,ENSOARG00000015044,ENSOARG00000007591,ENSOARG00000019535,ENSOARG00000019920,ENSOARG00000003455,ENSOARG00000008706,ENSOARG00000002395,ENSOARG00000007775,ENSOARG00000003252,ENSOARG00000015381,ENSOARG00000020858,ENSOARG00000008179,ENSOARG00000015974,ENSOARG00000001914,ENSOARG00000001434,ENSOARG00000018475,ENSOARG00000015213,ENSOARG00000012291,ENSOARG00000005792, ENSOARG00000019372,ENSOARG00000019424,ENSOARG00000017123,ENSOARG00000006395,ENSOARG00000007821,ENSOARG00000015044,ENSOARG00000007591,ENSOARG00000019535,ENSOARG00000019920,ENSOARG00000003455,ENSOARG00000008706,ENSOARG00000002395,ENSOARG00000007775,ENSOARG00000003252,ENSOARG00000015381,ENSOARG00000020858,ENSOARG00000008179,ENSOARG00000015974,ENSOARG00000001914,ENSOARG00000001434,ENSOARG00000018475,ENSOARG00000015213,ENSOARG00000012291,ENSOARG00000005792	24	0		24
**M88.0**	leukocyte migration	C17orf73,HEPH,CDH17,POF1B,CDX1,CDX2,FAM3D,MYO1A,NOX1,PRSS3,C19orf21,FABP1,C2orf89,GPR35,BCL2L14,TMEM45B,MUC3B,PRR15L,TMPRSS2,CEACAM1,VIL1,GMDS,CEACAM5,CLDN3,CLDN4,KRT20,CLDN7,PLS1,XK,GUCY2C,GPX2,EPB41L4B,ATP10B,RNF43,ST14,EPCAM,TSPAN8,EPS8L3,MUC13,LGALS4,PIP5K1B,FA2H,ACSL5,MAPK13,NR1I2,FOXA3,FUT4,FUT3,MYO7B,GPA33,MEP1A	myeloid cells/inflammation	ENSOARG00000005331,ENSOARG00000008009,ENSOARG00000002376,ENSOARG00000006690,ENSOARG00000012435,ENSOARG00000012025,ENSOARG00000007766,ENSOARG00000001070,ENSOARG00000020752,ENSOARG00000020888,ENSOARG00000014210,ENSOARG00000004462,ENSOARG00000010334,ENSOARG00000019575,ENSOARG00000002773,ENSOARG00000010845,ENSOARG00000025184,ENSOARG00000015493,ENSOARG00000011311,ENSOARG00000006047,ENSOARG00000018927,ENSOARG00000020763,ENSOARG00000021156,ENSOARG00000007009,ENSOARG00000013606,ENSOARG00000010239,ENSOARG00000014848,ENSOARG00000005218,ENSOARG00000014542,ENSOARG00000019334,ENSOARG00000020227,ENSOARG00000005839,ENSOARG00000013075,ENSOARG00000006220,ENSOARG00000011792,ENSOARG00000013006,ENSOARG00000019817,ENSOARG00000009829,ENSOARG00000016054,ENSOARG00000011969,ENSOARG00000011224	41	10	C17orf73,PRSS3,C19orf21,C2orf89,GPR35,MUC3B,CEACAM1,CEACAM5,FOXA3,FUT3	51
**M88.1**	enriched in hepatocyte nuclear factors (I)	FOXA2,GMDS,MUC13,HNF4G,HNF4A,HNF1B,FOXA3,C19orf21,CEACAM5,PIP5K1B,EPS8L3,MNX1,LGALS4	others	ENSOARG00000005070,ENSOARG00000002773,ENSOARG00000020227,ENSOARG00000007140,ENSOARG00000003928,ENSOARG00000003338,ENSOARG00000013075,ENSOARG00000019334,ENSOARG00000007317,ENSOARG00000005839	10	3	FOXA3,C19orf21,CEACAM5	13
**M88.2**	enriched in hepatocyte nuclear factors (II)	FOXA2,CDH17,GUCY2C,HNF4G,HNF4A,CALML4,HNF1B,FOXA3,C19orf21,CEACAM5,PIP5K1B,EPCAM	others	ENSOARG00000005070,ENSOARG00000008009,ENSOARG00000020763,ENSOARG00000007140,ENSOARG00000003928,ENSOARG00000018411,ENSOARG00000003338,ENSOARG00000013075,ENSOARG00000005218	9	3	FOXA3,C19orf21,CEACAM5	12
**M89.0**	putative targets of PAX3	PLK2,G0S2,CXCL2,SIK1,EGR2,EGR3,CD83,EGR1,NR4A1,HBEGF,NR4A2,IL8,MAFF,CDKN1A,GEM,DUSP2	others	ENSOARG00000007234,ENSOARG00000004314,ENSOARG00000014775,ENSOARG00000004489,ENSOARG00000010199,ENSOARG00000012936,ENSOARG00000016470,ENSOARG00000017214,ENSOARG00000017521,ENSOARG00000007984,ENSOARG00000014496,ENSOARG00000015236,ENSOARG00000013636,ENSOARG00000007839,ENSOARG00000014009	15	1	SIK1	16
**M89.1**	putative targets of PAX3	EGR2,EGR3,CD83,NR4A1,INHBA,NR4A2,RGS1,DUSP1,ATF3,DUSP2	others	ENSOARG00000004489,ENSOARG00000010199,ENSOARG00000012936,ENSOARG00000017214,ENSOARG00000017475,ENSOARG00000007984,ENSOARG00000009791,ENSOARG00000003963,ENSOARG00000010493,ENSOARG00000014009	10	0		10
**M90**	TBA	HMGCS2,SLC3A1,CRABP2,CDX1,HNF4G,RNF186,MYO1A,SLC39A5,NR1I2,DDC,GIPC2,PPP1R14D	others	ENSOARG00000020427,ENSOARG00000006308,ENSOARG00000006212,ENSOARG00000006690,ENSOARG00000007140,ENSOARG00000009608,ENSOARG00000007766,ENSOARG00000009687,ENSOARG00000019817,ENSOARG00000014391,ENSOARG00000013390,ENSOARG00000020288	12	0		12

**M91**	adhesion and migration, chemotaxis	ANXA1,CX3CR1,CCL5,CCL19,PROK2,F2R,CXCR2,ADRB2,CCR5,CCR7,CCL21,CXCL13,PF4	myeloid cells/inflammation	ENSOARG00000012672,ENSOARG00000014010,ENSOARG00000004964,ENSOARG00000009119,ENSOARG00000016937,ENSOARG00000019477,ENSOARG00000014352,ENSOARG00000014214,ENSOARG00000014777,ENSOARG00000009107,ENSOARG00000017732, ENSOARG00000012672,ENSOARG00000014010,ENSOARG00000004964,ENSOARG00000009119,ENSOARG00000016937,ENSOARG00000019477,ENSOARG00000014352,ENSOARG00000014214,ENSOARG00000014777,ENSOARG00000009107,ENSOARG00000017732	11	2	PROK2,PF4	13
**M92**	lipid metabolism, endoplasmic reticulum	PTGS2,CYP4F3,CYP1B1,TBXAS1,CYP4F12,MGST1,CYP3A7,CYP3A4,CYP3A5,CYP4F2	others	ENSOARG00000007592,ENSOARG00000002100,ENSOARG00000009090,ENSOARG00000011915,ENSOARG00000020582	5	5	CYP4F12,CYP3A7,CYP3A4,CYP3A5,CYP4F2	10
**M93**	TBA	TGFBR3,LIF,IL23A,PRKCA,CRTAM,PLAU,CAMK4,TNF,OSM,PTCH1	others	ENSOARG00000016047,ENSOARG00000006322,ENSOARG00000009246,ENSOARG00000015686,ENSOARG00000018699,ENSOARG00000008473,ENSOARG00000000184,ENSOARG00000008333,ENSOARG00000006353,ENSOARG00000008578	10	0		10
**M94**	growth factor induced, enriched in nuclear receptor subfamily 4	PPP1R15A,PLK2,CYR61,NR4A2,EGR3,CDKN1A,NR4A1,EPHA2,ID1,IL6,NR4A3,DUSP1	others	ENSOARG00000012344,ENSOARG00000007234,ENSOARG00000014336,ENSOARG00000007984,ENSOARG00000010199,ENSOARG00000013636,ENSOARG00000017214,ENSOARG00000012400,ENSOARG00000001111,ENSOARG00000012021,ENSOARG00000010939,ENSOARG00000003963	12	0		12
**M95.0**	enriched in antigen presentation (II)	CD53,IL10RA,ITGA4,FYB,HCLS1,PTPRC,ITGB2,GIMAP6,EVI2B,PLEK,SLA,SELL,IRF8,FGL2,ITGAL,ITGAM,AIF1,HLA-DMA,HLA-DRA,OVAR-DM,OVAR-DQA1,OVAR-DQA2,OVAR-DQB1,OVAR-DQB2,OVAR-DRB1,OVAR-DRB5,OVAR-DRB3	DC/antigen presentation	ENSOARG00000019463,ENSOARG00000007625,ENSOARG00000016867,ENSOARG00000009551,ENSOARG00000020050,ENSOARG00000015718,ENSOARG00000012367,ENSOARG00000001356,ENSOARG00000003991,ENSOARG00000019902,ENSOARG00000005721,ENSOARG00000010897,ENSOARG00000011513,ENSOARG00000018670,ENSOARG00000006423,ENSOARG00000009000,ENSOARG00000007932,ENSOARG00000007972,ENSOARG00000016610	19	8	OVAR-DM,OVAR-DQA1,OVAR-DQA2,OVAR-DQB1,OVAR-DQB2,OVAR-DRB1,OVAR-DRB5,OVAR-DRB3	27
**M95.1**	enriched in antigen presentation (III)	FYB,CD53,ITGB2,ICAM1,PLEK,LAPTM5,PTPRC,EVI2B,HLA-DMB,AIF1,HLA-DMA,HLA-DRA,OVAR-DM,OVAR-DRB1,OVAR-DRB5,OVAR-DRB3	DC/antigen presentation	ENSOARG00000009551,ENSOARG00000019463,ENSOARG00000012367,ENSOARG00000015656,ENSOARG00000019902,ENSOARG00000002094,ENSOARG0000015718,ENSOARG00000003991,ENSOARG00000007932,ENSOARG00000007972,ENSOARG00000016610	11	5	HLA-DMB,OVAR-DM,OVAR-DRB1,OVAR-DRB5,OVAR-DRB3	16
**M96**	Hox cluster V	PRDM16,CPNE8,HOXB2,HOXB3,HOXB7,HOXA11,HOXA4,HOXA2,NKX2-3,HOXA9	others	ENSOARG00000017237,ENSOARG00000020074,ENSOARG00000007134,ENSOARG00000007106,ENSOARG00000006908,ENSOARG00000009789,ENSOARG00000009842,ENSOARG00000012498,ENSOARG00000009680	9	1	HOXA11	10
**M97**	enriched for SMAD2/3 signaling	P4HA2,TCL1A,RGL1,RUNX2,MEF2C,MME,BIVM,LAMC1,VPREB1,GATA3	others	ENSOARG00000015394,ENSOARG00000015981,ENSOARG00000002070,ENSOARG00000010358,ENSOARG00000016099,ENSOARG00000003370,ENSOARG0000019180,ENSOARG00000013941	8	2	BIVM,VPREB1	10
**M98.0**	TBA	PLUNC,DMD,VSNL1,ERBB4,SPINLW1,OR10H3,TAS2R16,OPRM1,GRIN2B,HOXC10,DEFA5,HTR3B,GFRA3,C4orf31,ADH7,CYP11B1,NOS1AP,A2BP1	others	ENSOARG00000018256,ENSOARG00000016578,ENSOARG00000019232,ENSOARG00000001125,ENSOARG00000004083,ENSOARG00000020802,ENSOARG00000016331,ENSOARG00000000277,ENSOARG00000016086,ENSOARG00000000864,ENSOARG00000010597	11	7	PLUNC,SPINLW1,OR10H3,DEFA5,C4orf31,ADH7,A2BP1	18
**M98.1**	TBA	PLUNC,DMD,TUBB4,SLC7A11,DBC1,GRIN2B,DEFA5,CYP11B1,A2BP1,IFN-ALPHA	others	ENSOARG00000018256,ENSOARG00000014253,ENSOARG00000020802,ENSOARG00000000864	4	6	PLUNC,TUBB4,DBC1,DEFA5,A2BP1,IFN-ALPHA	10
**M99**	TBA	PDGFRA,GREM1,CYR61,EPHA2,MRGPRF,FSTL1,INHBA,CTGF,DACT1,CAV1	others	ENSOARG00000019070,ENSOARG00000014336,ENSOARG00000012400,ENSOARG00000017116,ENSOARG00000019900,ENSOARG00000017475,ENSOARG00000014240,ENSOARG00000021114,ENSOARG00000001337	9	1	GREM1	10
**M100**	MAPK, RAS signaling	MAP4K1,RASGRP2,RAC2,RASGRP4,AKT1,TGFB1,MAP2K2,MAP3K11,ARRB2,FLNA	others	ENSOARG00000005746,ENSOARG00000008574,ENSOARG00000005647,ENSOARG00000007468,ENSOARG00000010782,ENSOARG00000016243,ENSOARG00000007797,ENSOARG00000005208	8	2	RAC2,AKT1	10
**M101**	phosphatidylinositol signaling system	PHTF2,AGL,C6orf211,PIKFYVE,SP3,IMPA1,PIK3C2A,DEK,SYNJ1,PPP1R12A,CLK1,PIK3C3,EFHA1,SLC35A1	others	ENSOARG00000017915,ENSOARG00000017762,ENSOARG00000019028,ENSOARG00000000469,ENSOARG00000015101,ENSOARG00000004372,ENSOARG00000010301,ENSOARG00000013414,ENSOARG00000015054,ENSOARG00000016541,ENSOARG00000005184,ENSOARG00000013049	12	2	C6orf211,EFHA1	14

**M102**	TBA	AMOTL2,GHR,P2RX5,BTLA,CHRDL1,ELTD1,GNG12,SMAD1,STAP1,PNOC,CAV2,CD79A	others	ENSOARG00000008268,ENSOARG00000008837,ENSOARG00000017481,ENSOARG00000019308,ENSOARG00000017833,ENSOARG00000011369,ENSOARG00000009089,ENSOARG00000007050,ENSOARG00000014922,ENSOARG00000001379,ENSOARG00000008358	11	1	ELTD1	12
**M103**	cell cycle (III)	SMC1A,HDAC2,BUB1B,PLK1,CDKN1B,CDKN1A,PKMYT1,MAD2L1,ATR,CDC6,CDC7,CCNA2,CCNE2,SMC3,CCNE1,ANAPC10,TTK,ESPL1,DBF4,WEE1,CHEK2,CHEK1,PCNA,CDKN2C,CDKN2A,BUB1,RBL1,RBL2,RAD21,CDC25C,CDC25A,E2F5,MCM7,MCM6,MCM5,MCM4,MCM3,MCM2,PTTG1,SKP2,CCNB2,CCNB1,E2F2,E2F1,CDC23,CDC20,CDK1,CDK2,CDK4,CDC45,TFDP1	cell cycle	ENSOARG00000008581,ENSOARG00000009493,ENSOARG00000020126,ENSOARG00000017343,ENSOARG00000020853,ENSOARG00000013636,ENSOARG00000000736,ENSOARG00000015665,ENSOARG00000006192,ENSOARG00000014063,ENSOARG00000015943,ENSOARG00000014176,ENSOARG00000006180,ENSOARG00000009745,ENSOARG00000004067,ENSOARG00000009933,ENSOARG00000007151,ENSOARG00000016587,ENSOARG00000013264,ENSOARG00000012355,ENSOARG00000002461,ENSOARG00000011655,ENSOARG00000017133,ENSOARG00000004421,ENSOARG00000014305,ENSOARG00000017221,ENSOARG00000017777,ENSOARG00000010359,ENSOARG00000016166,ENSOARG00000002373,ENSOARG00000013715,ENSOARG00000017802,ENSOARG00000010614,ENSOARG00000018527,ENSOARG00000012797,ENSOARG00000014143,ENSOARG00000002774,ENSOARG00000020836,ENSOARG00000012913,ENSOARG00000007334,ENSOARG00000008548,ENSOARG00000015982,ENSOARG00000020542,ENSOARG00000004318,ENSOARG00000010667,ENSOARG00000005320,ENSOARG00000017745	47	4	CDKN2A,PTTG1,SKP2,TFDP1	51
**M104**	TBA	PDGFRA,ZNF423,MGLL,GNAZ,FSTL1,MEF2C,JUP,MLLT3,CRIM1,PF4	others	ENSOARG00000019070,ENSOARG00000017044,ENSOARG00000005578,ENSOARG00000019900,ENSOARG00000016099,ENSOARG00000017474,ENSOARG00000014289,ENSOARG00000010127	8	2	GNAZ,PF4	10
**M105**	TBA	CDKN2C,H1F0,BEX1,PROK2,BPI,CA2,PTX3,RETN,CLEC11A,SLC22A4,MGST1,LPCAT2,IGFBP7,CPNE2,IRAK3,JAG1,VSTM1,BTBD3	others	ENSOARG00000004421,ENSOARG00000020372,ENSOARG00000018736,ENSOARG00000012954,ENSOARG00000002754,ENSOARG00000002291,ENSOARG00000015213,ENSOARG00000020582,ENSOARG00000018091,ENSOARG00000005761,ENSOARG00000018900,ENSOARG00000002799,ENSOARG00000010523,ENSOARG00000010789	14	4	BEX1,PROK2,CLEC11A,VSTM1	18
**M106.0**	nuclear pore complex	NUP88,XPO1,NUP62,NUPL2,NUP133,NUP107,RAN,NUP160,NUP54,NUP35,NUP85,NUP37,NUP43,NUP155,SEH1L,RANBP1,NUP205	others	ENSOARG00000003688,ENSOARG00000020174,ENSOARG00000013686,ENSOARG00000012602,ENSOARG00000003060,ENSOARG00000000362,ENSOARG00000010707,ENSOARG00000007967,ENSOARG00000016734,ENSOARG00000016728,ENSOARG00000010987,ENSOARG00000015613,ENSOARG00000002841,ENSOARG00000010255,ENSOARG00000018318,ENSOARG00000009081	16	1	SEH1L	17
**M106.1**	nuclear pore complex (mitosis)	SMC1A,SLBP,SEH1L,NUP133,NUP107,GTF2H1,NUP85,NUP37,NUP43,CCNH,NUP160	others	ENSOARG00000008581,ENSOARG00000016090,ENSOARG00000003060,ENSOARG00000000362,ENSOARG00000009671,ENSOARG00000010987,ENSOARG00000015613,ENSOARG00000002841,ENSOARG00000015931,ENSOARG00000007967	10	1	SEH1L	11
**M107**	Hox cluster VI	HOXB9,HOXB2,HOXB3,HOXB6,HOXA10,HOXA6,HOXA4,HOXA3,HOXA2,C10orf140,HOXA9	others	ENSOARG00000006872,ENSOARG00000007134,ENSOARG00000007106,ENSOARG00000007002,ENSOARG00000009675,ENSOARG00000009758,ENSOARG00000009789,ENSOARG00000009797,ENSOARG00000009842,ENSOARG00000009680	10	1	C10orf140	11
**M108**	TBA	EPHB2,MUC3B,AXIN2,GPX2,ASCL2,RNF43,EHF,C19orf21,CEACAM5,CDCA7,TSPAN8	others	ENSOARG00000007903,ENSOARG00000015486,ENSOARG00000021156,ENSOARG00000010239,ENSOARG00000018677,ENSOARG00000000578,ENSOARG00000014542	7	4	MUC3B,ASCL2,C19orf21,CEACAM5	11
**M109**	receptors, cell migration	TGFBR3,NCKAP1L,LRRK2,C3AR1,S1PR1,SYNE2,IL6R,CCR1,CCR2,CORO1A,TRIB1,CCR7,LEF1,GATA3,BCL2	myeloid cells/inflammation	ENSOARG00000016047,ENSOARG00000016133,ENSOARG00000019970,ENSOARG00000020341,ENSOARG00000010691,ENSOARG00000021147,ENSOARG00000002562,ENSOARG00000014260,ENSOARG00000014236,ENSOARG00000003604,ENSOARG00000008183,ENSOARG00000014777,ENSOARG00000008064,ENSOARG00000013941,ENSOARG00000006262	15	0		15
**M110**	axon guidance	MYH10,PTK2,DPYSL3,RRAS,COL5A1,COL1A2,COL1A1,RHOC,NRP1,SEMA6A	others	ENSOARG00000001617,ENSOARG00000003418,ENSOARG00000003603,ENSOARG00000013273,ENSOARG00000002129,ENSOARG00000001508,ENSOARG00000004871,ENSOARG00000017485,ENSOARG00000001863	9	1	RHOC	10

**M111.0**	viral sensing & immunity; IRF2 targets network (I)	TAP1,KYNU,TLR7,ZBP1,IFI35,LY86,THBS1,TNFSF13B,PSMB8,EREG,FES,BST2,USP18,CTSS,RIPK2,AIF1,CXCL16	IFN type I	ENSOARG00000007738,ENSOARG00000009938,ENSOARG00000011288,ENSOARG00000017418,ENSOARG00000004413,ENSOARG00000018347,ENSOARG00000020058,ENSOARG00000005617,ENSOARG00000007622,ENSOARG00000014953,ENSOARG00000012648,ENSOARG00000019272,ENSOARG00000020861,ENSOARG00000011197,ENSOARG00000007932, ENSOARG00000007683, ENSOARG00000007738,ENSOARG00000009938,ENSOARG00000011288,ENSOARG00000017418,ENSOARG00000004413,ENSOARG00000018347,ENSOARG00000020058,ENSOARG00000005617,ENSOARG00000007622,ENSOARG00000014953,ENSOARG00000012648,ENSOARG00000019272,ENSOARG00000020861,ENSOARG00000011197,ENSOARG00000007932, ENSOARG00000007683	16	1	BST2	17
**M111.1**	viral sensing & immunity; IRF2 targets network (II)	TAP1,DDX58,PSMB10,ZBP1,CASP1,PSMB8,BST2,CXCL10,USP18,IFIT2,TNFSF13B	IFN type I	ENSOARG00000007738,ENSOARG00000014731,ENSOARG00000003243,ENSOARG00000017418,ENSOARG00000007622,ENSOARG00000016611,ENSOARG00000019272,ENSOARG00000015169,ENSOARG00000005617, ENSOARG00000007738,ENSOARG00000014731,ENSOARG00000003243,ENSOARG00000017418,ENSOARG00000007622,ENSOARG00000016611,ENSOARG00000019272,ENSOARG00000015169,ENSOARG00000005617	9	2	CASP1,BST2	11
**M112.0**	complement activation (I)	CR1,CFH,CFI,SERPING1,C3AR1,CFD,C1QC,C1QB,C1QA,C3,CFB,C5AR1,C2,C1S,C1R,C7,A2M	myeloid cells/inflammation	ENSOARG00000018430,ENSOARG00000005291,ENSOARG00000010035,ENSOARG00000020341,ENSOARG00000009779,ENSOARG00000007991,ENSOARG00000007977,ENSOARG00000008001,ENSOARG00000011005,ENSOARG00000004658,ENSOARG00000004515,ENSOARG00000009128,ENSOARG00000000950	13	4	CR1,C3,CFB,C2	17
**M112.1**	complement activation (II)	CFH,CFI,PROS1,CFB,C3,IL33,CLU,C1S,C1R,C7	myeloid cells/inflammation	ENSOARG00000018430,ENSOARG00000005291,ENSOARG00000017273,ENSOARG00000013628,ENSOARG00000004658,ENSOARG00000004515,ENSOARG00000009128	7	3	CFB,C3,CLU	10
**M113**	golgi membrane (I)	B3GNT5,FAM198B,WLS,PDGFC,LPCAT2,SGMS2,SORT1,TLR8,STEAP4,CSGALNACT1	others	ENSOARG00000020655,ENSOARG00000006033,ENSOARG00000011383,ENSOARG00000006586,ENSOARG00000018091,ENSOARG00000008597,ENSOARG00000019184,ENSOARG00000018099,ENSOARG00000011811,ENSOARG00000003933	10	0		10
**M114.0**	TBA	ELANE,SVIL,DOCK5,PKP4,OSBPL1A,CLGN,MGST1,VSTM1,MS4A3,AKR1C3,LGALS12,KCNK5,RGL4,FAH,NFE2,ST3GAL6,JAG1,GGH,PDGFC,TAL1,CDK6,ATP8B4,ACTN1,CLEC11A,TSPAN2,CPNE2,IGFBP7,C5orf13,ACCN2,ZNRF1,SLC40A1,SYNGR1,MPO,RUNX2,MLC1,BST1,FAM105A,CPA3,BTBD3	others	ENSOARG00000009696,ENSOARG00000014369,ENSOARG00000009918,ENSOARG00000007629,ENSOARG00000007396,ENSOARG00000012717,ENSOARG00000020582,ENSOARG00000012607,ENSOARG00000002555,ENSOARG00000016548,ENSOARG00000015252,ENSOARG00000016228,ENSOARG00000017765,ENSOARG00000010523,ENSOARG00000016793, ENSOARG00000006586,ENSOARG00000003450,ENSOARG00000018572,ENSOARG00000020996,ENSOARG00000021179,ENSOARG00000020131,ENSOARG00000018900,ENSOARG00000005761,ENSOARG00000006385,ENSOARG00000016390,ENSOARG00000016513,ENSOARG00000009287,ENSOARG00000010358,ENSOARG00000019692,ENSOARG00000009499, ENSOARG00000013160,ENSOARG00000005274,ENSOARG00000010789	33	6	VSTM1,AKR1C3,RGL4,CLEC11A,C5orf13,ACCN2	39
**M114.1**	glycerophospholipid metabolism	SVIL,SLC40A1,OSBPL1A,MBOAT2,FAH,ST3GAL6,LPCAT2,IGFBP7,PLA2G4A,AGPAT9,AKR1C3	others	ENSOARG00000014369,ENSOARG00000016390,ENSOARG00000007396,ENSOARG00000014789,ENSOARG00000015252,ENSOARG00000017765,ENSOARG00000018091,ENSOARG00000005761,ENSOARG00000007935,ENSOARG00000003059	10	1	AKR1C3	11
**M115**	cytokines - recepters cluster	IL7R,IL2RA,LIF,IL23A,IFNG,IL15,CSF2,CSF3,IL6,IL10,IL15RA	myeloid cells/inflammation	ENSOARG00000011239,ENSOARG00000012860,ENSOARG00000006322,ENSOARG00000009246,ENSOARG00000001958,ENSOARG00000012119,ENSOARG00000015430,ENSOARG00000012606,ENSOARG00000012021,ENSOARG00000006292,ENSOARG00000012768, ENSOARG00000011239,ENSOARG00000012860,ENSOARG00000006322,ENSOARG00000009246,ENSOARG00000001958,ENSOARG00000012119,ENSOARG00000015430,ENSOARG00000012606,ENSOARG00000012021,ENSOARG00000006292,ENSOARG00000012768	11	0		11

**M116**	TBA	ERRFI1,PDGFRA,CYR61,WWTR1,FSTL1,YAP1,OSMR,VCAM1,EPS8,GPR116,COL3A1,CXCL12,CAV1	others	ENSOARG00000010072,ENSOARG00000019070,ENSOARG00000014336,ENSOARG00000004797,ENSOARG00000019900,ENSOARG00000006660,ENSOARG00000009845,ENSOARG00000020622,ENSOARG00000016476,ENSOARG00000002991,ENSOARG00000001337	11	2	VCAM1,GPR116	13
**M117**	cell adhesion (GO)	PPP2R1A,CD40LG,NELL2,CD96,NINJ1,PSTPIP1,LY9,ADAM9,CXCR3,CD6,CORO1A,PTPRC,ZAP70,SPOCK2,PRKCQ,SELPLG,CYTIP,CD300A,PLEK,LEF1,BCL2	others	ENSOARG00000015049,ENSOARG00000011076,ENSOARG00000019622,ENSOARG00000019179,ENSOARG00000008116,ENSOARG00000001650,ENSOARG00000008981,ENSOARG00000001816,ENSOARG00000017562,ENSOARG00000016062,ENSOARG00000003604,ENSOARG00000015718,ENSOARG00000013646,ENSOARG00000007029,ENSOARG00000013242,ENSOARG00000007774,ENSOARG00000019902,ENSOARG00000008064,ENSOARG00000006262	19	2	SELPLG,CD300A	21
**M118.0**	enriched in monocytes (IV)	PTGS2,EMR1,AMICA1,DOCK5,CD4,LY96,ARHGEF10L,TNFSF13B,LTBR,PGD,TNFRSF1B,LRRK2,DPYD,MGAM,PTX3,LYZ,IL1R2,DOK3,CARD9,EVI5,GPR109B,DUSP6,MYO1F,FGD4,HHEX,HAL,ST3GAL6,DYSF,RNASE6,SLC24A4,VNN1,NAIP,RHOU,CD68,CXCR2,NACC2,SMARCD3,PADI4,TMEM176B,LGALS3,SAMHD1,CTSS,EMILIN2,ACPP,F5,STEAP4,C19orf59,ACSL1,PAK1,C1orf162,MOSC1,TLR1,PID1,BCL6,HLA-DMB,MPP1,AGPAT9	myeloid cells/inflammation	ENSOARG00000007592,ENSOARG00000009918,ENSOARG00000006301,ENSOARG00000006376,ENSOARG00000005617,ENSOARG00000008343,ENSOARG00000005335,ENSOARG00000017205,ENSOARG00000019970,ENSOARG00000017549,ENSOARG00000014509,ENSOARG00000002754,ENSOARG00000020515,ENSOARG00000013159,ENSOARG00000004300,ENSOARG00000004519,ENSOARG00000016422,ENSOARG00000015628,ENSOARG00000004207,ENSOARG00000019356,ENSOARG00000012169,ENSOARG00000017765,ENSOARG00000011500,ENSOARG00000012752,ENSOARG00000012969,ENSOARG00000014440,ENSOARG00000005446,ENSOARG00000003025,ENSOARG00000014729,ENSOARG00000019477,ENSOARG00000004768,ENSOARG00000003916,ENSOARG00000011018,ENSOARG00000001691,ENSOARG00000021088,ENSOARG00000016807,ENSOARG00000020861,ENSOARG00000009973,ENSOARG00000009106,ENSOARG00000010135,ENSOARG00000011811,ENSOARG00000007614,ENSOARG00000007238,ENSOARG00000019610,ENSOARG00000000538,ENSOARG00000020593,ENSOARG00000020487,ENSOARG00000003150,ENSOARG00000003059	49	8	EMR1,AMICA1,ARHGEF10L,GPR109B,HHEX,C19orf59,MOSC1,HLA-DMB	57
**M118.1**	enriched in monocytes (surface)	LTBR,CD4,TNFRSF1B,MCTP1,S1PR3,EMR1,C19orf59,SLC24A4,STEAP4,C1orf162,SLC16A3,NFAM1,TMEM176B,HLA-DMB,IL1R2,AGPAT9,TNFSF13B	myeloid cells/inflammation	ENSOARG00000008343,ENSOARG00000006301,ENSOARG00000017205,ENSOARG00000016842,ENSOARG00000007690,ENSOARG00000012969,ENSOARG00000011811,ENSOARG00000019610,ENSOARG00000016912,ENSOARG00000018955,ENSOARG00000001691,ENSOARG00000013159,ENSOARG00000003059,ENSOARG00000005617	14	3	EMR1,C19orf59,HLA-DMB	17
**M119**	enriched in activated dendritic cells (I)	IL1RN,CXCL2,RGL1,CD83,PRKCD,CD86,RIN2,SLAMF8,IL4I1,ATP1B1,PLXDC2	DC/antigen presentation	ENSOARG00000020828,ENSOARG00000014775,ENSOARG00000002070,ENSOARG00000012936,ENSOARG00000000156,ENSOARG00000020112,ENSOARG00000001865,ENSOARG00000007944,ENSOARG00000013677,ENSOARG00000009371,ENSOARG00000000344	11	0		11
**M120**	TBA	DGKZ,MAP4K1,LPXN,VAV1,HCLS1,ARHGAP15,ARRB2,SIGIRR,ARHGAP4,LYN,GATA3	others	ENSOARG00000003287,ENSOARG00000005746,ENSOARG00000011101,ENSOARG00000004983,ENSOARG00000020050,ENSOARG00000009910,ENSOARG00000007797,ENSOARG00000007476,ENSOARG00000001509,ENSOARG00000014778,ENSOARG00000013941	11	0		11
**M121**	TBA	IGKV2D-26,IGKV,TAP1,IRF4,CCL19,GPR183,CD27,IL15,BIRC3,LY9,PLSCR1,SLAMF7,IGKC	others	ENSOARG00000020807,ENSOARG00000007738,ENSOARG00000003822,ENSOARG00000009119,ENSOARG00000017217,ENSOARG00000008332,ENSOARG00000012119,ENSOARG00000006356,ENSOARG00000008981,ENSOARG00000005557,ENSOARG00000008967, ENSOARG00000020807,ENSOARG00000007738,ENSOARG00000003822,ENSOARG00000009119,ENSOARG00000017217,ENSOARG00000008332,ENSOARG00000012119,ENSOARG00000006356,ENSOARG00000008981,ENSOARG00000005557,ENSOARG00000008967	11	2	IGKV,IGKC	13
**M122**	enriched for cell migration	CCL2,PODN,TGFB2,IGF1,LAMA4,NBL1,MYLK,NR2F2,IGFBP5,CXCL12,NRP1	others	ENSOARG00000009627,ENSOARG00000006083,ENSOARG00000011583,ENSOARG00000015856,ENSOARG00000009580,ENSOARG00000009799,ENSOARG00000020201,ENSOARG00000010167,ENSOARG00000019421,ENSOARG00000002991,ENSOARG00000017485	11	0		11

**M123**	enriched in B cell differentiation	ICOSLG,HBEGF,LOC144481,NR4A1,GAB1,DLL1,LOC100288911,NR4A3,EBF1,FAM150B,BCL11B,VPREB1,VPREB3	B cells	ENSOARG00000011738,ENSOARG00000017521,ENSOARG00000017214,ENSOARG00000010978,ENSOARG00000005108,ENSOARG00000010939,ENSOARG00000000266,ENSOARG00000012733	8	5	LOC144481,LOC100288911,EBF1,FAM150B,VPREB1	13
**M124**	enriched in membrane proteins	ABCG2,RHAG,LTF,AQP1,C3AR1,HP,OVAR-DRB1,ELANE,CD24,NEDD4L,ALAS2,C5AR1,SLC4A1,ABCB6,SNCA,PROK2,TGM2,SLC40A1,KCNH2	others	ENSOARG00000001914,ENSOARG00000012291,ENSOARG00000008620,ENSOARG00000007775,ENSOARG00000020341,ENSOARG00000009696,ENSOARG00000011081,ENSOARG00000005399,ENSOARG00000005674,ENSOARG00000011005,ENSOARG00000007821,ENSOARG00000019920,ENSOARG00000018510,ENSOARG00000018528,ENSOARG00000016390, ENSOARG00000001434, ENSOARG00000001914,ENSOARG00000012291,ENSOARG00000008620,ENSOARG00000007775,ENSOARG00000020341,ENSOARG00000009696,ENSOARG00000011081,ENSOARG00000005399,ENSOARG00000005674,ENSOARG00000011005,ENSOARG00000007821,ENSOARG00000019920,ENSOARG00000018510,ENSOARG00000018528,ENSOARG00000016390, ENSOARG00000001434	16	3	HP,OVAR-DRB1,PROK2	19
**M125**	TBA	CDKN2B,GPR144,ZNF205,ELAVL3,TCF15,SOX15,PRKCG,GP1BB,FUT7,PSD,MAPRE3	others	ENSOARG00000001319,ENSOARG00000018464,ENSOARG00000019015,ENSOARG00000014928,ENSOARG00000000873,ENSOARG00000000653,ENSOARG00000019040	7	4	CDKN2B,GPR144,GP1BB,FUT7	11
**M126**	double positive thymocytes	MGC16121,TCF4,UBASH3A,SLC40A1,SH2D1A,APBB1,BCL11B,CD4,CHI3L2,ASRGL1,PCSK5,LAT,SPNS1,CD3E	T/NK cells	ENSOARG00000005018,ENSOARG00000010806,ENSOARG00000016390,ENSOARG00000014085,ENSOARG00000018427,ENSOARG00000000266,ENSOARG00000006301,ENSOARG00000019517,ENSOARG00000014809,ENSOARG00000012515,ENSOARG00000001158,ENSOARG00000001386,ENSOARG00000008984	13	1	MGC16121	14
**M127**	type I interferon response	TAP1,IFIH1,IRF7,PARP9,STAT1,PLSCR1,IFITM1,HERC5,DDX60,USP18,RSAD2,IFIT1	IFN type I	ENSOARG00000007738,ENSOARG00000006142,ENSOARG00000006626,ENSOARG00000020150,ENSOARG00000013903,ENSOARG00000005557,ENSOARG00000008115,ENSOARG00000000530,ENSOARG00000019272,ENSOARG00000014648,ENSOARG00000015177, ENSOARG00000007738,ENSOARG00000006142,ENSOARG00000006626,ENSOARG00000020150,ENSOARG00000013903,ENSOARG00000005557,ENSOARG00000008115,ENSOARG00000000530,ENSOARG00000019272,ENSOARG00000014648,ENSOARG00000015177	11	1	DDX60	12
**M128**	TBA	SP3,NDUFA5,USP1,CCNC,PIK3C2A,SFRS12,CLK1,RNF6,ARID4A,CCNT2,PSMC6,RNF139	others	ENSOARG00000000469,ENSOARG00000009203,ENSOARG00000011800,ENSOARG00000004372,ENSOARG00000016541,ENSOARG00000012743,ENSOARG00000021110,ENSOARG00000011259,ENSOARG00000017558,ENSOARG00000009072	10	2	NDUFA5,SFRS12	12
**M129**	inositol phosphate metabolism	PANK3,AVL9,PIKFYVE,MED23,PLEKHF2,SRP9,IMPA1,PIK3C2A,SYNJ1,CASC4,FNDC3A,PIK3C3,KIAA1109	others	ENSOARG00000002732,ENSOARG00000006850,ENSOARG00000019028,ENSOARG00000013991,ENSOARG00000014688,ENSOARG00000017438,ENSOARG00000015101,ENSOARG00000004372,ENSOARG00000013414,ENSOARG00000003595,ENSOARG00000008377,ENSOARG00000005184,ENSOARG00000000316	13	0		13
**M130**	enriched in G-protein coupled receptors	CCL5,XCL1,PTGER2,GPR18,F2R,ADRB2,S1PR5,PTGDR,ITPR3,PDE4D	others	ENSOARG00000004964,ENSOARG00000009179,ENSOARG00000017673,ENSOARG00000017207,ENSOARG00000016937,ENSOARG00000014352,ENSOARG00000020714,ENSOARG00000010180,ENSOARG00000007210	9	1	S1PR5	10
**M131**	TBA	HEMGN,ACTN1,GFI1B,XK,CMPK2,RGL4,S100P,FHL2,INHBA,PRKAR2B,TAL1,TNS1,MS4A3,GMPR	others	ENSOARG00000011165,ENSOARG00000021179,ENSOARG00000005567,ENSOARG00000018927,ENSOARG00000014636,ENSOARG00000012948,ENSOARG00000017475,ENSOARG00000004957,ENSOARG00000003450,ENSOARG00000019446,ENSOARG00000012607,ENSOARG00000012220	12	2	RGL4,S100P	14
**M132**	recruitment of neutrophils	PROK2,FPR1,FPR2,GNG10,C5AR1,GPR109B,CXCR1,CCR3,PTAFR,FFAR2,F2RL1	myeloid cells/inflammation	ENSOARG00000002977,ENSOARG00000011005,ENSOARG00000019492,ENSOARG00000014244,ENSOARG00000019840,ENSOARG00000011266,ENSOARG00000011584	7	4	PROK2,FPR1,FPR2,GPR109B	11
**M133.0**	cell adhesion, membrane	SFN,SPINT1,BIK,EHF,JUP,LSR,EPS8L1,CDH1,EPCAM,CDH3,CLDN3,CLDN4,KRT19,CLDN7	others	ENSOARG00000018302,ENSOARG00000020293,ENSOARG00000019123,ENSOARG00000018677,ENSOARG00000017474,ENSOARG00000004736,ENSOARG00000001938,ENSOARG00000003455,ENSOARG00000005218,ENSOARG00000003438,ENSOARG00000010845,ENSOARG00000025184,ENSOARG00000011311	13	1	KRT19	14

**M133.1**	cell cell adhesion	LAMA3,ITGB4,ITGB6,LAMC2,JUP,CDH1,CDH3,CLDN3,CLDN4,LAMB3,CLDN7	others	ENSOARG00000007694,ENSOARG00000009764,ENSOARG00000006967,ENSOARG00000000744,ENSOARG00000017474,ENSOARG00000003455,ENSOARG00000003438,ENSOARG00000010845,ENSOARG00000025184,ENSOARG00000013143,ENSOARG00000011311	11	0		11
**M134**	Membrane, ER proteins	C10orf10,NAV1,MRVI1,PTK2,CD72,ENG,RGL1,CIITA,SH2D4B,RASAL2,LARGE,STK32B,EMP2,CORO2B,CSRP2,TMED6,DHCR24	others	ENSOARG00000003708,ENSOARG00000018937,ENSOARG00000010763,ENSOARG00000003418,ENSOARG00000011957,ENSOARG00000011387,ENSOARG00000002070,ENSOARG00000006106,ENSOARG00000010332,ENSOARG00000015973,ENSOARG00000018279,ENSOARG00000007185,ENSOARG00000005780,ENSOARG00000018517,ENSOARG00000014916, ENSOARG00000003552,ENSOARG00000007493	17	0		17
**M135.0**	enriched in plasma membrane proteins (I)	LRP2,ENPEP,GALNT14,ALPK2,SEMA5B,BBOX1,CUBN,PAX2,DGCR5,AGXT2,SLC6A13,LOC100130232,NHLH2,NAT8,NAT8B,GAL3ST1, SLC17A3	others	ENSOARG00000003592,ENSOARG00000003714,ENSOARG00000019676,ENSOARG00000005476,ENSOARG00000020173,ENSOARG00000014571,ENSOARG00000009955,ENSOARG00000015552,ENSOARG00000011462,ENSOARG00000011827,ENSOARG00000016499,ENSOARG00000001926	12	5	DGCR5,LOC100130232,NHLH2,NAT8B,GAL3ST1	17
**M135.1**	enriched in plasma membrane proteins (II)	ENPEP,GALNT14,ALPK2,KCNJ16,CUBN,SLC3A1,DGCR5,SLC17A3,SLC6A13,ESM1,COL23A1,NAT8,NAT8B,GAL3ST1,ACMSD,DSCAML1	others	ENSOARG00000003714,ENSOARG00000019676,ENSOARG00000000206,ENSOARG00000005476,ENSOARG00000009955,ENSOARG00000006308,ENSOARG00000001926,ENSOARG00000011827,ENSOARG00000008037,ENSOARG00000006533,ENSOARG00000016499,ENSOARG00000011350,ENSOARG00000006595	13	3	DGCR5,NAT8B,GAL3ST1	16
**M136**	TBA	NUCB1,GPS1,ASNA1,COPE,PKN1,GNAI2,CTSD,GNB2,PPP2R1A,MAP2K2,NAPA,CAPN1,DNM2,FAM108A1,GBF1,NR1H2,PNPLA6	others	ENSOARG00000012427,ENSOARG00000017353,ENSOARG00000011392,ENSOARG00000010422,ENSOARG00000005558,ENSOARG00000010164,ENSOARG00000016272,ENSOARG00000015049,ENSOARG00000010782,ENSOARG00000011245,ENSOARG00000013465,ENSOARG00000017183,ENSOARG00000017794,ENSOARG00000013898,ENSOARG00000001448	15	2	CTSD,FAM108A1	17
**M137**	TBA	ZMYM2,PHTF2,PANK3,AGL,TLK1,TSNAX,PIKFYVE,USP1,PLEKHF2,TCERG1,ROCK1,ACBD3,SFRS12,RNF6,SMARCA5,ATF2	others	ENSOARG00000014028,ENSOARG00000017915,ENSOARG00000002732,ENSOARG00000017762,ENSOARG00000002212,ENSOARG00000019028,ENSOARG00000009203,ENSOARG00000014688,ENSOARG00000008819,ENSOARG00000000040,ENSOARG00000012743,ENSOARG00000010589,ENSOARG00000000194	13	3	TSNAX,TCERG1,SFRS12	16
**M138**	enriched for ubiquitination	PHTF2,UBE2Q2,RPS6KB1,NRAS,SRP9,ZBTB41,PIK3C2A,USP1,RNF6,MBNL2,UBR1	DC/antigen presentation	ENSOARG00000017915,ENSOARG00000001259,ENSOARG00000014401,ENSOARG00000020027,ENSOARG00000017438,ENSOARG00000015070,ENSOARG00000004372,ENSOARG00000009203,ENSOARG00000012743,ENSOARG00000002685,ENSOARG00000020579	11	0		11
**M139**	lysosomal/endosomal proteins	CTSH,SLC11A1,PSAP,CTSB,CTSD,CD68,NPC2,AP1S2,SORT1,CTSS,GAA	DC/antigen presentation	ENSOARG00000014664,ENSOARG00000019535,ENSOARG00000006957,ENSOARG00000015263,ENSOARG00000014729,ENSOARG00000001076,ENSOARG00000012817,ENSOARG00000019184,ENSOARG00000020861,ENSOARG00000002285	10	1	CTSD	11
**M140**	extracellular matrix, complement	SLPI,CRISPLD2,FCN1,LTBP2,PRTN3,LAMA2,TFF3,FLRT2,IGFBP7,CRISP3,MMP9,MMP8,CFP,TIMP2	others	ENSOARG00000004969,ENSOARG00000011051,ENSOARG00000001170,ENSOARG00000009685,ENSOARG00000013616,ENSOARG00000010659,ENSOARG00000017560,ENSOARG00000005761,ENSOARG00000012520,ENSOARG00000007908,ENSOARG00000005480,ENSOARG00000012877,ENSOARG00000003264	13	1	FCN1	14
**M141**	TBA	TRIM31,FABP1,FUT4,NOX1,C19orf21,EPCAM,PRR15L,B3GNT5,B3GNT3,CEACAM1,KLF5,GMDS,CEACAM6,TMEM45B,ST3GAL6,TMPRSS2,CKMT1A,CKMT1B,FERMT1,HEPH,LGALS4,PIP5K1B,FA2H,NR1I2,FOXA3,GPX2,FUT3,MEP1A	others	ENSOARG00000016280,ENSOARG00000020752,ENSOARG00000009829,ENSOARG00000001070,ENSOARG00000005218,ENSOARG00000004462,ENSOARG00000020655,ENSOARG00000015019,ENSOARG00000015505,ENSOARG00000002773,ENSOARG00000014210,ENSOARG00000017765,ENSOARG00000010334,ENSOARG00000010485,ENSOARG00000018536, ENSOARG00000005331,ENSOARG00000005839,ENSOARG00000013075,ENSOARG00000006220,ENSOARG00000019817,ENSOARG00000021156,ENSOARG00000011224	22	6	C19orf21,CEACAM1,CEACAM6,CKMT1B,FOXA3,FUT3	28
**M142**	transmembrane and ion transporters (I)	ASPA,NLGN1,CUBN,SLC13A1,SLC28A1,SLC17A1,LOC100422737, SLC17A3,LOC100130232,PAX2,SLC6A13,KCNIP4,SEMA5B	others	ENSOARG00000016806,ENSOARG00000020726,ENSOARG00000009955,ENSOARG00000000430,ENSOARG00000013352,ENSOARG00000002375,ENSOARG00000001926,ENSOARG00000015552,ENSOARG00000011827,ENSOARG00000005149,ENSOARG00000020173	11	2	LOC100422737,LOC100130232	13

**M143**	nuclear pore, transport; mRNA splicing, processing	SLBP,NCBP2,NUP133,NUP107,NUP160,NUP54,UPF3B,NUP155,SEH1L,NUPL2,NUP205	others	ENSOARG00000016090,ENSOARG00000020367,ENSOARG00000003060,ENSOARG00000000362,ENSOARG00000007967,ENSOARG00000016734,ENSOARG00000016051,ENSOARG00000010255,ENSOARG00000012602,ENSOARG00000009081	10	1	SEH1L	11
**M144**	cell cycle, ATP binding	RBM7,NEK7,UBA3,HDAC2,TLK1,MCTS1,RAD21,COPS5,PCNP,PSMA6,PPP1R12A,VPS4B,CCNC,CUL5,PSMC6,ERBB2IP	cell cycle	ENSOARG00000015482,ENSOARG00000010086,ENSOARG00000009493,ENSOARG00000002212,ENSOARG00000013384,ENSOARG00000010359,ENSOARG00000000428,ENSOARG00000007429,ENSOARG00000015054,ENSOARG00000006303,ENSOARG00000011800,ENSOARG00000010875,ENSOARG00000017558,ENSOARG00000006023	14	2	RBM7,PCNP	16
**M145.0**	cytoskeleton/actin (SRF transcription targets)	ACTA2,MYH11,MRVI1,SDPR,SVIL,ACTG2,CALD1,MYLK,MRGPRF,TPM2,PALLD,TPM1,EPS8,PLN,CAV2,TAGLN	others	ENSOARG00000014814,ENSOARG00000008975,ENSOARG00000010763,ENSOARG00000014183,ENSOARG00000014369,ENSOARG00000012108,ENSOARG00000007819,ENSOARG00000020201,ENSOARG00000017116,ENSOARG00000011889,ENSOARG00000015569,ENSOARG00000020797,ENSOARG00000020622,ENSOARG0000019971,ENSOARG0000001379,ENSOARG00000004174	16	0		16
**M145.1**	cytoskeleton/actin (SRF transcription targets)	ACTA2,MYH11,ACTG2,LMOD1,CALD1,MYLK,TPM1,TPM2,SYNPO2,SORBS1,TNS1,CAV2,VCL,MYL9	others	ENSOARG00000014814,ENSOARG00000008975,ENSOARG00000012108,ENSOARG00000000119,ENSOARG00000007819,ENSOARG00000020201,ENSOARG00000020797,ENSOARG00000011889,ENSOARG00000016878,ENSOARG00000006055,ENSOARG00000019446,ENSOARG00000001379,ENSOARG00000008576,ENSOARG00000015847	14	0		14
**M146**	MHC-TLR7-TLR8 cluster	CD74,CD4,HLA-E,TLR8,TLR7,DQA,HLA-DOA,HLA-DOB,HLA-DMA,HLA-DRA,OVAR-DM,OVAR-DQA1,OVAR-DQA2,OVAR-DQB1,OVAR-DQB2,OVAR-DRB1,OVAR-DRB5,OVAR-DRB3,OVAR-A, OVAR-B, OVAR-C	DC/antigen presentation	ENSOARG00000007229,ENSOARG00000006301,ENSOARG00000018099,ENSOARG00000011288,ENSOARG00000015485,ENSOARG00000008091,ENSOARG00000007458,ENSOARG00000007972,ENSOARG00000016610	9	12	HLA-E,OVAR-DM,OVAR-DQA1,OVAR-DQA2,OVAR-DQB1,OVAR-DQB2,OVAR-DRB1,OVAR-DRB5,OVAR-DRB3,OVAR-A, OVAR-B, OVAR-C	21
**M147**	intracellular transport	SIRT1,EXOC1,VPS4B,NUP133,NUP107,CEP120,CLINT1,ZFYVE16,PIKFYVE,SEC63,ZFAND6,PIK3C2A,TMX1,PPP1R12A,RAD21,G3BP2,SRP9	others	ENSOARG00000004826,ENSOARG00000002363,ENSOARG00000006303,ENSOARG00000003060,ENSOARG00000000362,ENSOARG00000018810,ENSOARG00000012788,ENSOARG00000014668,ENSOARG00000019028,ENSOARG00000010911,ENSOARG00000004372,ENSOARG00000020695,ENSOARG00000015054,ENSOARG00000010359,ENSOARG00000015652,ENSOARG00000017438	16	1	ZFAND6	17
**M148**	TBA	MORC3,SEC23A,SPAST,EEA1,NUP160,RB1,ADAM9,STXBP3,SYNJ1,SEC24B,TMEM30A	others	ENSOARG00000013952,ENSOARG00000008413,ENSOARG00000010758,ENSOARG00000015811,ENSOARG00000007967,ENSOARG00000008246,ENSOARG00000001816,ENSOARG00000018946,ENSOARG00000013414,ENSOARG00000006459,ENSOARG00000006481	11	0		11
**M149**	TBA	GREB1,SLC3A1,SIX4,HNF4G,RNF186,CLRN3,USH1C,CLDN2,GIPC2,TFAP2C	others	ENSOARG00000016103,ENSOARG00000006308,ENSOARG00000021130,ENSOARG00000007140,ENSOARG00000009608,ENSOARG00000014062,ENSOARG00000003142,ENSOARG00000018869,ENSOARG00000013390,ENSOARG00000018105	10	0		10
**M150**	innate antiviral response	TAP1,IRF7,PLSCR1,EIF2AK2,APOBEC3A,OASL,RXRA,OAS1,OAS3,RSAD2,IFIT1,SP100	IFN type I	ENSOARG00000007738,ENSOARG00000006626,ENSOARG00000005557,ENSOARG00000009740,ENSOARG00000013935,ENSOARG00000002366,ENSOARG00000002881,ENSOARG00000014648,ENSOARG00000015177,ENSOARG00000020656, ENSOARG00000007738,ENSOARG00000006626,ENSOARG00000005557,ENSOARG00000009740,ENSOARG00000013935,ENSOARG00000002366,ENSOARG00000002881,ENSOARG00000014648,ENSOARG00000015177,ENSOARG00000020656	10	2	APOBEC3A,OAS3	12
**M151**	TBA	PRKCH,ZNF521,GREM1,TIAM1,SMAD1,RORA,BCL11B,INHBA,ADAM12,HHIP	others	ENSOARG00000021134,ENSOARG00000007246,ENSOARG00000014016,ENSOARG00000009089,ENSOARG00000020816,ENSOARG00000000266,ENSOARG00000017475,ENSOARG00000012241,ENSOARG00000010036	9	1	GREM1	10

**M152.0**	TBA (source: B cells)	HTN1,CCR9,ST18,TAT,GAGE1,LOC100287445,IFNA10,IFNA16,TRDN,FAM123A,HERC2P7,PPEF2,POU4F2,TRIM49,GABRB1,ZPBP,LOC100271840,SYCP1,IMPG2,KCTD16,TMEFF2,LOC51152,MEFV,BAGE,LOC100131298	others	ENSOARG00000009514,ENSOARG00000013407,ENSOARG00000003890,ENSOARG00000008072,ENSOARG00000015945,ENSOARG00000015669,ENSOARG00000015154,ENSOARG00000020082,ENSOARG00000018281,ENSOARG00000014301,ENSOARG00000014195,ENSOARG00000001557	12	13	HTN1,GAGE1,LOC100287445,IFNA10,IFNA16,FAM123A,HERC2P7,POU4F2,TRIM49,LOC100271840,LOC51152,BAGE,LOC100131298	25
**M152.1**	TBA (source: naive B cells)	POU4F2,TAT,FAM123A,OR7E24,IMPG2,KCTD16,HERC2P7,LRRC37B2,TMEFF2,ANKRD20A1,LOC100286993,LOC100271840,LOC100132247,GTF2A1L,MEFV,TRIM49,ADAMTS20,LOC100286895,TPTE,IFNA10,LOC100131298	others	ENSOARG00000003890,ENSOARG00000016408,ENSOARG00000018281,ENSOARG00000014301,ENSOARG00000014195,ENSOARG00000004567,ENSOARG00000001557,ENSOARG00000019717	8	13	POU4F2,FAM123A,HERC2P7,LRRC37B2,ANKRD20A1,LOC100286993,LOC100271840,LOC100132247,TRIM49,LOC100286895,TPTE,IFNA10,LOC100131298	21
**M152.2**	TBA (source: memory B cells)	TAT,FAM123A,C14orf91,HERC2P7,TMEFF2,HTN1,PPEF2,MEFV,LOC51152,LOC100286993,GYPA,BAGE,ADAMTS20,GABRB1,CYP4A22,IFNA10,ZPBP,LOC100271840	others	ENSOARG00000003890,ENSOARG00000014195,ENSOARG00000015945,ENSOARG00000001557,ENSOARG00000019717,ENSOARG00000015669,ENSOARG00000015154	7	11	FAM123A,C14orf91,HERC2P7,HTN1,LOC51152,LOC100286993,GYPA,BAGE,CYP4A22,IFNA10,LOC100271840	18
**M153**	TBA	PHTF2,SP3,SEC23A,RAP2C,SGTB,AEBP2,NRAS,SRP9,C1D,PIK3C2A,CCNC,RNF6,FAM18B,C2orf69,RANBP6,TCF12	others	ENSOARG00000017915,ENSOARG00000000469,ENSOARG00000008413,ENSOARG00000012473,ENSOARG00000006113,ENSOARG00000020470,ENSOARG00000020027,ENSOARG00000017438,ENSOARG00000019931,ENSOARG00000004372,ENSOARG00000011800,ENSOARG00000012743,ENSOARG00000015837,ENSOARG00000020872	14	2	FAM18B,RANBP6	16
**M154.0**	amino acid metabolism and transport	FXYD2,BBOX1,NAT8,NAT8B,AGXT2,BHMT2,DMGDH,GAL3ST1,BHMT,SLC7A9,ACSM5,ACSM2A,ACSM2B, LOC100291873,DPYS,LOC100130232,NR1H4,SLC6A12,GBA3,PDZK1,NAT8,SLCO4C1,ACSM2A,SLC6A13,RBP5,AGMAT,ACMSD,ACSM2A, ACSM2B,SLC3A1,TMEM27,SLC17A1,LOC100422737,SLC17A3,C14orf105,GLYAT,SLC22A2	others	ENSOARG00000007027,ENSOARG00000014571,ENSOARG00000016499,ENSOARG00000011462,ENSOARG00000017231,ENSOARG00000017209,ENSOARG00000017250,ENSOARG00000004279,ENSOARG00000012681,ENSOARG00000012794,ENSOARG00000015650,ENSOARG00000013901,ENSOARG00000011751,ENSOARG00000005505,ENSOARG00000020568, ENSOARG00000016499,ENSOARG00000018280,ENSOARG00000011827,ENSOARG00000004288,ENSOARG00000009382,ENSOARG00000011350,ENSOARG00000006308,ENSOARG00000012651,ENSOARG00000002375,ENSOARG00000001926,ENSOARG00000021105,ENSOARG00000004692	27	10	NAT8B,GAL3ST1,ACSM2A, LOC100291873,LOC100130232,ACSM2A,ACSM2A, ACSM2B,LOC100422737,GLYAT	37
**M154.1**	transmembrane transport (SLC cluster)	SLC22A11,FXYD2,NLGN1,SLC13A1,SLC2A2,SLC28A1,SLC7A9,SLC16A4,SLC17A1,SLC5A10,SLC22A2,SLC3A1,SLC6A13,SLC6A12,SLC17A3,SLCO4C1,PDZK1	others	ENSOARG00000007865,ENSOARG00000007027,ENSOARG00000020726,ENSOARG00000000430,ENSOARG00000020765,ENSOARG00000013352,ENSOARG00000004279,ENSOARG00000019433,ENSOARG00000002375,ENSOARG00000018414,ENSOARG00000004692,ENSOARG00000006308,ENSOARG00000011827,ENSOARG00000011751,ENSOARG00000001926, ENSOARG00000018280,ENSOARG00000020568	17	0		17
**M155**	G protein coupled receptors cluster	S1PR3,C3AR1,FPR2,PTH2R,PTGER2,LTB4R,PTAFR,FPR1,P2RY2,PTGDR	others	ENSOARG00000007690,ENSOARG00000020341,ENSOARG00000019075,ENSOARG00000017673,ENSOARG00000019112,ENSOARG00000019840,ENSOARG00000020714	7	3	FPR2,FPR1,P2RY2	10

**M156.0**	plasma cells & B cells, immunoglobulins	CD52,C13orf18,VPREB3,ITM2C,P2RX5,CPNE5,ADAM28,STAP1,ABCB4,FCRL1,FCRL2,PCDH9,CD22,SPIB,IGKV3-20,BLK,IGJ,OSBPL10,CYAT1,E2F5,TNFRSF17,GNG7,MGC29506,IGHM,IGKV2D-26,IGKV,IGHA1,IGHE,IGHG,IGHD,IGLV,IGLC	B cells	ENSOARG00000004803,ENSOARG00000012733,ENSOARG00000020673,ENSOARG00000017481,ENSOARG00000013791,ENSOARG00000010026,ENSOARG00000007050,ENSOARG00000006960,ENSOARG00000007086,ENSOARG00000014957,ENSOARG00000004796,ENSOARG00000015215,ENSOARG00000014924,ENSOARG00000013715,ENSOARG00000013638,ENSOARG00000002846,ENSOARG00000020807,ENSOARG00000008862,ENSOARG00000008994,ENSOARG00000009269, ENSOARG00000004803,ENSOARG00000012733,ENSOARG00000020673,ENSOARG00000017481,ENSOARG00000013791,ENSOARG00000010026,ENSOARG00000007050,ENSOARG00000006960,ENSOARG00000007086,ENSOARG00000014957,ENSOARG00000004796,ENSOARG00000015215,ENSOARG00000014924,ENSOARG00000013715,ENSOARG00000013638,ENSOARG00000002846,ENSOARG00000020807,ENSOARG00000008862,ENSOARG00000008994,ENSOARG00000009269	20	12	C13orf18,ABCB4,SPIB,IGKV3-20,IGJ,CYAT1,TNFRSF17,MGC29506,IGKV,IGHG,IGLV,IGLC	32
**M156.1**	plasma cells, immunoglobulins	TNFRSF17,TXNDC5,DERL3,MUTED,CD27,PNOC,CYAT1,POU2AF1,MGC29506,IGHM,IGKV2D-26,IGKV,IGHA1,IGHE,IGHG,IGHD,IGLV,IGLC	B cells	ENSOARG00000017127,ENSOARG00000008332,ENSOARG00000014922,ENSOARG00000013870,ENSOARG00000002846,ENSOARG00000020807,ENSOARG00000008862,ENSOARG00000008994,ENSOARG00000009269, ENSOARG00000017127,ENSOARG00000008332,ENSOARG00000014922,ENSOARG00000013870,ENSOARG00000002846,ENSOARG00000020807,ENSOARG00000008862,ENSOARG00000008994,ENSOARG00000009269	9	9	TNFRSF17,DERL3,MUTED,CYAT1,MGC29506,IGKV,IGHG,IGLV,IGLC	18
**M157**	enriched in NK cells (III)	KLRC3,PPP1R16B,RASGRP1,CCL4,CX3CR1,GPR18,TIGIT,CD7,C1orf21,PVRIG,GIMAP6,TRGC2,MATK,PTPN4	Tc/NK	ENSOARG00000000405,ENSOARG00000020044,ENSOARG00000004253,ENSOARG00000014010,ENSOARG00000017207,ENSOARG00000019616,ENSOARG00000016754,ENSOARG00000002600,ENSOARG00000017076,ENSOARG00000001356,ENSOARG00000001797,ENSOARG00000011674,ENSOARG00000012857, ENSOARG00000000405,ENSOARG00000020044,ENSOARG00000004253,ENSOARG00000014010,ENSOARG00000017207,ENSOARG00000019616,ENSOARG00000016754,ENSOARG00000002600,ENSOARG00000017076,ENSOARG00000001356,ENSOARG00000017970,ENSOARG00000011674,ENSOARG00000012857	13	1	KLRC3	14
**M158.0**	interferon alpha response (I)	LHCGR,COL8A1,IMPG2,ITGB4,MMP12,TNR,SFN,LAMC2,ST14,ADAMTS20,FGF5,IFN-ALPHA,IFN-BETA	IFN type I	ENSOARG00000017875,ENSOARG00000018281,ENSOARG00000009764,ENSOARG00000004930,ENSOARG00000014202,ENSOARG00000018302,ENSOARG00000000744,ENSOARG00000014848,ENSOARG00000019717,ENSOARG00000018999	10	3	LHCGR,IFN-ALPHA,IFN-BETA	13
**M158.1**	interferon alpha response (II)	LHCGR,FAM123A,PRL,ADAMTS20,IFN-ALPHA,IFN-BETA	IFN type I	ENSOARG00000009137,ENSOARG00000019717	2	4	LHCGR,FAM123A,IFN-ALPHA,IFN-BETA	6
**M159**	G protein mediated calcium signaling	PLCB1,GNAQ,CAMK4,GNAZ,ADCY9,GNA15,GNG11,PRKAR2B,CALM3,GNAI1	others	ENSOARG00000009195,ENSOARG00000012393,ENSOARG00000000184,ENSOARG00000002932,ENSOARG00000012997,ENSOARG00000001101,ENSOARG00000004957,ENSOARG00000005297,ENSOARG00000017377	9	1	GNAZ	10
**M160**	leukocyte differentiation	KLF13,RARA,LIF,NAA15,BTG2,EGR2,CD83,JUNB,ADAM8,ID1,KLF6,MED1,NR4A2,IL1A,NFKB2,BCL3	others	ENSOARG00000016409,ENSOARG00000014119,ENSOARG00000006322,ENSOARG00000013690,ENSOARG00000001574,ENSOARG00000004489,ENSOARG00000012936,ENSOARG00000010854,ENSOARG00000011855,ENSOARG00000001111,ENSOARG00000012925,ENSOARG00000011074,ENSOARG00000007984,ENSOARG00000020877,ENSOARG00000000483, ENSOARG00000009239	16	0		16
**M161**	TBA	BCL2L1,SP3,TSNAX,SPAST,SLC7A5,RB1,RBM38,HERC4,RNF6,FNDC3A,MBNL3,ATF1,KIAA1109,BNIP2	others	ENSOARG00000001262,ENSOARG00000000469,ENSOARG00000010758,ENSOARG00000012129,ENSOARG00000008246,ENSOARG00000017883,ENSOARG00000004840,ENSOARG00000012743,ENSOARG00000008377,ENSOARG00000012405,ENSOARG00000017519,ENSOARG00000000316,ENSOARG00000020826	13	1	TSNAX	14

**M162.0**	plasma membrane, cell junction	TMC4,TJP3,SPINT1,LLGL2,GRHL2,PRR15L,MYH14,MAL2,ST14,EHF,ESRP1,PKP3,CDH1,EPCAM,ELF3,CLDN3,CLDN4,CLDN7	others	ENSOARG00000002868,ENSOARG00000011941,ENSOARG00000020293,ENSOARG00000010325,ENSOARG00000018325,ENSOARG00000004462,ENSOARG00000013801,ENSOARG00000009525,ENSOARG00000014848,ENSOARG00000018677,ENSOARG00000007042,ENSOARG00000007888,ENSOARG00000003455,ENSOARG00000005218,ENSOARG00000000544, ENSOARG00000010845,ENSOARG00000025184,ENSOARG00000011311	18	0		18
**M162.1**	cell junction	BSPRY,EPB41L5,TJP3,DSP,PKP3,CDH1,EPCAM,CLDN3,CLDN4,S100A14,CLDN7	others	ENSOARG00000006287,ENSOARG00000012888,ENSOARG00000011941,ENSOARG00000017552,ENSOARG00000007888,ENSOARG00000003455,ENSOARG00000005218,ENSOARG00000010845,ENSOARG00000025184,ENSOARG00000002406,ENSOARG00000011311	11	0		11
**M163**	enriched in neutrophils (II)	NPL,ARAP3,HSPA6,TYROBP,CFD,IMPA2,DENND3,BTNL8,FRAT2,MBOAT7,TSPAN2,BEST1,FLJ10357,SLC40A1	myeloid cells/inflammation	ENSOARG00000001160,ENSOARG00000007452,ENSOARG00000005167,ENSOARG00000009779,ENSOARG00000001725,ENSOARG00000003114,ENSOARG00000014905,ENSOARG00000002806,ENSOARG00000020131,ENSOARG00000014494,ENSOARG00000016390	11	3	NPL,BTNL8,FLJ10357	14
**M164**	xenobiotic metabolism	LRRC19,HHLA2,SLC3A1,UGT2A3,DPYS,UPB1,CYP3A7,GIPC2,CYP3A5,C19orf77	others	ENSOARG00000014554,ENSOARG00000018906,ENSOARG00000006308,ENSOARG00000009742,ENSOARG00000015650,ENSOARG00000014074,ENSOARG00000013390	7	3	CYP3A7,CYP3A5,C19orf77	10
**M165**	enriched in activated dendritic cells (II)	CSF2RB,IFIH1,CSF2RA,TNFAIP6,HESX1,CCL8,MX2,DOCK4,PDGFRL,MGST1,IFNGR2,IFIT3,IFIT2,IFIT1,HLX,SERPING1,RSAD2,CCL20,ST3GAL6,RBM47,IFI27,DAPK1,PRRG4,PDGFC,PARP9,LAMP3,CXCL11,DSE,IL18,BCL2A1,FAM20C,PRKAR2B,HERC5,SIGLEC1,ATF3	DC/antigen presentation	ENSOARG00000019095,ENSOARG00000006142,ENSOARG00000007541,ENSOARG00000009019,ENSOARG00000014637,ENSOARG00000009241,ENSOARG00000010231,ENSOARG00000002931,ENSOARG00000009217,ENSOARG00000020582,ENSOARG00000013168,ENSOARG00000014800,ENSOARG00000015169,ENSOARG00000015177,ENSOARG00000013206, ENSOARG00000010035,ENSOARG00000014648,ENSOARG00000020576,ENSOARG00000017765,ENSOARG00000012711,ENSOARG00000014451,ENSOARG00000008706,ENSOARG00000016840,ENSOARG00000006586,ENSOARG00000020150,ENSOARG00000020657,ENSOARG00000016668,ENSOARG00000009347,ENSOARG00000016193,ENSOARG00000015167, ENSOARG00000006178,ENSOARG00000004957,ENSOARG00000000530,ENSOARG00000002007,ENSOARG00000010493, ENSOARG00000019095,ENSOARG00000006142,ENSOARG00000007541,ENSOARG00000009019,ENSOARG00000014637,ENSOARG00000009241,ENSOARG00000010231,ENSOARG00000002931,ENSOARG00000009217,ENSOARG00000020582,ENSOARG00000013168,ENSOARG00000014800,ENSOARG00000015169,ENSOARG00000015177,ENSOARG00000013206, ENSOARG00000010035,ENSOARG00000014648,ENSOARG00000020576,ENSOARG00000017765,ENSOARG00000012711,ENSOARG00000014451,ENSOARG00000008706,ENSOARG00000016840,ENSOARG00000006586,ENSOARG00000020150,ENSOARG00000020657,ENSOARG00000016668,ENSOARG00000009347,ENSOARG00000016193,ENSOARG00000015167, [illegible]	35	0		35
**M166**	TBA	FMO3,PDGFRA,AMOTL2,GHR,PDGFRB,OLFML3,CHRDL1,ECM2,CHL1,NNMT,GFRA1,TNC,C1S,IGF2,INS-IGF2,C7,OSMR,ABCA8	others	ENSOARG00000012259,ENSOARG00000019070,ENSOARG00000008268,ENSOARG00000008837,ENSOARG00000006538,ENSOARG00000019942,ENSOARG00000017833,ENSOARG00000008233,ENSOARG00000009067,ENSOARG00000016179,ENSOARG00000005941,ENSOARG00000004658,ENSOARG00000003586,ENSOARG00000009128,ENSOARG00000009845	15	3	NNMT,INS-IGF2,ABCA8	18
**M167**	enriched in cell cycle	BCL2L1,PPP2R1A,GSPT1,EHMT2,SESN3,MAD1L1,CDC34,TGFB1,PSMD3,AKT1,DNM2,FLNA,TSC2,RBM38,STRADB	cell cycle	ENSOARG00000001262,ENSOARG00000015049,ENSOARG00000007088,ENSOARG00000003417,ENSOARG00000009085,ENSOARG00000008672,ENSOARG00000007468,ENSOARG00000012513,ENSOARG00000017183,ENSOARG00000005208,ENSOARG00000017283,ENSOARG00000017883,ENSOARG00000017141	13	2	MAD1L1,AKT1	15
**M168**	enriched in dendritic cells	CLEC10A,B3GNT5,PGD,JAG1,FCER1A,EMILIN2,CLEC5A,CA2,CTSB,FAM20C,ADAM9,RRAGD,TACSTD2,CTSL1,IL1R1,GGTA1,DAB2,CAPG,AGPAT9	DC/antigen presentation	ENSOARG00000009867,ENSOARG00000020655,ENSOARG00000005335,ENSOARG00000010523,ENSOARG00000007787,ENSOARG00000009973,ENSOARG00000014270,ENSOARG00000012954,ENSOARG00000015263,ENSOARG00000006178,ENSOARG00000001816,ENSOARG00000012738,ENSOARG00000008349,ENSOARG00000013144,ENSOARG00000009431, ENSOARG00000020511,ENSOARG00000003059	17	2	CTSL1,GGTA1	19

**M169**	mitosis (TF motif CCAATNNSNNNGCG)	PCNA,SMC1A,TMPO,ORC4L,NOC3L,CASP8AP2,XPO1,CETN3,NUP37,UPF3B,ORC3L,NIF3L1,PMS1,ACTR6,ARMC1,ACTL6A	cell cycle	ENSOARG00000017133,ENSOARG00000008581,ENSOARG00000012899,ENSOARG00000003978,ENSOARG00000012409,ENSOARG00000020174,ENSOARG00000016247,ENSOARG00000015613,ENSOARG00000016051,ENSOARG00000016650,ENSOARG00000016301,ENSOARG00000013563,ENSOARG00000017043,ENSOARG00000020696	14	2	ORC4L,ORC3L	16
**M170**	TBA	LPHN2,GHR,EFEMP1,ECM2,GNG12,ELTD1,IL33,VGLL3,CHL1,GPR116,MPDZ,ABCA8	others	ENSOARG00000008837,ENSOARG00000002053,ENSOARG00000008233,ENSOARG00000011369,ENSOARG00000013628,ENSOARG00000016976,ENSOARG00000009067,ENSOARG00000013861	8	4	LPHN2,ELTD1,GPR116,ABCA8	12
**M171**	heme biosynthesis (I)	HMBS,HEMGN,AHSP,ALAD,E2F2,CPOX,ALAS2,TMOD1,PLEK2,FECH,GATA1	others	ENSOARG00000012985,ENSOARG00000011165,ENSOARG00000009793,ENSOARG00000006271,ENSOARG00000007334,ENSOARG00000017706,ENSOARG00000005674,ENSOARG00000011271,ENSOARG00000021167,ENSOARG00000005202,ENSOARG00000012017	11	0		11
**M172**	enriched for TF motif TTCNRGNNNNTTC	CXCL3,EGR3,FOSB,GJB2,PLAU,NRIP3,NR4A3,EDN1,AG2,PHLDA2	others	ENSOARG00000014841,ENSOARG00000010199,ENSOARG00000009983,ENSOARG00000017089,ENSOARG00000008473,ENSOARG00000013951,ENSOARG00000010939,ENSOARG00000014073	8	2	AG2,PHLDA2	10
**M173**	erythrocyte differentiation	ETV1,FHL2,NTF3,DMD,KLF1,BPGM,TRIM10,EPB42,MYH10,HMBS,FEV,GFRA3,LYL1,GATA1	others	ENSOARG00000007763,ENSOARG00000012948,ENSOARG00000009046,ENSOARG00000018256,ENSOARG00000010245,ENSOARG00000007716,ENSOARG00000016038,ENSOARG00000020591,ENSOARG00000001617,ENSOARG00000012985,ENSOARG00000019766,ENSOARG00000016086,ENSOARG00000009454,ENSOARG00000012017	14	0		14
**M174**	TBA	TSN,MUDENG,SRP9,SEC63,ANP32E,MATR3,EED,SMARCA5,PANK3,SACM1L,RNF138,CUL5,MORF4L2,ZBED5,MDFIC,BMI1,PCNP,TMX1,ZBTB11,TNFAIP8,SEP15,CCNC,RNF6,BZW1	others	ENSOARG00000013339,ENSOARG00000017438,ENSOARG00000010911,ENSOARG00000020772,ENSOARG00000016931,ENSOARG00000004477,ENSOARG00000010589,ENSOARG00000002732,ENSOARG00000010162,ENSOARG00000006124,ENSOARG00000010875,ENSOARG00000009966,ENSOARG00000017129,ENSOARG00000001754,ENSOARG00000020695,ENSOARG00000018474,ENSOARG00000016662,ENSOARG00000011800,ENSOARG00000012743,ENSOARG00000016520	20	4	MUDENG,BMI1,PCNP,SEP15	24
**M175**	cell development	EPHB2,SFRP2,GREM1,AXIN2,ASCL2,CDX2,IHH,COL5A1,SATB2,GAS1,CTHRC1,IRX3,COL11A1	others	ENSOARG00000007903,ENSOARG00000001680,ENSOARG00000015486,ENSOARG00000012435,ENSOARG00000019793,ENSOARG00000002129,ENSOARG00000015783,ENSOARG00000016509,ENSOARG00000017964,ENSOARG00000018591	10	3	GREM1,ASCL2,GAS1	13
**M176**	TBA	GJA1,IGF1,AQP1,FRZB,DNASE1L3,IGFBP3,SNAI2,CXCL12,C7,ALDH1A3	others	ENSOARG00000019963,ENSOARG00000015856,ENSOARG00000007775,ENSOARG00000016764,ENSOARG00000012532,ENSOARG00000012862,ENSOARG00000013030,ENSOARG00000002991,ENSOARG00000009128,ENSOARG00000009599,ENSOARG00000019963,ENSOARG00000015856,ENSOARG00000007775,ENSOARG00000016764,ENSOARG00000012532,ENSOARG00000012862,ENSOARG00000013030,ENSOARG00000002991,ENSOARG00000009128,ENSOARG00000009599	10	0		10
**M177.0**	TBA	STX1A,ZNF234,CCR2,ZNF780A,ZFP3,TNFRSF12A,C20orf94,RNF170,HIC1,ZNF167,PPARGC1B,VKORC1L1,ZNF573,DUSP4,PPP1R16B,LOC100131067,ZNF559,FAM135A,ZMYM1,ATG4C,CTR9,NR4A3,ATP10D,GIMAP8,IL1RAP,GIMAP4,GLCE,GIMAP6,GIMAP1,ZNF623,CX3CR1,NRIP3,BBS10,C15orf48,CSRP2BP	others	ENSOARG00000012120,ENSOARG00000008999,ENSOARG00000014236,ENSOARG00000004280,ENSOARG00000000747,ENSOARG00000003520,ENSOARG00000014839,ENSOARG00000005658,ENSOARG00000010272,ENSOARG00000000405,ENSOARG00000011336,ENSOARG00000019437,ENSOARG00000009546,ENSOARG00000010482,ENSOARG00000010939,ENSOARG00000015932,ENSOARG00000001131,ENSOARG00000020462,ENSOARG00000018604,ENSOARG00000001356,ENSOARG00000014010,ENSOARG00000013951,ENSOARG00000014852,ENSOARG00000021041,ENSOARG00000018339	25	10	ZNF780A,C20orf94,ZNF167,VKORC1L1,ZNF573,LOC100131067,ZNF559,GIMAP4,GIMAP1,ZNF623	35
**M177.1**	TBA	ZNF623,NCRNA00183,C20orf94,CX3CR1,TMOD2,NR4A3,ZNF485,VKORC1L1,CCR2,ZNF573,FOSL1,KIAA1919,GIMAP6,ZFP3,LOC100131067	others	ENSOARG00000014010,ENSOARG00000020962,ENSOARG00000010939,ENSOARG00000014236,ENSOARG00000000072,ENSOARG00000009835,ENSOARG00000001356,ENSOARG00000004280	8	7	ZNF623,NCRNA00183,C20orf94,ZNF485,VKORC1L1,ZNF573,LOC100131067	15
**M178**	enriched for promoter motif NATCACGTGAY (putative SREBF1 targets)	ARHGEF12,NGFRAP1,SOCS2,CAMK2D,PRKCE,KIT,AKAP12,PNOC,GEM,BLNK	others	ENSOARG00000017361,ENSOARG00000009921,ENSOARG00000015864,ENSOARG00000018416,ENSOARG00000005847,ENSOARG00000000078,ENSOARG00000003112,ENSOARG00000014922,ENSOARG00000007839,ENSOARG00000007320	10	0		10

**M179**	enriched for TF motif PAX3	TRMT11,TNFAIP8,NUP54,USP1,CASP8AP2,BMI1,FAM35A,PURA,PMS1,RANBP6	others	ENSOARG00000007813,ENSOARG00000016662,ENSOARG00000016734,ENSOARG00000009203,ENSOARG00000012409,ENSOARG00000000827,ENSOARG00000013578,ENSOARG00000016301	8	2	BMI1,RANBP6	10
**M180**	TBA	OSTM1,SIRT1,NPTN,CHUK,HIF1A,ROCK1,SYNJ1,ACSL4,TCF12,ATF2,IREB2	others	ENSOARG00000010883,ENSOARG00000004826,ENSOARG00000019020,ENSOARG00000013829,ENSOARG00000021136,ENSOARG00000008819,ENSOARG00000013414,ENSOARG00000017954,ENSOARG00000020872,ENSOARG00000000194,ENSOARG00000000881	11	0		11
**M181**	nucleotide metabolism	CTPS,ATIC,PAICS,RRM2,RRM1,GART,CAD,ADSL,PFAS,GMPS	cell cycle	ENSOARG00000019309,ENSOARG00000015333,ENSOARG00000001301,ENSOARG00000013139,ENSOARG00000019222,ENSOARG00000017303,ENSOARG00000003146	7	3	CTPS,PAICS,PFAS	10
**M182**	enriched in DNA interacting proteins	STIL,DNAJC9,STMN1,CCDC99,SUV39H1,TAF5,BLM,ACAT1,YWHAH,POLD3,KIAA0494,RRM1,ALS2CR4,PPIL5,TFDP1,DONSON	others	ENSOARG00000003564,ENSOARG00000007522,ENSOARG00000012293,ENSOARG00000012096,ENSOARG00000003445,ENSOARG00000012875,ENSOARG00000011112,ENSOARG00000004824,ENSOARG00000010224,ENSOARG00000001301,ENSOARG00000013016	11	5	CCDC99,KIAA0494,ALS2CR4,PPIL5,TFDP1	16
**M183**	TBA	FXYD3,CDX1,EHF,KLF5,GMDS,LAD1,PKP3,TMEM45B,TRIM29,GALNT3	others	ENSOARG00000004696,ENSOARG00000006690,ENSOARG00000018677,ENSOARG00000015505,ENSOARG00000002773,ENSOARG00000007888,ENSOARG00000014210,ENSOARG00000016590,ENSOARG00000005138	9	1	LAD1	10
**M184.0**	TBA	CDKN2B,GPR144,BMP4,DLG4,ACVR1C,ARHGEF4,FOXO4,CSF3,MAP3K10,MAP2K7,MYOCD,FOXL1,TRPM4,APLP1,COL16A1,GP1BB,WNT11,RGMA	others	ENSOARG00000021069,ENSOARG00000010397,ENSOARG00000007755,ENSOARG00000015878,ENSOARG00000016065,ENSOARG00000012606,ENSOARG00000006462,ENSOARG00000002988,ENSOARG00000015496,ENSOARG00000011648,ENSOARG00000012721,ENSOARG00000005139,ENSOARG00000011113,ENSOARG00000012643,ENSOARG00000010215	15	3	CDKN2B,GPR144,GP1BB	18
**M184.1**	TBA	ADAMTSL2,CDKN2B,DLG4,CARD10,PSD,INS,MAP3K10,WNT11,FOXL1,TRPM4,MAPK8IP2	others	ENSOARG00000003129,ENSOARG00000010397,ENSOARG00000019253,ENSOARG00000000653,ENSOARG00000006462,ENSOARG00000012643,ENSOARG00000011648,ENSOARG00000012721,ENSOARG00000019988	9	2	CDKN2B,INS	11
**M185**	TBA	HDAC2,NPTN,DLD,HPRT1,TCF12,PRNP,RB1,CTR9,SYNJ1,ATG5,RNF6,FNDC3A,PTPN11,BNIP2	others	ENSOARG00000009493,ENSOARG00000019020,ENSOARG00000007136,ENSOARG00000020872,ENSOARG00000008246,ENSOARG00000010482,ENSOARG00000013414,ENSOARG00000011199,ENSOARG00000012743,ENSOARG00000008377,ENSOARG00000009565,ENSOARG00000020826	12	2	HPRT1,PRNP	14
**M186**	TBA	PDLIM7,ELAVL3,EVX1,SOX15,CPEB3,GP1BB,TCF15,YBX1,YBX1P2,PSD,ARMC9,GRIN1	others	ENSOARG00000005380,ENSOARG00000018464,ENSOARG00000009439,ENSOARG00000014928,ENSOARG00000016671,ENSOARG00000019015,ENSOARG00000020447,ENSOARG00000000653,ENSOARG00000020693,ENSOARG00000001953	10	2	GP1BB,YBX1P2	12
**M187**	metabolism in mitochondria, peroxisome	SLC10A2,SLC28A1,CYP4A11,PRODH2,SLC2A2,LOC100422737,AGXT2,LOC100130232,GLYAT,CYP4A22,EHHADH,ACMSD	others	ENSOARG00000005368,ENSOARG00000013352,ENSOARG00000005080,ENSOARG00000020765,ENSOARG00000011462,ENSOARG00000020569,ENSOARG00000011350	7	5	CYP4A11,LOC100422737,LOC100130232,GLYAT,CYP4A22	12
**M188**	TBA	LCN2,BPI,CARD11,OLFM4,SYTL2,PLCXD2,RNF144A,ATP8B4,CEACAM8,KIF21A	others	ENSOARG00000018736,ENSOARG00000004356,ENSOARG00000006710,ENSOARG00000004896,ENSOARG00000019192,ENSOARG00000014661,ENSOARG00000020996,ENSOARG00000020036	8	2	LCN2,CEACAM8	10
**M189**	extracellular region cluster (GO)	CCL2,SEPP1,BMP2,ENG,CCL18,MMP28,HSPG2,CD34,MMP19,ECM1,CMTM8,NPY,CHI3L2,CCL23,CD5L,GH1	others	ENSOARG00000009627,ENSOARG00000008775,ENSOARG00000018738,ENSOARG00000011387,ENSOARG00000005655,ENSOARG00000008248,ENSOARG00000013714,ENSOARG00000010943,ENSOARG00000020820,ENSOARG00000015016,ENSOARG00000010915,ENSOARG00000019517,ENSOARG00000013932	13	3	CCL18,CCL23,CD5L	16
**M190**	TBA	PPP1CC,BUB3,HAUS3,BMI1,CCNG1,OSM,EREG,IL1A,IL1B,CD2AP,USP16	others	ENSOARG00000018756,ENSOARG00000015302,ENSOARG00000014048,ENSOARG00000006353,ENSOARG00000014953,ENSOARG00000020877,ENSOARG00000020866,ENSOARG00000011675	8	3	BUB3,BMI1,USP16	11
**M191**	transmembrane transport (II)	SLC19A2,SIRT1,AXIN2,NUP54,KCNK5,MDFIC,ATP8A1,SLC35A3,ACSL5,SPNS3,SLC26A3,ATP11C,HEPH,ATP2C2,CUL5,KCNQ1,SLC12A2,CFTR	others	ENSOARG00000004826,ENSOARG00000015486,ENSOARG00000016734,ENSOARG00000016548,ENSOARG00000001754,ENSOARG00000014541,ENSOARG00000017849,ENSOARG00000011792,ENSOARG00000019062,ENSOARG00000006903,ENSOARG00000010246,ENSOARG00000005331,ENSOARG00000010703,ENSOARG00000010875,ENSOARG00000002794,ENSOARG00000017613,ENSOARG00000018133	17	1	SLC19A2	18

**M192**	TBA	TGFB1,OGDH,PRMT1,G6PD,DEAF1,ILK,TLN1,MAP1S,NR1H2,NAPA,ARHGDIA,NBEAL2	others	ENSOARG00000007468,ENSOARG00000014862,ENSOARG00000004237,ENSOARG00000006285,ENSOARG00000017258,ENSOARG00000011863,ENSOARG00000015551,ENSOARG00000013898,ENSOARG00000011245,ENSOARG00000017985,ENSOARG00000005857	11	1	PRMT1	12
**M193**	TBA	XK,NFIA,CTTN,C6orf192,ANKH,ATP1B2,SPTB,MGLL,PLEK2,GMPR,PBX1	others	ENSOARG00000018927,ENSOARG00000008860,ENSOARG00000000456,ENSOARG00000013109,ENSOARG00000015381,ENSOARG00000021154,ENSOARG00000005578,ENSOARG00000021167,ENSOARG00000012220,ENSOARG00000011306	10	1	C6orf192	11
**M194**	TBA	SMC1A,XPO1,ADNP,NOC3L,CETN3,NUP37,TXNDC9,TTC35,MTO1,PPP2R5C,ATF1,CRNKL1,CTCF,ARMC1	others	ENSOARG00000008581,ENSOARG00000020174,ENSOARG00000013868,ENSOARG00000003978,ENSOARG00000016247,ENSOARG00000015613,ENSOARG00000013417,ENSOARG00000006136,ENSOARG00000002427,ENSOARG00000017519,ENSOARG00000002150,ENSOARG00000003053,ENSOARG00000017043	13	1	TTC35	14
**M195**	muscle contraction, SRF targets	LOC728264,MYH11,CNN1,ACTG2,PGM5,DES,MRGPRF,DES,FAM48A,MYOCD,COL1A1,PLN,LMOD1	others	ENSOARG00000008975,ENSOARG00000018621,ENSOARG00000012108,ENSOARG00000013106,ENSOARG00000020185,ENSOARG00000017116,ENSOARG00000020185,ENSOARG00000015496,ENSOARG00000004871,ENSOARG00000019971,ENSOARG00000000119	11	2	LOC728264,FAM48A	13
**M196**	platelet activation - actin binding	ITGB3,ITGB5,WASF3,ITGA2B,MPL,TPM4,PROS1,TGFB2,TPM1,THBS1,ACSBG1,ABLIM3,MFAP3L,ARHGAP6,HSPC159,ASAP2,PDE5A	myeloid cells/inflammation	ENSOARG00000020222,ENSOARG00000012699,ENSOARG00000020527,ENSOARG00000000875,ENSOARG00000017273,ENSOARG00000011583,ENSOARG00000020797,ENSOARG00000020058,ENSOARG00000000664,ENSOARG00000005014,ENSOARG00000015459,ENSOARG00000010821,ENSOARG00000014894,ENSOARG00000016017	14	3	ITGB3,ITGA2B,HSPC159	17
**M197**	TBA	LPHN2,PLSCR4,WWTR1,NOVA1,TANC1,ELTD1,NR2F2,LDB2,GUCY1A3,FLRT2,ARHGAP29,NRP1,MPDZ,YAP1	others	ENSOARG00000005630,ENSOARG00000004797,ENSOARG00000004870,ENSOARG00000007585,ENSOARG00000010167,ENSOARG00000010267,ENSOARG00000008232,ENSOARG00000017560,ENSOARG00000017193,ENSOARG00000017485,ENSOARG00000013861,ENSOARG00000006660	12	2	LPHN2,ELTD1	14
**M198**	TBA	CEBPB,MRVI1,PDLIM7,LST1,CCNJL,PISD,TECPR2,XPO6,TSEN34,PHC2,LYN,CPPED1	others	ENSOARG00000013395,ENSOARG00000010763,ENSOARG00000005380,ENSOARG00000008167,ENSOARG00000013371,ENSOARG00000011283,ENSOARG00000004698,ENSOARG00000000960,ENSOARG00000002763,ENSOARG00000020832,ENSOARG00000014778	11	1	CPPED1	12
**M199**	platelet activation & blood coagulation	PROS1,LY6G6D,LY6G6F,GP1BB,SEPT5,SH3BGRL2,PCSK6,VWF,GP1BA,PRKAR2B,GP9,GP5,GP6,EGF,TTC7B	myeloid cells/inflammation	ENSOARG00000017273,ENSOARG00000005880,ENSOARG00000007077,ENSOARG00000016657,ENSOARG00000008752,ENSOARG00000004957,ENSOARG00000020421,ENSOARG00000002001,ENSOARG00000003519	9	6	LY6G6F,GP1BB,SEPT5,GP1BA,GP9,EGF	15
**M200**	antigen processing and presentation	CD1D,WDFY4,METTL7A,OVAR-DQA1,OVAR-DQA2,OVAR-DQB1,OVAR-DQB2,HLA-DMA,OVAR-DM, DQA	DC/antigen presentation	ENSOARG00000007252,ENSOARG00000001659,ENSOARG00000007972, ENSOARG00000007252,ENSOARG00000001659,ENSOARG00000007972	3	7	METTL7A,OVAR-DQA1,OVAR-DQA2,OVAR-DQB1,OVAR-DQB2, OVAR-DM, DQA	10
**M201**	TBA	MYH10,NFIA,ABCG2,C17orf99,SLC14A1,PDGFA,ANKH,TSPAN5,PAQR9,ARHGEF12,TMOD1,HEPACAM2,TGM2,TNXA,TNXB,CDH1,C2orf88,ABCC13,PBX1	others	ENSOARG00000001617,ENSOARG00000008860,ENSOARG00000001914,ENSOARG00000002395,ENSOARG00000006153,ENSOARG00000013109,ENSOARG00000016064,ENSOARG00000017361,ENSOARG00000011271,ENSOARG00000018713,ENSOARG00000018528,ENSOARG00000001254,ENSOARG00000003455,ENSOARG00000019590,ENSOARG00000011306	15	4	C17orf99,PAQR9,TNXA,ABCC13	19
**M202**	enriched in extracellular matrix & associated proteins	PDGFRA,SFRP4,TWIST1,CCDC80,PCDH7,PCOLCE,GLT8D2,GAS1,PRRX1,FBLN1,CALD1,LUM	others	ENSOARG00000019070,ENSOARG00000008899,ENSOARG00000009754,ENSOARG00000019332,ENSOARG00000009059,ENSOARG00000016574,ENSOARG00000016640,ENSOARG00000012029,ENSOARG00000019404,ENSOARG00000007819,ENSOARG00000015727	11	1	GAS1	12
**M203**	TBA	PHTF2,AGL,RCN2,EEA1,ATF1,MAP3K7,ZBTB41,ATP11C,C12orf29,IKZF5,MBNL2,C12orf23,ATF2	others	ENSOARG00000017915,ENSOARG00000017762,ENSOARG00000001570,ENSOARG00000015811,ENSOARG00000017519,ENSOARG00000012321,ENSOARG00000015070,ENSOARG00000010246,ENSOARG00000015472,ENSOARG00000009064,ENSOARG00000002685,ENSOARG00000000194	12	1	C12orf23	13
**M204.0**	chaperonin mediated protein folding (I)	CCT8,ATIC,CCT2,CCT3,NOP56,CCT7,CCT4,CCT5,HSPD1,SUCLG1,HSPE1,SSBP1,FBXW2,CCT6A	others	ENSOARG00000014500,ENSOARG00000019309,ENSOARG00000020314,ENSOARG00000005845,ENSOARG00000006470,ENSOARG00000011826,ENSOARG00000014259,ENSOARG00000015476,ENSOARG00000020397,ENSOARG00000014046,ENSOARG00000005690,ENSOARG00000010011	12	2	CCT4,HSPE1	14

**M204.1**	chaperonin mediated protein folding (II)	CCT8,PFDN5,PFDN4,CCT2,CCT3,CCT7,CCT4,CCT5,CCT6A,VBP1	others	ENSOARG00000014500,ENSOARG00000020314,ENSOARG00000005845,ENSOARG00000011826,ENSOARG00000014259,ENSOARG00000010011,ENSOARG00000003674	7	3	PFDN5,PFDN4,CCT4	10
**M205**	TBA	SIRT1,BAG4,RNF111,RPS6KB1,CHUK,RB1,ZFAND6,RAPGEF6,BMPR2,ARFGEF2,AKAP11	others	ENSOARG00000004826,ENSOARG00000001325,ENSOARG00000020839,ENSOARG00000014401,ENSOARG00000013829,ENSOARG00000008246,ENSOARG00000016418,ENSOARG00000017641,ENSOARG00000011349,ENSOARG00000007363	10	1	ZFAND6	11
**M206**	Wnt signaling pathway	LOXL2,LAMC1,ANTXR1,SFRP4,FRZB,MAP1B,YAP1,CHL1,PMP22,KITLG,CXCL12,NRP1,SOX4,PDZRN3	others	ENSOARG00000010126,ENSOARG00000019180,ENSOARG00000019828,ENSOARG00000008899,ENSOARG00000016764,ENSOARG00000005188,ENSOARG00000006660,ENSOARG00000009067,ENSOARG00000016485,ENSOARG00000015592,ENSOARG00000002991,ENSOARG00000017485,ENSOARG00000009518,ENSOARG00000009378	14	0		14
**M207**	TBA	LRP2,SMG6,RAB26,NPY5R,SLC2A2,SLC22A2,SERPINF2,SLC6A13,ATP7B,RIMS3	others	ENSOARG00000003592,ENSOARG00000015076,ENSOARG00000017671,ENSOARG00000000234,ENSOARG00000020765,ENSOARG00000004692,ENSOARG00000014081,ENSOARG00000011827,ENSOARG00000009056,ENSOARG00000020276	10	0		10
**M208**	TBA	SCAI,HACE1,APP,HOXA7,HOXB9,JMY,ARHGAP5,CCL23,NKX2-1,TUBE1	others	ENSOARG00000013085,ENSOARG00000011435,ENSOARG00000015056,ENSOARG00000006872,ENSOARG00000017278,ENSOARG00000006439,ENSOARG00000008021,ENSOARG00000009694	8	2	HOXA7,CCL23	10
**M209**	lysosome	GNS,CTSB,GM2A,PSAP,CD68,CTSZ,CTSL1,CTSL2,SORT1,FUCA1	DC/antigen presentation	ENSOARG00000003247,ENSOARG00000015263,ENSOARG00000008438,ENSOARG00000006957,ENSOARG00000014729,ENSOARG00000016127,ENSOARG00000019184,ENSOARG00000007012	8	2	CTSL1,CTSL2	10
**M210**	extracellular matrix, collagen	COL10A1,TIMP3,COL11A1,TIMP1,SPARCL1,MFAP5,MFAP4,MFAP2,LAMB1,DCN,PODN,ADAMTS1,MGP,TGFB1I1,COL8A2,SERPINA1,EFEMP1,COMP,EFEMP2,COL4A1,TNC,SFRP2,EMILIN1,OMD,COL14A1,C1QA,CTGF,ADAMTS12,PXDN,COL6A3,CTHRC1,COL12A1	others	ENSOARG00000009417,ENSOARG00000018210,ENSOARG00000018591,ENSOARG00000013157,ENSOARG00000005006,ENSOARG00000001968,ENSOARG00000018248,ENSOARG00000011553,ENSOARG00000007228,ENSOARG00000015737,ENSOARG00000006083,ENSOARG00000014898,ENSOARG00000020724,ENSOARG00000009529,ENSOARG00000019635,ENSOARG00000014882,ENSOARG00000002053,ENSOARG00000010946,ENSOARG00000018379,ENSOARG00000006115,ENSOARG00000005941,ENSOARG00000001680,ENSOARG00000019087,ENSOARG00000008263,ENSOARG00000016790,ENSOARG00000008001,ENSOARG00000014240,ENSOARG00000011993,ENSOARG00000005275,ENSOARG00000019080,ENSOARG00000016509,ENSOARG00000006410	32	0		32
**M211**	TBA	NEK7,ADD3,RNF11,SLC25A46,PLEKHF2,CMPK1,MDFIC,BMI1,RAP1B,CCNG1,PRKRIR	others	ENSOARG00000015482,ENSOARG00000009304,ENSOARG00000004451,ENSOARG00000018956,ENSOARG00000014688,ENSOARG00000003581,ENSOARG00000001754,ENSOARG00000001039,ENSOARG00000014048,ENSOARG00000012702	10	1	BMI1	11
**M212**	purine nucleotide biosynthesis	CHCHD3,ATIC,MTHFD1,RUVBL2,CCT3,CCT7,IMPDH2,PAICS,FKBP3,ADSL,PFAS,GMPS	others	ENSOARG00000006757,ENSOARG00000019309,ENSOARG00000021150,ENSOARG00000012568,ENSOARG00000005845,ENSOARG00000011826,ENSOARG00000015447,ENSOARG00000009326,ENSOARG00000017303,ENSOARG00000003146	10	2	PAICS,PFAS	12
**M213**	regulation of transcription, transcription factors	CREBZF,KIAA1109,C14orf138,PTBP2,KIAA1468,STAG2,MED23,ZNF700,ZDHHC17,DUSP10,SDCCAG1,NCRNA00201,PPP1R12A,BDP1,FMR1,CLK1,ARID4B,CCNT2,CLK4,BTAF1,SFRS12	others	ENSOARG00000005058,ENSOARG00000000316,ENSOARG00000017531,ENSOARG00000006048,ENSOARG00000013895,ENSOARG00000013991,ENSOARG00000014899,ENSOARG00000013213,ENSOARG00000015054,ENSOARG00000005347,ENSOARG00000009673,ENSOARG00000016541,ENSOARG00000003481,ENSOARG00000011259,ENSOARG00000006692,ENSOARG00000016528	16	5	C14orf138,ZNF700,SDCCAG1,NCRNA00201,SFRS12	21
**M214**	TBA	TGFBR1,RP2,SOS2,C6orf211,CHUK,PIK3CG,ZFAND6,STXBP3,ATP11B,TMEM30A,SPAST,LPGAT1	others	ENSOARG00000010971,ENSOARG00000013968,ENSOARG00000020627,ENSOARG00000013829,ENSOARG00000004785,ENSOARG00000018946,ENSOARG00000020665,ENSOARG00000006481,ENSOARG00000010758,ENSOARG00000011368	10	2	C6orf211,ZFAND6	12
**M215**	small GTPase mediated signal transduction	TAGAP,RAC2,FGD3,ARAP1,RHOH,GMIP,VAV1,ARHGAP30,HMHA1,ARHGAP15,ARHGDIB,ARHGAP9,ARHGAP25,ARHGDIA,ARHGAP4,RHOG	others	ENSOARG00000004469,ENSOARG00000008161,ENSOARG00000005950,ENSOARG00000000571,ENSOARG00000007313,ENSOARG00000004983,ENSOARG00000009399,ENSOARG00000010834,ENSOARG00000009910,ENSOARG00000020698,ENSOARG00000006591,ENSOARG00000019876,ENSOARG00000017985,ENSOARG00000001509	14	2	RAC2,RHOG	16
**M216**	respiratory electron transport chain (mitochondrion)	NDUFA7,NDUFB7,COX6A1,UQCRB,NDUFB10,ATP5J,UQCR10,ATP5H,NDUFS6,COX7B,COX7C,COX6C	others	ENSOARG00000003683,ENSOARG00000005411,ENSOARG00000012290,ENSOARG00000005495,ENSOARG00000016876,ENSOARG00000015187,ENSOARG00000005560,ENSOARG00000011397,ENSOARG00000011680	9	3	COX7B,COX7C,COX6C	12

**M217**	TBA (source: B cells)	FMO3,PLSCR4,PROS1,FLRT2,MXRA5,ABI3BP,TMEM47,CDH5,LUM,MEOX2	B cells	ENSOARG00000012259,ENSOARG00000005630,ENSOARG00000017273,ENSOARG00000017560,ENSOARG00000008760,ENSOARG00000018194,ENSOARG00000018704,ENSOARG00000001849,ENSOARG00000015727,ENSOARG00000008280	10	0		10
**M218**	TBA	PREPL,ATG4C,CTR9,C3orf1,GTF2H1,ALG6,SLC35B3,POT1,HPS5,SLC35A5,C11orf73,RPE,PMS1,ELP3,ACADM,CSRP2BP	others	ENSOARG00000006193,ENSOARG00000009546,ENSOARG00000010482,ENSOARG00000009671,ENSOARG00000009650,ENSOARG00000016835,ENSOARG00000001527,ENSOARG00000009767,ENSOARG00000019330,ENSOARG00000004398,ENSOARG00000016301,ENSOARG00000014910,ENSOARG00000018339	13	3	C3orf1,RPE,ACADM	16
**M219**	respiratory electron transport chain (mitochondrion)	UQCRC2,COX5A,COX5B,UQCR10,COX7B,CYC1,UQCRB,UQCRQ,UQCRH,COX8A,ATP5L,COX6B1,UQCRFS1,UQCRC1,COX6A1,COX7C,COX6C,COX7A2L	others	ENSOARG00000015094,ENSOARG00000002812,ENSOARG00000013670,ENSOARG00000005560,ENSOARG00000015048,ENSOARG00000005495,ENSOARG00000013236,ENSOARG00000016841,ENSOARG00000004946,ENSOARG00000016718,ENSOARG00000012290	11	7	COX7B,UQCRH,COX8A,UQCRFS1,COX7C,COX6C,COX7A2L	18
**M220**	TBA	SFN,SPDEF,KIAA1324,KCNC3,TFAP2A,SOX9,GRIN2B,AR,REEP1,NOS1AP	others	ENSOARG00000018302,ENSOARG00000010751,ENSOARG00000019083,ENSOARG00000013837,ENSOARG00000016395,ENSOARG00000013779,ENSOARG00000020802,ENSOARG00000014525,ENSOARG00000020650,ENSOARG00000010597	10	0		10
**M221**	TBA	RNF17,ACCN5,ESCO2,LBX1,IFNA7,POU4F2,IFNA4,FLJ37543,CCDC144C,NCRNA00119,TRIM49,CCDC26,MST1,MST1P2,MST1P9	others	ENSOARG00000014641,ENSOARG00000014845,ENSOARG00000016434,ENSOARG00000013151	4	11	ACCN5,IFNA7,POU4F2,IFNA4,FLJ37543,CCDC144C,NCRNA00119,TRIM49,CCDC26,MST1P2,MST1P9	15
**M222**	heme biosynthesis (II)	HMBS,SPTA1,ALAD,RFESD,TRIM10,RHCE,RHD,RHCE,CPOX,PPOX,KLF1,UROD,BLVRB,YPEL4	others	ENSOARG00000012985,ENSOARG00000007488,ENSOARG00000006271,ENSOARG00000017275,ENSOARG00000016038,ENSOARG00000017706,ENSOARG00000009648,ENSOARG00000010245,ENSOARG00000001472,ENSOARG00000006640,ENSOARG00000010100	11	3	RHCE,RHD,RHCE	14
**M223**	enriched in T cells (II)	SLC38A1,HOPX,CD40LG,DENND2D,TC2N,PLEKHF1,LRRN3,CLEC2D,FGFBP2,LBH,TRGC2,DPP4	T/NK cells	ENSOARG00000019508,ENSOARG00000004859,ENSOARG00000011076,ENSOARG00000019512,ENSOARG00000012362,ENSOARG00000004052,ENSOARG00000003374,ENSOARG00000021075,ENSOARG00000019636,ENSOARG00000001797,ENSOARG00000006454, ENSOARG00000019508,ENSOARG00000004859,ENSOARG00000011076,ENSOARG00000019512,ENSOARG00000012362,ENSOARG00000004052,ENSOARG00000003374,ENSOARG00000021075,ENSOARG00000019636,ENSOARG00000017970,ENSOARG00000006454	11	1	FGFBP2	12
**M224**	transmembrane and ion transporters (II)	NTF3,DMD,GRIK3,SLC24A2,CACNA1F,JPH2,SLC4A3,CALCA,SLC1A6,KCNA5,ATP7B	others	ENSOARG00000009046,ENSOARG00000018256,ENSOARG00000019747,ENSOARG00000014276,ENSOARG00000010594,ENSOARG00000003672,ENSOARG00000020255,ENSOARG00000006109,ENSOARG00000004610,ENSOARG00000009056	10	1	KCNA5	11
**M225**	metabolism of steroids	GALNT14,ALPK2,UGT2A3,LOC100422737,UGT2B15,MIOX,UGT2B17,PKHD1,HAVCR1,UGT2B28	others	ENSOARG00000019676,ENSOARG00000005476,ENSOARG00000009742,ENSOARG00000019864,ENSOARG00000013288	5	5	LOC100422737,UGT2B15,UGT2B17,HAVCR1,UGT2B28	10
**M226**	proteasome	TAF9,RAN,PSMD14,NUP37,PSMA2,PSMA3,PSMA6,PSMC6,PSMA4,PSMA5,PSMC2,POLR2K	DC/antigen presentation	ENSOARG00000010707,ENSOARG00000006699,ENSOARG00000015613,ENSOARG00000017342,ENSOARG00000021109,ENSOARG00000007429,ENSOARG00000017558,ENSOARG00000000996,ENSOARG00000019203,ENSOARG00000019082,ENSOARG00000001480	11	1	TAF9	12
**M227**	translation initiation	ETFA,EIF3J,PSMD14,METTL5,NDUFA8,COPS5,EIF4H,NUP37,EIF2S1,EIF2S2	others	ENSOARG00000003638,ENSOARG00000006699,ENSOARG00000002884,ENSOARG00000014386,ENSOARG00000000428,ENSOARG00000011868,ENSOARG00000015613,ENSOARG00000021166,ENSOARG00000009171	9	1	ETFA	10

**M228**	olfactory receptors	OR7C2,OR7E24,NPIPL2,NPIPL3,OR2C3,IFN-ALPHA,CCDC144C,,OR7D2,OR2H1,MST1,MST1P2,MST1P9,GUCA1C	others	ENSOARG00000016173,ENSOARG00000016408,ENSOARG00000016795,ENSOARG00000011001,ENSOARG00000013151, ENSOARG00000016173,ENSOARG00000016408,ENSOARG00000016795,ENSOARG00000017439,ENSOARG00000013151, ENSOARG00000016173,ENSOARG00000016408,ENSOARG00000016795,ENSOARG00000004662,ENSOARG00000013151, ENSOARG00000016173,ENSOARG00000016408,ENSOARG00000016795,ENSOARG00000013618,ENSOARG00000013151, ENSOARG00000016173,ENSOARG00000016408,ENSOARG00000016795,ENSOARG00000000558,ENSOARG00000013151, ENSOARG00000016173,ENSOARG00000016408,ENSOARG00000016795,ENSOARG00000005067,ENSOARG00000013151, ENSOARG00000016173,ENSOARG00000016408,ENSOARG00000016795,ENSOARG00000013309,ENSOARG00000013151, ENSOARG00000016173,ENSOARG00000016408,ENSOARG00000016795,ENSOARG00000016418,ENSOARG00000013151, ENSOARG00000016173,ENSOARG00000016408,ENSOARG00000016795,ENSOARG00000006617,ENSOARG00000013151, ENSOARG00000016173,ENSOARG00000016408,ENSOARG00000016795,ENSOARG00000004535,ENSOARG00000013151, ENSOARG00000016173,ENSOARG00000016408,ENS[illegible]	5	9	NPIPL2,NPIPL3,IFN-ALPHA,CCDC144C,OR7D2,OR2H1,MST1P2,MST1P9,GUCA1C	14
**M229**	TBA	MIER3,CAND1,CDKN2AIP,AEBP2,CRY1,DYNLT3,BMI1,RAB18,MAPK6,CCNC,PTBP2	others	ENSOARG00000007314,ENSOARG00000002131,ENSOARG00000007148,ENSOARG00000020470,ENSOARG00000017816,ENSOARG00000000125,ENSOARG00000016208,ENSOARG00000020954,ENSOARG00000011800,ENSOARG00000017531	10	1	BMI1	11
**M230**	cell cycle, mitotic phase	HACE1,AHCTF1,SMC3,TP53BP2,CENPC1,CD28,JMY,NAE1,MIS12,TTC19,TUBE1,RANBP2	cell cycle	ENSOARG00000011435,ENSOARG00000003217,ENSOARG00000009745,ENSOARG00000015629,ENSOARG00000018277,ENSOARG00000017278,ENSOARG00000002298,ENSOARG00000017392,ENSOARG00000009694,ENSOARG00000000558	10	2	CENPC1,MIS12	12
**M231**	respiratory electron transport chain (mitochondrion)	ATP5C1,NDUFAB1,COX5A,NDUFB4,NDUFB3,NDUFB1,NDUFS3,NDUFA8,ATP5L,UQCRFS1,NDUFB11	others	ENSOARG00000000521,ENSOARG00000002812,ENSOARG00000019904,ENSOARG00000016831,ENSOARG00000020197,ENSOARG00000007122,ENSOARG00000014386,ENSOARG00000016841	8	3	ATP5C1,UQCRFS1,NDUFB11	11
**M232**	enriched for TF motif TNCATNTCCYR	TBCK,VPS41,RAD51C,GTF2H1,TCHP,MEN1,HDDC3,SMARCAL1,PDE6D,RAD50,TTC1,ELP4,HSD17B4	others	ENSOARG00000009441,ENSOARG00000017742,ENSOARG00000011574,ENSOARG00000009671,ENSOARG00000014399,ENSOARG00000012506,ENSOARG00000019393,ENSOARG00000020725,ENSOARG00000015021,ENSOARG00000013209,ENSOARG00000016057,ENSOARG00000000722	12	1	MEN1	13
**M233**	TBA	MAP3K10,BMP4,ELAVL3,PRDM8,HOXD10,JPH2,CRYBB3,PCDHB11,BOK,PLA2G2D,GFRA4,C8orf39,NCRNA00105,FOXN1,HOXB13	others	ENSOARG00000006462,ENSOARG00000021069,ENSOARG00000018464,ENSOARG00000018972,ENSOARG00000017514,ENSOARG00000003672,ENSOARG00000018985,ENSOARG00000017652,ENSOARG00000009461,ENSOARG00000002457,ENSOARG00000018638,ENSOARG00000006844	12	3	PCDHB11,C8orf39,NCRNA00105	15
**M234**	transcription elongation, RNA polymerase II	MEA1,NDUFA3,AURKAIP1,TCEB2,NDUFB11,NDUFB10,POLR2E,UQCR10,POLR2L,BOLA2,BOLA2B,UQCRQ,MIF,POLR2I,POLR2J	others	ENSOARG00000003076,ENSOARG00000003838,ENSOARG00000000340,ENSOARG00000016876,ENSOARG00000010994,ENSOARG00000005560,ENSOARG00000015719,ENSOARG00000013236,ENSOARG00000005266,ENSOARG00000014204	10	5	MEA1,NDUFB11,BOLA2,BOLA2B,MIF	15
**M235**	mitochondrial cluster	DNAJA3,ALG8,EIF2B3,PUS7,C3orf1,WDR92,TCHP,C11orf73,NDUFA8,C14orf126,SMARCAL1,MCCC2,ALDH6A1,POP5,HIBCH,HIBADH,ALDH18A1,TMEM218,ACADM	others	ENSOARG00000003302,ENSOARG00000006601,ENSOARG00000001396,ENSOARG00000003160,ENSOARG00000019929,ENSOARG00000014399,ENSOARG00000004398,ENSOARG00000014386,ENSOARG00000019393,ENSOARG00000005258,ENSOARG00000000940,ENSOARG00000012764,ENSOARG00000016273,ENSOARG00000006313,ENSOARG00000011312	15	4	C3orf1,C14orf126,HIBADH,ACADM	19
**M236**	TBA	GTF2E1,BET1,PAQR8,FTSJD1,ZNF189,CRNKL1,POLR1B,UTP14C,ZKSCAN4,GIMAP4,EXOSC3,GIMAP6,C17orf80,VPRBP	others	ENSOARG00000019957,ENSOARG00000008750,ENSOARG00000007528,ENSOARG00000002150,ENSOARG00000020904,ENSOARG00000017908,ENSOARG00000011386,ENSOARG00000001356,ENSOARG00000013622,ENSOARG00000007546	10	4	BET1,FTSJD1,UTP14C,GIMAP4	14

**M237**	golgi membrane (II)	ARFIP1,ZDHHC17,GALNT7,C3orf58,GOLPH3L,PAQR3,RAB33B,C1GALT1C1,GOLT1B,SLC35B3,RAB21,PNPLA8,SGMS1,SLC35A3,SLC35A5,COPB2,GOPC	others	ENSOARG00000003643,ENSOARG00000014899,ENSOARG00000015402,ENSOARG00000005732,ENSOARG00000020842,ENSOARG00000018831,ENSOARG00000013460,ENSOARG00000009640,ENSOARG00000020337,ENSOARG00000016835,ENSOARG00000004691,ENSOARG00000008013,ENSOARG00000013961,ENSOARG00000017849,ENSOARG00000019330,ENSOARG00000007072,ENSOARG00000008696	17	0		17
**M238**	respiratory electron transport chain (mitochondrion)	NDUFA6,NDUFA4,NDUFA2,NDUFA3,ATP5J2,NDUFB6,NDUFB2,UQCRQ,NDUFC2,ATP5I,ATP5O,NDUFA11,UQCRH,NDUFS7,NDUFS8,NDUFS4,ATP5D	others	ENSOARG00000018854,ENSOARG00000018374,ENSOARG00000003076,ENSOARG00000014760,ENSOARG00000013450,ENSOARG00000013236,ENSOARG00000006694,ENSOARG00000008425,ENSOARG00000012773,ENSOARG00000007018,ENSOARG00000011589,ENSOARG00000014412,ENSOARG00000008081,ENSOARG00000011366	14	3	NDUFA4,ATP5J2,UQCRH	17
**M239**	enriched in calcium signaling proteins	SLITRK6,FLJ35409,FAM196A,EPHB1,DMD,C1orf114,SPOCK3,GRIA4,TRPC3,LOC441666,CAPS2,LOC642426,MYOZ2,F11,LOC338739	others	ENSOARG00000017171,ENSOARG00000013914,ENSOARG00000008094,ENSOARG00000018256,ENSOARG00000002844,ENSOARG00000001072,ENSOARG00000014754,ENSOARG00000016717,ENSOARG00000008283	9	6	FLJ35409,C1orf114,SPOCK3,LOC441666,LOC642426,LOC338739	15
**M240**	chromosome Y linked	XIST,DPF3,UTY,NCRNA00185,CYorf15B,CYorf15A,MAP7D2,TTTY15,TSIX,KAL1,USP9Y,MYOCD,KDM5D,BCORL2,RGMA,PRKY,DDX3Y	others	ENSOARG00000000185,ENSOARG00000015259,ENSOARG00000015496,ENSOARG00000010215	4	13	XIST,UTY,NCRNA00185,CYorf15B,CYorf15A,TTTY15,TSIX,KAL1,USP9Y,KDM5D,BCORL2,PRKY,DDX3Y	17
**M241**	TBA	PAN2,CCNL2,TBC1D3F,TBC1D3H,LOC440354,AHSA2,LOC641298,TBC1D3C,LOC595101,CALCA	others	ENSOARG00000009379,ENSOARG00000003648,ENSOARG00000020279,ENSOARG00000006109	4	6	TBC1D3F,TBC1D3H,LOC440354,LOC641298,TBC1D3C,LOC595101	10
**M242**	TBA	SNX14,SNX13,STRN3,MAP3K2,ACAP2,MED1,RASA2,REL,DYRK1A,CREBBP,FBXW7	others	ENSOARG00000013234,ENSOARG00000009409,ENSOARG00000005730,ENSOARG00000016128,ENSOARG00000020394,ENSOARG00000011074,ENSOARG00000006643,ENSOARG00000001457,ENSOARG00000014368,ENSOARG00000002484,ENSOARG00000004146	11	0		11
**M243**	TBA	BCAP31,NDUFB9,TMEM160,NDUFB7,TBCB,DDT,SCAND1,APRT,PRELID1,SRM,PSMB3,TUFM	others	ENSOARG00000007184,ENSOARG00000008825,ENSOARG00000010898,ENSOARG00000005411,ENSOARG00000005271,ENSOARG00000005354,ENSOARG00000013556,ENSOARG00000004188,ENSOARG00000004158,ENSOARG00000009831,ENSOARG00000002357	11	1	DDT	12
**M244**	TBA	ADCY1,ITGB1BP3,TTLL9,C21orf89,DKFZp779M0652,ADCY8,TLE6,TRIM17,HTR6,CES4,CAMK2N1,AVPR2,GNG8	others	ENSOARG00000013527,ENSOARG00000002139,ENSOARG00000007455,ENSOARG00000013345,ENSOARG00000009788,ENSOARG00000001441,ENSOARG00000010648	7	6	ITGB1BP3,C21orf89,DKFZp779M0652,TRIM17,CES4,CAMK2N1	13
**M245**	translation initiation factor 3 complex	EIF3H,UXT,EIF3K,PHB2,EEF1G,EIF3D,EIF3E,EIF3F,EEF1G,TUT1,RPL3,EIF3G,EEF1B2,FBL	others	ENSOARG00000010674,ENSOARG00000012787,ENSOARG00000005760,ENSOARG00000005015,ENSOARG00000015142,ENSOARG00000018926,ENSOARG00000014786,ENSOARG00000015265,ENSOARG00000015142,ENSOARG00000016495,ENSOARG00000015095,ENSOARG00000018622,ENSOARG00000006339	13	1	TUT1	14
**M246**	TBA	RUNDC2C,RUNDC2B,C14orf82,SNAPC1,RPA4,C15orf5,LOC100128439,PMEPA1,RASSF6,SLC5A8,LOC145474,LOC644852,POLR2J4,KIAA0754,PLIN5,KIAA1659	others	ENSOARG00000021137,ENSOARG00000017358,ENSOARG00000014363,ENSOARG00000014402,ENSOARG00000009333	5	11	RUNDC2C,RUNDC2B,C14orf82,RPA4,C15orf5,LOC100128439,LOC145474,LOC644852,POLR2J4,KIAA0754,KIAA1659	16
**M247**	enriched in nuclear pore complex interacting proteins	IL17A,LOC613037,FAM153A,ANKRD34C,LIN28A,LOC728888,HRH4,CNTNAP3B,EFCAB5,NPIPL3,NPIPL2	others	ENSOARG00000013930,ENSOARG00000017467,ENSOARG00000004614,ENSOARG00000007300,ENSOARG00000009452	5	6	LOC613037,FAM153A,LOC728888,CNTNAP3B,NPIPL3,NPIPL2	11
**M248**	TBA	C11orf2,C17orf70,SSNA1,C1orf86,RASSF7,E4F1,C12orf10,FBXL15,TBL3,ALDH16A1,CCDC85B	others	ENSOARG00000002485,ENSOARG00000006993,ENSOARG00000017910,ENSOARG00000016542,ENSOARG00000001248,ENSOARG00000017059,ENSOARG00000013087	7	4	C11orf2,C17orf70,C1orf86,CCDC85B	11

**M249**	TBA	ANKRD44,TIMP4,LOC339524,HS2ST1,CIDEC,CD300LG,LY75,LEP,PLIN4,C11orf63	others	ENSOARG00000015246,ENSOARG00000014907,ENSOARG00000005878,ENSOARG00000006241,ENSOARG00000002407,ENSOARG00000009400,ENSOARG00000018791, ENSOARG00000015246,ENSOARG00000014907,ENSOARG00000005878,ENSOARG00000006241,ENSOARG00000002407,ENSOARG00000009400,ENSOARG00000018791	7	3	TIMP4,LOC339524,LY75	10
**M250**	spliceosome	SNRPE,SNRPD3,SNRPD1,LSM3,LSM5,SNRPG,RBMX,SF3B14,SNRPA,SNRPF,MAGOH,SNRPD2	others	ENSOARG00000006169,ENSOARG00000013984,ENSOARG00000003413,ENSOARG00000000600,ENSOARG00000006944,ENSOARG00000019701,ENSOARG00000010884,ENSOARG00000006963,ENSOARG00000011853,ENSOARG00000006235,ENSOARG00000010203	11	1	SF3B14	12
**S0**	T cell surface signature	NPDC1,ANKH,LAG3,CD6,CD2,CD320,SLC37A3,C6orf129,NDFIP2,CXCR6,SIT1,SIRPG,ICOS,CD28,SLC38A1,TMEM106C,PTPRCAP,FLT3LG,C12orf23,GIMAP2,LRRN3,CLEC2D,CD3E,ITM2A,GPR171,MAL,CD3G	T/NK cells	ENSOARG00000013109,ENSOARG00000006528,ENSOARG00000016062,ENSOARG00000020249,ENSOARG00000003676,ENSOARG00000012780,ENSOARG00000000420,ENSOARG00000014277,ENSOARG00000011944,ENSOARG00000018331,ENSOARG00000018277,ENSOARG00000019508,ENSOARG00000019371,ENSOARG00000011983,ENSOARG00000013140, ENSOARG00000001542,ENSOARG00000003374,ENSOARG00000021075,ENSOARG00000008984,ENSOARG00000000398,ENSOARG00000015348,ENSOARG00000009256, ENSOARG00000013109,ENSOARG00000006528,ENSOARG00000016062,ENSOARG00000020249,ENSOARG00000003676,ENSOARG00000012780,ENSOARG00000000420,ENSOARG00000014277,ENSOARG00000011944,ENSOARG00000018331,ENSOARG00000018277,ENSOARG00000019508,ENSOARG00000019371,ENSOARG00000011983,ENSOARG00000013140, ENSOARG00000001542,ENSOARG00000003374,ENSOARG00000021075,ENSOARG00000008984,ENSOARG00000000398,ENSOARG00000015348,ENSOARG00000009256	22	5	NPDC1,C6orf129,SIRPG,C12orf23,MAL	27
**S1**	NK cell surface signature	PDGFRB,TNFSF11,IL12RB2,TNFSF14,SLC7A5,ADRB2,S1PR5,ATP9A,TIE1,PTGDR,NCAM1,KLRF1,ELOVL6,IL18RAP,KIR3DP1,KIR3DX1,PTGER2,SLCO4C1,GPR82,TGFBR3,KLRC3,ENPP5,IL18R1,NCR1,DLL1,FASLG,KIR3DL1,KIR2DL5A,KIR3DL3,IL2RB,TMEM64,CX3CR1,KLRK1,CD97,SYNGR3,RARRES3,KIT,ATP8B4,SLAMF7,PVRIG,GPR114,HAVCR2	Tc/NK	ENSOARG00000006538,ENSOARG00000007376,ENSOARG00000011173,ENSOARG00000002699,ENSOARG00000012129,ENSOARG00000014352,ENSOARG00000014351,ENSOARG00000020522,ENSOARG00000020714,ENSOARG00000016809,ENSOARG00000021058,ENSOARG00000004328,ENSOARG00000013079,ENSOARG00000002371,ENSOARG00000002102, ENSOARG00000017673,ENSOARG00000018280,ENSOARG00000019378,ENSOARG00000016047,ENSOARG00000010545,ENSOARG00000013096,ENSOARG00000002157,ENSOARG00000005108,ENSOARG00000014582,ENSOARG00000019197,ENSOARG00000010236,ENSOARG00000014010,ENSOARG00000021000,ENSOARG00000017169,ENSOARG00000000078, ENSOARG00000020996,ENSOARG00000008967,ENSOARG00000017076,ENSOARG00000011033, ENSOARG00000006538,ENSOARG00000007376,ENSOARG00000011173,ENSOARG00000002699,ENSOARG00000012129,ENSOARG00000014352,ENSOARG00000014351,ENSOARG00000020522,ENSOARG00000020714,ENSOARG00000016809,ENSOARG00000021058,ENSOARG00000004328,ENSOARG00000013079,ENSOARG00000002217,ENSOARG00000002102, ENSOARG00000017673,ENSOARG00000018280,ENSOARG00000019378,ENSOARG00000016047,ENSOARG00000010545,ENSOARG00000013096,ENSOARG00000002157,ENSOARG00000005108,ENSOARG00000014582,ENSOARG00000019197,ENSOARG00000010236,ENSOARG00000014010,ENSOARG00000021000,ENSOARG00000017169,ENSOARG00000000078, ENSOARG00000020996,ENSOARG00000008967,ENS[illegible]	34	8	S1PR5,KLRC3,KIR3DL1,KIR2DL5A,KIR3DL3,CD97,RARRES3,GPR114	42

**S2**	B cell surface signature	SSPN,AVPR1A,KCNE4,TSPAN13,CD37,HEPACAM2,GYPA,SEMA4B,EGF,GPR37,MS4A1,C11orf87,USH2A,PPP1R3A,OR12D3,POPDC3,DCC,SLC12A1,GP5,CDH5,SLITRK6,OVAR-DPB1,PCDHA3,TRHDE,FAIM3,CD22,GHR,LY9,TAS2R13,FLRT2,TPTE,TMEM100,GYPE,PLD4,ADAM7,KCNMB3,FCRL3,GPR6,CSPG4,SYT2,ROBO2,NRXN3,FREM2,TRPC1,GPR116,TMEM47,SCN3A,NPY1R,LPPR4,NOX4,PDZK1IP1,CCR9,CD200,GABBR1,EDNRB,GJC1,LHCGR,PCDHB8,NT5E,PDPN,PCDHB4,CLDN8,LRRTM4,HRH4,NMBR,NCAM2,GRM5,TM4SF20,IL5RA,JAM2,JAM3,IL28RA,MC3R,F2RL3,ASTN1,MOGAT2,SLC26A7,SLC24A1,TACR3,CR2,IL13RA2,IMPG2,LRRC19,TMEFF2,ITM2C,PCDH10,BTC,GPR98,AGTR1,CD19,TMEM133,CD72,UGT2A3,OR11A1,TLR10,CSMD1,TRPC7,CCR6,GABRA5,PLP1,CD79B,CLCA4,CD79A,CNR1,EPHA3,SLC30A10,SGCE,BTLA,CHRNB4,PCDHB10,PCDHB16,PCDHB15,PCDHB14,CADM2,GPR85,SLC6A15,GPR87,NLGN1,SLC13A1,CELSR1,OR1E1,GABRB1,CD180,ADAM28,CXADR,TAAR2,HHLA2,SLC [illegible]	B cells	ENSOARG00000019951,ENSOARG00000004113,ENSOARG00000008934,ENSOARG00000012851,ENSOARG00000018713,ENSOARG00000012066,ENSOARG00000001400,ENSOARG00000013081,ENSOARG00000016968,ENSOARG00000010714,ENSOARG00000001892,ENSOARG00000008901,ENSOARG00000004732,ENSOARG00000021024,ENSOARG00000020421,ENSOARG00000001849,ENSOARG00000017171,ENSOARG00000014716,ENSOARG00000004796,ENSOARG00000008837,ENSOARG00000008981,ENSOARG00000017560,ENSOARG00000013740,ENSOARG00000007988,ENSOARG00000009977,ENSOARG00000020707,ENSOARG00000006866,ENSOARG00000001908,ENSOARG00000016559,ENSOARG00000002748,ENSOARG00000009812,ENSOARG00000005994,ENSOARG00000018704,ENSOARG00000005501,ENSOARG00000013669,ENSOARG00000003844,ENSOARG00000003430,ENSOARG00000009514,ENSOARG00000019298,ENSOARG00000016848,ENSOARG00000000158,ENSOARG00000000133,ENSOARG00000013284,ENSOARG00000010890,ENSOARG00000001028,ENSOARG00000020300,ENSOARG00000007300,ENSOARG00000001289,ENSOARG00000015555,ENSOARG00000020557,ENSOARG00000008664,ENSOARG00000015207,ENSOARG00000013611,ENSOARG00000004827,ENSOARG00000018466,ENSOARG00000015182,ENSOARG00000009573,ENSOARG00000018007,ENSOARG00000010861,ENSOARG00000008208,ENSOARG00000017220,ENSOARG00000018281,ENSOARG00000014554,ENSOARG00000014195,ENSOARG00000020673,ENSOARG00000014523,ENSOARG [illegible]	131	37	KCNE4,GYPA,EGF,POPDC3,OVAR-DPB1,PCDHA3,FAIM3,TAS2R13,TPTE,GYPE,GPR6,SYT2,GPR116,LPPR4,LHCGR,PCDHB8,PCDHB4,GRM5,IL28RA,MOGAT2,GPR98,TMEM133,OR11A1,PCDHB10,PCDHB16,OR1E1,CXADR,CNTNAP3,ABCA8,OR2J2,OR2W1,LPHN3,LPHN2,ABCB4,PCDH20,NLGN4Y,BAI3	168
**S3**	Plasma cell surface signature	PPAP2C,KCNG2,SLC5A4,CAV1,TEK,TMEM37,GP1BB,SLC16A14,ICAM2,SLC44A1,CCR10,CHRM1,CLPTM1L,KCNN3,HM13,GPR25,KCNH1,TXNDC15,SDC1,DRD4,KRTCAP2,TRAM2,SCARB2,AMIGO3	B cells	ENSOARG00000004123,ENSOARG00000012208,ENSOARG00000001337,ENSOARG00000014586,ENSOARG00000019713,ENSOARG00000020626,ENSOARG00000014212,ENSOARG00000007301,ENSOARG00000002956,ENSOARG00000016392,ENSOARG00000002973,ENSOARG00000000910,ENSOARG00000012144,ENSOARG00000014499,ENSOARG00000017166,ENSOARG00000003747,ENSOARG00000014535,ENSOARG00000013040	18	6	PPAP2C,GP1BB,CHRM1,GPR25,DRD4,SCARB2	24
**S4**	Monocyte surface signature	FXYD6,KCNE3,AMICA1,CD33,CD36,C5AR1,FCGR1B,APCDD1,LTBR,PECAM1,CYBRD1,GPR109B,PLXND1,FCAR,DYSF,FPR1,FPR2,HLA-DRB4,CSF3R,CCR1,C19orf59,TLR2,CD163,TLR1,TLR4,TLR5,TREM1,TNFSF12,CLEC12B,CLEC12A,EMR1,EMR2,LPPR2,IL1R2,MARCO,LILRB3,LILRB2,LILRB1,SLC11A1,MGAM,C10orf54,TMEM71,SLC46A2,VNN2,KCNQ1,FCGR2A,TNFRSF1B,MBOAT7,CD93,ITGAM,SIRPB1,GPER,CD4,TNFRSF10B,CCR2,CD302,PTAFR,MCTP1,FCER1A,S1PR3,P2RY13,SLC7A7,AGTRAP,CECR6,IL6R,ASGR1,ASGR2,TMEM154,LRP1,LRRC33,C1orf162,TMTC2,ABCA1,STEAP4,SPNS1,P2RX1,VSTM1,BRI3,NFAM1,TMEM55A,PYCARD,FCGRT,GLIPR1,MFSD1,SLC24A4,PTGIR,MS4A6A,P2RY2,SLC40A1,SLC16A3,ANO10,PTPRE,SIGLEC9,GPR133	myeloid cells/inflammation	ENSOARG00000007061,ENSOARG00000006737,ENSOARG00000017123,ENSOARG00000011005,ENSOARG00000001340,ENSOARG00000008343,ENSOARG00000014779,ENSOARG00000002042,ENSOARG00000013617,ENSOARG00000002196,ENSOARG00000011500,ENSOARG00000019729,ENSOARG00000014260,ENSOARG00000015044,ENSOARG00000002862,ENSOARG00000000538,ENSOARG00000005792,ENSOARG00000005017,ENSOARG00000001869,ENSOARG00000014149,ENSOARG00000021025,ENSOARG00000021026,ENSOARG00000013159,ENSOARG00000012328,ENSOARG00000019535,ENSOARG00000014509,ENSOARG00000006883,ENSOARG00000006277,ENSOARG00000006452,ENSOARG00000014486,ENSOARG00000002794,ENSOARG00000017205,ENSOARG00000002806,ENSOARG00000005169,ENSOARG00000009000,ENSOARG00000006301,ENSOARG00000017294,ENSOARG00000014236,ENSOARG00000007138,ENSOARG00000019840,ENSOARG00000016842,ENSOARG00000007787,ENSOARG00000007690,ENSOARG00000004209,ENSOARG00000019424,ENSOARG00000000056,ENSOARG00000017619,ENSOARG00000002562,ENSOARG00000010137,ENSOARG00000010000,ENSOARG00000003733,ENSOARG00000007332,ENSOARG00000019610,ENSOARG00000015285,ENSOARG00000007344,ENSOARG00000011811,ENSOARG00000001386,ENSOARG00000017892,ENSOARG00000000797,ENSOARG00000018955,ENSOARG00000010089,ENSOARG00000008908,ENSOARG00000013202,ENSOARG00000014795,ENSOARG00000002318,ENSOARG00000012969,ENSOARG [illegible]	72	22	AMICA1,CD33,FCGR1B,GPR109B,FPR1,FPR2,HLA-DRB4,C19orf59,EMR1,EMR2,LPPR2,LILRB3,LILRB2,LILRB1,FCGR2A,SIRPB1,GPER,LRRC33,VSTM1,MS4A6A,P2RY2,GPR133	94

**S5**	DC surface signature	CSF2RB,TM2D2,CD1E,CD1B,PPAP2B,SLC7A8,CD1A,KMO,CD58,LRFN4,CCRL2,SIGLEC1,CD151,ADAM12,TM7SF4,SLC36A1,PDCD1LG2,PSEN2,GPR137B,C19orf28,TMEM158,PTGFRN,IL1R1,SLC6A6,SPINT2,LRP11,FZD5,TSPAN33,DIRC2,ALCAM,CD83,GJB2,GPNMB,ABHD12,CD9,EMP1,TFRC,DQA,LHFPL2,TACSTD2,COLEC12,JAG1,SUCNR1,TMEM51,TGFA,SLC38A6,ITPRIPL2,CALCRL,CLDN23,ATP1B2,GRINA,FPR3,RAMP1,ATP1B1,TREM2,SRD5A3,SLC41A2,FAM70A,LAMP2,ABCC3,DENND1B,P2RY6,NRP1,NRP2,CXCL16,PRRG4,CRIM1,GPR157,OLR1,CD274,SDC2,SLCO2B1,SLC7A11,ADAM9,ABCA6,MSR1,ACE,AGPAT3,SLAMF8,SLC1A3,OVAR-DQA1,OVAR-DQA2,OVAR-DQB1,OVAR-DQB2,TNFRSF11A	DC/antigen presentation	ENSOARG00000019095,ENSOARG00000001785,ENSOARG00000007307,ENSOARG00000007282,ENSOARG00000019354,ENSOARG00000007267,ENSOARG00000008426,ENSOARG00000020234,ENSOARG00000014203,ENSOARG00000002007,ENSOARG00000012241,ENSOARG00000008663,ENSOARG00000013525,ENSOARG00000001381,ENSOARG00000003605,ENSOARG00000014287,ENSOARG00000020252,ENSOARG00000013144,ENSOARG00000000248,ENSOARG00000005552,ENSOARG00000002898,ENSOARG00000018896,ENSOARG00000004145,ENSOARG00000020167,ENSOARG00000018737,ENSOARG00000012936,ENSOARG00000017089,ENSOARG00000012748,ENSOARG00000007550,ENSOARG00000008605,ENSOARG00000020808,ENSOARG00000020307,ENSOARG00000015485,ENSOARG00000017150,ENSOARG00000008349,ENSOARG00000009301,ENSOARG00000010523,ENSOARG00000003740,ENSOARG00000010392,ENSOARG00000011111,ENSOARG00000021133,ENSOARG00000010732,ENSOARG00000016547,ENSOARG00000004177,ENSOARG00000015381,ENSOARG00000015553,ENSOARG00000018990,ENSOARG00000009371,ENSOARG00000001744,ENSOARG00000001390,ENSOARG00000016898,ENSOARG00000013179,ENSOARG00000003559,ENSOARG00000015345,ENSOARG00000006714,ENSOARG00000017485,ENSOARG00000018434,ENSOARG00000007683,ENSOARG00000016840,ENSOARG00000010127,ENSOARG00000001740,ENSOARG00000021012,ENSOARG00000013509,ENSOARG00000004813,ENSOARG00000011065,ENSOARG00000014253,ENSOARG00000001816,ENSOARG00000014385,ENSOARG000[illegible]	73	12	PPAP2B,LRFN4,CD151,TM7SF4,C19orf28,FPR3,FAM70A,ACE,OVAR-DQA1,OVAR-DQA2,OVAR-DQB1,OVAR-DQB2	85
**S6**	CD4 T cell surface signature Th1-stimulated	F2R,TMEM200A,C6orf129,GPR19,LAG3,NDFIP2,TSPAN5,SV2A,GPR114	T/NK cells	ENSOARG00000016937,ENSOARG00000020015,ENSOARG00000020281,ENSOARG00000006528,ENSOARG00000000420,ENSOARG00000016064,ENSOARG00000020706	7	2	C6orf129,GPR114	9
**S7**	CD4 T cell surface signature Th2-stimulated	SLC37A3,GPR15,CD28,CLDND1,TMEM200A,SLC4A7,IL17RB,ITM2A,GPR171,SIT1,C6orf129,MAL,CD3G	T/NK cells	ENSOARG00000012780,ENSOARG00000001639,ENSOARG00000018277,ENSOARG00000017694,ENSOARG00000020015,ENSOARG00000014224,ENSOARG00000015876,ENSOARG00000000398,ENSOARG00000015348,ENSOARG00000011944,ENSOARG00000009256	11	2	C6orf129,MAL	13
**S8**	Naive B cell surface signature	LGR4,KCNE4,TSPAN13,UGT2A3,CD37,TRHR,CNTNAP3,PPAPDC1B,CCR6,CD200,GABBR1,EDNRB,GHSR,CLCA4,CNR1,CD72,P2RY14,BTLA,HRH4,PCDHB4,SLC38A11,C4orf34,PCDHB16,CXCR4,TMEM56,GRM5,PCDH20,CDH5,KCNJ16,JAM2,ITGB6,CHRNB4,PCDHB14,SLC24A1,SLC26A3,HTR1E,MUC15,OR1G1,IL13RA2,KCNMB3,FCRL3,IMPG2,GPR6,HHLA2,TAS2R7,HVCN1,NRXN3,FAM26F,HLA-DOA,BTC,SELE,GABRE,GPR116,HLA-DMA	B cells	ENSOARG00000014908,ENSOARG00000008934,ENSOARG00000009742,ENSOARG00000012851,ENSOARG00000014552,ENSOARG00000004933,ENSOARG00000019298,ENSOARG00000016848,ENSOARG00000000158,ENSOARG00000020744,ENSOARG00000014794,ENSOARG00000019995,ENSOARG00000011957,ENSOARG00000015318,ENSOARG00000019308,ENSOARG00000007300,ENSOARG00000005610,ENSOARG00000010462,ENSOARG00000001849,ENSOARG00000000206,ENSOARG00000015207,ENSOARG00000006967,ENSOARG00000001184,ENSOARG00000018844,ENSOARG00000018007,ENSOARG00000006903,ENSOARG00000020007,ENSOARG00000014295,ENSOARG00000004069,ENSOARG00000017220,ENSOARG00000020707,ENSOARG00000006866,ENSOARG00000018281,ENSOARG00000018906,ENSOARG00000020322,ENSOARG00000017389,ENSOARG00000002748,ENSOARG00000018074,ENSOARG00000008091,ENSOARG00000015138,ENSOARG00000011075,ENSOARG00000008475,ENSOARG00000007972	43	11	KCNE4,CNTNAP3,PPAPDC1B,PCDHB4,C4orf34,PCDHB16,TMEM56,GRM5,PCDH20,GPR6,GPR116	54
**S9**	Memory B cell surface signature	SSPN,CD52,CLDN17,SORCS3,EPHA3,OR11A1,TNFRSF17,CSMD1,NOX4,CHL1,CCR9,NCAM2,LINGO1,TACR3,JAM3,NKAIN4,CADM2,PCDHA3,TRHDE,NLGN1,FCGR2B,NLGN4Y,TAS2R10,ASTN1,GRAMD1C,GYPA,TNFRSF13B,CXADR,ADAM2,PTPRR,NPY1R,GJC1,ITM2C,FREM2,ABCA8,SCN1A,GLP2R	B cells	ENSOARG00000019951,ENSOARG00000004803,ENSOARG00000001063,ENSOARG00000008134,ENSOARG00000017153,ENSOARG00000005968,ENSOARG00000003844,ENSOARG00000009067,ENSOARG00000009514,ENSOARG00000015555,ENSOARG00000001848,ENSOARG00000010861,ENSOARG00000013611,ENSOARG00000011497,ENSOARG00000016962,ENSOARG00000014716,ENSOARG00000020726,ENSOARG00000010094,ENSOARG00000020317,ENSOARG00000015182,ENSOARG00000019513,ENSOARG00000002304,ENSOARG00000002101,ENSOARG00000020111,ENSOARG00000013669,ENSOARG00000000133,ENSOARG00000020673,ENSOARG00000009812,ENSOARG00000004852,ENSOARG00000004183	30	7	OR11A1,TNFRSF17,PCDHA3,NLGN4Y,GYPA,CXADR,ABCA8	37

**S10**	Resting dendritic cell surface signature	CLEC10A,CD1E,CD1B,CD1C,SLC7A8,CD1A,SERINC5,IGSF6,TREM2,TSPAN15,CCRL2,CD151,ADAM12,TM7SF4,SLC36A1,LRFN4,GPR137B,STAB1,CD86,IL1R2,IL1R1,IFNGR1,FZD1,FCER1A,FZD5,DIRC2,PSEN2,CSF1R,GPNMB,ABHD12,PTGFRN,KMO,EMP1,OVAR-DQB1,OVAR-DQB2,LHFPL2,SLC1A3,CCDC90A,LPCAT2,JAG1,PLXDC2,SUCNR1,TMEM51,SFXN3,SPINT2,SLC38A6,ITPRIPL2,ENG,MFSD1,TTYH3,SRD5A3,TACSTD2,TMEM144,FAM70A,ABCC3,PLXND1,DENND1B,ATP1B1,P2RY6,NRP1,SLC27A3,SLCO2B1,ITGAX,SLC26A11,CRIM1,CLDN23,PLD3,SLC7A11,CD9,ADAM9,ABCA6,ACE,SLAMF8,HLA-DMA,AGPAT9,CH25H	DC/antigen presentation	ENSOARG00000009867,ENSOARG00000007307,ENSOARG00000007282,ENSOARG00000019354,ENSOARG00000007267,ENSOARG00000017517,ENSOARG00000015971,ENSOARG00000001744,ENSOARG00000005596,ENSOARG00000014203,ENSOARG00000012241,ENSOARG00000008663,ENSOARG00000003605,ENSOARG00000003517,ENSOARG00000020112,ENSOARG00000013159,ENSOARG00000013144,ENSOARG00000000510,ENSOARG00000016629,ENSOARG00000007787,ENSOARG00000018896,ENSOARG00000020167,ENSOARG00000001381,ENSOARG00000006358,ENSOARG00000012748,ENSOARG00000007550,ENSOARG00000020252,ENSOARG00000008426,ENSOARG00000020808,ENSOARG00000017150,ENSOARG00000010729,ENSOARG00000018091,ENSOARG00000010523,ENSOARG00000000344,ENSOARG00000003740,ENSOARG00000010392,ENSOARG00000016225,ENSOARG00000005552,ENSOARG00000021133,ENSOARG00000010732,ENSOARG00000011387,ENSOARG00000002318,ENSOARG00000004887,ENSOARG00000001390,ENSOARG00000008349,ENSOARG00000005762,ENSOARG00000003559,ENSOARG00000013617,ENSOARG00000015345,ENSOARG00000009371,ENSOARG00000006714,ENSOARG00000017485,ENSOARG00000001265,ENSOARG00000011065,ENSOARG00000009130,ENSOARG00000001513,ENSOARG00000010127,ENSOARG00000004177,ENSOARG00000006607,ENSOARG00000014253,ENSOARG00000008605,ENSOARG00000001816,ENSOARG00000014385,ENSOARG00000007944,ENSOARG00000007972,ENSOARG00000003059,ENSOARG[illegible]	67	9	CD1C,CD151,TM7SF4,LRFN4,OVAR-DQB1,OVAR-DQB2,CCDC90A,FAM70A,ACE	76
**S11**	Activated (LPS) dendritic cell surface signature	CSF2RB,ANTXR2,IFI27,SLC31A2,TNFSF13B,TGFA,LY75,CD80,CD58,TMCC3,SLCO5A1,F11R,TTYH2,ITGB8,RNF144B,SGPP2,CALCRL,NINJ1,CD40,IL3RA,LAMP2,TMEM140,PDCD1LG2,KITLG,SLC43A2,NRP2,PRRG4,IL13RA1,SLC41A2,GPR157,IFI6,CD274,LRRC32,ELOVL7,COLEC12,EREG,SIGLEC1	DC/antigen presentation	ENSOARG00000019095,ENSOARG00000018889,ENSOARG00000014451,ENSOARG00000006395,ENSOARG00000005617,ENSOARG00000011111,ENSOARG00000019762,ENSOARG00000020234,ENSOARG00000015940,ENSOARG00000003495,ENSOARG00000009280,ENSOARG00000013043,ENSOARG00000010027,ENSOARG00000010155,ENSOARG00000020313,ENSOARG00000016547,ENSOARG00000008116,ENSOARG00000008369,ENSOARG00000007618,ENSOARG00000013179,ENSOARG00000001191,ENSOARG00000013525,ENSOARG00000015592,ENSOARG00000013025,ENSOARG00000018434,ENSOARG00000016840,ENSOARG00000016570,ENSOARG00000016898,ENSOARG00000001740,ENSOARG00000003341,ENSOARG00000013509,ENSOARG00000012979,ENSOARG00000007114,ENSOARG00000009301,ENSOARG00000014953,ENSOARG00000002007, ENSOARG00000019095,ENSOARG00000018889,ENSOARG00000014451,ENSOARG00000006395,ENSOARG00000005617,ENSOARG00000011111,ENSOARG00000019762,ENSOARG00000020234,ENSOARG00000015940,ENSOARG00000003495,ENSOARG00000009280,ENSOARG00000013043,ENSOARG00000010027,ENSOARG00000010155,ENSOARG00000020313,ENSOARG00000016547,ENSOARG00000008116,ENSOARG00000008369,ENSOARG00000007618,ENSOARG00000013179,ENSOARG00000001191,ENSOARG00000013525,ENSOARG00000015592,ENSOARG00000013025,ENSOARG00000018434,ENSOARG00000016840,ENSOARG00000016570,ENSOARG00000016898,ENSOARG00000001740,ENSOARG00000003341,[illegible]	36	1	LY75	37
