# Supplementary material for: Author Correction: System immunology-based identification of blood transcriptional modules correlating to antibody responses in sheep
Source: NPJ Vaccines. 2019 Feb 19;4:10. doi: 10.1038/s41541-019-0100-1 (PMC6381089; doi:10.1038/s41541-019-0100-1)
Supplement: Supplementary file 1 — Supplementary Information [file 41541_2019_100_MOESM1_ESM.pdf]

npj | Vaccines

www.nature.com/npjvaccines

ARTICLE OPEN

System immunology-based identification of blood transcriptional modules correlating to antibody responses in sheep

Roman Othmar Braun[1,2,3], Livia Brunner[4], Kurt Wyler[5], Gaël Auray[1,3], Obdulio García-Nicolás[1,3], Sylvie Python[1,3], Beatrice Zumkehr[1], Véronique Gaschen[6], Michael Hubert Stoffel[6], Nicolas Collin[4], Christophe Barnier-Quer[4], Rémy Bruggmann[5] and Artur Summerfield[1,3]

~~Inactivated vaccines lack immunogenicity and therefore~~ require potent adjuvants. To understand ~~the in vivo~~ effects ~~of adjuvants~~, we used a system immunology-based analysis of ovine blood transcriptional modules (BTMs) to dissect innate immune responses relating to either antibody or haptoglobin levels. Using inactivated foot-and-mouth disease virus as an antigen, we compared non-adjuvanted to liposomal-formulated vaccines complemented or not with TLR4 and TLR7 ligands. Early after vaccination, BTM relating to myeloid cells, innate immune responses, dendritic cells, and antigen presentation correlated positively, whereas BTM relating to T and natural killer cells, as well as cell cycle correlated negatively with antibody responses. Interestingly, ~~similar~~ BTM also correlated with haptoglobin, but in a reversed manner, indicating that acute systemic inflammation is not beneficial for early antibody responses. Analysis of vaccine-dependent BTM modulation showed that liposomal formulations induced similar responses to those correlating to antibody levels~~. Surprisingly, the~~ addition of ~~the~~ TLR ligands ~~appeared to reduce early immunological perturbations and mediated anti-inflammatory effects,~~ despite promoting antibody responses. When pre-vaccination BTM were analyzed, we found that high vaccine responders expressed higher levels of ~~many BTM relating to cell cycle, antigen-presenting cells, and innate responses~~ as compared with low responders. In conclusion, we have transferred human BTM to sheep and identified early vaccine-induced responses associated with antibody levels or unwanted inflammation ~~in a heterogeneous and small group of animals.~~ Such readouts are applicable to other veterinary species and very useful to identify efficient vaccine adjuvants, their mechanism of action, and factors related to low responders.

npj Vaccines (2018)3:41 ; doi:10.1038/s41541-018-0078-0

INTRODUCTION

In the past, vaccines were developed empirically requiring many animal studies with a long-term follow-up to assess vaccine efficacy. Furthermore, although the mechanisms of many vaccine adjuvants at the molecular and cellular level are clear, in vivo protection is much more complex and still difficult to understand and predict. Systems immunology offers powerful approaches towards dissecting the early innate immune response relating to potent adaptive immune responses in human.[1–3] This enabled to identify genes expressed in peripheral blood leukocytes within the first days after vaccination, which correlated to CD8 T-cell or antibody responses.[2,3] Nevertheless, a main problem with this relatively simple approach was the high heterogeneity of individual responders and the difficulty to understand immunological processes from individual gene information. For these reasons, a much more powerful analysis of transcriptomic data was developed by identifying gene sets such as blood transcriptional modules (BTMs) through large-scale data integration.[1,4] To this end, Li et al.[1] established human blood transcriptomes from 540 data series containing over 32,000 samples obtained from many disease-related studies to identify master networks of genes co-regulated and interacting in ~~cells of the~~ immune system during many physiological and pathological conditions. Certain BTM were demonstrated to correlate with adaptive immune responses in a vaccine type-specific manner enabling conclusions on immunological processes associated with desired vaccine responses.[1]

For foot-and-mouth disease (FMD), a devastating viral infection affecting cloven-hooved animals, traditional vaccine approaches are based on the formulation of inactivated antigen with conventional adjuvants such as oil-in-water emulsions.[5] In countries free of FMD, besides culling infected herds, vaccines can have an important role in the control of outbreaks. Nevertheless, the ability to induce early protection is an essential element of such vaccines. A problem associated with the elimination of FMD from endemic areas is the fact that current vaccines offer protection with a very limited duration of immunity.[6] A potential to improve such vaccines would be to enhance their ability to activate the innate immune system by targeting Toll-like receptors (TLRs), which can promote T-cell help and differentiation of B lymphocytes into memory or long-lived plasma cells.[7] TLR are a family of pathogen recognition receptors (PRR) typically expressed at high levels on specialized antigen-

[1]Institute of Virology and Immunology, Mittelhäusern, Switzerland; [2]Graduate School for Cellular and Biomedical Sciences, University of Bern, Bern, Switzerland; [3]Vetsuisse Faculty, Department of Infectious Disease and Pathobiology, University of Bern, Länggassstrasse 122, 3001 Bern, Switzerland; [4]Vaccine Formulation Laboratory, Department of Biochemistry, University of Lausanne, Lausanne, Switzerland; [5]Interfaculty Bioinformatics Unit and Swiss Institute of Bioinformatics, University of Bern, Bern, Switzerland and [6]Division of Veterinary Anatomy, University of Bern, Bern, Switzerland
Correspondence: Artur Summerfield (artur.summerfield@ivi.admin.ch)

Received: 10 January 2018 Revised: 07 July 2018 Accepted: 31 July 2018
Published online: 03 October 2018

presenting cells and are central for alerting the innate immune system in case of infection. Cell surface TLRs, such as TLR4, recognize microbial pattern molecules such as bacterial lipopolysaccharides, whereas endosomal TLRs such as TLR7 and TLR9 sense single-stranded RNA or microbial unmethylated DNA with many CpG motifs, respectively. In fact, TLR4 and TLR7 ligands were shown to strongly enhance antibody responses in the mouse model,[7] and were therefore selected for the present study.

Based on this, one aim of this work was to determine the potential of such TLR ligands to improve the immunogenicity of an inactivated vaccine in large animals using an FMD vaccine as a model. We used sheep as a model for an ungulate that is naturally susceptible to FMD virus (FMDV). Although in western countries small ruminants usually are not considered in vaccination program, the immune system of sheep and cattle are relatively similar and the use of sheep requires less space and financial resources. Furthermore, in certain areas of the world small ruminant represent the main FMD-susceptible livestock and can play an important role for spreading the infection during outbreaks.[8–10]

Our second important aim was to transfer human BTMs to the sheep model and identify gene sets correlating with antibody levels as well as vaccine side effects. We also successfully employed this bioinformatics pipeline to characterize vaccine-adjuvant-dependent modulation of the immune responses as well as the pre-vaccination transcriptome associated with high immune responses.

RESULTS

Antibody responses

Three groups of sheep were vaccinated with FMDV antigen formulated either in buffer (PBS), in liposomes (L) or in liposomes containing TLR4 and TLR7 ligands (L(TLRL)), and the ability of the vaccines to induce an early neutralizing antibody response was tested (Fig. 1~~a~~). For the physicochemical characterization of the vaccines see Supplementary Fig. 1. ~~Although animals from all groups responded to vaccination, at day 7 p.v. (D7) only the serum neutralization titers (SNTs) of those receiving liposomal formulations raised above 1:20, which is generally considered to be associated with protection.[6] Over the time observed, most animals stayed above this critical titer in the L(TLRL) group. Nevertheless, due to the high variation in the response of individual animals, statistically significant differences were only found when the L (TLRL) was compared with the PBS group (Fig. 1a).~~

Vaccine-induced haptoglobin and transcriptional modulations

As liposomal formulations caused lameness in some animals for 1–2 days after injection, we also quantified systemic inflammation by measuring serum haptoglobin, an acute-phase protein induced in the liver by pro-inflammatory cytokines,[11] and which represents a sensitive marker of inflammation following sheep vaccination.[12] Our results demonstrate that liposomal formulations were indeed associated with an acute-phase response peaking at D3 and still detectable in most animals at D7 (Fig. 1~~b~~). Interestingly, the TLRL did not enhanced but actually reduced this response ($p = 0.0$~~13~~ for L versus L(TLRL) at D3).

Also at the transcriptional level, the liposomal formulations caused a stronger perturbation of the immune system, but this was dampened by the addition of TLRL (Fig. 1~~c~~). For example, when comparing and peripheral blood mononuclear cells (PBMCs) before and at D3 (D0 vs. D3), PBS induced the significant upregulation of ~~116~~ genes, L of ~~216~~ genes, and L(TLRL) only of ~~7~~ genes. Nevertheless, at D7, the TLRL-adjuvant group had clearly the most up- or downregulated transcripts (Fig. 1~~c~~ and Supplementary Fig. 2). We found ~~53~~ common genes between the L and L(TLRL) groups, and ~~63~~ common genes for the L and PBS groups ~~(Supplementary Fig. 2) but no~~ common genes for all three formulations.

Individual transcripts correlating with neutralizing antibody titers

Individual changes in gene expression were calculated and correlated with the SNT on day 7, 14, and 28 p.v. (Supplementary Fig. 3). For the D0 vs. D3 differentially expressed genes, 26 transcripts (ZNF646, STIM2, C9orf3, NPEPPS, SDR39U1, HEXDC, NUCB2, GHDC, LSR, MYO1E, TRAPPC6A, DCAF12, ABCC2, ZDHHC24, TRIM41, GZMA, NAGK, GMNN, OLFML3, MYO7A, TNFSF13B, GGA3, ASB8, ARNTL, GNG3, PLA2G12A) were identified to correlate with the SNT of all three time points (7, 14, and 28 days). For the D0 vs. D7 differentially expressed genes, six transcripts were found to correlate with SNT of all three time points (CHPT1, RPL12, RNASEL, CCDC93, MFAP2, MYO1E). Only MYO1E was correlating at D3 as well as D7 with the SNT at all time points.

Translation of human BTM to the sheep

While some of the genes identified above are clearly linked to antibody responses such as TNFSF13B (B-cell-activating factor), the potential involvement of most genes in promoting antibody responses remained elusive. We therefore employed gene set enrichment (GSEA) using BTM developed in the human system. These have been demonstrated to provide a comprehensive picture of human immune responses correlating with adaptive immune responses induced by different classes of vaccines.[1] ~~Figure 2a shows the number of genes in the human and corresponding sheep modules, and Fig. 2b the coverage of the BTM using annotated sheep genes.~~ The list of all BTM with their gene compositions can be downloaded from the Supplementary Data. ~~At the time of writing this manuscript, the incomplete annotation of the sheep genome enabled an overall 76.79% coverage of the BTM (mean value).~~

BTM correlating with antibody responses

The correlation of BTM with antibody responses measured at 7, 14, and ~~21~~ days p.v. were determined for the D3 and D7 transcriptome (Fig. ~~3a,b, respectively~~). Although some BTM correlated only with one of the time points p.v., other correlated with two or all of the time points analyzed. This was expected as some animals with low early antibody levels caught up at day 28 p.v. (Fig. 1~~a~~). Nevertheless, for D3 the BTM M209 (lysosome) and M23 (RA, WNT, CSF receptor network) positively correlated with the SNT at all time points. Furthermore, despite the variation in antibody responses over time, a number of BTM were found that negatively correlated to antibody responses measured at all three time points. These were mainly BTM related to cell proliferation (M4.10, M37.3, M10.0) and T/natural killer (NK) cells (M7.2, M61.2, ~~M35.1,~~ M7.3).

Antibody-correlating D7 BTM were reduced in numbers (Fig. ~~3~~b). Nevertheless, some BTM were found again, ~~such as~~ M146 (MHC-TLR7-TLR8 cluster, positive correlation), M35.1 (signaling in T cells, negative correlation)~~,~~ and M2~~5~~0 (~~spliceosome,~~ negative correlation).

To enable an easier interpretation of the BTM results, we classified them into six BTM families (Table 1 and Supplementary Excel file "BTM composition and families"). To this end, BTM were attributed to immune cell types or immunological processes, for which we decided to make large families to obtain a simplified synopsis of early immune responses correlating to antibody titers. As an example, the BTM family "myeloid cells/inflammation" was composed of all BTM's relating to monocytic cells, neutrophils, and other elements of inflammatory reactions. We also included BTM related to coagulation and complement activation, as these are processes induced during inflammation (Table 1). The result of

a Study design

Groups and adjuvants	Antigen	Route	Sheep per group	Age	Gender	PBMC Sampling	Serum sampling
1. None (PBS)<br>2. Liposomes<br>3. Liposomes with MPLA & guardiquimod	40µg inactivated FMDV particles	One injection i.m.	6	nine 5-6 month old and nine 12-20 month old, equally distributed	Five female and one castrated male in each group	0, 3, 7 days p.v.	0, 3, 7, 14, 28 days p.v.

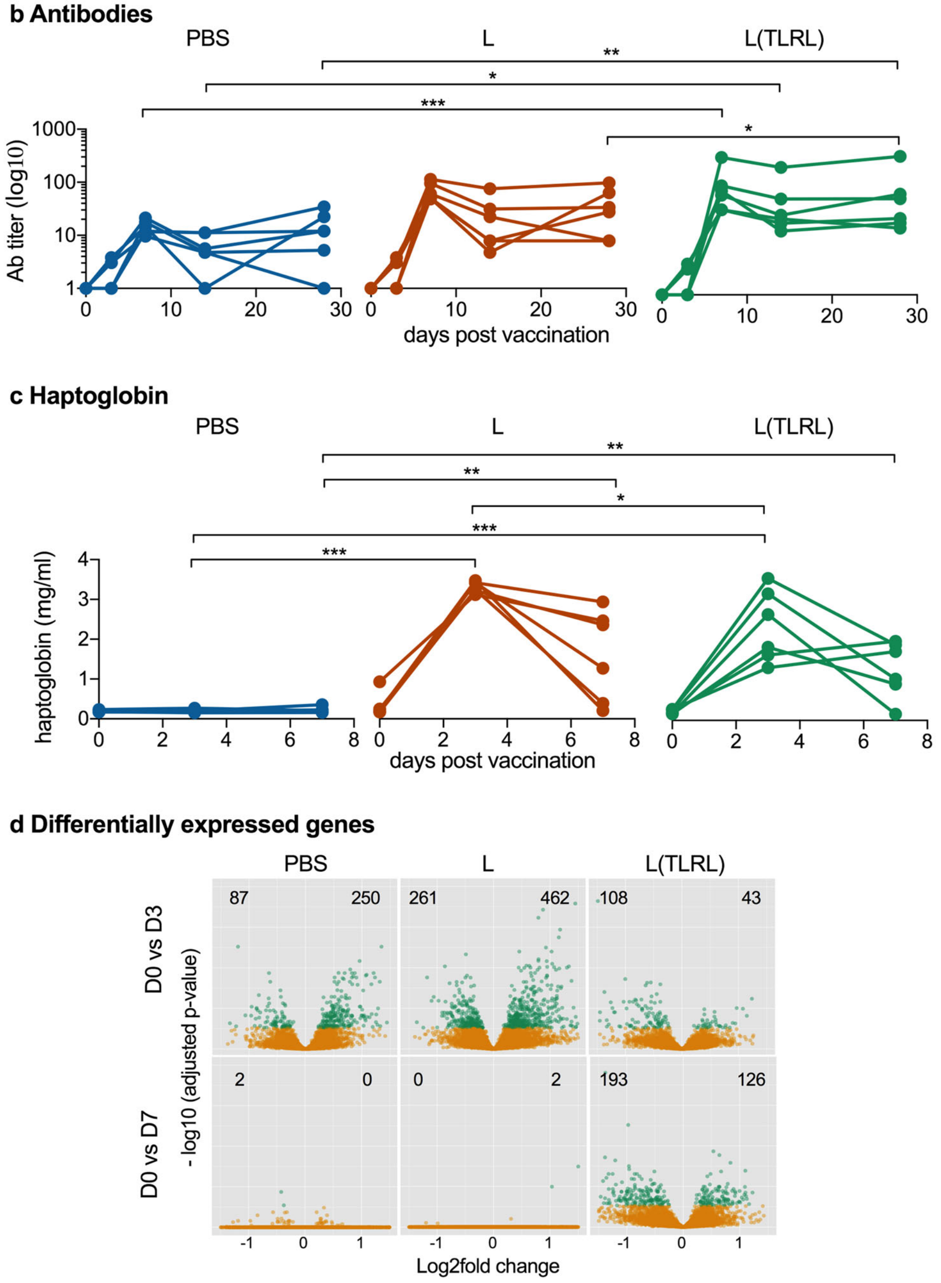

Fig. 1 Neutralizing antibodies, haptoglobin and differentially expressed genes in PBMC induced by vaccination. **a** Serum neutralizing antibody titers following vaccination of sheep with antigen in phosphate buffered saline (PBS), antigen formulated in liposomes (L) and antigen formulated in liposomes with TLRL (L(TLRL)). ~~The dotted line represents the titer predicted to be protective.~~ **b** Vaccine-induced haptoglobin responses. **c** Vulcano plots showing differentially expressed genes which were up- or downregulation on day 3 (D3) and day 7 (D7) compared with before immunization, respectively. Green dots represent significant genes (~~$p < 0.01$~~). Digits in the plots indicate the number of significantly down or upregulated genes. In **a** and **b**, statistical significant differences between two groups were calculated using unpaired two-way ANOVA followed by Tukey's multiple comparisons test (***$p < 0.001$; **$p < 0.002$; *$p < 0.033$)

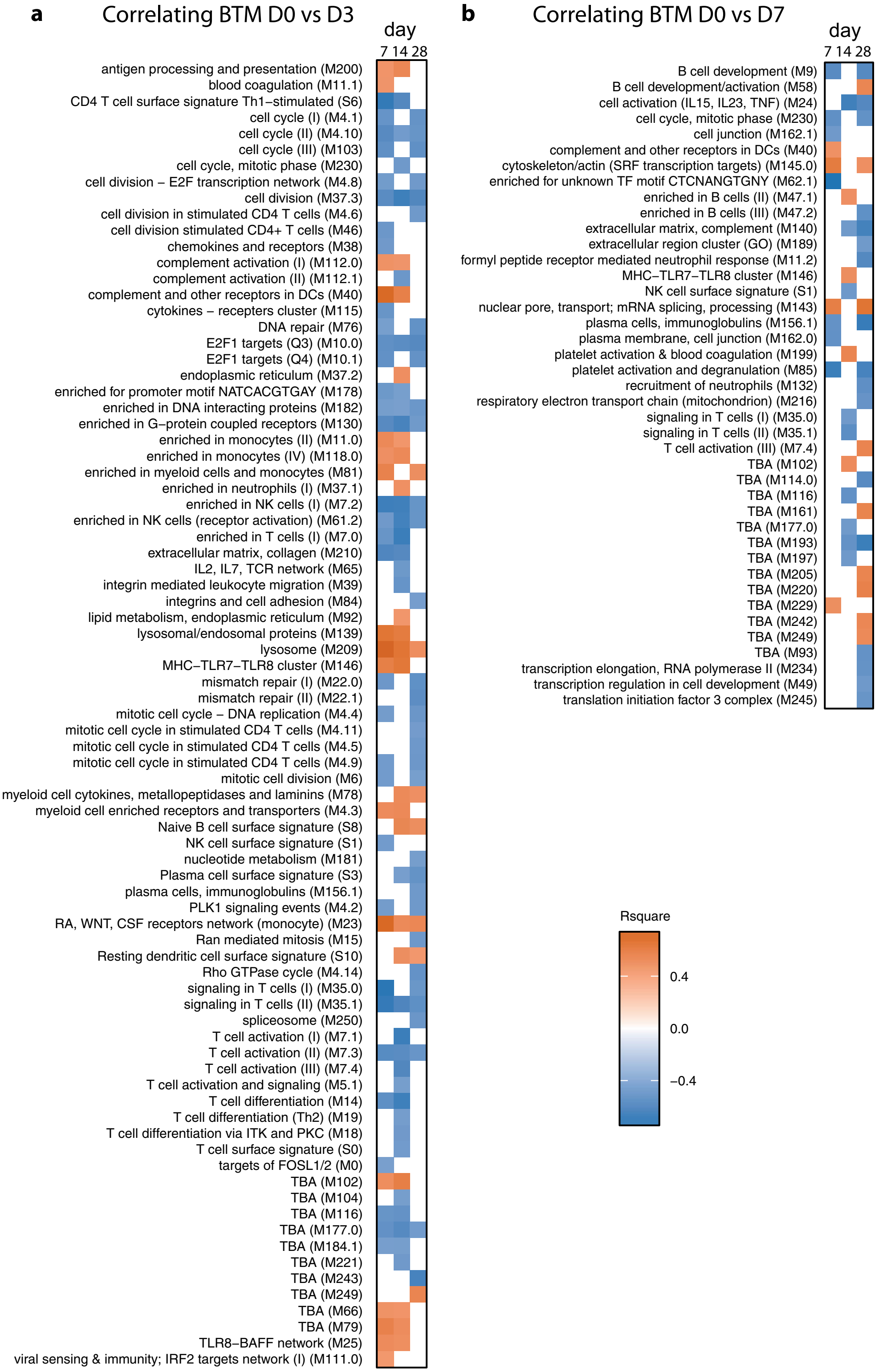

~~**Fig. 2** Translation of human blood transcriptional modules to the ovine system. **a** Number of human and sheep genes assigned to 349 BTM (based on annotation of the sheep genome at the time of analyses). **b** Coverage of sheep genes assigned to BTM compared with the original composition.[1]~~

such an analysis for the correlating BTM is shown in Fig. 4a and demonstrate that the D3 dominant BTM families positively correlating with antibody responses were "myeloid cells/inflammation" and "DC/antigen presentation," whereas the BTM families "T/NK cells" and "cell cycle" were the most prominent to negatively correlate. This dominance was seen in terms of the number of correlating BTM's attributed to the family for each of the analyses. The polar plots created for day 7 did not enable the identification of clearly dominant BTM families because of the reduced number of correlating BTMs. Interestingly, at this later

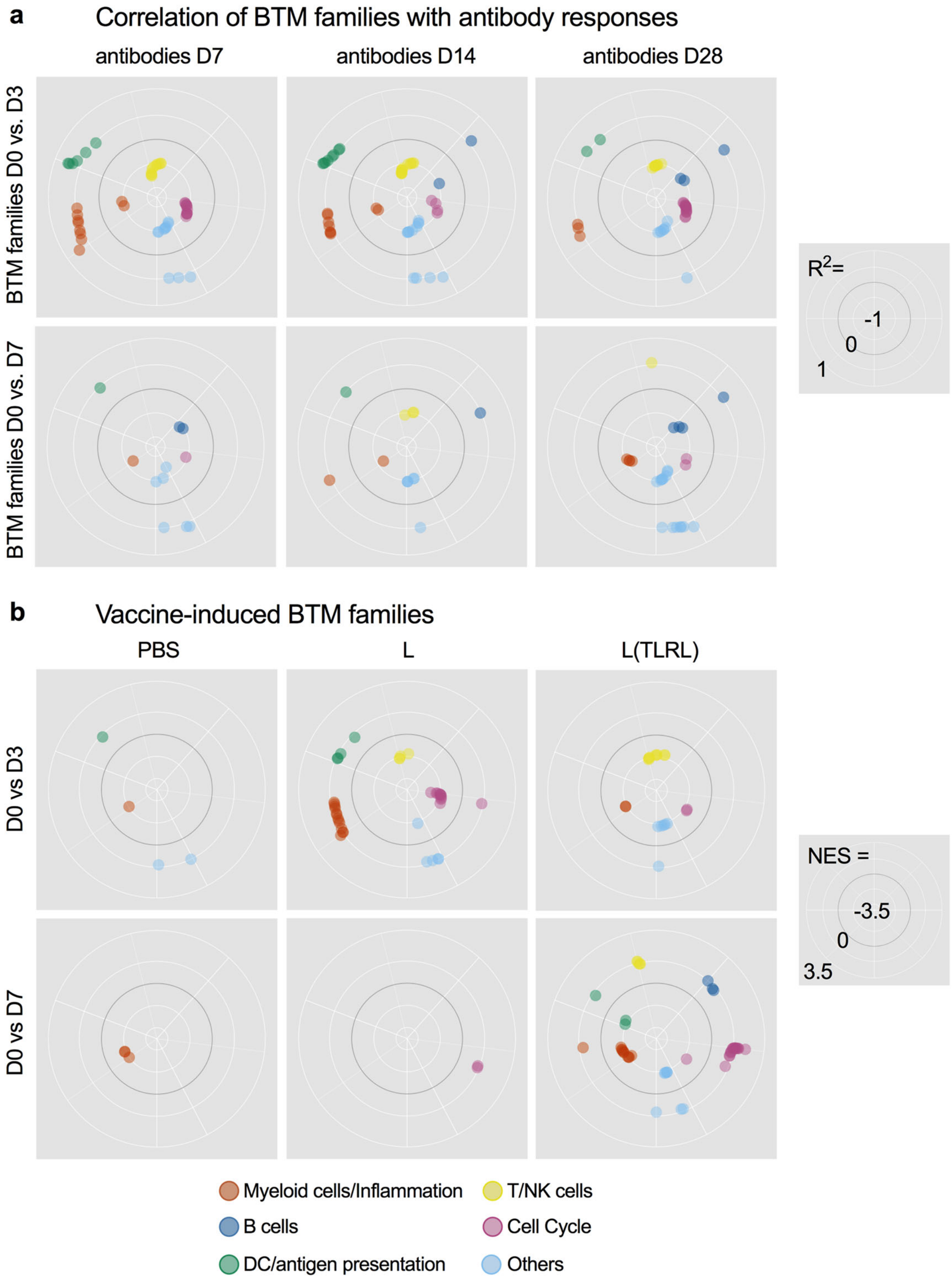

~~**Fig. 3** BTM correlating to serum neutralizing antibody titers. **a** Heatmap showing the correlation of D3 BTM with antibody levels on day 7, 14, or 28 after immunization. Animals from all groups were included ($n = 18$, cutoff $p < 0.05$). **b** Heatmap showing the correlation of D7 BTM with antibody levels in analogy to **a**~~

time point the BTM family "myeloid cells/inflammation" was negatively correlating with antibody levels.

Vaccine-induced BTM's

We next calculated BTM's significantly induced by the different vaccines with the aim to compare these responses with the antibody-correlating BTM, and to determine the effects of formulation and TLRL. The results are shown as BTM families in Fig. 4b and as heatmap for individual BTM in Supplementary Fig. 4. On D3~~, both~~ liposomal formulations strongly promoted "myeloid cells/inflammation" and "DC/antigen presentation" but downregulated "T/NK cells" and "cell cycle" BTM families.~~ The~~ addition of the TLRL ~~reduced~~ "myeloid cells/inflammation" and "DC/antigen presentation" BTM. ~~The PBS group had a milder effect~~

Table 1. Definition and composition of BTM families

BTM families	Immune cells and processes	BTM included
Myeloid cells/ inflammation	Myeloid cells, innate/inflammatory response (inflammatory cytokines and chemokines), leukocyte migration, coagulation and platelet activation, complement activation	M0, M4.3, M4.15, M11.0, M11.1, M11.2, M16, M21, M23, M24, M27.0, M27.1, M29, M33, M37.1, M38, M39, M45, M53, M59, M73, M78, M81, M85, M86.0, M86.1, M88.0, M91, M112.0, M112.1, M115, M118.0, M118.1, M132, M163, M199, S4, M30, M32.0, M32.1, M37.0, M42
DC/antigen presentation	DC surface (resting and activated), antigen processing and presentation, DC activation	M5.0, M25, M28, M40, M43.0, M43.1, M50, M64, M67, M71, M87, M95.0, M95.1, M119, M138, M139, M146, M165, M168, M200, M209, S5, S10, S11
T/NK cells	NK and T-cell surface, differentiation, activation, signaling, co-stimulation, and proliferation	M4.5, M4.6, M4.9, M4.11, M5.1, M7.0, M7.1, M7.2, M7.3, M7.4, M12, M14, M18, M19, M35.0, M35.1, M36, M44, M46, M52, M61.0, M61.1, M61.2, M65, M126, M157, M223, S0, S1, S6, S7
B cells	B cells, BCR signaling, B-cell development and differentiation, plasma cells, immunoglobulins	M9, M47.0, M47.1, M47.2, M47.3, M47.4, M58, M69, M83, M123, M156.0, M156.1, M217, S2, S3, S8, S9, M152.0, M152.1, M152.2, M182
Cell cycle	Cell cycle, proliferation, DNA repair, nucleotide and amino acid metabolism, splicing	M76, M103, M4.10, M4.12, M4.0, M4.1, M4.2, M4.4, M4.7, M4.8, M6, M8, M10.0, M10.1, M20, M22.0, M22.1, M31, M32.2, M32.4, M37.3, M49, M144, M167, M175, M181, M230, M250, M15, M143, M154.0, M169
IFN type-I	Nucleic acid sensing, IFN type-I induction, and IFN response signature	M13, M68, M75, M111.0, M111.1, M127, M150, M158.0, M158.1

~~on upregulated BTM families and, more interesting, an opposing effect on the "T/NK cells" and "cell cycle" families. Interestingly, at D3 and D7 the "B-cell" family was dominantly upregulated only in the TLRL group. This could be relevant with respect to the ability of TLRL to enhance antibody responses, since "B cells" BTM were found to positively correlate with antibody responses (Fig. 4a). In all vaccine groups, an opposing modulation was found for "myeloid cells/inflammation," "DC/antigen presentation," and "cell cycle" BTM families, when D7 was compared with D3.~~

~~Inverse correlation of BTM to the acute-phase response compared with that of antibody responses~~

To differentiate innate immune responses associated with antibody responses or unwanted inflammation, we calculated the correlation of BTM with the haptoglobin responses (Fig. 5 and Supplementary Fig. 5). ~~At D3,~~ negative correlations were found for the "myeloid cells/inflammation" and "DC/antigen presentation" but positive correlation for the "T/NK cells" and "cell cycle" BTM families. ~~As this represents a reverse correlation compared with that found for the antibody levels, we conclude that acute-phase reactions likely indicate unwanted inflammatory side effects. Nevertheless, this conclusion was not possible for D7, when most BTM showed a negative correlation.~~

Pre-vaccination BTM associated with antibody levels

Considering the heterogeneity of vaccine-induced antibody responses (Fig. 1a), we tested ~~is~~ certain BTM would be associated with higher levels of antibody response. To this end, in each vaccine group animals having an SNT at day 28 of > 50% above the median were defined as "high responders" ($n = 6$) and those with < 50% of the median as "low responders" ($n = 6$). A comparison of high and low responders using GSEA demonstrated that ~~16~~ BTM related to cell cycle and DNA repair, ~~seven~~ BTM related to myeloid cells, ~~six BTM related to DC, and six~~ BTM related to inflammation and innate immune responses were expressed at relatively higher levels in the "high responders" (Fig. 6a). ~~Only a total of five~~ BTM ~~were~~ higher in the "low responder" animals~~, which included BTM related to interferon (IFN) type-I and B-cell responses. Figure 6b shows a heatmap for the BTM "monocyte surface signature (S4) as an example of this analyses~~.

DISCUSSION

The present study adapted BTM originally developed for human blood to sheep PBMC to understand vaccine-induced responses correlating with antibody and acute-phase responses. For efficient delivery to antigen-presenting cells, we employed synthetic liposomes as vaccine platform and tested the effect of a combination of TLR4 and TLR7 ligands on antibody and acute-phase protein response. These ligands were selected based on their synergistic potentiation of antibody responses in a mouse model.[7] Specifically, the TLR7 ligand Gardiquimod was selected based on its ability to induce proliferation and induce IFN-α in PBMC,[13,14] and the TLR4 ligand monophosphoryl lipid A (MPLA) based on its potent triggering of nuclear factor-κB signaling, which promotes pro-inflammatory responses in mononuclear phagocytes.[15,16] Furthermore, MPLA is clinically approved and was also recently reported to induce a similar response in sheep compared with human PBMC.[17] The vaccination of sheep was successful in terms of induction of neutralizing antibody titers ~~above a titer of 1:20, which has been proposed as a critical threshold for protective immunity.[6] Also, the addition of the TLRL significantly enhanced the number of animals reaching titers above this threshold. Nevertheless, due to high variability between the animals, no statistically significant differences in the antibody titers where found when comparing the L with the L (TLRL) groups.~~ Nevertheless, it is clear that the present vaccine still requires significant improvements, and thus future studies focussing on the effects of formulation, TLR ligand selection and dose in sheep are needed.

Interestingly, although the increase in antibody responses induced by the TLRL was ~~disappointing,~~ it had a clear effect on the expression of individual genes. Strikingly, the overall perturbation of gene expression was reduced at D3 in terms of differentially up- or downregulated genes when compared with the L group. Furthermore, on day three p.v., the PBS group shared many upregulated genes with the L, but not with the L(TLRL) group. At day 7, this was even more evident in that differentially regulated genes were found almost exclusively in the TLRL~~-adjuvanted~~ group. Also, comparing the vaccine groups using BTM confirmed clear effects of the TLRL. The reduction of upregulated "myeloid cells/inflammation" and DC/antigen presentation" BTM at D3 together with the above reduced perturbation of individual

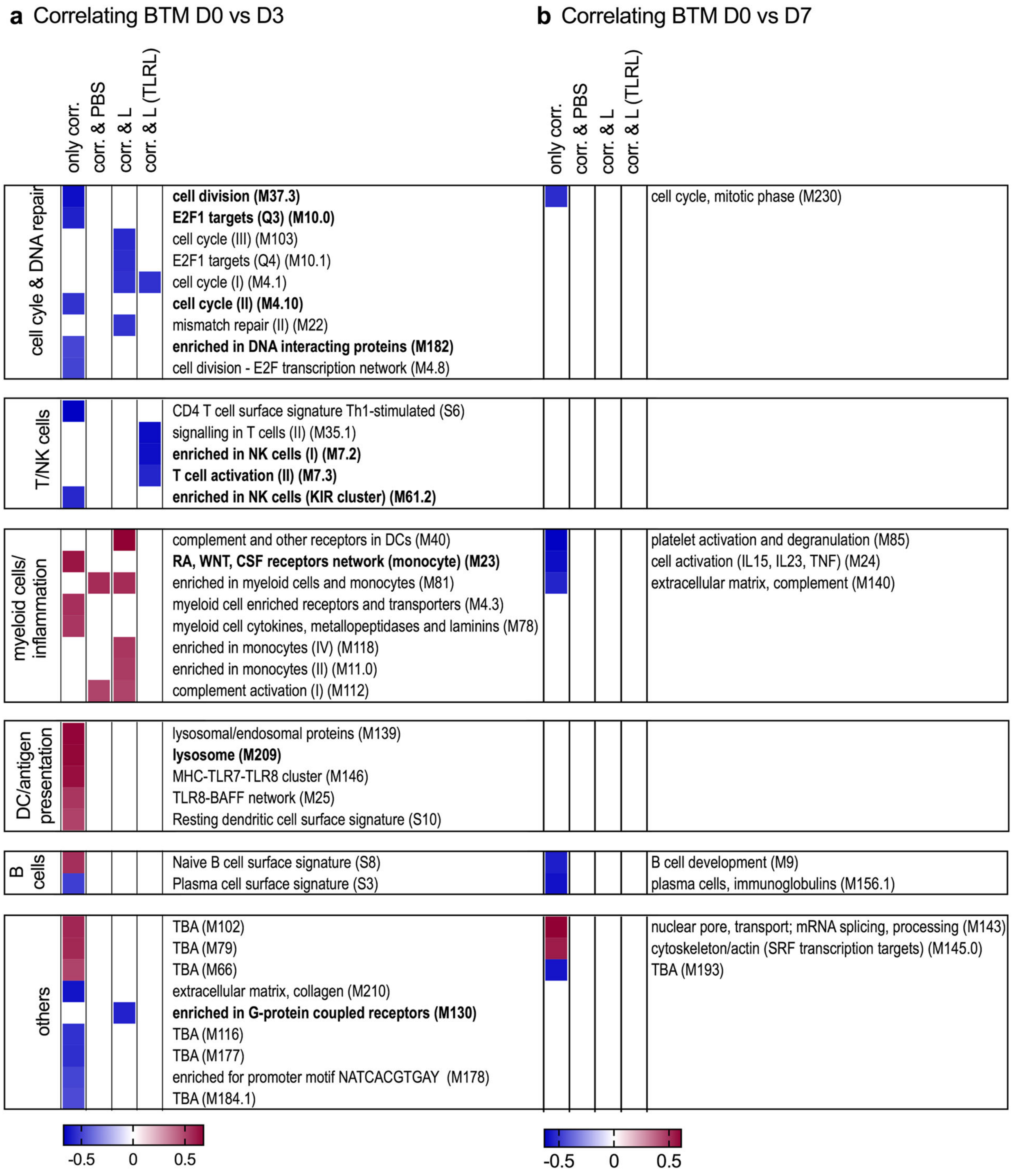

Fig. 4 BTM families correlating with antibody responses and induced by the vaccines. BTM families were created as described in Table 1. **a** The polar plots show the correlation coefficients for BTM of the module families with significant positive or negative correlation to neutralizing antibody levels in serum ($p < 0.05$). BTM for the D3 (upper plots) and D7 (lower plots) transcriptome are shown. **b** Vaccine-dependent BTM modulations were calculated as normalized enrichment scores (NES) using GSEA. The significantly ($p < 0.05$) modulated BTM were grouped as BTM families as in **a**. The data are shown was calculated for each vaccine group with the D3 (upper plots) and D7 (lower plots) transcriptome

genes and the reduced induction of haptoglobin indicates an anti-inflammatory effect of the TLRL. Future studies are required to understand how this is induced and if it is an effect of one of the TLRL or their combinations. The fact that at D7 the L(TLRL) group showed the strongest perturbations, both at the individual gene and BTM level, demonstrates that this is not a simple suppressive effect but that rather long-lasting immunomodulatory circuits are induced.

The analysis of genes correlating positively or negatively with antibody titers revealed a relatively large number of genes. We focused mainly on those found to correlate at least for two different time points to increase the chance to have truly relevant genes. Nevertheless, for most of the correlating genes, their role in immune responses is unclear, with the exception of TNFSF13B representing BAFF. Furthermore, only few of the correlating genes matched those identified in PBMC from humans vaccinated with

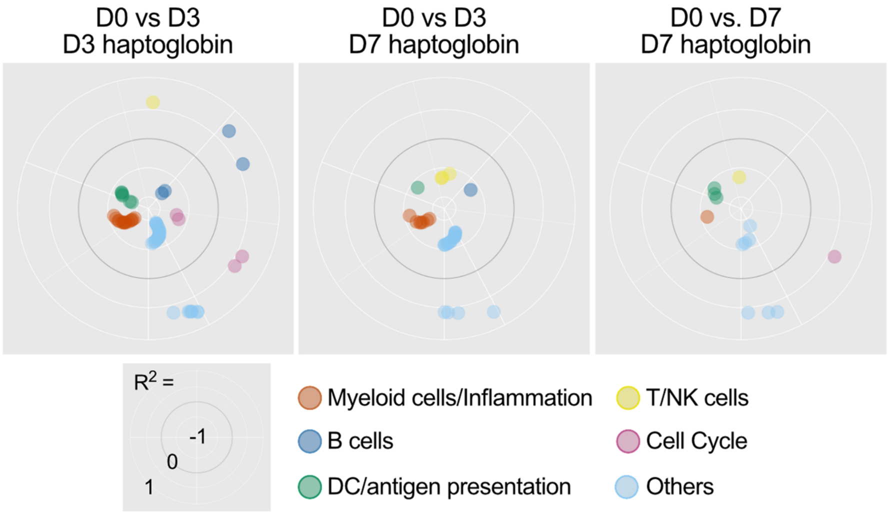

Fig. 5 BTM families correlating to vaccine-induced haptoglobin levels. BTM families were created as described in Table 1. The polar plots show correlation coefficients for module families with significant ($p < 0.05$) positive or negative correlation to serum haptoglobin levels. BTM families for the D3 (upper plots) and D7 (lower plots) transcriptome are shown

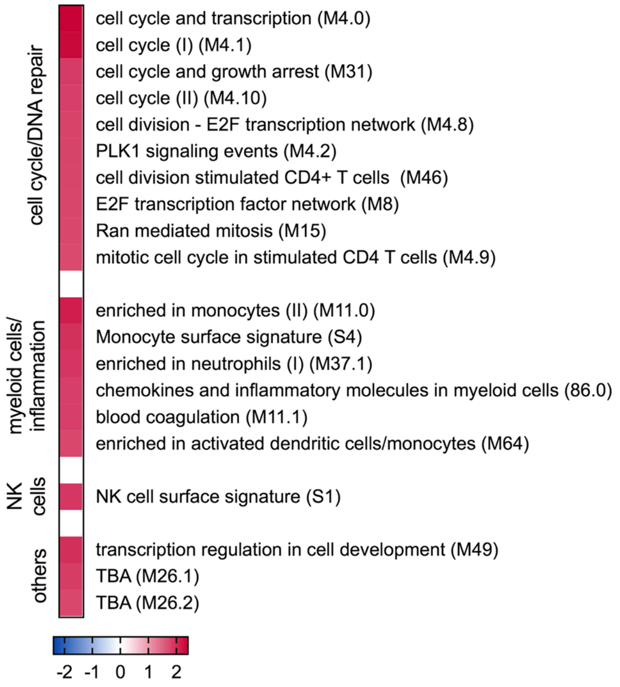

Fig. 6 Pre-vaccination BTM associated with antibody levels. **a** GSEA was used to determine the BTM enriched in "high responders" ($n = 6$, red) and "low responders" ($n = 6$, blue). The colors indicates the NES. **b** Heatmap for the BTM "monocyte surface signature" (S4) as an example of the analysis in a. Each square represents the indicated gene expression for individual animals (same color code as in **a**)

an inactivated viral vaccine.[2] For these reasons, we employed the BTM as a much more powerful bioinformatics analysis of transcriptomic data. BTM represent master networks of immunological genes strongly co-regulated and interacting during many physiological and pathological conditions.[1] This approach permitted to identify many BTM expressed on D3 and D7 correlating with antibody responses on day 7, 14, and 28 p.v. Remarkably, many of the correlating BTMs were also found in human vaccination studies using protein-based or inactivated vaccines. Prominent amongst these are positively correlating BTM relating to monocytes such as M11.0, M23, and M118,[1,18–20] to complement activation such as M112,[1,20] to TLR/antigen presentation such as M146 and M25[1,18,20], and to B cells and plasma cells such as M47, M156, and S3.[18] Among prominent negatively correlating modules appearing in both human and sheep vaccination studies are the NK cell signature BTM M61.1, M61.2, and M61.3[18,20,21], and less frequent M250 "spliceosome".[1] In sheep, many T-cell modules including CD4 T-cell BTM (M4.X) and general T-cell activation modules (M7.X) were negatively correlated with antibodies. Nevertheless, the various reports on human studies indicate that this can strongly vary depending on the time point being analyzed, the vaccine employed, the number of vaccinations, and the age;[1,20,21] although M7.1 and M7.2 were often negatively correlated with antibody response salso in human studies.[1,18,20,21] For the cell cycle modules which were also among the BTM with a negative correlation in sheep, this was rarely found in the human studies using protein-based vaccines, indicating possible species differences or alternatively a specific effect of the liposomal vaccine employed in the present study. Clearly, the correlating BTM response strongly depends on the type of vaccine.[1] Altogether, we propose that the common correlating BTM found for several different inactivated/protein-based vaccines in both species could be particular robust measurements. From our data, we also conclude that measuring the transcriptome early after vaccines provides more correlating BTM and, therefore, more information regarding the effects of the vaccine on the immune system.

For the immunological interpretation of these results, it must be considered that blood represents only a transit compartment for immune cells. As a result of vaccine-induced innate immune responses, haematopoiesis of myeloid cells and DC is likely to be enhanced explaining the increase of many modules relating to these cell types. Furthermore, it is obvious that despite the intramuscular injection, the vaccines cause systemic inflammatory effects, some of which obviously correlate with antibody responses. These are not only a number of modules relating the DC and antigen presentation but also to complement activation and blood coagulation. With these reactions, it is also possible to explain the negative correlation of T/NK cell modules, as these cells are very quickly recruited to lymphoid tissues following acute innate immune response such as virus infections.[22] The negative correlation of cell cycle BTM could be related to the same mechanisms as T cell represent the main cell fraction in PBMCs

and many such BTM~~s~~ were actually related to T cells, in particular CD4 T cells.

An important question arising from such studies is whether the above BTM represent mechanistically relevant modules or~~, on the contrary,~~ modules that also reflect unwanted side effects of strong adjuvants. To address this question, we performed two additional analyses, which were to compare the correlating BTM with the vaccine-induced BTM, as well as to identify BTM correlating with an acute-phase protein. ~~Although the overall pattern of BTM modulation was similar for the liposomal formulations on D3, the BTM correlating to the haptoglobin levels were opposite. In fact, the liposomal vaccines induced an upregulation of "myeloid cells/inflammation," "DC/antigen presentation," "B cells" BTM, and a downregulation of "T/NK cells" and "cell cycle" BTM, whereas the correlation of these BTM families to the haptoglobin response was inverse. This suggests that the way the vaccine modulates these BTM early after injection would be rather beneficial to the immune response and not directly linked to vaccine side effects. We also performed a correlation analysis of haptoglobin and antibody responses, and found neither evidence for a positive nor for a negative correlation. Nevertheless, at D7 many of the induced BTM were now relatively downregulated and this change was only partially recapitulated by the D7 BTM correlating to the antibody levels. It could therefore be speculated that some of these long-lasting perturbations of the immune system could be related to unwanted inflammation rather than vaccine potency. More studies with different kinds of adjuvants will shed light on this question.~~

~~The main difference with the PBS group was that it did not impact the "T/NK cell" BTM and had an opposite effect (upregulation) on the "cell cycle" BTM. We speculate that in the absence of liposomal formulation the inflammatory responses and the presence of antigen-loaded activated DC in draining lymph nodes could be reduced explaining a reduced recruitment of these cells to lymphoid tissue.~~

Finally, we also tested whether different pre-vaccination expression levels of BTM can be identified in low or high antibody-producing sheep. Our data indicate that this ~~is~~ possible even with relatively small group sizes. The relative upregulation of cell cycle, monocyte, DC and innate/inflammatory response BTM in the "high responders" would indicate that the immune system of those animals is in a kind of pre-activated status. ~~Nevertheless, this status was different from that induced by the TLR-adjuvanted vaccines which induced an opposite regulation of cell cycle, T/NK cells, and B-cell BTM.~~ A possible explanation for the combination of increased levels of myeloid cell/DC and cell cycle BTM could be increased hematopoiesis and egress of cells from the bone marrow and thymus. Future studies could aim at inducing such a status to enhance vaccine-induced responses.

Obviously, there many other possibilities to analyze gene sets and a choice from a multitude of possible gene sets, including pathways and molecular network-based gene sets,[23,24] must be made. An example for a different approach selected in cattle is the study by Laughlin et al.[25] using established canonical pathways and gene ontology networks analyzed using a method called Dynamic Bayesian Gene Group Activation. Although many of the gene sets identified to correlate are related to those found using the BTM method, a direct comparison is difficult. To enable a comparison of our data with a relatively large set of human vaccination data, we selected the BTM method for the present study.

In conclusion, the present work has developed a system immunology readout in the ovine model composed of many immunologically relevant gene expression networks, which correlate with antibody responses. We anticipate that this is applicable to other species and will be very useful to both the identification of efficient vaccine adjuvants causing little unwanted side effects and to find factors related to low vaccine responders.

METHODS

Vaccine preparation

The TLR4 ligand MPLA was from Avanti Polar Lipids (USA) and the TLR7 ligand guardiquimod from Chemdea (USA). DPPC, DCChol, and PHAD were purchased from Avanti Polar Lipids; Hepes from Applichem; sucrose, chloroform, and ethanol from Sigma-Aldrich (Switzerland). Extruder membranes of 400 and 200 nm pore size, as well as draining disks, were purchased from VWR International (Switzerland). Small-scale liposome formulations for evaluation were prepared as follows. DPPC and DCChol stocks were prepared at 50 mg/ml in methanol/methylen-chloride 1:1 (Sigma-Aldrich). Sixteen milligrams of total lipid mix was prepared and evaporated in an Eppendorf Vacufuge. Lipids were resuspended in 1 ml 20 mM HEPES, 10% sucrose buffer on a shaker for 60 min. Liposomes were serially extruded using an Avanti Mini Extruder (Avanti, USA) with 400, 200, and 100 nm membranes (Avanti). For each filtration step, liposomal solutions were passed 11 times. Large-scale liposomal formulation for the in vivo vaccine trial were made as follows. A solution of DPPC (2.6 mL) at 30 mg/mL in chloroform (78.5 mg) and 1.9 mL of a solution of DCChol at 30 mg/mL in chloroform (57.3 mg) were mixed in a 50 ml round-bottom flask. The solvent was evaporated with rotary evaporator (Laborota 4001, Heidolph) at 40 °C. The lipid film was rehydrated with 8.5 ml of pre-warmed (40 °C) 20 mM Hepes buffer containing 10% sucrose, pH 7.4, to obtain a lipid concentration (DPPC + DCChol) of 16 mg/ml, a DPPC concentration of 9.2 mg/ml, and DCChol concentration of 6.7 mg/ml. The mixture was magnetically stirred for 25 min and incubated for 5 min at room temperature (RT). The mixture was inserted into an extruder (T.001, Northern Lipids, Inc.) equipped with a thermo barrel of 10 ml and equilibrated at 50 °C. After five extrusion cycles with a filter of pore size of 400 nm and five cycles with a filter of 200 nm, the liposome suspension was filtered through a 0.22 µm filter to recover 7.5 ml of the sterile product. The filtered product was flushed with a nitrogen stream and stored at 4 °C.

Cationic liposomes containing gardiquimod were prepared with the same procedure by rehydrating the lipid film with 8.5 ml of pre-warmed (40 °C) 20 mM Hepes buffer containing 10% sucrose and gardiquimod at 1 mg/ml, final pH 7.4. Cationic liposomes containing MPLA were prepared with the same procedure, except that 1.7 ml of a solution of MPLA in chloroform at 1 mg/ml was added to the chloroform solutions of DPPC and DCChol before evaporation and formation of the lipid film. Liposomes were formulated with antigen to final concentration of 8 mg/ml lipid and 20 µg/ml FMDV 146S antigen ~~(FMDV A Iran96, kindly provided by Merial Ltd, Pirbright, UK)~~ using a HEPES/Sucrose buffer under stirring.

Vaccine characterization

The liposome products were characterized immediately after preparation by measuring pH, average particle size, polydispersion, zeta potential (light scattering, Zetasizer Nano ZS, Malvern), and lipid component concentration (RP-HPLC, Agilent). In addition, liposomes were assessed by transmission electron microscopy after negative staining. A droplet of the liposome suspension diluted 1:5 in buffer was placed on a collodion/carbon-coated 300-mesh copper grid (Electron Microscopy Sciences, Hatfield, PA). After waiting 1 min to permit adsorption, excess liquid was blotted with a filter paper and a drop of 2% phosphotungstic acid at pH 7.0 was added. After another 45 s, the negative stain was removed by touching a filter paper to the edge of the grid. Samples were dried overnight at RT and examined with a Philips CM12 transmission electron microscope (FEI, The Netherlands) at an accelerating voltage of 80 kV with primary magnifications ranging from ×25,000 to ×110,000. Micrographs were captured with a Mega View III camera using the iTEM software version 5.2 (Olympus Soft Imaging Solutions GmbH, Münster, Germany). For the data see Supplementary Fig. 1.

Immunization of sheep

Three groups of six Skudden sheep were immunized intramuscular with 2 ml vaccine dose containing 146S antigen in PBS (PBS), antigen formulated in liposomes (L), or antigen formulated in liposomes together with MPLA/Gardiquimod as TLR ligands (L(TLRL)). ~~A power calculation to determine the number of animals was performed. We assumed to find an increase of antibodies by 90% with a power of $1-\beta = 0.8$ (80% security) a group size of six would be enough, estimating a SE of lower than 40%.~~ An

error α of 0.05 and an error β of 0.2 was employed. Each group was composed of three young (5–6 month of age) and three older (1–2 years of age). Serum and PBMC samples were taken previous to vaccination and on day 3, 14, and 28. The animal experiment was performed in accordance to the Article 18 of the Swiss Animal Welfare Act (TSchG) and was approved by the Cantonal Ethical Committee for animal experiments.

Serum neutralization assay

Clot activator tubes (Becton Dickinson) were used to sample serum, which was centrifuged at 1000 × *g* and stored at − 20 °C until analysis. Serum was heat-inactivated at 56 °C for 30 min. Twofold serial dilutions of serum starting with 1:10 were performed in 50 µl final volume. Fifty microliters of FMDV A Iran96 at 100 $TCID_{50}$ was added to each well and incubated at 37 °C 5% CO_2 for 1 h. Samples of vaccinated cattle were used for standardization purpose. LFBK-αVβ6 cells[26] (kindly obtained from Dr. Luis Rodriguez, Plum Island Animal Disease Center, New York, USA) were added to wells in suspension (100 µl at 2×10^5 cells/ml) and incubated for 72 h. Cytopathic effect was used for readout. The Reed and Muench formula was used for calculation of neutralizing titer.[27] Statistical analyses for differences in antibody responses used two-way analysis of variance (ANOVA) combined with Tukey's multiple comparison test.

Haptoglobin

Serum haptoglobin was measured using a the Tridelta PHASE haptoglobin assay (Tridelta Development Ltd, Ireland). Statistical analyses for differences in haptoglobin responses used two-way ANOVA combined with Tukey's multiple comparison test.

Isolation of PBMC RNA

Blood was collected in EDTA-coated tubes (Becton Dickinson) and PBMCs were isolated by density centrifugation (1000 × *g*, 25 min) over Ficoll-Paque (GE Healthcare, UK) using citrated blood. Five million PBMCs were centrifuged at 350 × *g* and the pellet was resuspended in 1 ml TRIzol(R) (Thermo Fisher). RNA was isolated using the NucleoSpin RNA kit (Macherey-Nagel, Germany). Chloroform:IAA (0.2 ml; 49:1) was added per ml of TRIzol and tubes were shaken vigorously for 15 s followed by incubation for 3 min at RT. Then, the tubes were centrifuged at 12,000 × *g*, 4 °C for 15 min and the aqueous phase was transferred to new tubes and mixed with 500 µl isopropanol. After incubation for 10 min at RT, the samples were loaded on to NucleoSpin RNA columns and centrifuged at 11,000 × *g* for 30 s. MDB (350 µl) was added to columns followed by centrifugation at 11,000 × *g* for 30 s. DNase mixture (95 µl) was applied on the membranes for 20 min followed by washing with RAW2 buffer and centrifugation as above. This was repeated twice with 600 µl and 250 µl RA3 buffer, respectively. RNA was eluted with 40 µl RNase-free water and centrifugation at 11,000 × *g* for 1 min. Samples were stored at − 70 °C until analysis.

RNA-seq data analysis

Between 2.9 and 22.5 million read pairs were obtained per sample and the quality of the reads was assessed using fastqc v.0.11.2 (http://www.bioinformatics.babraham.ac.uk/projects/fastqc/). Ribosomal RNA (rRNA) was removed by mapping the reads to a collection of rRNA sequences (ensembl release 3.1) with Bowtie2 v. 2.2.1.[28] The remaining reads were mapped to the Ovis aries reference genome (Oar_v3.1) with Tophat v.2.0.13.[29] We used htseq-count v.0.6.1[30] to count the number of reads overlapping with each gene, as specified in the ensembl annotation (Oar_v3.1.86). The Bioconductor package DESeq2 v. 1.10.1[31] was used to test for differential gene expression between the different time points and vaccines. Detailed information about the genes including the Entrez Gene ID, the description of the gene, the human homolog ensembl gene ID and the human homolog gene name was obtained using the Bioconductor package BioMart v. 2.26.1.[32]

BTM analyses

To further analyze the molecular signatures of the response to different vaccines, we performed a GSEA using blood transcription modules defined by Li et. al.[1] These modules are composed of 9 up to 347 different genes. The GSEA tool (software.broadinsititute.org)[33] was used to calculate enrichment scores for BTMs with each individual animal. Pearson's correlation of enrichment scores with antibody or haptoglobin levels was calculated in R. GSEA was also used to compare to compare the different vaccines. To this end, a grouped GSEA was performed. To analyze difference in high and low antibody responders, animals in each vaccine group having an SNT at day 28 of > 50% above the median were defined as "high responders" ($n = 6$) and those with < 50% of the median as "low responders" ($n = 6$). Two animals per vaccine group fitted into each these criteria. Then GSEA was used to compare BTM levels found in high and low responders. Heatmaps, scatter plots, and polar plots were created using the ggplot 2 package or Graphpad Prism 7.0.

DATA AVAILABILITY

The raw sequence data was uploaded to the European Nucleotide Archive accession number PRJEB26387. Other data sets are available in the Supplementary Data. Raw flow cytometry data are available from the corresponding author upon reasonable request.

ACKNOWLEDGEMENTS

This work was supported by the Swiss Federal Food Safety and Veterinary Office FVSO grant 1.13.05. We thank Hans-Peter Lüthi and Roman Troxler for care of the animals and regular bleeding; Muriel Fragnière and Tosso Leeb for sequencing support; and Irene Keller for bioinformatics advice.

AUTHOR CONTRIBUTIONS

R.O.B.: design of the study, data acquisition and analysis, and drafting the manuscript. L.B.: vaccine preparation, data acquisition, and analysis. K.W.: design of the study and bioinformatic analysis. G.A., O.G.N., S.P., B.Z., and V.G.: data acquisition and data analysis. M.S.: data analysis and critically reviewing the manuscript. N.C., C.B., and R.B.: design of the study and data analysis. A.S.: design of the study, data analysis, and finalizing manuscript

ADDITIONAL INFORMATION

Supplementary information accompanies the paper on the *npj Vaccines* website (https://doi.org/10.1038/s41541-018-0078-0).

Competing interests: The authors declare no competing interests.

Publisher's note: Springer Nature remains neutral with regard to jurisdictional claims in published maps and institutional affiliations.

REFERENCES

1. Li, S. et al. Molecular signatures of antibody responses derived from a systems biology study of five human vaccines. *Nat. Immunol.* **15**, 195–204 (2014).
2. Nakaya, H. I. et al. Systems biology of vaccination for seasonal influenza in humans. *Nat. Immunol.* **12**, 786–795 (2011).
3. Querec, T. D. et al. Systems biology approach predicts immunogenicity of the yellow fever vaccine in humans. *Nat. Immunol.* **10**, 116–125 (2009).
4. Obermoser, G. et al. Systems scale interactive exploration reveals quantitative and qualitative differences in response to influenza and pneumococcal vaccines. *Immunity* **38**, 831–844 (2013).
5. Patil, P. K. et al. Early antibody responses of cattle for foot-and-mouth disease quadrivalent double oil emulsion vaccine. *Vet. Microbiol.* **87**, 103–109 (2002).
6. Doel, T. R. Natural and vaccine induced immunity to FMD. *Curr. Top. Microbiol. Immunol.* **288**, 103–131 (2005).
7. Kasturi, S. P. et al. Programming the magnitude and persistence of antibody responses with innate immunity. *Nature* **470**, 543–547 (2011).
8. Ferguson, N. M., Donnelly, C. A. & Anderson, R. M. The foot-and-mouth epidemic in Great Britain: pattern of spread and impact of interventions. *Science (New York)* **292**, 1155–1160 (2001).
9. McFadden, A. M. et al. Epidemiology of the 2010 outbreak of foot-and-mouth disease in Mongolia. *Transbound. Emerg. Dis.* **62**, e45–51 (2015).
10. Ramanoon, S. Z., Robertson, I. D., Edwards, J., Hassan, L. & Isa, K. M. Outbreaks of foot-and-mouth disease in Peninsular Malaysia from 2001 to 2007. *Trop. Anim. Health Prod.* **45**, 373–377 (2013).
11. Quaye, I. K. Haptoglobin inflammation and disease. *Trans. R. Soc. Trop. Med. Hyg.* **102**, 735–742 (2008).
12. Eckersall, P. D. et al. Maternal undernutrition and the ovine acute phase response to vaccination. *BMC Vet. Res* **4**, 1 (2008).

13. Auray, G. et al. Characterization and transcriptomic analysis of porcine blood conventional and plasmacytoid dendritic cells reveals striking species-specific differences. *J. Immunol.* **197**, 4791–4806 (2016).
14. Braun, R. O., Python, S. & Summerfield, A. Porcine B cell subset responses to Toll-like receptor ligands. *Front Immunol.* **8**, 1044 (2017).
15. Raetz, C. R. & Whitfield, C. Lipopolysaccharide endotoxins. *Annu Rev. Biochem* **71**, 635–700 (2002).
16. Takeda, K. & Akira, S. TLR signaling pathways. *Semin Immunol.* **16**, 3–9 (2004).
17. Enkhbaatar, P. et al. Comparison of gene expression by sheep and human blood stimulated with the TLR4 agonists lipopolysaccharide and monophosphoryl lipid A. *PLoS ONE* **10**, e0144345 (2015).
18. Kazmin, D. et al. Systems analysis of protective immune responses to RTS,S malaria vaccination in humans. *Proc. Natl Acad. Sci. USA* **114**, 2425–2430 (2017).
19. Nakaya, H. I. & Pulendran, B. Vaccinology in the era of high-throughput biology. *Philos. Trans. R Soc. Lond. B Biol. Sci.* **370**, pii: 20140146 https://doi.org/10.1098/rstb.2014.0146 (2015).
20. Nakaya, H. I. et al. Systems biology of immunity to MF59-adjuvanted versus nonadjuvanted trivalent seasonal influenza vaccines in early childhood. *Proc. Natl. Acad. Sci. USA* **113**, 1853–1858 (2016).
21. Nakaya, H. I. et al. Systems analysis of immunity to influenza vaccination across multiple years and in diverse populations reveals shared molecular signatures. *Immunity* **43**, 1186–1198 (2015).
22. Summerfield, A. & Ruggli, N. Immune responses against classical swine fever virus: between ignorance and lunacy. *Front Vet. Sci.* **2**, 10 (2015).
23. Zak, D. E., Tam, V. C. & Aderem, A. Systems-level analysis of innate immunity. *Annu Rev. Immunol.* **32**, 547–577 (2014).
24. Chaussabel, D. & Baldwin, N. Democratizing systems immunology with modular transcriptional repertoire analyses. *Nat. Rev. Immunol.* **14**, 271–280 (2014).
25. Laughlin, R. C., Drake, K. L., Morrill, J. C. & Adams, L. G. Correlative gene expression to protective seroconversion in rift valley fever vaccinates. *PLoS ONE* **11**, e0147027 (2016).
26. ~~LaRocco, M. et al. A continuous bovine kidney cell line constitutively expressing bovine alphavbeta6 integrin has increased susceptibility to foot-and-mouth disease virus. *J. Clin. Microbiol* **51**, 1714–1720 (2013).~~
27. Reed, L. J. & Muench, H. A simple method of estimating fifty per cent endpoints. *Am. J. Epidemiol.* **27**, 493–497 (1938).
28. Langmead, B. & Salzberg, S. L. Fast gapped-read alignment with Bowtie 2. *Nat. Methods* **9**, 357–359 (2012).
29. Kim, D. et al. TopHat2: accurate alignment of transcriptomes in the presence of insertions, deletions and gene fusions. *Genome Biol.* **14**, R36 (2013).
30. Anders, S., Pyl, P. T. & Huber, W. HTSeq–a Python framework to work with high-throughput sequencing data. *Bioinformatics* **31**, 166–169 (2015).
31. Love, M. I., Huber, W. & Anders, S. Moderated estimation of fold change and dispersion for RNA-seq data with DESeq2. *Genome Biol.* **15**, 550 (2014).
32. Durinck, S. et al. BioMart and Bioconductor: a powerful link between biological databases and microarray data analysis. *Bioinformatics* **21**, 3439–3440 (2005).
33. Subramanian, A. et al. Gene set enrichment analysis: a knowledge-based approach for interpreting genome-wide expression profiles. *Proc. Natl Acad. Sci. USA* **102**, 15545–15550 (2005).

Open Access This article is licensed under a Creative Commons Attribution 4.0 International License, which permits use, sharing, adaptation, distribution and reproduction in any medium or format, as long as you give appropriate credit to the original author(s) and the source, provide a link to the Creative Commons license, and indicate if changes were made. The images or other third party material in this article are included in the article's Creative Commons license, unless indicated otherwise in a credit line to the material. If material is not included in the article's Creative Commons license and your intended use is not permitted by statutory regulation or exceeds the permitted use, you will need to obtain permission directly from the copyright holder. To view a copy of this license, visit http://creativecommons.org/licenses/by/4.0/.

© The Author(s) 2018
